# Electrocatalytic Oxidation of Ammonia by (Salen)ruthenium(III) Ammine Complexes: Direct Evidence for a Ruthenium(VI) Nitrido Active Intermediate

**DOI:** 10.1021/jacs.4c16902

**Published:** 2025-04-17

**Authors:** Jianhui Xie, Tingting Yang, Longzhu Hong, Hui Li, Bing Li, Zhenguo Guo, Yingying Liu, Tai-Chu Lau

**Affiliations:** † Anhui Province Key Laboratory of Advanced Catalytic Materials and Reaction Engineering, School of Chemistry and Chemical Engineering, Hefei University of Technology, Hefei 230009, P. R. China; ‡ Institute of Intelligent Machines, 53040 Hefei Institutes of Physical Science, Chinese Academy of Sciences, Hefei 230031, P. R. China; § Department of Chemistry, City University of Hong Kong, Kowloon Tong 999077 Hong Kong, P. R. China

## Abstract

The electrocatalytic oxidation of ammonia using molecular catalysts has attracted much attention recently due to its potential for fuel cell applications. In this study, we report the electrocatalytic ammonia oxidation (AO) by [Ru^III^(salchda)­(NH_3_)­(CH_3_CN)]^+^ (**RuNH**_**3**_, salchda = *N,N*′-bis­(salicylidene)-*o*-cyclohexyldiamine dianion) and its bromo derivative. Controlled potential electrolysis at 0.65 V versus Fc^+/0^ for 3.2 h of a solution of **RuNH**_**3**_ and NH_3_ in CH_3_CN produced N_2_ with a TON of 26 and Faradaic efficiency (FE) close to 100%. The TON was increased to 79 and 147 when electrolysis was carried out at 0.7 and 0.80 V vs Fc^+/0^, respectively, with FE maintained at >99%, which are the highest among molecular ruthenium catalysts. An active intermediate was detected and shown to be the corresponding ruthenium­(VI) nitrido complex [Ru^VI^(salchda)­(N)]^+^ (**RuN**) by direct comparison with an authentic sample of **RuN**, which we have previously synthesized and fully characterized. Direct kinetic studies on the oxidation of NH_3_ to N_2_ have also been carried out and the results are consistent with parallel electrophilic attack of NH_3_ by **RuN** and bimolecular N···N coupling of **RuN** to produce N_2_. DFT calculations have also been performed to support the proposed mechanism.

## Graphical abstract

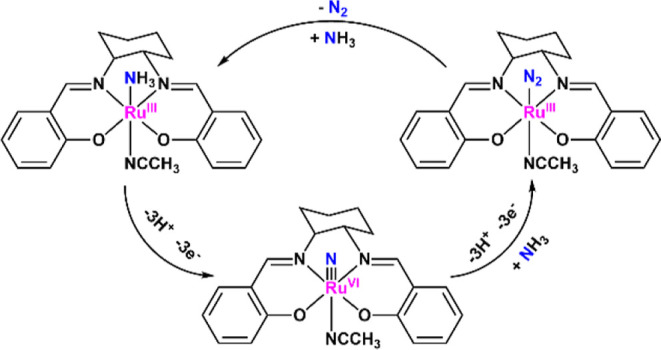

## Introduction

The electrocatalytic oxidation of NH_3_ to N_2_ has attracted much interest in recent years, mainly because of its potential applications in ammonia fuel cells. The use of ammonia as a carbon-free fuel is appealing since it has high energy density, is nonexplosive, and can be readily transported in liquid form.^1–3^ Ammonia is also readily available via its production from the Haber–Bosch process.

The thermodynamic potential for NH_3_ oxidation to N_2_ is relatively low (+0.092 V vs NHE at pH = 0),^4^ but since this is a multiple proton-coupled electron transfer process, high energy intermediates are involved.^5^ Hence, development of selective and efficient catalysts for the oxidation of NH_3_ to N_2_ at low overpotentials remains a significant challenge.^6^

A number of heterogeneous electrocatalysts, including those based on metals, metal alloys, metal hydroxides, and metal nitrides, have been reported,^7^ but they often suffer from surface poisoning by strongly adsorbed nitrogen species or exhibit low activity.^8^ Recently, there has been growing interest in the design of molecular complexes for electrocatalytic ammonia oxidation (AO) in homogeneous solutions; these include complexes of Ru,^9–17^ Fe,^18–20^ Mn,^21^ Cu,^22,23^ and Ni.^24^ Molecular catalysts usually have higher catalytic activity compared with solid-state materials, and their catalytic activity can be readily tuned by suitable modifications of the ligands. In addition, their stability can be enhanced by anchoring onto various solid supports.^25^

We report herein ruthenium­(III) complexes bearing salen-type ligands,^26,27^ [Ru^III^(salchda)­(NH_3_)­(CH_3_CN)]^+^ (**RuNH**_**3**_, salchda = *N,N*′-bis­(salicylidene)-*o*-cyclohexyl-diamine dianion) and its bromo derivative (^**Br**^ **RuNH**_**3**_), as highly efficient catalysts for the oxidation of NH_3_ to N_2_, with a turnover number (TON) up to 147 and Faradaic efficiency close to 100%, which are the highest among molecular ruthenium catalysts. Metal nitrido (M≡N), imido (M=NH), or amido (M–NH_2_) species have often been invoked as active intermediates in AO by molecular metal catalysts.^9–24^ However, such intermediates have not been isolated or fully characterized. In the present case of AO by **RuNH**_**3**_, we have detected an active intermediate which was shown to be the corresponding ruthenium­(VI) nitrido complex (Figure S1), [Ru^VI^(salchda)­(N)]^+^ (**RuN**), by direct comparison with an authentic sample of **RuN**. We have previously isolated and fully characterized **RuN** ^28^ and have shown that it is highly electrophilic and reacts readily with a variety of organic substrates (Scheme S1), such as amines,^29,30^ isocyanide,^31^ phenol,^32^ alkenes,^33^ alkynes,^34^ and alkanes.^35^ In this work, we have also carried out direct kinetic and mechanistic studies on the oxidation of NH_3_ by **RuN** by UV/vis spectrophotometry. The results are consistent with the parallel electrophilic attack of NH_3_ by **RuN** and bimolecular N···N coupling of **RuN** to produce N_2_. DFT calculations were also performed to support the proposed mechanism.

## Results and Discussion

[Ru^III^(salchda)­(NH_3_)­(CH_3_CN)]^+^ (**RuNH**_**3**_, Figure 1a) was prepared by the reaction of [Ru^VI^(N)­(salchda)­(CH_3_OH)]^+^ with thiophenol, as described in the literature.^36^ The electrospray ionization mass spectrometry (ESI/MS) of **RuNH**_**3**_ in CH_3_CN shows the parent peak at *m*/*z* = 480 (Figure S2). The bromo analogue, [Ru^III^(Br_2_-salchda)­(NH_3_)­(CH_3_CN)]^+^ (^**Br**^ **RuNH**_**3**_), which bears two bromo substituents *para* to the phenoxy moiety of the salchda ligand, was similarly prepared and its molecular structure was determined by X-ray crystallography (Figure 1b, Table S1). The parent ion can be observed at *m*/*z* = 638 in the ESI/MS (Figure S3). The p*K*_a_ values of **RuNH**_**3**_ and ^**Br**^ **RuNH**_**3**_ were determined to be 25 and 21, respectively (Figures S4 and S5). The smaller p*K*_a_ of ^**Br**^ **RuNH**_**3**_ is attributed to the electron-withdrawing effects of the bromo substituents.

**Figure 1:**
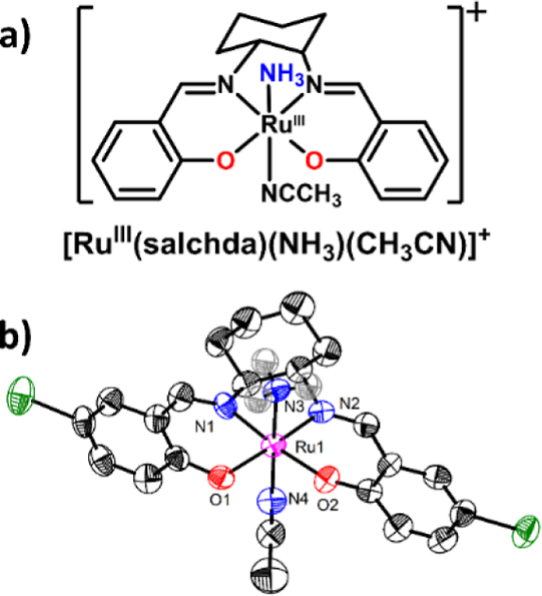
(a) Structure of **RuNH**_**3**_; (b) ORTEP diagram of ^**Br**^ **RuNH**_**3**_; thermal ellipsoids are drawn at 50% probability (H atoms were omitted for clarity except N3–H). The Ru1–N3 bond distance is 2.097(6) Å.

### Cyclic Voltammetry of RuNH_3_ and ^Br^RuNH_3_

The cyclic voltammetry (CV) of **RuNH**_**3**_ (Figure 2a) shows a Ru^IV/III^ couple at a *E*_1/2_ of 0.52 V; a Ru^III/II^ couple is also found at a *E*_1/2_ of −0.82 V (Figure S6). All potentials are versus Fc^+/0^, unless otherwise stated. Upon the addition of excess 2,4,6-collidine (0.01 M), an additional wave with a higher current appears at ca. 0.45 V (Figure 2a, blue line). An irreversible wave is also found at −0.67 V (Figure S7), which is at the same potential as the **RuN** ^**VI/V**^ reduction wave (Figure S8).^28^ The wave at 0.45 V is assigned to the three-electron oxidation of **RuNH**_**3**_ to **RuN**: **Ru** ^**III**^–**NH**_**3**_–3H^+^–3e^–^ → **Ru** ^**VI**^≡**N**. A similar result was obtained using 2,6-lutidine as the base instead of 2,4,6-collidine. As shown in Figure 2a, the CV of **RuNH**_**3**_ upon adding excess NH_3_ shows a catalytic current with an onset potential of ca. 0.23 V vs Fc^+/0^ (Figure 2a, red line). The catalytic wave should arise from oxidation of NH_3_ by **RuN**, with the potential shifted cathodically to ca. 0.35 V, consistent with the presence of a higher concentration and the stronger base NH_3_, which lowers the potential for the three-electron oxidation of **RuNH**_**3**_. A similar catalytic current was also observed with **RuN** in the presence of excess NH_3_ (Figure 2b). These results indicate that catalytic AO occurs with the initial oxidation of **RuNH**_**3**_ to form **RuN**.

**Figure 2:**
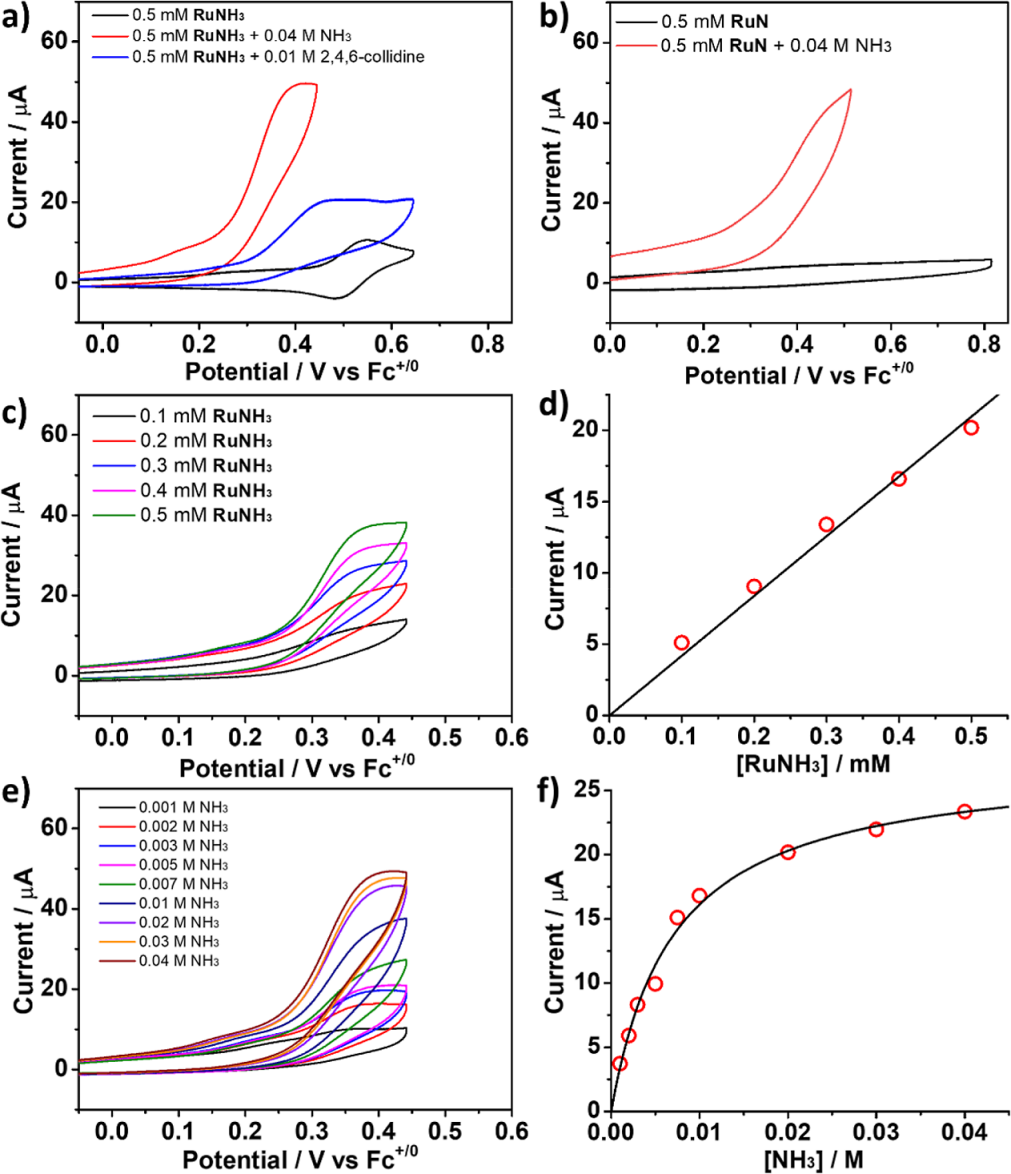
(a) CV of **RuNH**_**3**_ (0.5 mM) (black) and in the presence of 2,4,6-collidine (0.01 M) (blue) or NH_3_ (0.04 M) (red). (b) CV of **RuN** (0.5 mM) (black) and after addition of NH_3_ (0.04 M) (red). (c) CV in CH_3_CN of 0.01 M NH_3_ and 0.1 M [^*n*^Bu_4_N]­PF_6_ under various **[RuNH**_**3**_]. (d) Plot of the current density at *E*_1/2_ vs [**RuNH**_**3**_]. (e) CV in CH_3_CN of 0.5 mM **RuNH**_**3**_ and 0.1 M [^*n*^Bu_4_N]­PF_6_ under various [NH_3_]. (f) Plot of the current density at *E*_1/2_ vs [NH_3_]. Scan rate: 0.1 V/s.

A small current increase was observed at 0.15 V (Figure S9a), which is attributed to the presence of a small amount of the diammine complex [Ru^III^(salchda)­(NH_3_)_2_]^+^ (**Ru­(NH**_**3**_ **)**_**2**_), which has been independently prepared and characterized as the PF_6_^–^ salt (see Supporting Information). The CV of **Ru­(NH**_**3**_ **)**_**2**_ shows a oxidation wave at the same potential of 0.15 V (Figure S9c), which is assigned to oxidation to the Ru­(IV) amido species, [Ru­(salchda)­(NH_2_)­(NH_3_)]^+^; such an assignment is supported by DFT calculations described below. There is also a catalytic current attributed to AO, but it occurs at a potential that is anodically shifted relative to that of **RuNH**_**3**_ (Figure S9d). In CH_3_CN containing 0.1 M NH_3_, **RuNH**_**3**_ was found to undergo slow substitution of the CH_3_CN ligand by NH_3_ to give **Ru­(NH**_**3**_ **)**_**2**_, with a pseudo-first-order rate constant, *k*_obs_, of (2.8 ± 0.3)×10^–4^ s^–1^ at 25.0 °C, as monitored by UV/vis spectrophotometry (Figure S10). Around 14 ± 1% of **Ru­(NH**_**3**_ **)**_**2**_ was formed after 1.5 h (Figure S11). Substitution of NH_3_ by CH_3_CN also occurred when **Ru­(NH**_**3**_ **)**_**2**_ was added to the CH_3_CN solution of 0.1 M NH_3_, with a *k’*_obs_ of (1.7 ± 0.1) × 10^–3^ s^–1^ (Figure S12), resulting in a similar equilibrium concentration of **Ru­(NH**_**3**_ **)**_**2**_ of ca. 14%. From the equilibrium constant *K* = *k*_obs_/*k*′_obs_ = 0.16, the percentage of [**Ru­(NH**_**3**_ **)**_**2**_]_eq_ was also calculated to be 14%. We conclude from our results that the observed catalytic activity in Figure 2 is mainly attributed to **RuNH**_**3**_. The catalytic current by **RuNH**_**3**_ exhibits a linear dependence on [**RuNH**_**3**_], indicating that the reaction is first-order with respect to [**RuNH**_**3**_] (Figure 2c,d). As the concentration of NH_3_ increases, the catalytic current also rises, eventually reaching saturation (Figure 2e,f). The foot of the wave analysis (FOWA) was performed for all CVs, and the plot of *i*/*i*_p_ versus 1/{1 + exp­[F × (*E*_cat_° – *E*)/*RT*]} shows a linear relationship (Figure S13). The *k*_obs_ (ca. 0.045 s^–1^ at 10 mM NH_3_) calculated from the FOWA equation gives a rate constant, *k*′_obs_, of 4.5 M^–1^s^–1^.

**^Br^RuNH_3_** exhibits a CV profile similar to that of **RuNH_3_**, showing a Ru^IV/III^ couple at 0.68 V (Figure S14a, black line). A Ru^III/II^ couple is observed at −0.65 V (Figure S15). Again, in the presence of 0.01 M 2,4,6-collidine, an irreversible oxidation peak is observed at 0.59 V (Figure S14a, red line), also attributed to the three-electron oxidation of **^Br^RuNH_3_** to **^Br^Ru^VI^≡N**. An irreversible reduction wave is also found at −0.49 V (Figure S16), at the same potential as ^B^ **^r^RuN^VI/V^** (Figure S17). These findings suggest that the oxidation of **^Br^RuNH_3_** in the presence of 2,4,6-collidine also affords the **^Br^RuN** species. When the CV of **^Br^RuNH** **_3_** was carried out in the presence of NH_3_, a catalytic current occurred with the onset potential of ca. 0.4 V (Figure S14b, red line). Compared to **RuNH**_**3**_, the potentials of **^Br^RuNH**_**3**_ are higher, which is attributed to the electron-withdrawing effect of the bromo substituents. The catalytic current displays a linear dependence on [**^Br^RuNH**_**3**_] (Figure S18a,b), but saturation occurs with increasing [NH_3_] (Figure S18c,d). The plot of *i*/*i*_p_ versus 1/{1 + exp­[F×(*E*_cat_° – E)/*RT*]} is linear and *k*_obs_ (ca. 0.37 s^–1^ at 50 mM NH_3_) affords the rate constant, *k*′_obs_, of 7.4 M^–1^ s^–1^ (Figure S19).

### Controlled Potential Electrolysis (CPE)

CPE of a solution of **RuNH**_**3**_ (0.1 mM) and NH_3_ (0.2 M) in CH_3_CN was carried out at an applied potential of 0.65 V under an argon atmosphere. After 3.2 h of electrolysis, a total charge of 10.8 C was recorded (Figure S20). Gas chromatographic (GC) analysis of the headspace gas revealed the production of 18.5 μmol of N_2_, corresponding to a turnover number (TON = mol of N_2_/mol of **RuN**) of 26 and a Faradaic efficiency (FE) of 99%. H_2_ (40.5 μmol) was also detected, with a TON of 58 and a FE of 73% (Figure S21). This reduced FE/stoichiometric yield of H_2_ compared to N_2_ in catalytic NH_3_ oxidation has been observed in previous studies, where the ratio of N_2_:H_2_ is around 1:2.5.^18,20^ This is most likely due to some escape of H_2_ from the cell, since it is the lightest molecule. At a higher applied potential of 0.7 V, electrolysis for 3 h afforded a total charge of 32.4C, with 55.4 μmol of N_2_ produced, corresponding to a TON of 79 and a FE of 99%. 127.7 μmol of H_2_ was also produced with TON 182 (Figure S22). When the electrolysis was carried out at an even more positive potential of 0.8 V, the total charge reached 59.7 C after 3 h. GC analysis indicated 103.2 μmol N_2_ and 238.2 μmol H_2_ were generated and the TON of N_2_ increased to 147 with again nearly 100% FE (Figure S23). The FE is the highest among molecular catalysts; the TON is also among the highest among ruthenium catalysts (Table 1) and is close to the value of 149 (48 h) for the iron catalyst [(bpyPy_2_Me)­Fe­(MeCN)_2_]^+^.^19^ We also investigated the working electrode after CPE at 0.8 V by measuring CV. As shown in Figure S24, the glassy carbon plate afforded no redox peaks in CH_3_CN. In the presence of 0.2 M NH_3_, the current is still very small (ca. 0.2 mA at 0.8 V vs Fc^+/0^) compared to the currents of the CPE experiments in the presence of Ru catalysts. These results suggest that no active species were adsorbed on the working electrode.

**Table 1:** Selected Electrochemical and Catalytic Data for Related Catalysts for Ammonia Oxidation to Dinitrogen^a^

catalyst	solvent	*E*_onset_/V	*E*_CPE_/V	FE (N_2_) %	TON (duration) h	ref
**RuNH**_**3**_	CH_3_CN	0.23	0.65	99	26 (3.2)	this work
			0.70	99	79 (3)	
			0.80	100	147 (3)	
^**Br**^ **RuNH**_**3**_	CH_3_CN	0.4	0.65	99	25 (3.2)	
			0.80	98	69 (3)	
[Ru(bda)(py)_2_]^2+^	CH_3_CN	0.2				^12^
[Ru(NH_3_)(trpy)(dmabpy)]^2+^	THF	0.68^b^	0.73^b^	86	2 (3)	^13^
[Ru(tda-κ-N^3^O)(py)_2_]	CH_3_CN	1.05^b^	1.40^b^	74	7.5 (11)	^14^
Ru_2_(chp)_4_OTf	CH_3_CN	–0.26	0.0	52	5 (0.5)	^15^
[Ru^II^(H_2_L)(pic)_2_]^2+^	CH_3_CN	–0.02	0.15	96		^16^
[Fe(NH_3_)_2_(TPA)]^+^	CH_3_CN	0.70	1.10	80	16 (18)	^18^
[(bpyPy_2_Me)Fe(MeCN)_2_]^+^	CH_3_CN	0.45	0.85	87	149 (48)	^19^
[Cp*Fe(1,2-Ph_2_PC_6_H_4_NH)(NH_3_)]^+^	THF	–0.04	0.22	74	4.4 (8)	^20^
Mn(salen)	CH_3_CN	0.66	0.86	96	6.6 (18)	^21^
[^*i*^Pr_2_NN_F6_]Cu^I^–NH_3_	CH_3_CN	–0.24	0.0	84	18.2 (26.5)	^22^

a Potentials are versus Fc^+/0^, unless otherwise stated.

b Potentials are versus NHE.

CPE of **RuN** at 0.8 V was also carried out and afforded a total charge of 34.5 C after 3 h (Figure S25). GC analysis showed that 58.3 μmol N_2_ and 134.2 μmol H_2_ were generated (Figure S26), corresponding to a N_2_ TON of 83 and a 98% FE. The lower TON and FE for N_2_ is attributed to some decomposition of the **RuN**.^37^

The results for the electrolysis of ^**Br**^ **RuNH**_**3**_ at 0.65 V are similar to that of **RuNH**_**3**_, with a TON of 25 for N_2_ and a Faradaic efficiency of 99%. H_2_ (38.7 μmol) was formed with a Faradaic efficiency of 72% (Figures S27 and S28). When the electrolysis was carried out at 0.8 V, a total charge of 28.6 C was achieved after ca. 3 h, and GC analysis showed that 48.4 μmol of N_2_ and 106.6 μmol of H_2_ were generated. The TON of N_2_ is 69 with 98% FE (Figure S29).

Control experiments indicated that only ca. 0.024 C was obtained in electrolysis without **RuNH**_**3**_ or ^**Br**^ **RuNH**_**3**_ (Figure S30).

Electrolysis was also conducted using **RuNH**_**3**_ and ^15^NH_3_ in CH_3_CN for 2300s (Figure S31). Analysis by GC–MS revealed the formation of ^14^N^15^N and ^15^N^15^N in a ratio of 1:14, with nearly no ^14^N^14^N detected (Figure S32). These results indicate that N_2_ are derived from the oxidation of NH_3_.

In order to obtain more information about the mechanism of AO, we carried out the oxidation of **RuNH**_**3**_ by tris­(4-bromophenyl)­aminium hexachloroantimonate ([(*p*-BrC_6_H_4_)_3_N]­[SbCl_6_], “Magic Blue”). The ESI/MS after addition of [(*p*-BrC_6_H_4_)_3_N]­[SbCl_6_] to **RuNH**_**3**_ shows a peak at *m*/*z* = 480, which corresponds to **RuNH**_**3**_, while the peak at *m*/*z* = 436 is attributed to the Ru­(VI) nitrido species, [Ru^VI^(salchda)­(N)]^+^ (**RuN**) (Figure 3). There is also a peak at *m*/*z* = 504 due to [Ru^III^(salchda)­(CH_3_CN)_2_]^+^ which should arise from N···N coupling of the **RuN**.^28^ These results show that **RuNH**_**3**_ can be readily oxidized to **RuN**, in line with the CV results. When catalytic AO was carried out using 0.32 mM **RuNH**_**3**_, 200 mM NH_3_, and 32 mM Magic Blue, N_2_ was produced with a TON of 8 (48% yield). No other nitrogen-containing products were detected. When **RuN** was used as the catalyst instead of **RuNH**_**3**_, a N_2_ TON of 9 was obtained. The relatively low TONs and yields of N_2_ were also observed in other catalytic AO systems, which are in part due to the instability of Magic Blue.^16^ Investigation of the chemical AO solution of **RuNH**_**3**_ and NH_3_ by ESI/MS indicated the formation of **RuN** at *m*/*z* = 436 (Figure S34). Furthermore, use of ^15^NH_3_ instead of ^14^NH_3_ gave a peak at *m*/*z* = 437 in the ESI/MS which is attributed to **Ru** ^**15**^ **N** species, [Ru^VI^(salchda)­(^15^N)]^+^ (Figure S35). These results show that **RuN** is a key intermediate in the catalytic oxidation of NH_3_.

**Figure 3:**
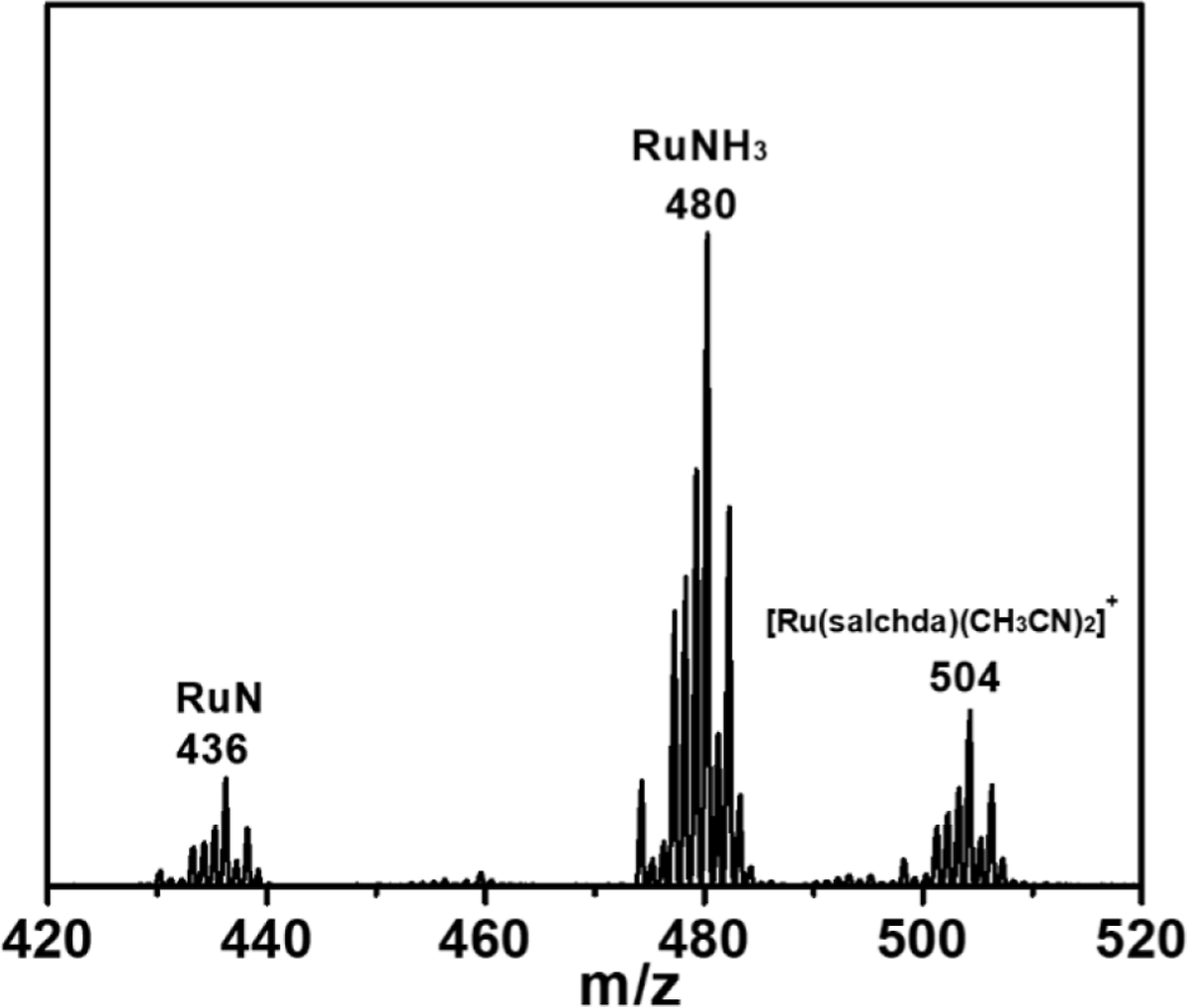
ESI/MS spectrum of the reaction of **RuNH**_**3**_ (1 mM) with [(*p*-BrC_6_H_4_)_3_N]­[SbCl_6_] (3 mM) in the presence of 2,4,6-collidine (10 mM) in CH_3_CN.

### Kinetics of the Reactions of RuN with NH_3_

Since **RuN** is identified as the active intermediate, we then directly studied the kinetics of the reaction between **RuN** and NH_3_ by UV/vis spectroscopy (Figure 4). Repetitive scanning of **RuN** with excess NH_3_ in CH_3_CN indicated rapid increase in absorbance at around 345 and 715 nm, consistent with the formation of **RuNH**_**3**_ (λ_max_ = 680 nm, Figure S36) and/or [Ru^III^(salchda)­(CH_3_CN)_2_]^+^ (λ_max_ = 735 nm, Figure S37). ESI/MS of the final solution indicated the presence of a mixture of the ruthenium­(III) complexes.

**Figure 4:**
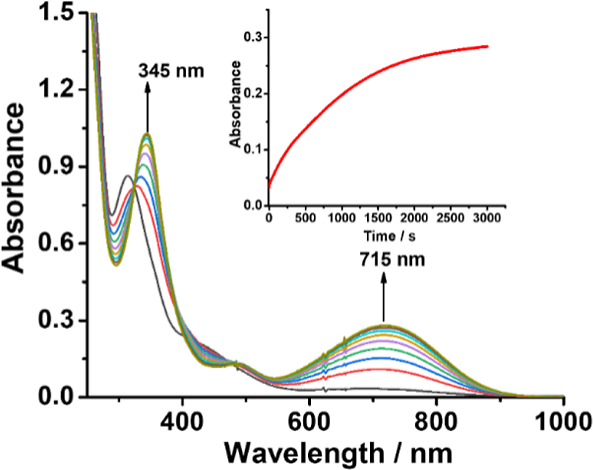
UV/vis spectral changes at 300s intervals of the reaction between **RuN** (6.0 × 10^–5^ M) and NH_3_ (2.0 × 10^–3^ M) in CH_3_CN at 25 °C. The inset shows the absorbance–time trace at 700 nm.

Since the increase in absorbance at 345 or 715 nm did not follow clean pseudo-first-order kinetics, the initial rates (*k*_int_) at various [**RuN]** and [NH_3_] were evaluated. Plots of *k*_int_/[**RuN**] vs [**RuN**] (Figure 5a) and *k*_int_ vs [NH_3_] (Figure 5b) are linear, consistent with the rate law shown in eq 1.

1 $$-\frac{d\left[Ru\right]}{dt}=k_{a}\left[Ru\right]\left[NH_{3}\right]+k_{b}{\left[Ru\right]}^{2}$$

*k*_a_ and *k*_b_ were found to be 0.353 ± 0.10 M^–1^s^–1^ and 9.14 ± 0.32 M^–1^s^–1^, respectively, at 25.0 °C. The *k*_a_ pathway is attributed to electrophilic attack of **RuN** on [NH_3_], similar to its reaction with aliphatic amines.^28,30^ The *k*_b_ pathway involves the N···N coupling of **RuN** (Scheme 1).^28,37^

**Figure 5:**
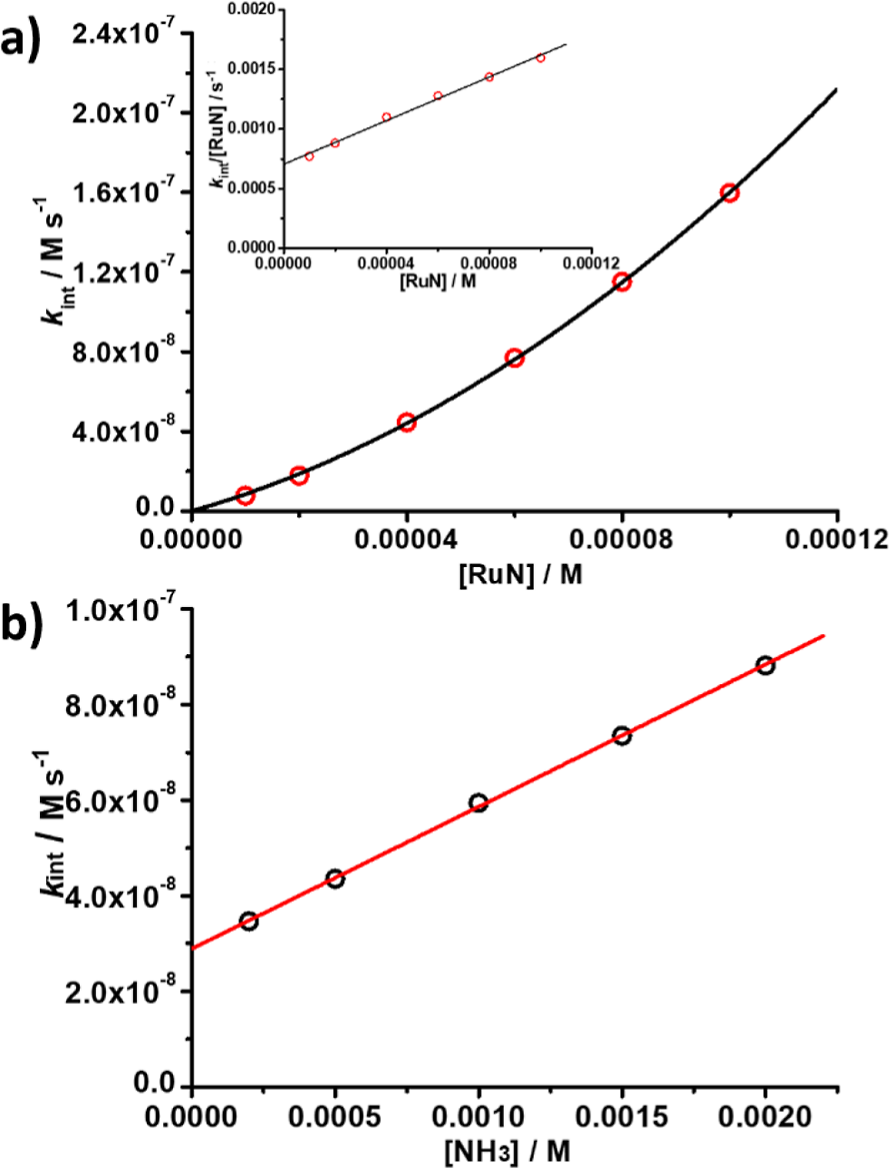
(a) Plot of initial rate *k*_int_ vs [**RuN**] for the reaction of **RuN** with NH_3_ (2.0 × 10^–3^ M) in CH_3_CN at 25 °C. The inset is the corresponding plot of initial rate *k*_int_/**[RuN**] vs [**RuN**]. Slope = 9.14 ± 0.32, *y* intercept = (7.06 ± 0.20) × 10^–4^, *r* ^2^ = 0.99. (b) Plot of *k*_int_ vs [NH_3_]. Slope = (2.97 ± 0.03) × 10^–5^, *y* intercept = (2.89 ± 0.04) × 10^–8^, *r* ^2^ = 0.9995.

**Scheme 1:**
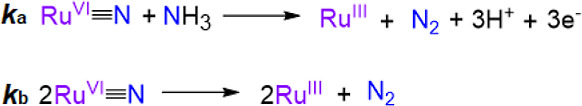
Reactions of RuN in the Presence of NH_3_

The kinetics were also investigated as a function of temperature, and the activation parameters were obtained from the Eyring plots (Figures S41 and S42), together with the calculated values (see DFT section) listed in Table 2. Notably, the large negative Δ*S* ^‡^ values are indicative of ordered transition states involving two molecules. The much more negative value for pathway *k*_b_ is consistent with two **RuN** molecules in close contact in the transition state. The relatively small enthalpy change (Δ*H* ^‡^) for path *k*_b_ indicates that the N···N coupling pathway does not involve substantial bond breaking/elongation in the transition state.

**Table 2:** Activation Parameters for the Reaction of RuN with NH_3_ in CH_3_CN

	Δ*H* ^‡^/kcal mol^–1^	Δ*S* ^‡^/cal mol^–1^ K^–1^	Δ*G* ^‡^/kcal mol^–1^	Δ*G* ^‡^_calc._/kcal mol^–1^
path *k*_a_	12.8 ± 1.3	–37 ± 8	23.8 ± 3.6	19.6
path *k*_b_	3.1 ± 0.2	–82 ± 1	27.6 ± 0.5	23.6

The effects of various substituents that are *para* to the phenoxy group of the salchda ligand were also investigated. The rate constants *k*_a_ and *k*_b_ for the various complexes are listed in Table S2. Electron-withdrawing substituents on the ligand were found to accelerate the reactions, and a Hammett plot of log *k*_a_ versus σ_p_ gives a linear relationship with a ρ value of 4.9 ± 0.5 (Figure 6). Such a positive ρ value is consistent with electrophilic attack on NH_3_ by **RuN**.^38^ Similar ρ values are also observed in the reaction of anilines with **RuN**.^29^ A ρ value of 3.7 ± 1.6 was obtained in the Hammett plot of log *k*_b_ versus σ_p_, but the data are more scattered (Figure S43).

**Figure 6:**
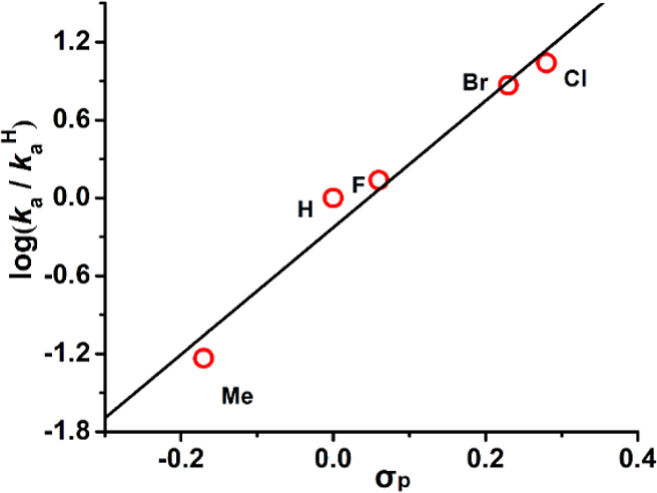
Plot of log­(*k*_a_/*k*_a_^H^) versus σ_p_ for the reaction of **^X^RuN** with NH_3_ in CH_3_CN at 25.0 °C. Slope = (4.9 ± 0.5), *r* ^2^ = 0.96.

The ESI/MS of the reaction of **RuN** with NH_3_ indicates the disappearance of **RuN** and the appearance of [Ru^III^(salchda)­(NH_3_)­(CH_3_CN)]^+^ (*m*/*z* = 480) and [Ru^III^(salchda)­(CH_3_CN)_2_]^+^ (*m*/*z* = 504) (Figure S44), which are consistent with the results from UV/vis spectroscopy (Figure 4). When ^15^NH_3_ was used, [Ru^III^(salchda)­(^15^NH_3_)­(CH_3_CN)]^+^ was detected (Figure S45). For the reaction of **RuN** with ^15^NH_3_, the N_2_ evolved is a mixture of ^15^N^14^N and ^14^N^14^N in the ratio of 1:1.7 (Figure S46), consistent with the ratio of 1:1.8 calculated from the parallel pathways *k*_a_ and *k*_b_ in kinetic studies.

### Mechanism of AO/DFT Calculations

Based on the experimental results, a proposed mechanism for catalytic AO by **RuNH**_**3**_ is shown in Scheme 2. **RuNH**_**3**_ first undergoes 3e^–^/3H^+^ oxidation to give **RuN**. This is followed by parallel electrophilic attack of **RuN** on NH_3_ and N···N coupling. Electrophilic attack on NH_3_ followed by deprotonation produces initially a ruthenium­(IV) hydrazido species, which is then oxidized to Ru­(V). A similar Ru­(IV) hydrazido species has been isolated in the reaction of **RuN** with amines.^28^ The Ru­(V) hydrazido intermediate is further oxidized by 2e^–^/2H^+^ to give Ru^III^–N_2_, which then adds NH_3_ to regenerate **RuNH**_**3**_ with loss of N_2_. On the other hand, N···N coupling of two **RuN** results in the formation of a **Ru**_**2**_ **N**_**2**_ species; each Ru then picks up a NH_3_ to release the N_2_.

**Scheme 2:**
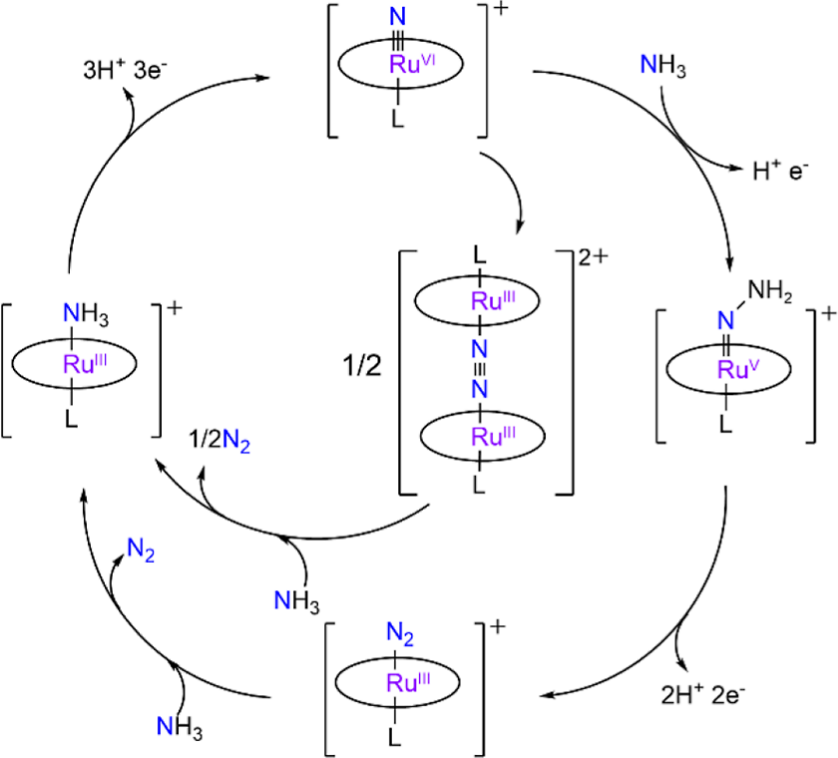
Proposed Catalytic Cycle for AO by RuNH_3_

This proposed mechanism is supported by the DFT calculations. Figure 7 shows the free energy profile for N_2_ formation starting from **RuN**. Two pathways are considered: (a) electrophilic attack of NH_3_ by **RuN** (red line) and (b) coupling of two **RuN** (blue line). In pathway (a), **RuN** first forms an intermediate with NH_3_ (^1^ **[INT2]** ^**+**^), which is then converted to ^1^ **[INT3]** via ^1^ **[TS1]** ^**+**^; this is the rate-limiting step with barrier 19.6 kcal mol^–1^. ^1^ **[INT3]** is formally a ruthenium­(IV) hydrazido species with a Ru–N distance of 1.889 Å and N–N distance of 1.271 Å. ^1^ **[INT3]** then undergoes 1e^–^ oxidation to generate ^2^ **[INT4]** ^**+**^, which is formally a ruthenium­(V) hydrazido species but with partial Ru–N and N–N double-bond character; the Ru–N and N–N distances are 1.938 Å and 1.242 Å, respectively. Loss of 1H^+^ + 1e^–^ from ^2^ **[INT4]** ^**+**^ gives ^1^ **[INT5]** ^**+**^, which is formally a ruthenium­(V) imido species with Ru = N (1.762 Å) and N = N (1.174 Å) bonds. Further loss of 1H^+^ + 1e^–^ from ^1^ **[INT5]** ^**+**^ gives ^2^ **[INT6]** ^**+**^, a ruthenium­(III) dinitrogen complex. Addition of NH_3_ and loss of N_2_ from ^2^ **[INT6]** ^**+**^ produces **RuNH**_**3**_ (^2^ **[INT7]** ^**+**^), which can be readily oxidized to **RuN**.

**Figure 7:**
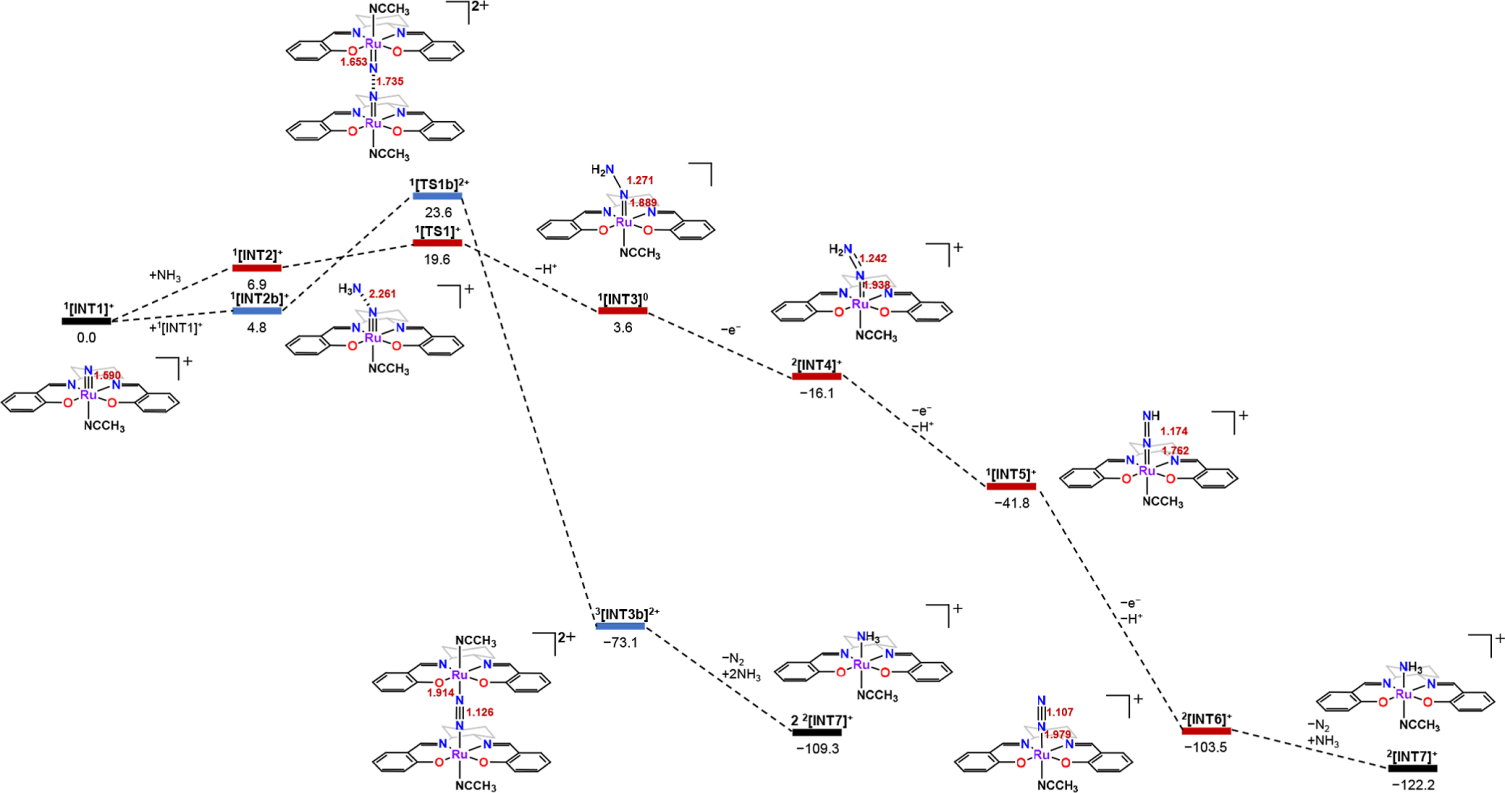
Free energy profile for the N_2_ formation starting from **RuN**. Relative Gibbs free energies at 298 K in CH_3_CN are given in kcal mol^–1^; bond lengths are given in Å.

In pathway (b), two **RuN** molecules first combine to form ^1^ **[INT2b]** ^**+**^. N···N coupling then occurs to produce ^3^ **[INT3b]** ^**2+**^ via ^1^ **[TS1b]** ^**2+**^, with a barrier of 23.6 kcal mol^–1^. ^3^ **[INT3b]** ^**2+**^ is a ruthenium­(III) μ-dinitrogen complex with a N–N distance of 1.126 Å. ^3^ **[INT3b]** ^**2+**^ then undergoes loss of N_2_ and addition of NH_3_ to give **RuNH**_**3**_.

The overall energy barrier of pathway (a) is 4.0 kcal mol^–1^ lower than that of pathway (b) (19.6 vs 23.6 kcal mol^–1^), in agreement with the experimental result (3.8 kcal mol^–1^) that the electrophilic attack pathway is more favorable. However, the difference in barrier is small enough that the predominant pathway may depend on the catalyst concentration. For instance, under chemical oxidation using a high [**RuN**] or [**RuNH**_**3**_], pathway (b) may be preferred. On the other hand, under electrochemical oxidation, [**RuN**] generated at the electrode surface would be small enough so that (a) should be the predominant pathway.

We further investigate the catalytic AOR by the diammine complexes (**Ru­(NH**_**3**_ **)**_**2**_), including the oxidation of **Ru­(NH**_**3**_ **)**_**2**_ to nitrido species (Figure S48) and the subsequent processes with NH_3_ as an ancillary ligand (Figure S49). The oxidation of **Ru­(NH**_**3**_ **)**_**2**_ to the first amido intermediate has a barrier of 5.8 kcal mol^–1^, which is 1.1 kcal mol^–1^ lower than that of **RuNH**_**3**_ (6.9 kcal mol^–1^) (Figure S47), indicating that the first one-electron oxidation of **Ru­(NH**_**3**_ **)**_**2**_ is more facile and may account for the observation of the oxidation wave at 0.15 V in the CV. In the subsequent reaction leading to the formation of N_2_, the pathway of nucleophilic attack by NH_3_ is 4.0 kcal mol^–1^ lower than that of N···N coupling (18.5 vs 22.5 kcal mol^–1^), which is the same as in **RuNH**_**3**_. We also calculated the formation of a diazene intermediate (Figure S50), which has a highly energetic transition state and was ruled out.

## Conclusions

We have shown that ruthenium­(III) ammine complexes bearing Schiff-base-type ligands [Ru^III^(salchda)­(NH_3_)­(CH_3_CN)]^+^ and its derivatives are highly efficient catalysts for the oxidation of NH_3_ to N_2_, with TON and FE being the highest among molecular ruthenium catalysts. We provided evidence that the active intermediate is the ruthenium­(VI) nitrido species [Ru^VI^(salchda)­(N)]^+^ by direct comparison with an authentic sample of RuN, which we previously synthesized. We have also directly studied the kinetics and mechanism of the oxidation of NH_3_ by **RuN** and the results are consistent with parallel electrophilic attack of NH_3_ by **RuN** and bimolecular N···N coupling of **RuN** to produce N_2_.

## Supplementary Material



## Notes

General experimental considerations, details on experimental and computational methods, and additional results and discussions (PDF)
