# Supplementary material for: Electrocatalytic Oxidation of Ammonia by (Salen)ruthenium(III) Ammine Complexes: Direct Evidence for a Ruthenium(VI) Nitrido Active Intermediate
Source: J Am Chem Soc. 2025 Apr 17;147(17):14211–8. doi: 10.1021/jacs.4c16902 (PMC12758798; doi:10.1021/jacs.4c16902)
Supplement: Supplementary file 1 [file ja4c16902_si_001.pdf]

Electrocatalytic Oxidation of Ammonia by (Salen)ruthenium(III) Ammine Complexes. Direct Evidence for a Ruthenium(VI) Nitrido Active Intermediate.

Jianhui Xie,[a,*] Tingting Yang,[a] Longzhu Hong,[a] Hui Li,[b] Bing Li,[a] Zhenguo Guo,[a] Yingying Liu,[b,*] Tai-Chu Lau[c,*]

[a] Anhui Province Key Laboratory of Advanced Catalytic Materials and Reaction Engineering, School of Chemistry and Chemical Engineering, Hefei University of Technology, Hefei 230009, P. R. China.

[b] Institute of Intelligent Machines, Hefei Institutes of Physical Science, Chinese Academy of Sciences, Hefei 230031, P. R. China.

[c] Department of Chemistry, City University of Hong Kong, Kowloon Tong 999077, Hong Kong, P. R. China.

Email: Jianhui Xie (jianhuixie@hfut.edu.cn), Yingying Liu (yyliu@iim.ac.cn), Tai-Chu Lau (bhtclau@cityu.edu.hk)

Contents

Experimental Section......S4
Coordinates of INTs and TSs......S**Error! Bookmark not defined.**40
References......S202

Scheme S1 Reactivity of [Ru^{VI}(salchda)(N)(CH_3OH)]$^+$......S9
Figure S1 Structure of ^{X}**RuN**......S10
Figure S2 ESI/MS of **$RuNH_3$** and NH_3 in CH_3CN......S10
Figure S3 ESI/MS of ^{Br}**$RuNH_3$** and NH_3 in CH_3CN......S11
Figure S4 UV/Vis spectra of the titration of **$RuNH_3$** using DBU......S11
Figure S5 UV/Vis spectra of the titration of ^{Br}**$RuNH_3$** using Et_3N......S12
Figure S6 CV of **$RuNH_3$**......S12
Figure S7 CV of **$RuNH_3$** in the presence of 2,4,6-collidine......S13
Figure S8 CV of **RuN** in the presence of 2,4,6-collidine......S13
Figure S9 DPV of **$RuNH_3$** in the presence of NH_3 and CVs of **$(RuNH_3)_2$**......S14
Figure S10 UV/vis spectral changes of **$RuNH_3$** with NH_3......S15
Figure S11 UV/vis spectra of **$Ru(NH_3)_2$** and **$Ru(NH_3)$**......S15
Figure S12 UV/vis spectral changes of **$Ru(NH_3)_2$** in CH_3CN......S16
Figure S13 FOWA of **$RuNH_3$**......S17
Figure S14 CV of ^{Br}**$RuNH_3$** in the presence of NH_3......S18
Figure S15 CV of ^{Br}**$RuNH_3$**......S18
Figure S16 CV of ^{Br}**$RuNH_3$** in the presence of 2,4,6-collidine......S19
Figure S17 CV of ^{Br}**RuN** in the presence of 2,4,6-collidine......S19
Figure S18 CVs of [^{Br}**$RuNH_3$**] various [NH_3]......S20
Figure S19 FOWA of ^{Br}**$RuNH_3$**......S21
Figure S20 CPE using **$RuNH_3$** at 0.65 V......S22
Figure S21 GC of gas products from CPE using **$RuNH_3$** catalyst......S22
Figure S22 CPE using **$RuNH_3$** at 0.70 V......S23
Figure S23 CPE using **$RuNH_3$** at 0.80 V......S23
Figure S24 CVs of the glass carbon plate after the CPE at 0.8 V......S24
Figure S25 CPE using **RuN** catalyst at 0.80 V......S24
Figure S26 GC of the gas products from CPE using **RuN** catalyst......S25
Figure S27 CPE using ^{Br}**$RuNH_3$** at 0.65 V......S25
Figure S28 GC of the gas products from CPE using ^{Br}**$RuNH_3$**......S26
Figure S29 CPE using ^{Br}**$RuNH_3$** at 0.80 V......S26
Figure S30 CPE without catalysts......S27
Figure S31 CPE using **$RuNH_3$** at 0.65 V with $^{15}NH_3$......S27
Figure S32 N_2 distribution after CPE for 2300s using **$RuNH_3$** + $^{15}NH_3$......S28
Figure S33 GC of air showing N_2 distribution and O_2 of air......S28
Figure S34 ESI/MS of **$Ru^{14}NH_3$** + $^{14}NH_3$ + [(*p*-BrC_6H_4)$_3$N][$SbCl_6$]......S29
Figure S35 ESI/MS of **$Ru^{14}NH_3$** + $^{15}NH_3$ + [(*p*-BrC_6H_4)$_3$N][$SbCl_6$]......S29
Figure S36 UV/vis Spectrum of **$RuNH_3$** (0.06 mM) in CH_3CN......S30

Figure S37 UV/vis Spectrum of $[Ru(salchda)(CH_3CN)_2]^+$ (0.06 mM) in CH_3CN. S30
Figure S38 Plot of initial rate vs $[NH_3]$ for the reaction of **RuN** with NH_3 at 15 °C. S31
Figure S39 Plot of initial rate vs $[NH_3]$ for the reaction of **RuN** with NH_3 at 35 °C. S31
Figure S40 Plot of initial rate vs $[NH_3]$ for the reaction of **RuN** with NH_3 at 45 °C. S32
Figure S41 Plot of $\ln(k_a/T)$ vs 1/T for the reaction of **RuN** with NH_3 in CH_3CN. S32
Figure S42 Plot of $\ln(k_b/T)$ vs 1/T for the reaction of **RuN** with NH_3 in CH_3CN. S33
Figure S43 Plot of $\log(k_b/k_b^H)$ versus σ_p for the reaction of **^{X}RuN**...S33
Figure S44 ESI/MS of **Ru^{14}N** with $^{14}NH_3$ in CH_3CN. S34
Figure S45 ESI/MS of **Ru^{14}N** with $^{15}NH_3$ in CH_3CN. S34
Figure S46 N_2 isotope distribution for $^{15}NH_3$ + **Ru^{14}N**. S35
Figure S47 Free energy profile for the oxidation of **RuNH$_3$** to **Ru≡N**. S36
Figure S48 Free energy profile for the oxidation of **Ru(NH$_3$)$_2$** to **Ru≡N(NH$_3$)**. S36
Figure S49 Free energy profile for the N_2 formation starting from **RuN(NH$_3$)**. S37
Figure S50 Free energy profile for the formation of a diazene intermediate. S37
Table S1 Crystal data and structure refinement for **[^{Br}RuNH$_3$]PF$_6$**. S38
Table S2 Summary of the rate constants from the plot of initial rate vs [**^{X}RuN**] + NH_3.............S39

Experimental Section

Materials. All chemicals were used as purchased unless otherwise specified. [Ru^{VI}(N)(salchda)(CH_3OH)](PF_6) and the other ruthenium nitrido complexes with salchda ligands bearing substituents were prepared according to a literature method.[1] All solvents including acetonitrile (CH_3CN) were dried and degassed by solvent purification systems (MBRAUN). NH_3 solution was prepared by bubbling NH_3 gas into CH_3CN for 1 h and the concentration was titrated. $^{15}NH_3$ was prepared by reacting $^{15}NH_4Cl$ (Cambridge Isotope Laboratories, 99 atm % ^{15}N) with 1,8-diazabicyclo[5.4.0]undec-7-ene (DBU) in CH_3CN at room temperature under Ar. The $^{15}NH_3$ solution was then collected under vacuum by using a Schlenk tube in a liquid nitrogen bath.

Physical measurements. Electrospray ionization mass spectrometry (ESI-MS) was performed on a SCIEX TRIPLE QUAD 3500 mass spectrometer. The analyte solution was continuously infused at a constant flow rate of 10 μL min^{-1} using a syringe pump into the pneumatically assisted electrospray probe with nitrogen serving as the nebulizing gas. The declustering potential was typically set between 1−10 V. Elemental analysis was conducted using an Elementar vario EL cube elemental analyzer. Electrochemistry experiments were conducted on a CH Instruments Electrochemical Workstation CHI660E. UV-Vis spectral measurements and kinetics experiments were conducted using an Agilent 8453 diode-array spectrophotometer. The temperature of the solutions was maintained using an IKEA HRC 2 temperature controller connected to a circulating water bath. Gas chromatographic (GC) analyses were performed on a Shimadzu GC2010 Pro TCD gas chromatograph equipped with a Chrompack 5 Å molecular sieve column (30 m × 0.32 mm × 1.5 mm). GC-MS measurements were carried out on a Thermo Scientific Trace 1300 GC interfaced with an ISQ 7000 MS equipped with a TG-5SILMS (30 m × 0.25 mm) capillary column.

X-ray crystallography. Crystals were coated with paratone-N and mounted on a nylon cryoloop for X-ray diffraction analysis. Data were collected at 170 K using ω-scan mode on a Bruker APEX-II with a CCD detector, employing mirror monochromatized Mo-Kα radiation (λ = 0.71073 Å). The data were recorded with absorption correction using the multi-scan method by CrysAlis.[2] The structure was solved using intrinsic phasing methods with SHELXT[3] and refined with SHELXL[4] within the Olex2 graphical user interface.[5,6] The final structural refinement included anisotropic temperature factors for all non-hydrogen atoms. Hydrogen atoms were placed at calculated positions using the riding model and included in the final R-indices calculation. The automatic "solvent masking procedure" in Olex2 was used to mask solvent molecules present in the structure.[5-7]

Electrochemical studies. Unless mentioned otherwise, the cyclic voltammetry (CV) and controlled-potential electrolysis (CPE) were measured in CH_3CN at a scan rate of 0.1 V s^{-1} containing 0.1 M $[^nBu_4N]PF_6$ as electrolyte. CV was carried out with a typical three-electrode system including a glassy carbon electrode (3 mm diameter) as the working electrode, Pt wire as the counter electrode, and an $Ag/AgNO_3$ reference electrode. The $Ag/AgNO_3$ reference electrode was employed using an CH_3CN solution containing 10 mM $AgNO_3$ and 0.1 M $[^nBu_4N]PF_6$.

Controlled-potential electrolysis (CPE) was performed using the similar three-electrode system, except that a glassy carbon plate (area: 1.0 cm^2) and a Pt plate (area: 2.0 cm^2) were used as working electrode and counter electrode, respectively. The total volume of the reaction cell was 22.2 mL, with solution of 7 mL and 15.2 mL for headspace. All redox potentials in the present work are reported versus the $Fc^{+/0}$ couple, measured before each experiment to be +0.095 V versus the $Ag/AgNO_3$ reference electrode. CPEs were performed in a sealed electrolytic cell under an Ar atmosphere. Then, 100 μL of the headspace was injected into a GC-TCD chromatograph at intervals for analysis.

Foot-of-the-wave analysis (FOWA) was investigated using the following equation.[8]

$$\frac{i_{cat}}{i_p} = \frac{\mathrm{n}\cdot 2.24\cdot\sqrt{\frac{RT}{Fv}}\cdot k_{\mathrm{obs}}}{1 + \exp\left[\frac{F}{RT}\cdot(E^{\mathrm{o}}_{cat} - E)\right]}$$

F is Faraday's constant, *R* is the gas constant, and *T* is the temperature. In this case, n = 6 (ammonia oxidation to dinitrogen). The intensity of the one electron wave (i_{p}) was estimated from the one-electron oxidation catalytic wave. E^{o}_{cat} was determined as the potential of the half-wave for the catalytic process. The catalytic current (i_{cat}) used for the FOWA has been corrected based on the background current. To ensure a fair comparison, the FOWA was conducted in similar regions of the potential range for each condition. The region was selected as the foot-of-the-wave where the slope of $i_{\mathrm{cat}}/i_{\mathrm{p}}$ vs (1+exp[F/RT × ($E^{\mathrm{o}}_{cat}-E$)]) behaves linearly. From the slope of the plot, the apparent pseudo-first order rate constant k_{obs} can be obtained and thus the value of the apparent second-order rate constant k_{obs}'.

Kinetics. Kinetic studies were carried out using an Agilent 8453 diode-array spectrophotometer. The temperatures of the solutions were maintained with a PolyScience digital temperature controller connected to a circulating water bath. The concentrations of NH_3 were at least in 10-fold excess to that of **RuN**. The reaction progress was monitored by observing absorbance changes around 700 nm. Initial rate was done by first fitting the absorbance-time trace for one half-life using the equation $A = a_0 + a_1t + a_2t^2 + ... + a_9t^9$. The slope dA/dt was then obtained by differentiating with respect to *t*; the initial rates measured under pseudo-first-order conditions, were determined by setting t=0.[9]

Computational details. The DFT calculations were performed using the ORCA software package (version 5.0.4),[10] incorporating the RIJCOSX[11] method for hybrid DFT calculations. Geometry optimizations for Ru complexes were carried out at the B3LYP-D3(BJ)/def2-SVP/def2-TZVP(Ru) level in CH_3CN, utilizing the Conductor-like Polarizable Continuum Model (CPCM).[12] All geometries were optimized and verified for the correct number of imaginary frequencies via vibrational frequency

calculations. Ground states were confirmed by the absence of imaginary frequencies, while transition states had only one imaginary frequency. All relevant spin states were evaluated for the complexes in their reactant, transition, and product states. The lowest free energy spin multiplicities were used to calculate thermodynamics (ΔG) and free energy barriers ($\Delta G^{\ddagger}$). Single-point calculations were conducted with the same functional and a larger basis set (def2-TZVP) with gCP[13] on the optimized geometries. The SMD implicit solvation model with CH_3CN was applied to account for solvation effects. Oxidation free energies were calculated relative to $FeCp_2 \rightarrow [FeCp_2]^+ + e^-$ in SMD-MeCN solvent.[8] $\Delta G(H^+)$ was determined from the proton transfer free energy in the reaction $NH_3 + H^+ \rightarrow NH_4^+$ in CH_3CN solvent. Transition state (TS) structures were determined using TS-optimization in ORCA. Initial geometries for the TS search were derived from previous studies on similar Ru catalysts.[14] The transition states were validated through normal mode vibrational vector analysis and intrinsic reaction coordinate (IRC) calculations. Activation energies ($\Delta G^{\ddagger}$) were calculated as the Gibbs free energy difference between the transition state and the optimized encounter complex.

Synthesis of [Ru^{III}(^{Br}salchda)(CH_3CN)(NH_3)]PF_6 ([^{Br}RuNH$_3$]PF_6). This complex was synthesized by a slightly modified method of the literature method.[15] *p*-Toluenethiol (31 mg, 0.25 mmol) was added to a solution of **^{Br}RuN** (50 mg, 0.065 mmol) in CH_3CN (10 mL). The mixture was stirred for an hour in air. Then, the solution was allowed to evaporate slowly in air. Blue single crystals suitable for X-ray analysis were obtained. Yield: 60 %. Anal. calcd. (found) for $C_{22}H_{24}N_4O_2Br_2PF_6Ru$: C, 33.78 (33.65); H, 3.09 (3.21); N, 7.16 (7.14). ESI/MS in acetonitrile: *m/z* = 638.

Synthesis of [Ru^{III}(salchda)(NH_3)$_2$]PF_6 ([Ru(NH_3)$_2$]PF_6). A solution of NH_3 in dioxane (1.5 mL, 0.4 M) was added to a solution of **RuN** (20 mg, 0.0326 mmol) in CH_2Cl_2 (10 mL). The mixture was stirred for 2 hours in air. Then, the solution was concentrated to 1 mL and was passed through a column of silica gel and eluted with CH_2Cl_2/acetone (v/v =7/3). After remove the solvent, blue solid was obtained. Yield:

73 %. Anal. calcd. (found) for $C_{20}H_{26}N_4O_2PF_6Ru$: C, 40.00 (39.89); H, 4.36 (4.39); N, 9.33 (9.28). ESI/MS in CH_2Cl_2: *m/z* = 456.

Determination of N_2 for the catalytic AO by $RuNH_3$ using $[(p\text{-}BrC_6H_4)_3N][SbCl_6]$ in CH_3CN. In a typical procedure, a deaerated solution of **$RuNH_3$** (1.2 mg, 1.92 μmol) in CH_3CN (2 mL) was transferred to a round-bottom flask (total volume ≈ 16.7 mL), which was then sealed with a septum under an argon atmosphere at room temperature. NH_3 in CH_3CN (2 mL, 0.6 M) was added using a gastight syringe, and the mixture was stirred for 1 minute. Next, a deaerated solution of $[(p\text{-}BrC_6H_4)_3N][SbCl_6]$ (2 mL, 96 mM) was introduced in the same manner. The reaction mixture was allowed to proceed for 10 minutes. A 100 μL sample of gas from the headspace was then withdrawn and analyzed by GC-MS.

Determination of N_2 for the reaction of RuN with $^{15}NH_3$ in CH_3CN. In a typical reaction, a deaerated solution of **RuN** (5.5 mg, 9.0 μmol) in CH_3CN (57 mL) was placed in a round bottom flask (total volume = ca.117 mL) sealed with a septum under argon at room temperature. $^{15}NH_3$ in CH_3CN (3 mL, 0.04 M) was added using a gastight syringe and the mixture was stirred for 60 min. 100 μL gas in the headspace was withdrawn and analyzed by GC-MS.

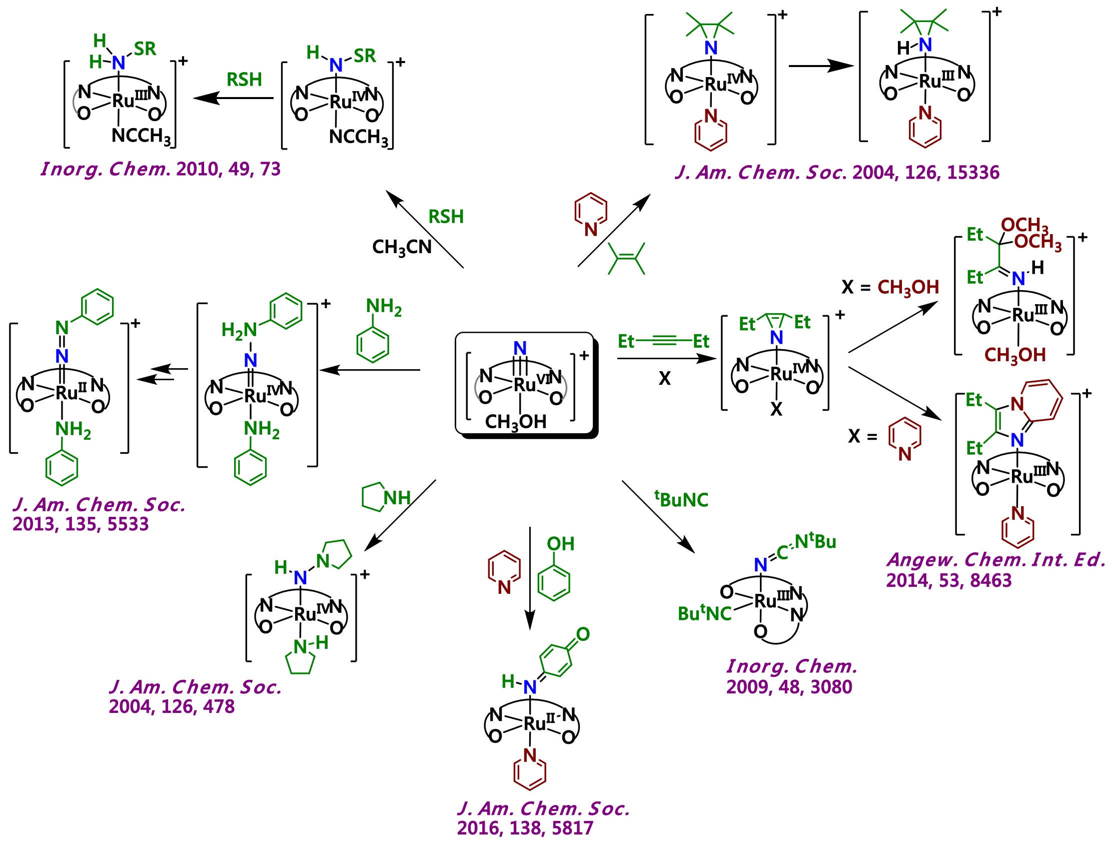

Scheme S1 Reactivity of $[Ru^{VI}(salchda)(N)(CH_3OH)]^+$.

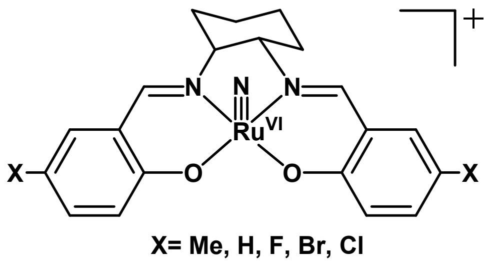

Figure S1 Structure of (salchda)Ru^{VI} nitrido complexes (^{X}**RuN**).

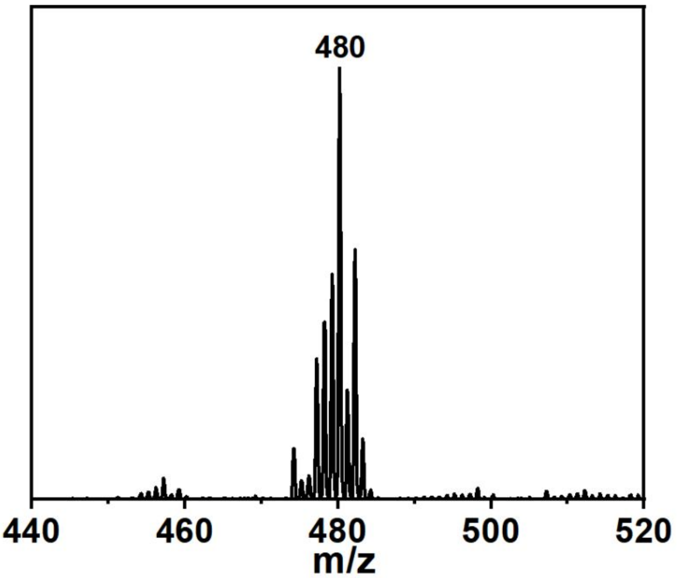

Figure S2 ESI/MS spectrum of the solution of **RuNH$_3$** (0.1 mM) and NH_3 (0.2 M) in CH_3CN.

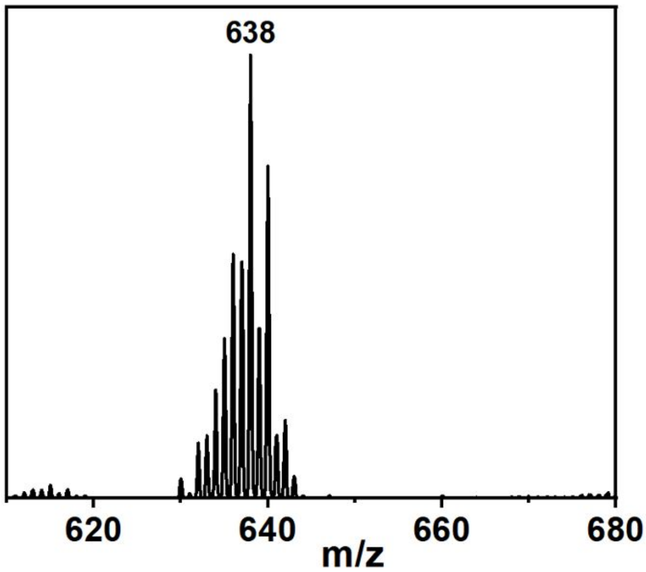

Figure S3 ESI/MS spectrum of the solution of ^{Br}**RuNH$_3$** (0.1 mM) and NH_3 (0.2 M) in CH_3CN.

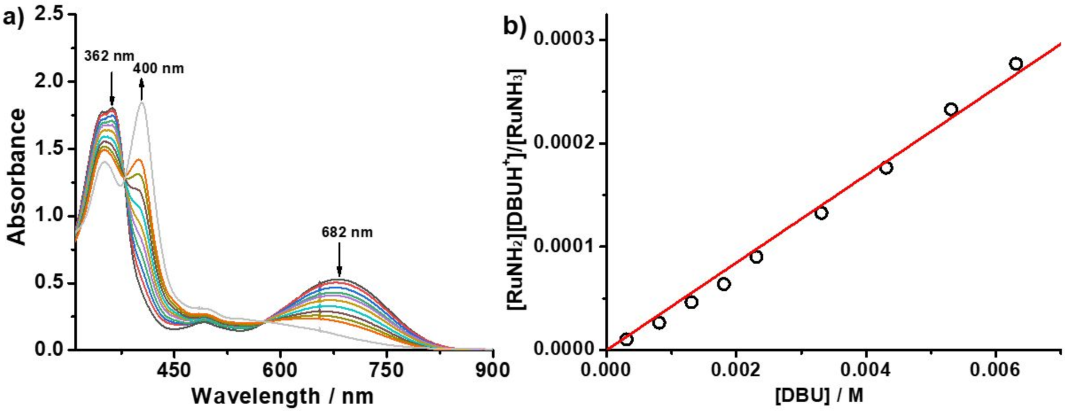

Figure S4 a) UV/Vis spectra of the titration of **RuNH$_3$** using DBU in CH_3CN. b) Plot of [**RuNH$_2$**][$DBUH^+$]/[**RuNH$_3$**] vs [DBU] for the equilibrium titration: **RuNH$_3$** + DBU ⇌ **RuNH$_2$** + $DBUH^+$ in CH_3CN. The slope of the linear plot (R^2 = 0.99) is the equilibrium constant K_{eq} = (4.23 ± 0.10) × 10^{-2}. pK_a[**RuNH$_3$**] = pK_a[DBU] – logK_{eq} = 25.

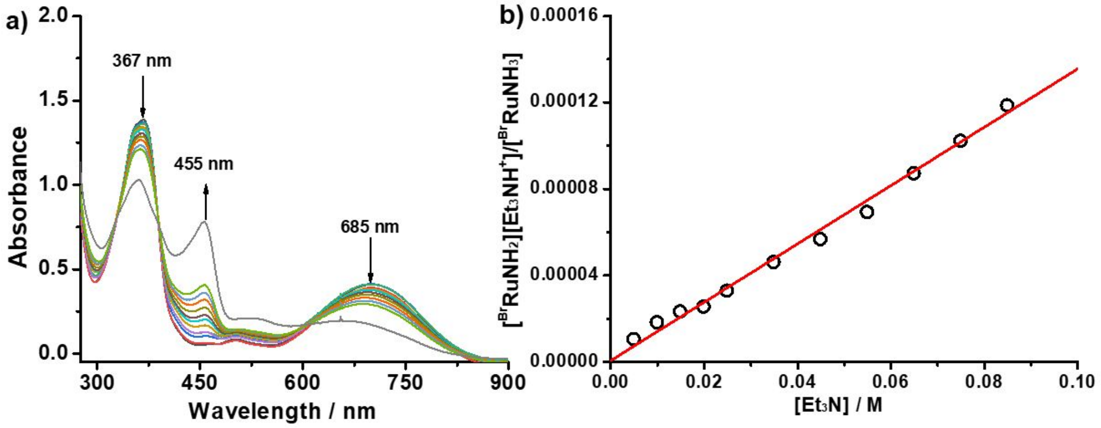

Figure S5 a) UV/Vis spectra of the titration of ^{Br}**RuNH$_3$** using Et_3N in CH_3CN. b) Plot of [^{Br}**RuNH$_2$**][Et_3NH^+]/[^{Br}**RuNH$_3$**] vs [Et_3N] for the equilibrium titration: ^{Br}**RuNH$_3$** + Et_3N ⇌ ^{Br}**RuNH$_2$** + Et_3NH^+ in CH_3CN. The slope of the linear plot ($R^2 = 0.99$) is the equilibrium constant $K_{eq} = (1.35 \pm 0.03) \times 10^{-3}$. p$K_a$[^{Br}**RuNH$_3$**] = p$K_a$[$Et_3N$] – log$K_{eq}$ = 21.

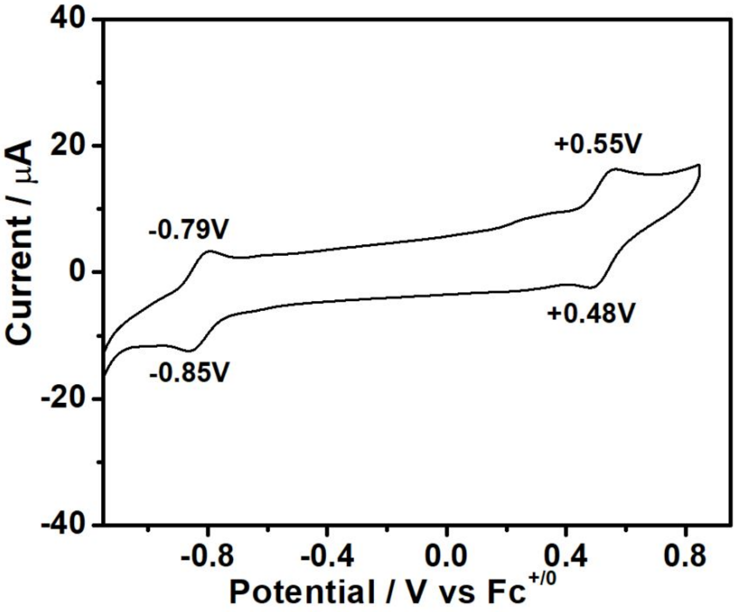

Figure S6 CV of **RuNH$_3$** at 0.1 V/s with [^{n}Bu$_4$N]PF$_6$ (0.1 M) as the supporting electrolyte in CH_3CN.

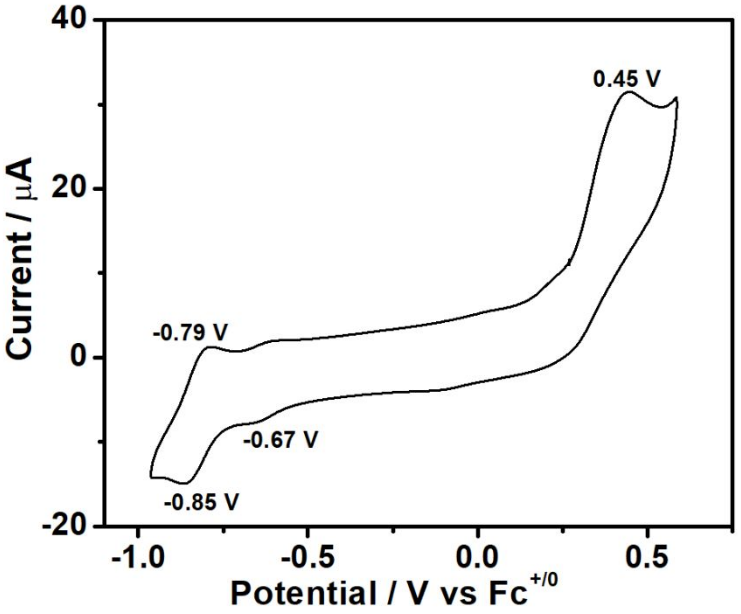

Figure S7 CV of **$RuNH_3$** at 0.1 V/s with [^{n}Bu$_4$N]PF$_6$ (0.1 M) as the supporting electrolyte in the presence of 0.01 M 2,4,6-collidine in CH_3CN.

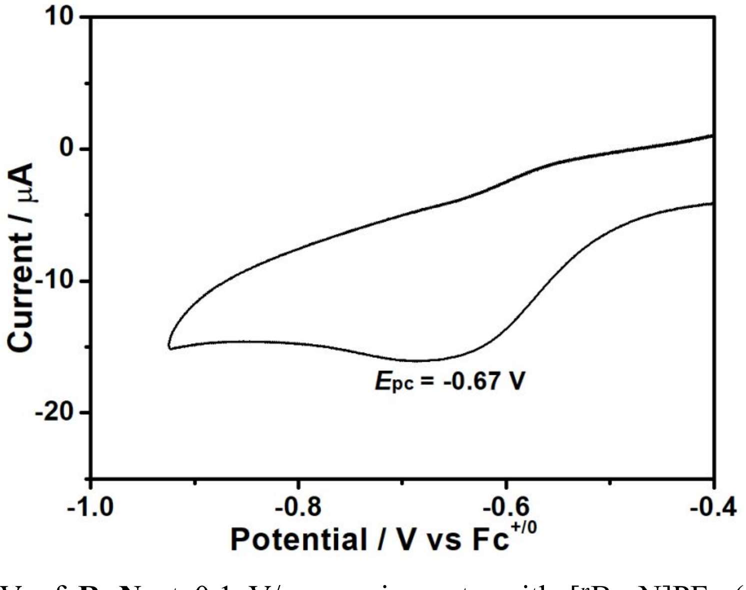

Figure S8 CV of **RuN** at 0.1 V/s scanning rate with [^{n}Bu$_4$N]PF$_6$ (0.1 M) as the supporting electrolyte in the presence of 0.01 M 2,4,6-collidine in CH_3CN.

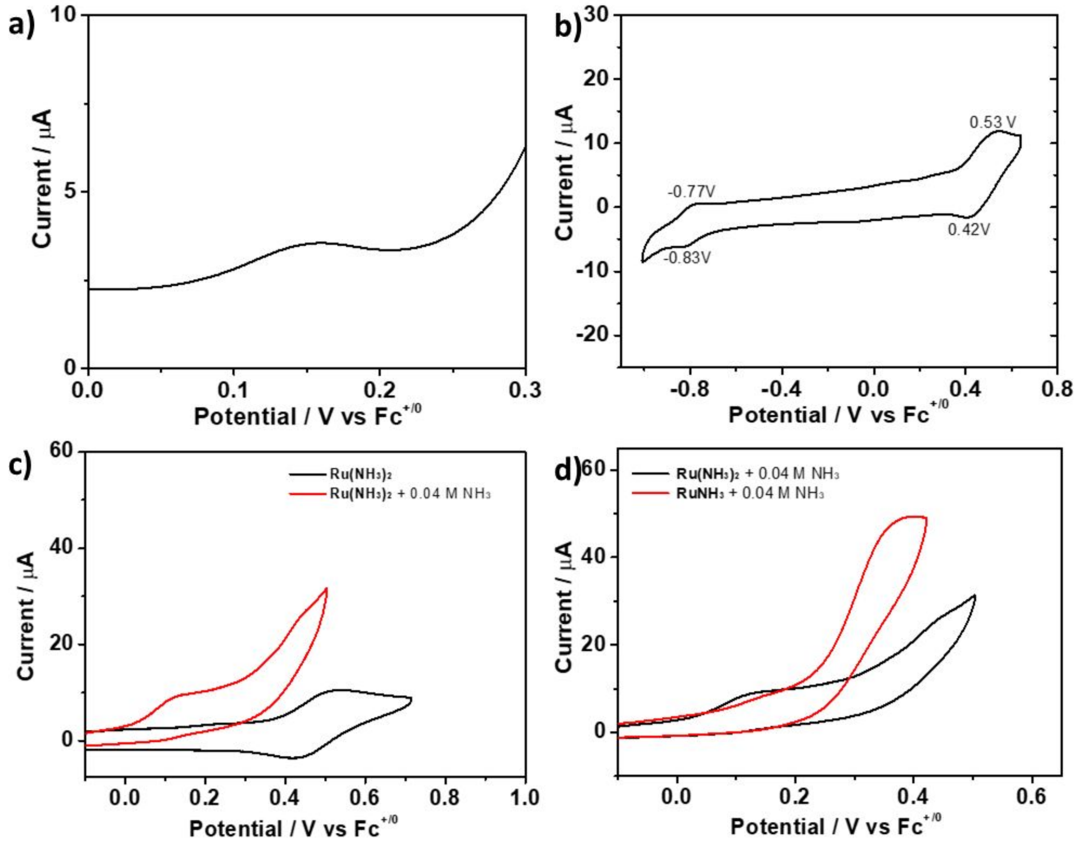

Figure S9 a) Differential pulse voltammetry (DPV) of **$RuNH_3$** (0.5 mM) in the presence of NH_3 (0.04 M) in CH_3CN. b) CV of **$(RuNH_3)_2$** (0.5 mM) in CH_3CN. There is a quasi-reversible wave at 0.48 V assigned to $[Ru(salchda)(NH_3)_2]^{2+/+}$ couple. The wave at –0.80 V is assigned to the $[Ru(salchda)(NH_3)_2]^{+/0}$ couple. c) CVs of **$Ru(NH_3)_2$** (0.5 mM) (black) and **$Ru(NH_3)_2$** (0.5 mM) (red) in the presence of NH_3 (0.04 M) in CH_3CN. d) CVs of **$Ru(NH_3)_2$** (0.5 mM) (black) and **$RuNH_3$** (0.5 mM) (red) in the presence of NH_3 (0.04 M) in CH_3CN. Scan rate = 0.1 V/s.

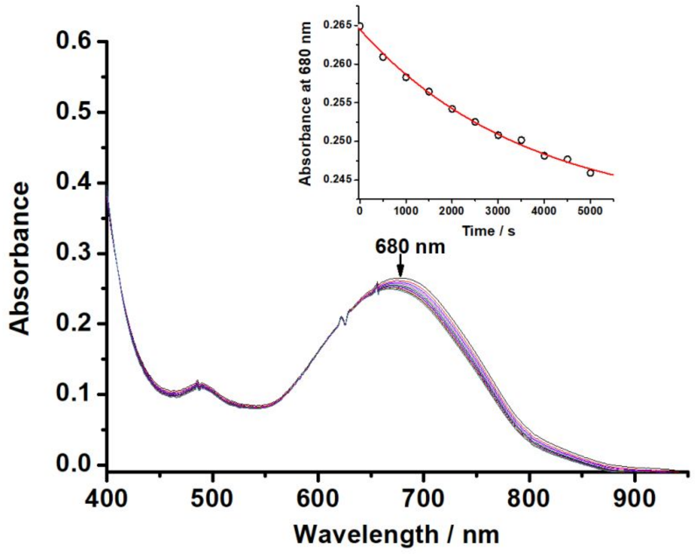

Figure S10 UV/vis spectral changes at 500s intervals of the reaction between **RuNH$_3$** (6.0×10^{-5} M) and NH_3 (0.1 M) in CH_3CN at 25.0 ℃. Inset shows the absorbance-time trace at 680 nm fitted by first-order equation. $k_{obs} = (2.8 \pm 0.3) \times 10^{-4}$ s^{-1}.

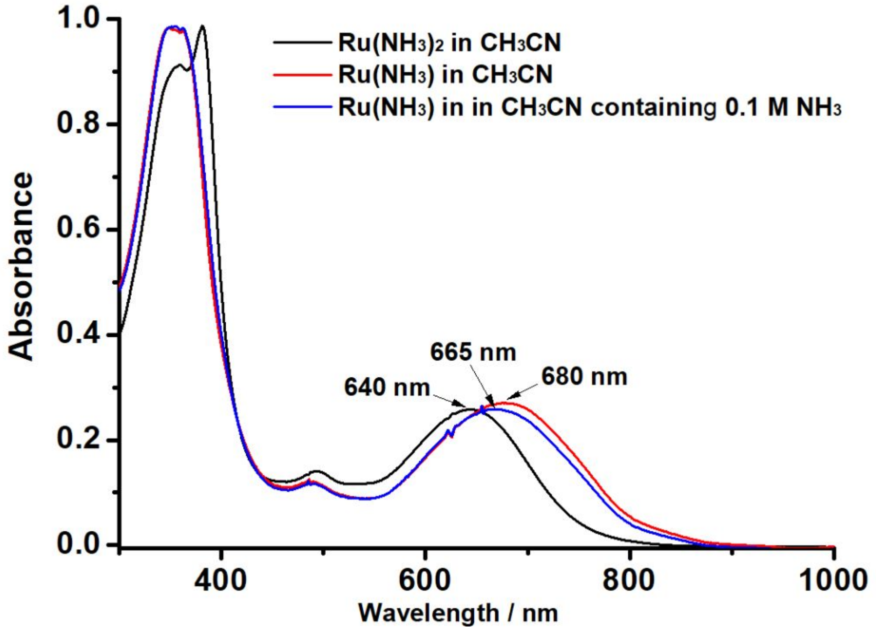

Figure S11 UV/Vis Spectra of **Ru(NH$_3$)$_2$** (6.0×10^{-5} M) (black line) and **Ru(NH$_3$)** (6.0×10^{-5} M) (red line) in CH_3CN along with the final spectrum from Figure S10 (blue line). Based on the final spectrum, where the total [**Ru**] is 6.0×10^{-5} M and the absorbance is attributed to a mixture of **Ru(NH$_3$)$_2$** and **Ru(NH$_3$)**, the concentrations of **Ru(NH$_3$)$_2$** and **Ru(NH$_3$)** were calculated to be 8.7×10^{-6} M and 5.13×10^{-5} M, respectively.

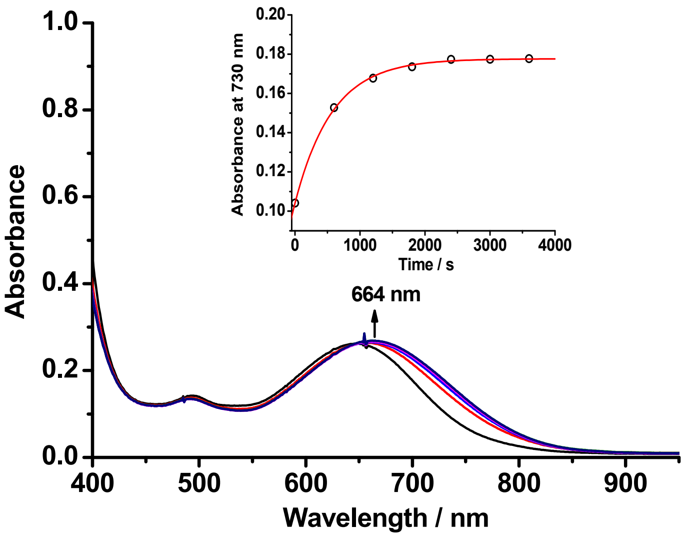

Figure S12 UV/Vis spectral changes at 500s intervals of the reaction of **Ru(NH$_3$)$_2$** (6.0 × 10^{-5} M) in CH_3CN containing 0.1 M NH_3 at 25.0 °C. Inset shows the absorbance-time trace at 730 nm fitted by first-order equation. $k_{obs} = (1.7 \pm 0.1) \times 10^{-3}\ s^{-1}$.

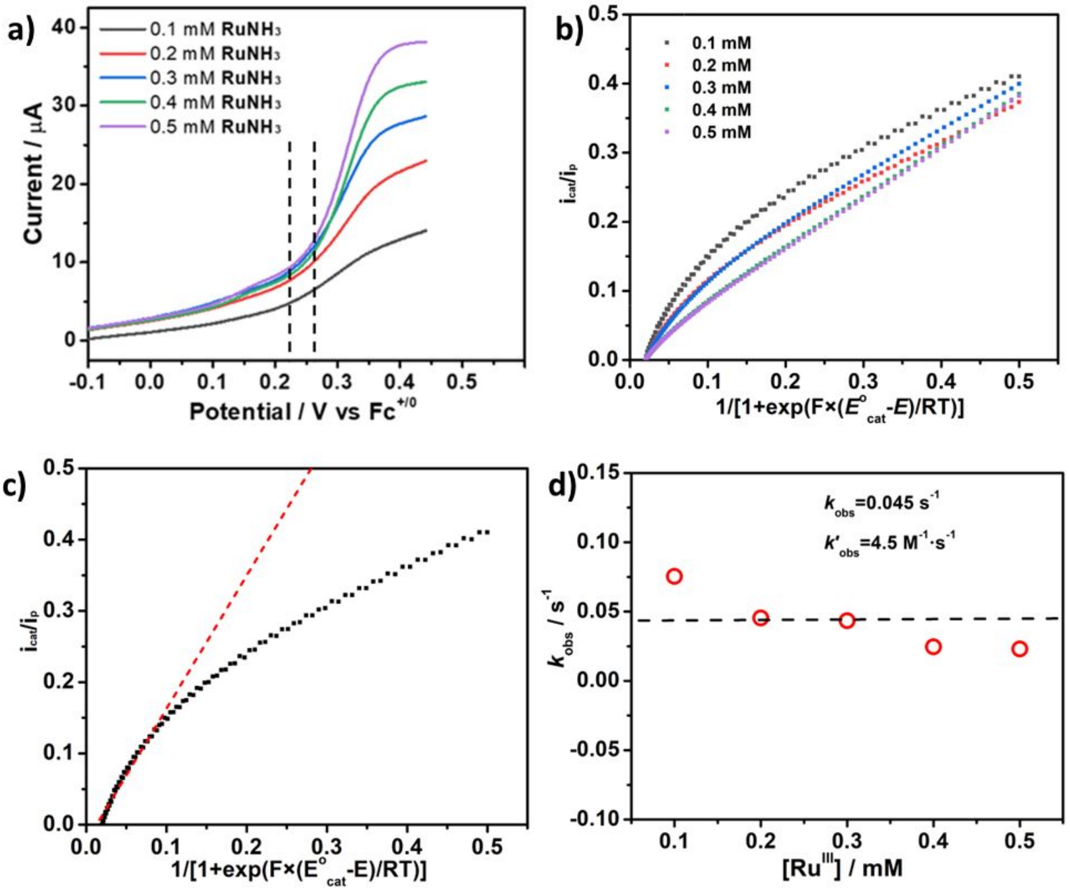

Figure S13 a) Linear sweep voltammograms of NH_3 (10 mM) at various [**$RuNH_3$**] in CH_3CN; the region between the dash lines was used for FOWA analysis. b) Corresponding FOWA plots from linear sweep voltammograms. c) Representative linear sweep voltammogram for FOWA with 0.1 mM **$RuNH_3$**. d) k_{obs} derived from FOWA as a function of [**$RuNH_3$**]. *Conditions*: [^{n}Bu$_4$N]PF$_6$ (0.1 M) as the supporting electrolyte; scan rate = 0.1V/s; potentials vs $Fc^{+/0}$; under Ar.

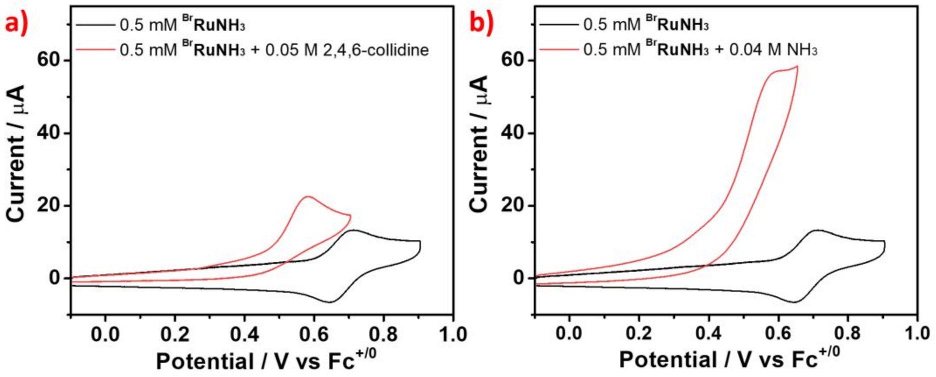

Figure S14 a) CV of ^{Br}**RuNH$_3$** (0.5 mM) (black) and that in the presence of 2,4,6-collidine (0.01 M) (red). b) CV of ^{Br}**RuNH$_3$** (0.5 mM) (black) and that in the presence of NH_3 (0.04 M) (red).

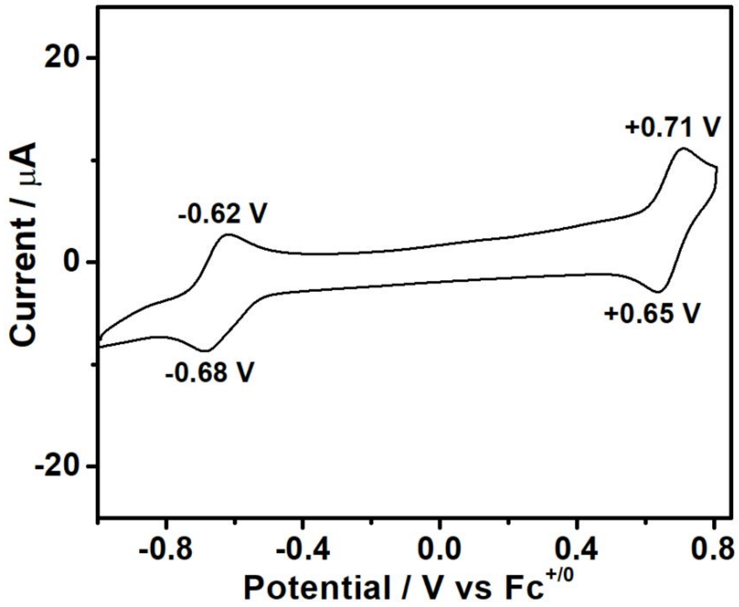

Figure S15 CV of ^{Br}**RuNH$_3$** at 0.1 V/s with 0.1 M [^{n}Bu$_4$N]PF$_6$ as the supporting electrolyte in CH_3CN.

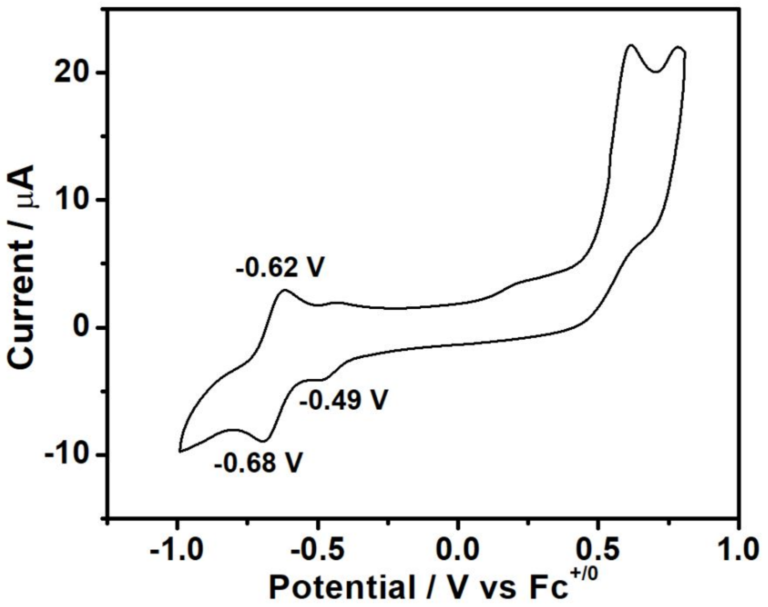

Figure S16 CV of **^{Br}RuNH$_3$** at 0.1 V/s with 0.1 M [^{n}Bu$_4$N]PF$_6$ as the supporting electrolyte in the presence of 0.01 M 2,4,6-collidine in CH_3CN.

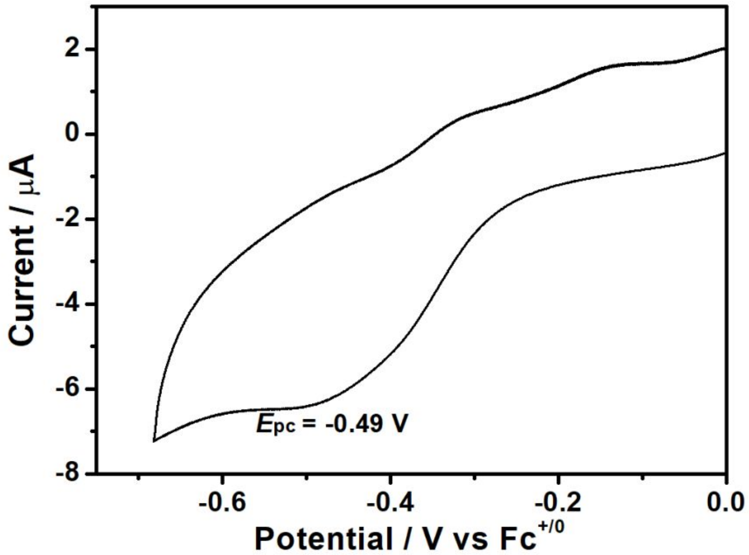

Figure S17 CV of **^{Br}RuN** at 0.1 V/s with [^{n}Bu$_4$N]PF$_6$ (0.1 M) as the supporting electrolyte in the presence of 0.01 M 2,4,6-collidine in CH_3CN.

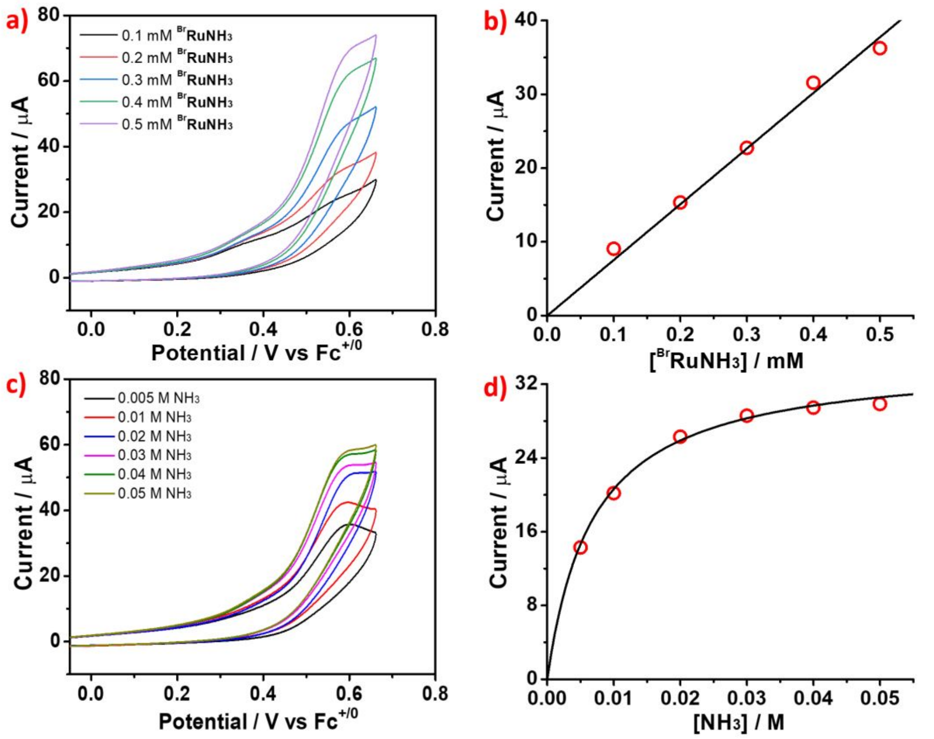

Figure S18 a) CVs in CH_3CN solutions of 0.05 M NH_3 and 0.1 M [$^nBu_4N$]PF_6 at various [^{Br}**RuNH$_3$**]. b) Plot of the current at $E_{1/2}$ vs [^{Br}**RuNH$_3$**]. c) CVs in CH_3CN solutions of 0.4 mM ^{Br}**RuNH$_3$** and 0.1 M [$^nBu_4N$]PF_6 at various [NH_3]. d) Plot of the current density at $E_{1/2}$ vs [NH_3].

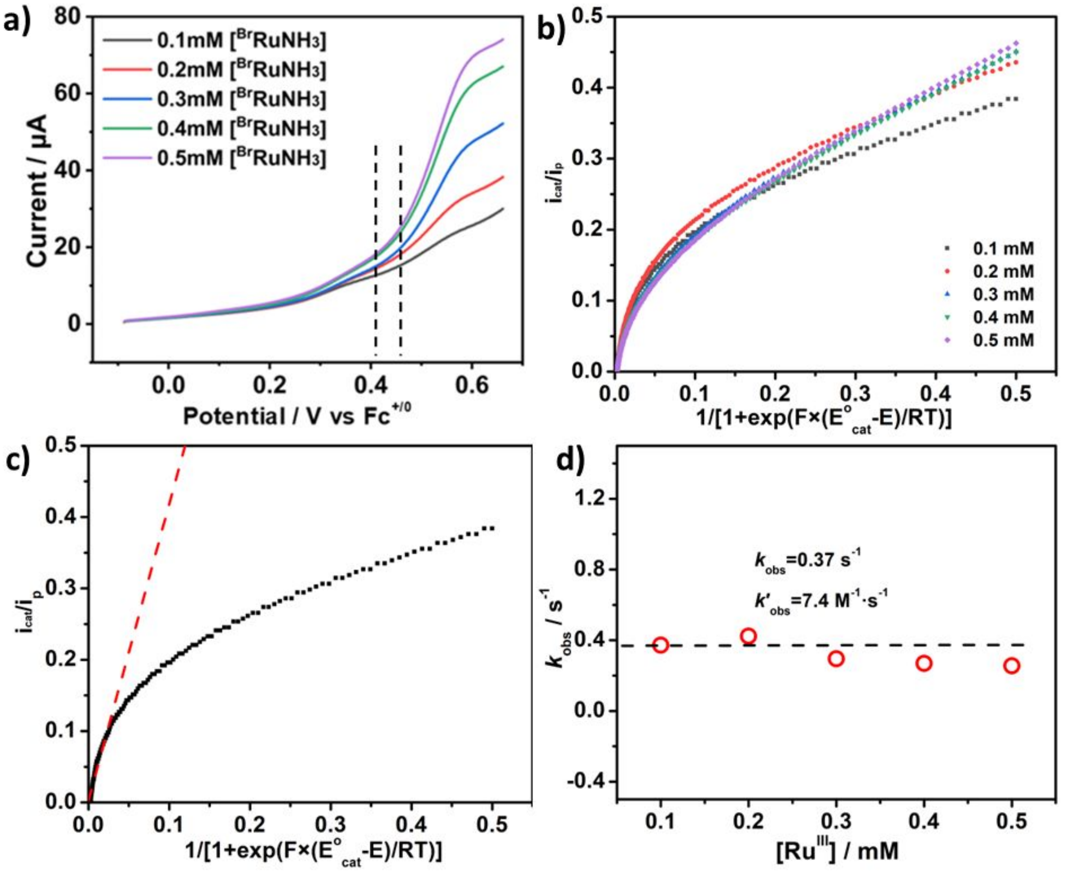

Figure S19 (a) Linear sweep voltammograms of NH_3 (50 mM) at various [^{Br}**RuNH$_3$**] in CH_3CN; the region between the dash lines was used for FOWA analysis. (b) Corresponding FOWA plots from linear sweep voltammograms. (c) Representative FOWA to calculate k_{obs} from the linear sweep voltammogram of ^{Br}**RuNH$_3$** (0.1 mM). d) k_{obs} derived from FOWA as a function of [^{Br}**RuNH$_3$**].

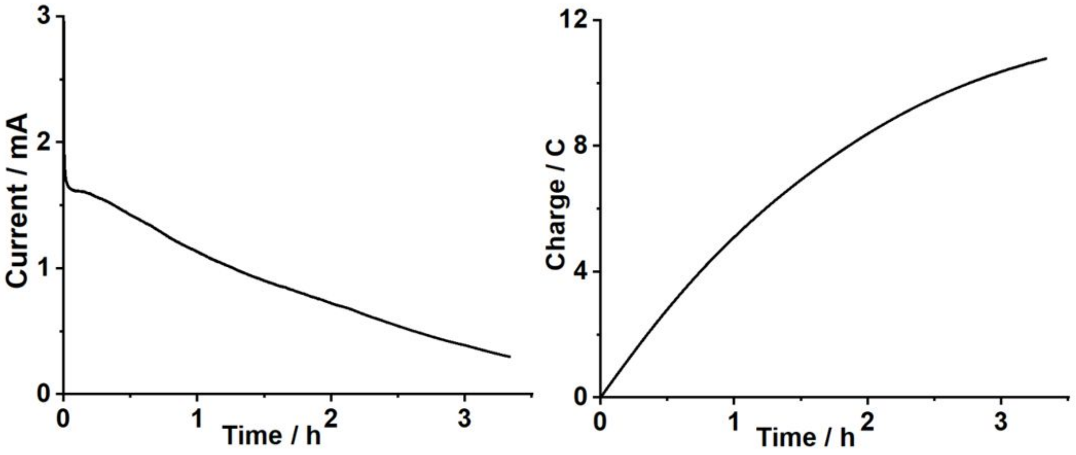

Figure S20 CPE using **RuNH$_3$** catalyst (7 mL CH_3CN; [Ru] = 0.1 mM; [NH_4OTf] = 0.1 M; [NH_3] = 0.2 M; potential = 0.65 V vs $Fc^{+/0}$).

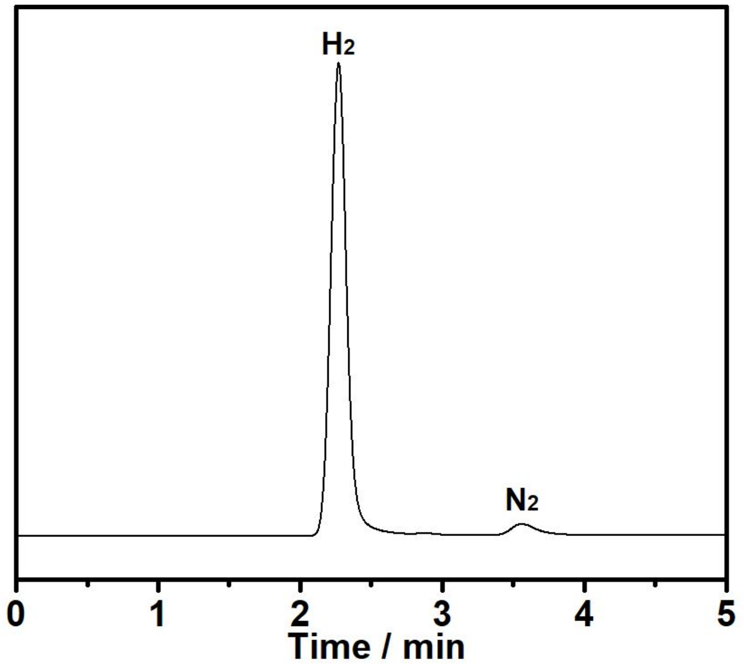

Figure S21 GC of gas products from CPE using **RuNH$_3$** catalyst (7 mL CH_3CN; [Ru] = 0.1 mM; [NH_4OTf] = 0.1 M; [NH_3] = 0.2 M; potential = 0.65 V vs $Fc^{+/0}$).

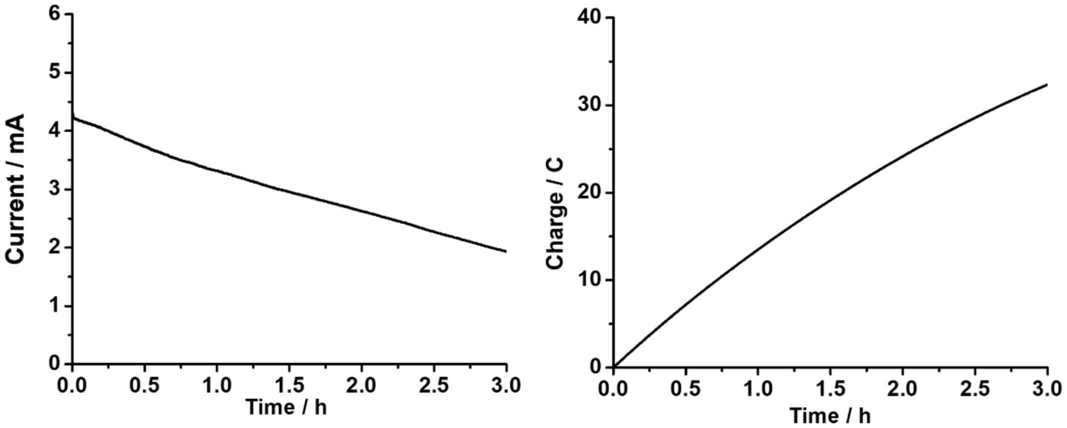

Figure S22 CPE using **$RuNH_3$** catalyst (7 mL CH_3CN; [Ru] = 0.1 mM; [NH_4OTf] = 0.1 M; [NH_3] = 0.2 M; potential = 0.70 V vs $Fc^{+/0}$).

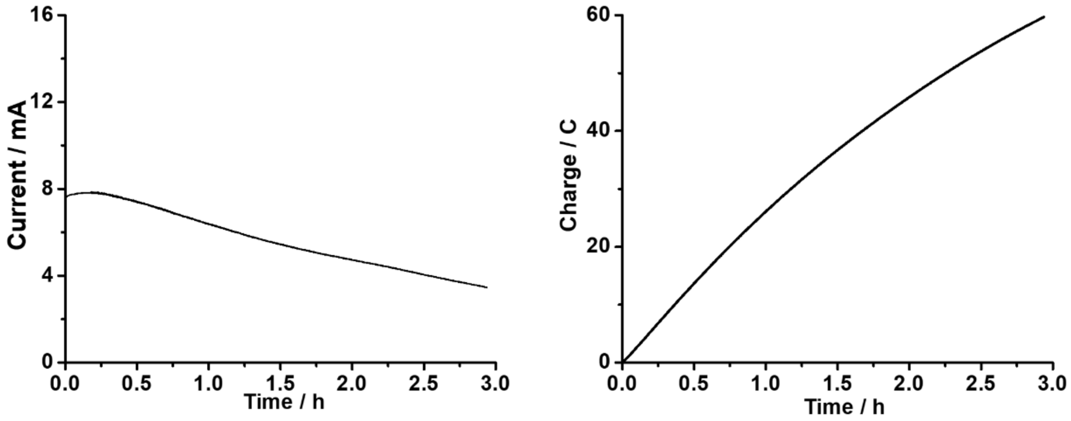

Figure S23 CPE using **$RuNH_3$** catalyst (7 mL CH_3CN; [Ru] = 0.1 mM; [NH_4OTf] = 0.1 M; [NH_3] = 0.2 M; potential = 0.80 V vs $Fc^{+/0}$).

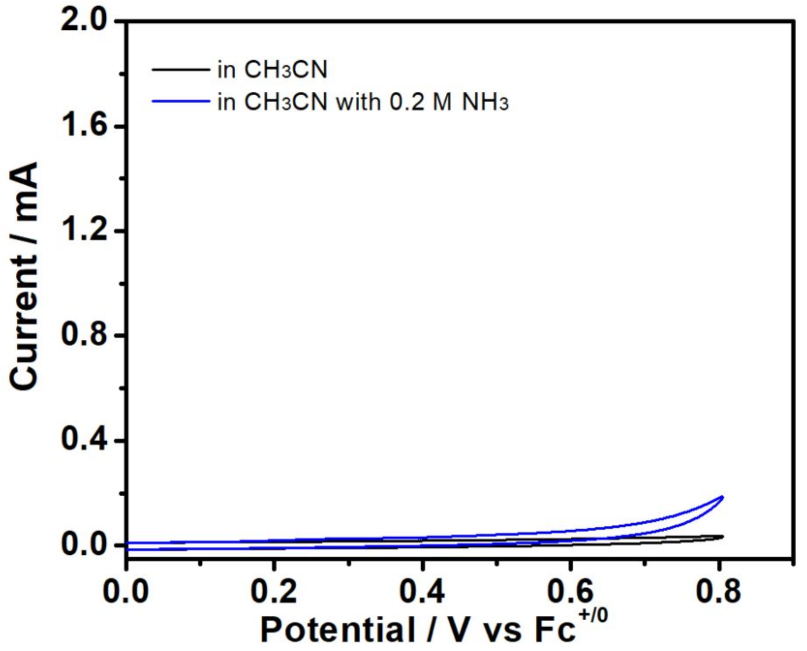

Figure S24 CVs of the glass carbon plate after the CPE at 0.8 V vs $Fc^{+/0}$ in CH_3CN with and without NH_3 using [^{n}Bu_4N]PF_6 (0.1 M) as the supporting electrolyte.

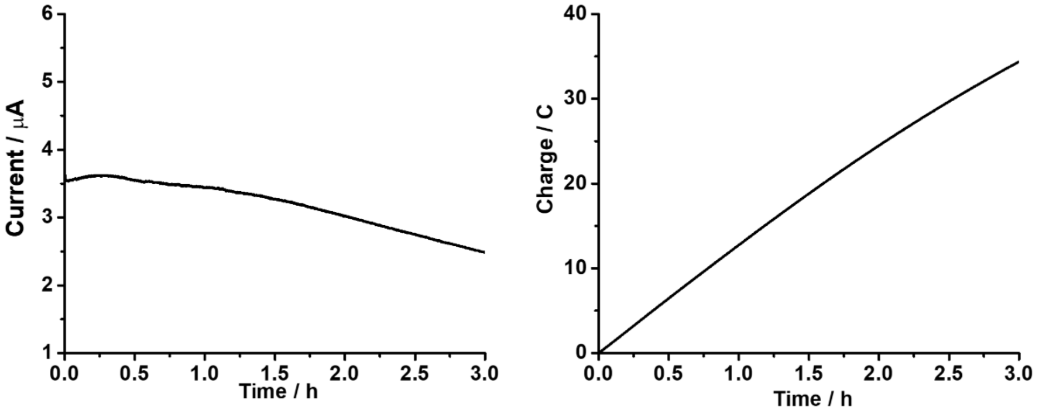

Figure S25 CPE using **RuN** catalyst (7 mL MeCN; [Ru] = 0.1 mM; [NH_4OTf] = 0.1 M; [NH_3] = 0.2 M; potential = 0.80 V vs $Fc^{+/0}$).

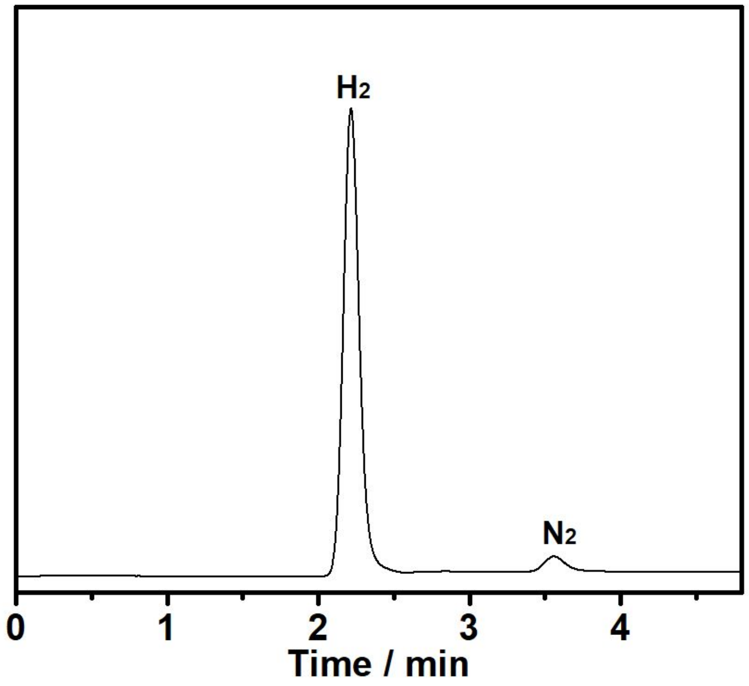

Figure S26 GC of the gas products from CPE using **RuN** catalyst (7 mL CH_3CN; [Ru] = 0.1 mM; [NH_4OTf] = 0.1 M; [NH_3] = 0.2 M; potential = 0.80 V vs $Fc^{+/0}$).

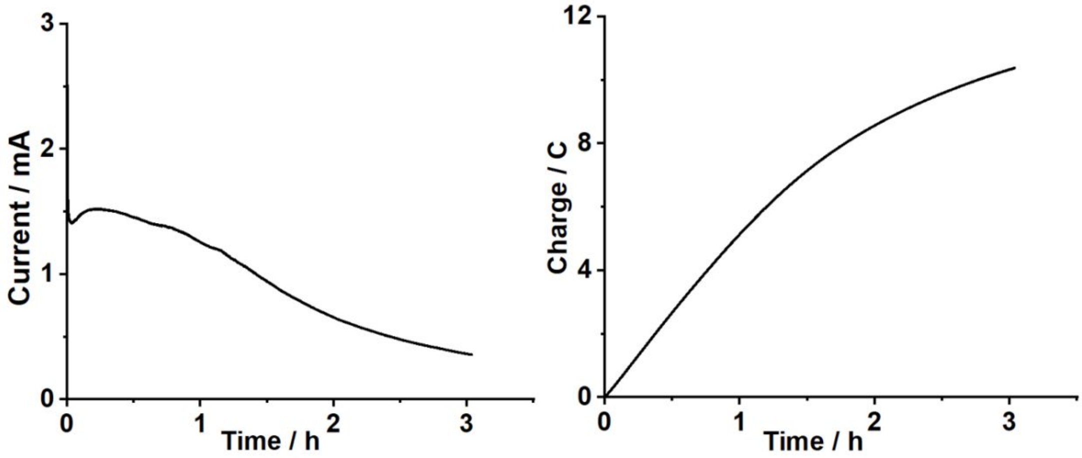

Figure S27 CPE using ^{Br}**RuNH$_3$** catalyst (7 mL CH_3CN; [Ru] = 0.1 mM; [NH_4OTf] = 0.1 M; [NH_3] = 0.2 M; potential = 0.65 V vs $Fc^{+/0}$).

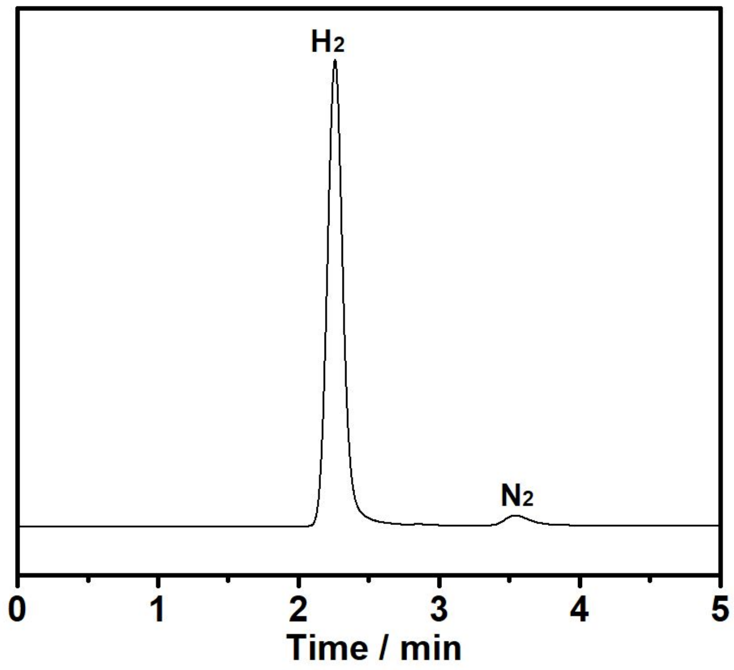

Figure S28 GC of the gas products from CPE using ^{Br}**RuNH$_3$** catalyst (7 mL CH_3CN; [Ru] = 0.1 mM; [NH_4OTf] = 0.1 M; [NH_3] = 0.2 M; potential = 0.65 V vs $Fc^{+/0}$).

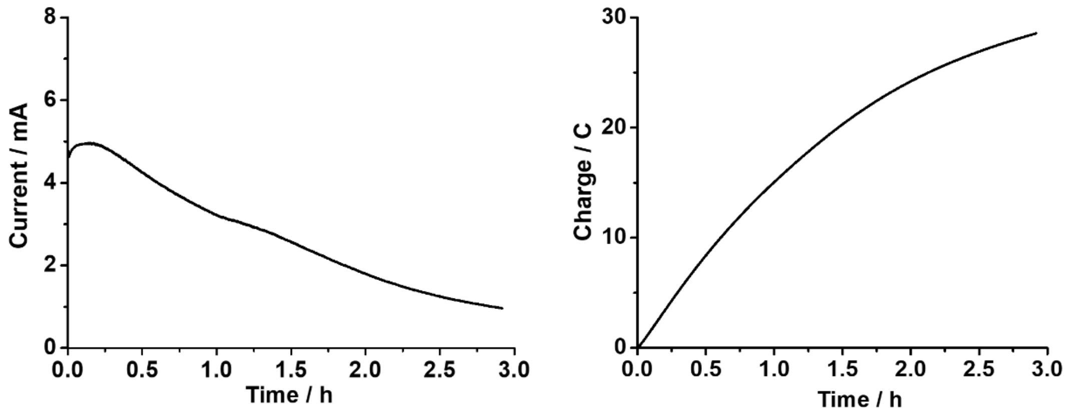

Figure S29 CPE using ^{Br}**RuNH$_3$** catalyst (7 mL CH_3CN; [Ru] = 0.1 mM; [NH_4OTf] = 0.1 M; [NH_3] = 0.2 M; potential = 0.80 V vs $Fc^{+/0}$).

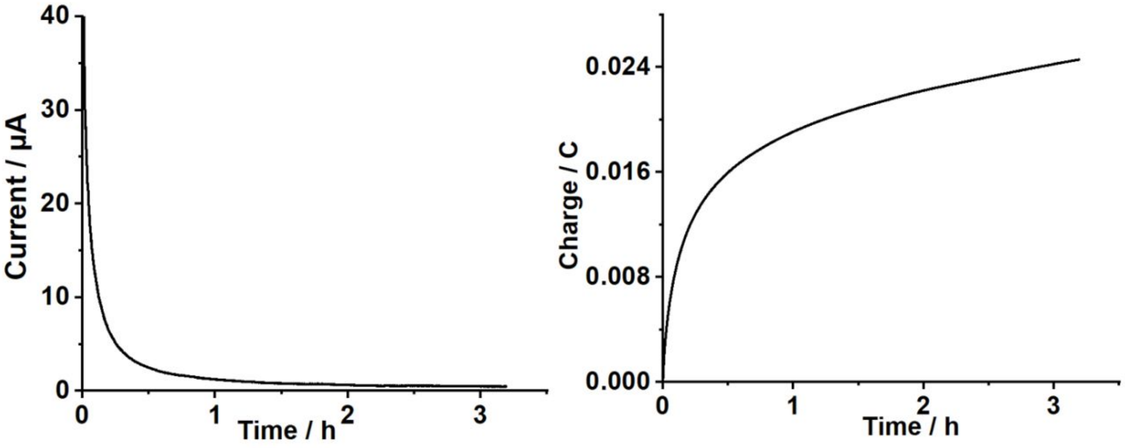

Figure S30 CPE without catalysts (7 mL CH_3CN; [NH_4OTf] = 0.1 M; [NH_3] = 0.2 M; potential = 0.65 V vs $Fc^{+/0}$).

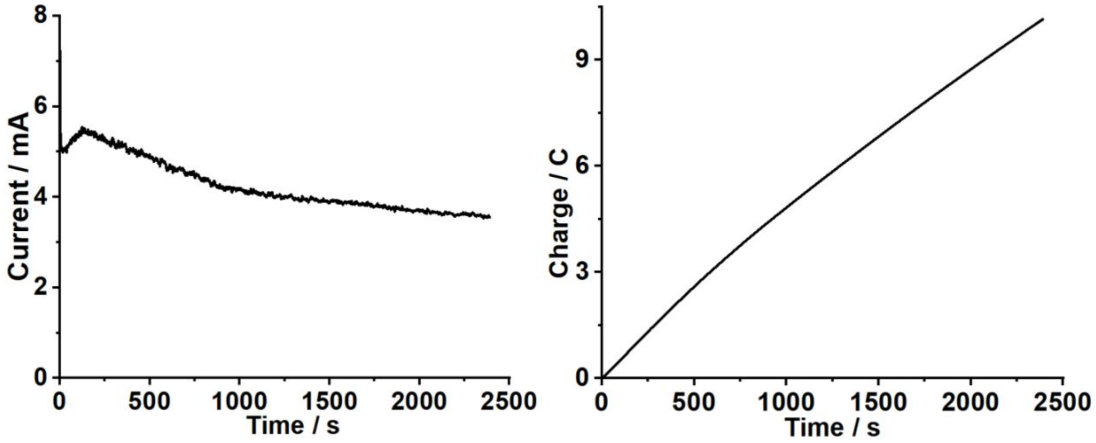

Figure S31 CPE using **$RuNH_3$** catalyst (5 mL CH_3CN; [Ru] = 0.2 mM; [$^{n}Bu_4NPF_6$] = 0.1 M; [$^{15}NH_3$] = 0.4 M; potential = 0.65 V vs $Fc^{+/0}$).

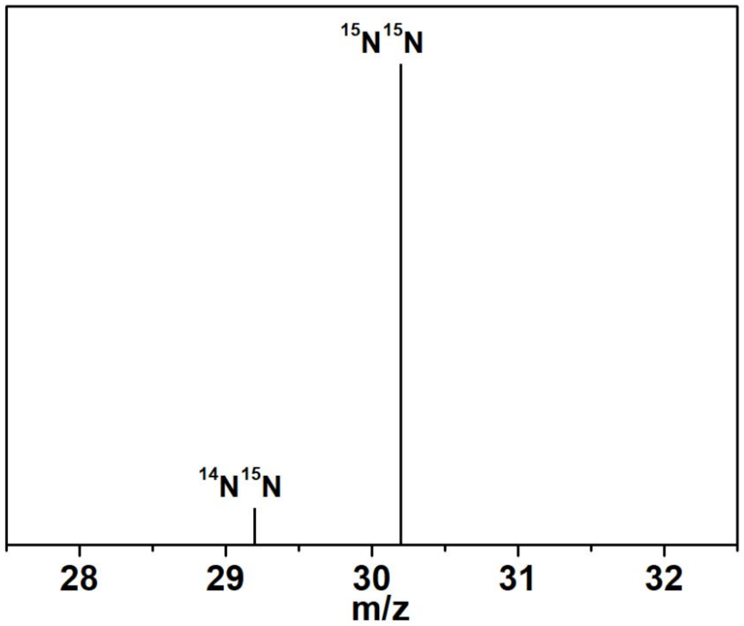

Figure S32 N_2 distribution after CPE for 2300s using **$RuNH_3$** catalyst (5 mL CH_3CN; [Ru] = 0.2 mM; [$^nBu_4NPF_6$] = 0.1 M; [$^{15}NH_3$] = 0.4 M; potential = 0.65 V vs $Fc^{+/0}$). N_2 was corrected using O_2 (Figure S29). $^{14}N^{14}N_{background} = O_{2(sample)} / O_{2(air)} \times {}^{14}N^{14}N_{(air)}$; $^{14}N^{15}N_{background} = O_{2(sample)} / O_{2(air)} \times {}^{14}N^{15}N_{(air)}$.

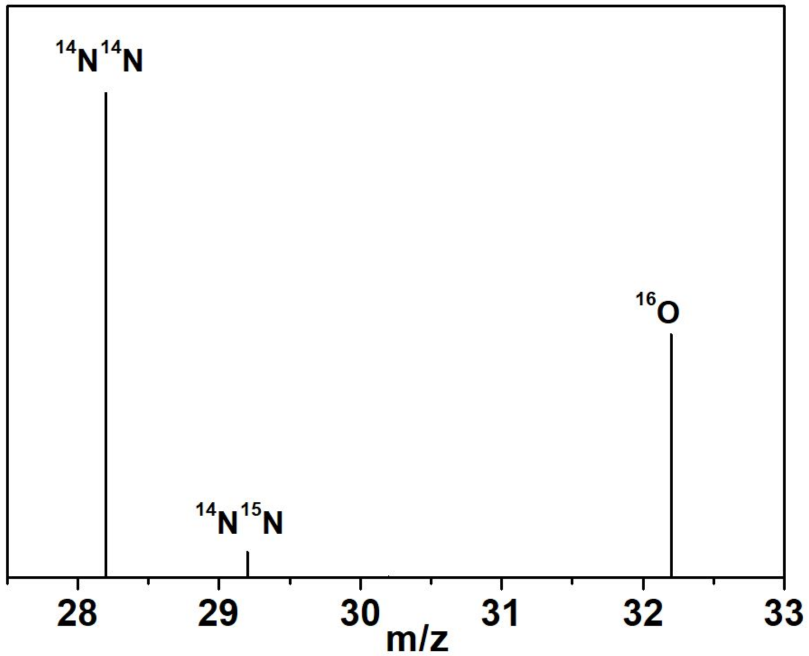

Figure S33 GC of air showing N_2 distribution and O_2 of air.

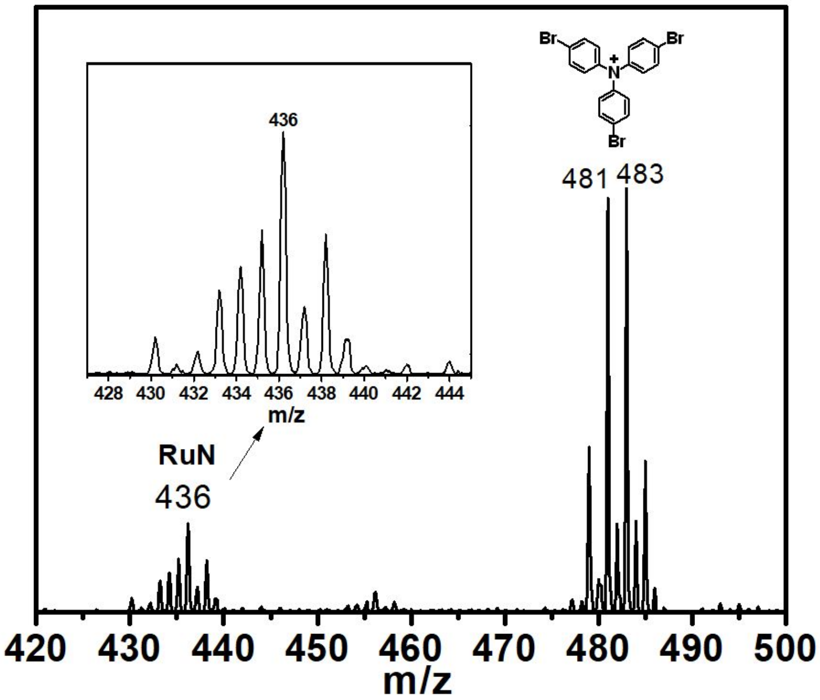

Figure S34 ESI/MS of the reaction solution of **$Ru^{14}NH_3$** (1 mM) with $^{14}NH_3$ (20 mM) and $[(p\text{-}BrC_6H_4)_3N][SbCl_6]$ (50 mM) in CH_3CN.

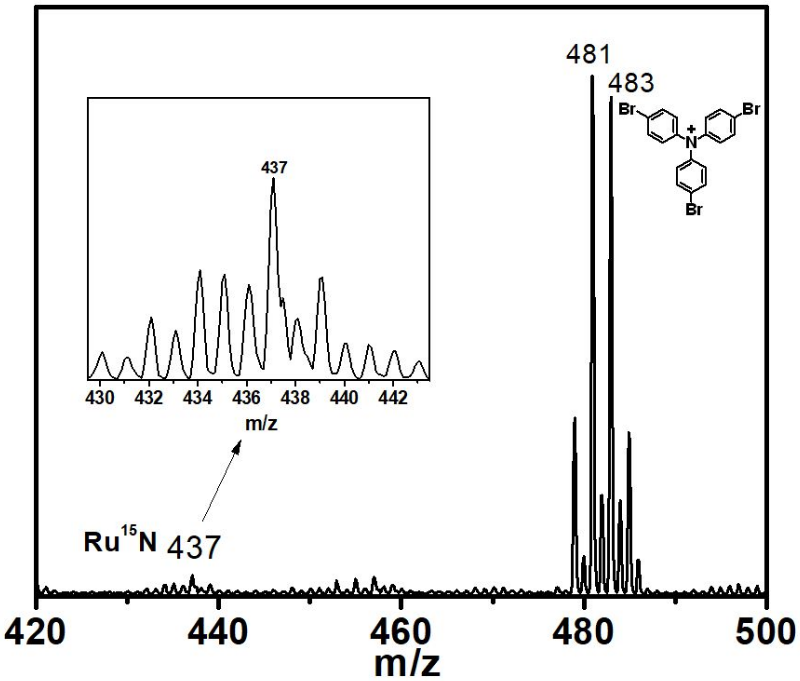

Figure S35 ESI/MS of the reaction solution of **$Ru^{14}NH_3$** (1 mM) with $^{15}NH_3$ (10 mM) and $[(p\text{-}BrC_6H_4)_3N][SbCl_6]$ (50 mM) in CH_3CN.

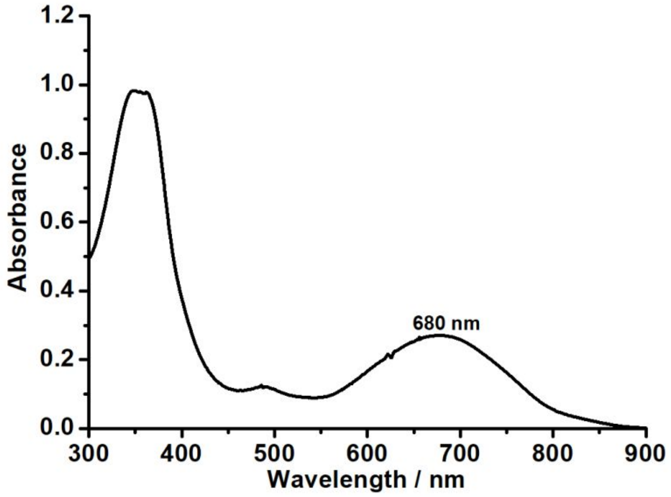

Figure S36 UV/vis Spectrum of **$RuNH_3$** (0.06 mM) in CH_3CN.

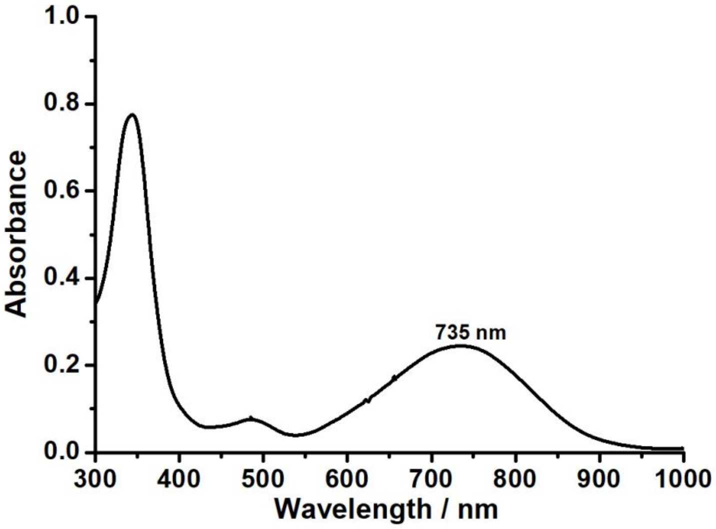

Figure S37 UV/vis Spectrum of $[Ru(salchda)(CH_3CN)_2]^+$ (0.06 mM) in CH_3CN.

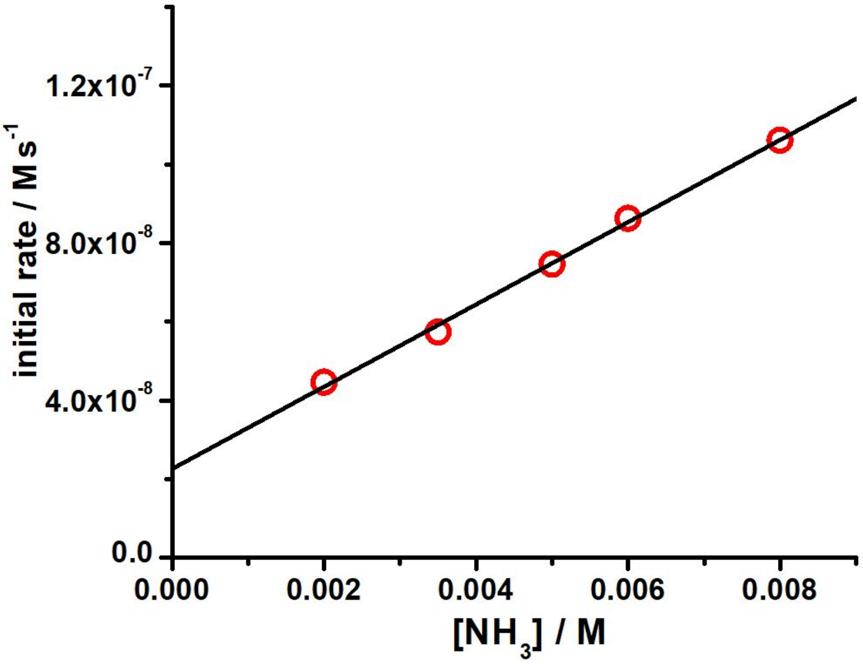

Figure S38 Plot of initial rate vs [NH_3] for the reaction of **RuN** with NH_3 in CH_3CN at 15.0 °C. Slope = (1.05 ± 0.03) × 10^{-5}, *y*-intercept = (2.26 ± 0.16) × 10^{-8}, r^2 = 0.999.

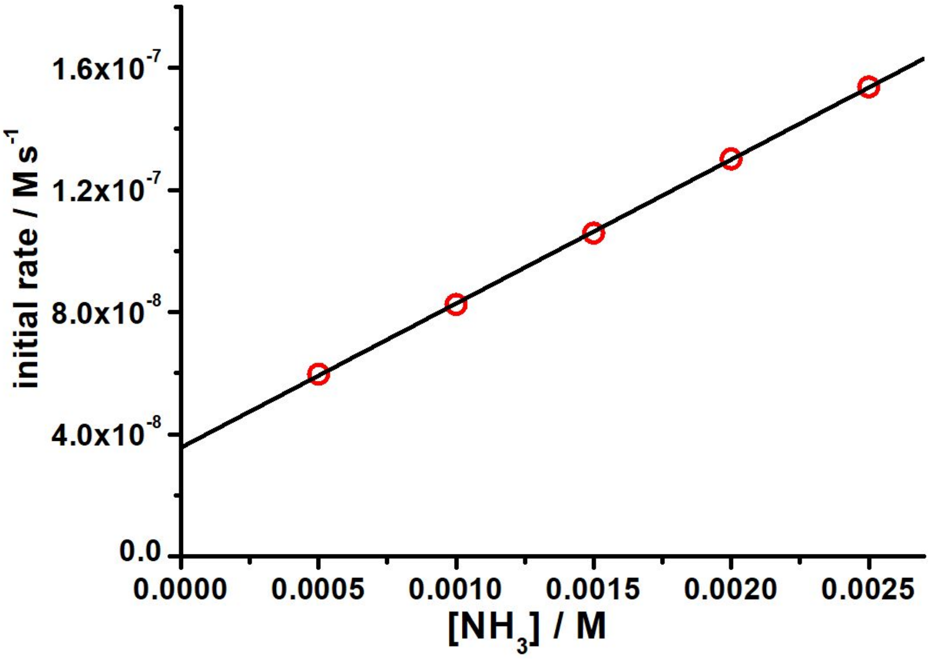

Figure S39 Plot of initial rate vs [NH_3] for the reaction of **RuN** with NH_3 in CH_3CN at 35.0 °C. Slope = (4.71 ± 0.03) × 10^{-5}, *y*-intercept = (3.57 ± 0.05) × 10^{-8}, r^2 = 0.9999.

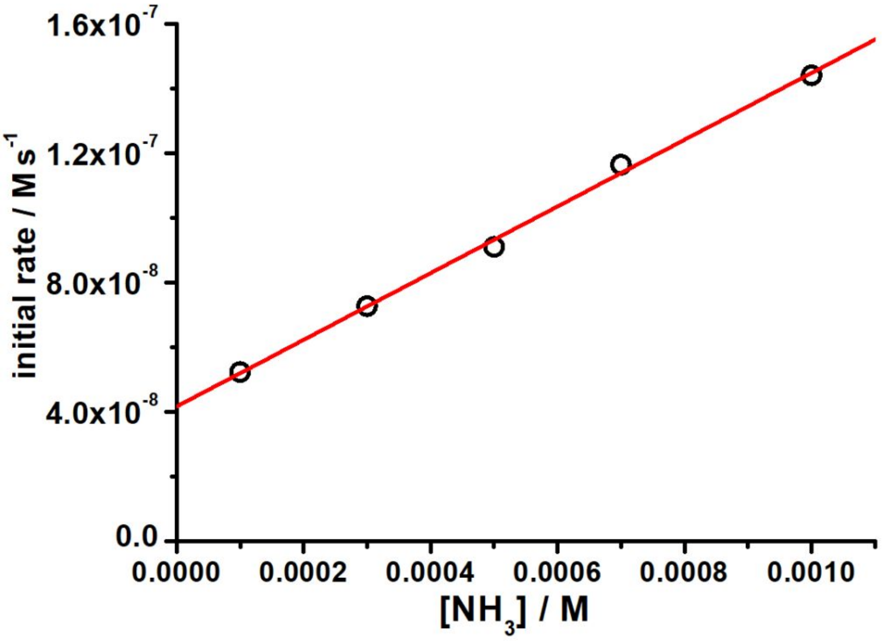

Figure S40 Plot of initial rate vs [NH_3] for the reaction of **RuN** with NH_3 in CH_3CN at 45.0 °C. Slope = (1.03 ± 0.03) × 10^{-4}, *y*-intercept = (4.17 ± 0.17) × 10^{-8}, r^2 = 0.999.

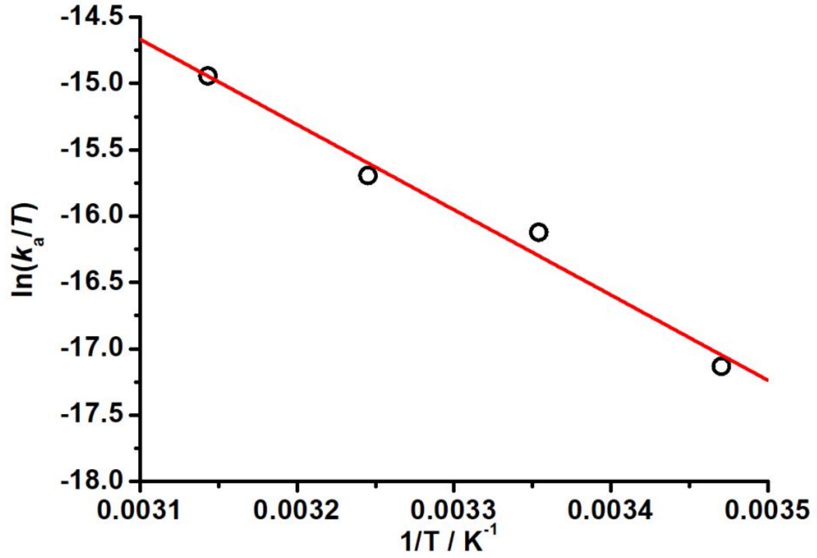

Figure S41 Plot of ln(k_a/T) vs 1/T for the reaction of **RuN** with NH_3 in CH_3CN. Slope = –(6.43 ± 0.63) × 10^3, *y*-intercept = (5.25 ± 2.09), r^2 = 0.97.

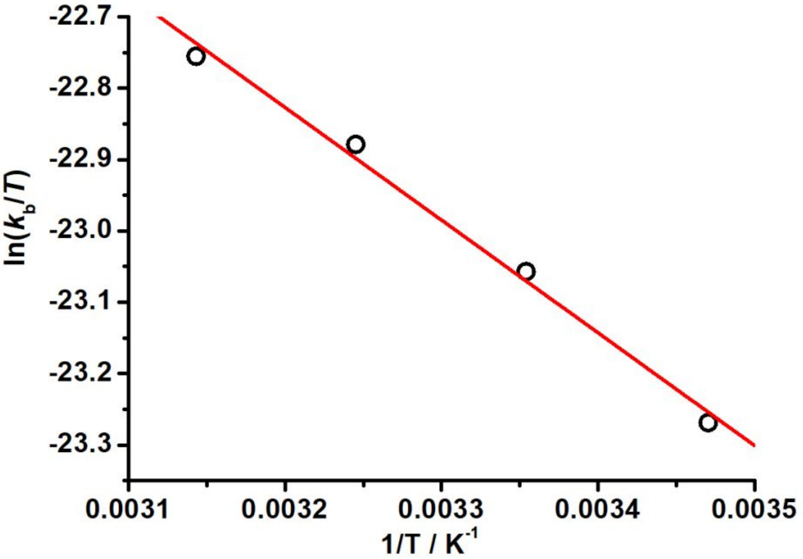

Figure S42 Plot of ln(k_b/T) vs 1/T for the reaction of **RuN** with NH_3 in CH_3CN. Slope = –(1.58 ± 0.10) × 10^3, *y*-intercept = –(17.3 ± 0.3), r^2 = 0.99.

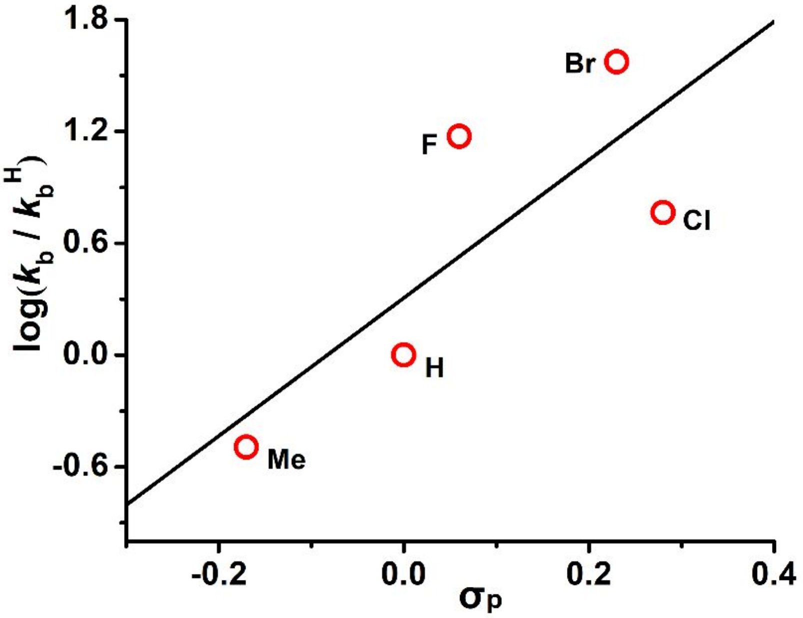

Figure S43 Plot of log(k_b/k_b^H) versus σ_p for the reaction of ^{X}**RuN** with NH_3 in CH_3CN at 25.0 ºC. Slope = (3.7 ± 1.6), r^2 = 0.51.

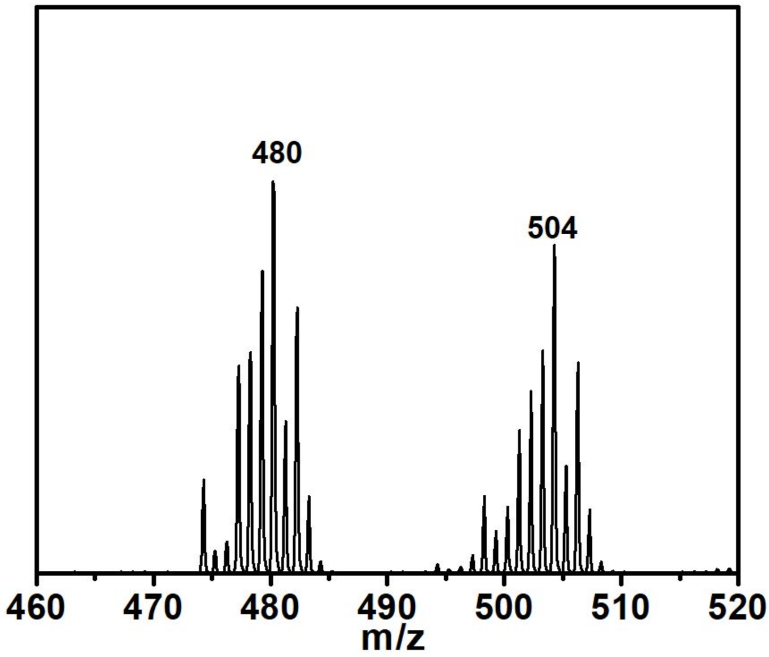

Figure S44 ESI/MS of the reaction of **Ru^{14}N** with $^{14}NH_3$ in CH_3CN.

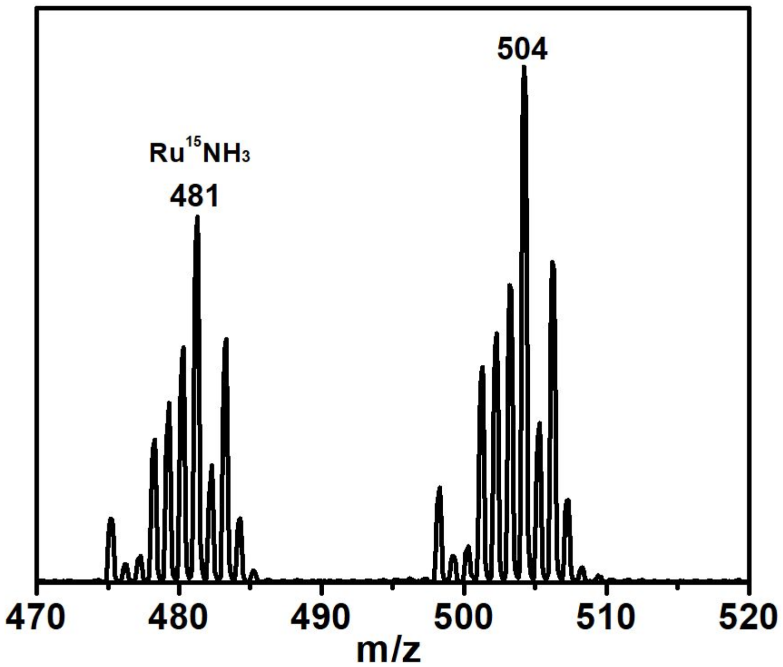

Figure S45 ESI/MS of the reaction of **Ru^{14}N** with $^{15}NH_3$ in CH_3CN.

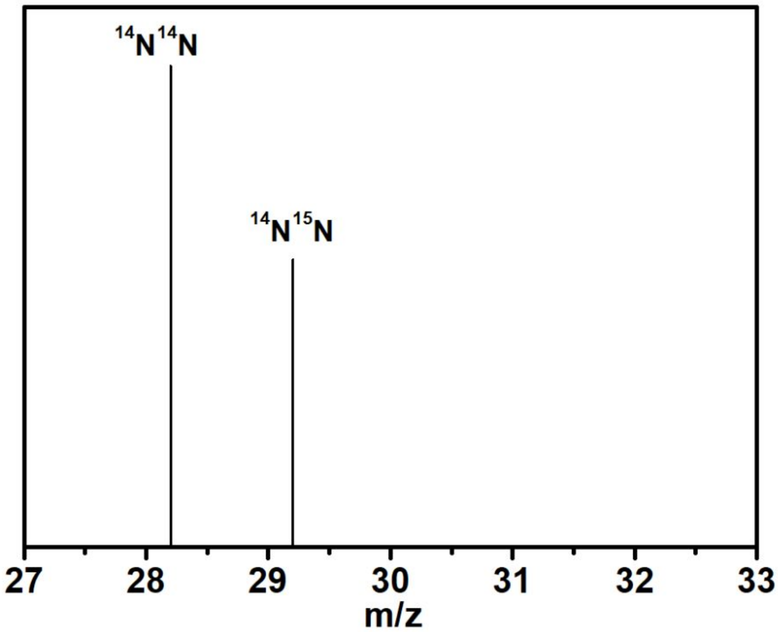

Figure S46 N_2 isotope distribution for the reaction of $^{15}NH_3$ (2 mM) with **Ru^{14}N** (0.15 mM) in 60 mL CH_3CN at 20 ºC under Ar. $^{14}N^{14}N$ has been correct based on O_2 and the percentage was found to be 62.6%. The percentage of $^{14}N^{15}N$ was 37.4%. No $^{15}N^{15}N$ was observed.

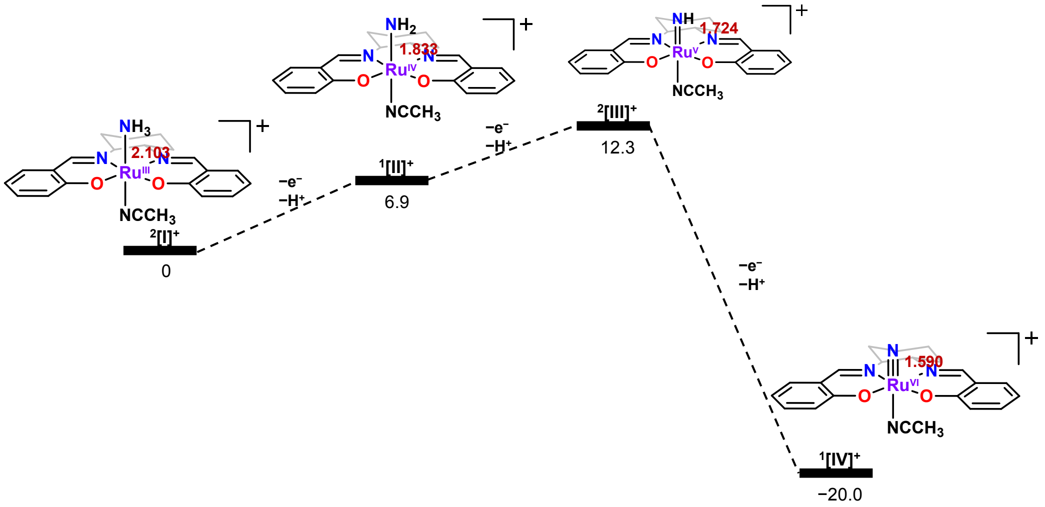

Figure S47 Free energy profile for the oxidation of **$RuNH_3$** to **Ru≡N** with CH_3CN as ancillary ligand. Relative Gibbs free energies at 298 K in CH_3CN are given in kcal mol^{-1}.

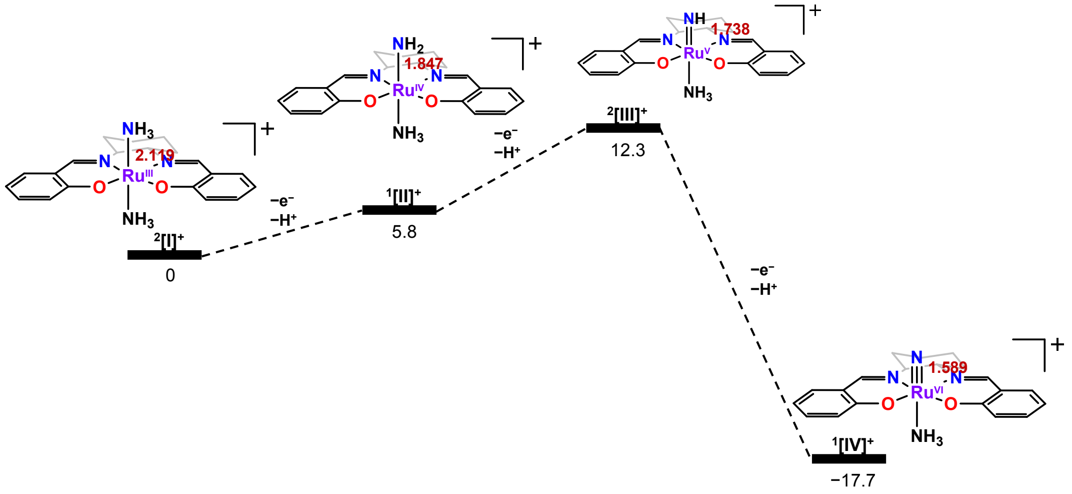

Figure S48 Free energy profile for the oxidation of **$Ru(NH_3)_2$** to **Ru≡N** with NH_3 as ancillary ligand. Relative Gibbs free energies at 298 K in CH_3CN are given in kcal mol^{-1}.

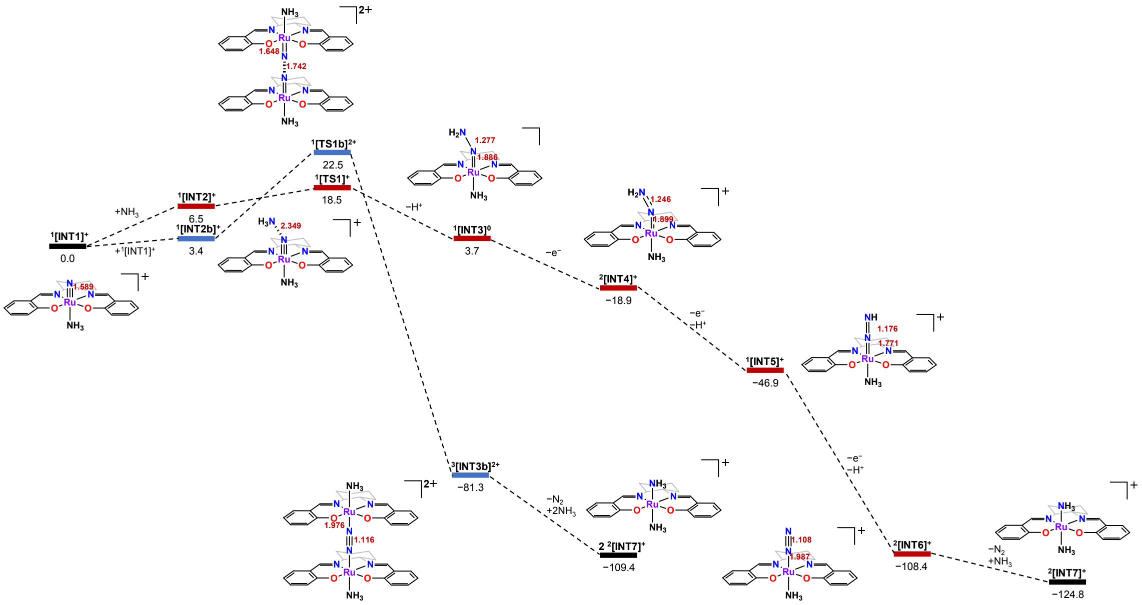

Figure S49 Free energy profile for the N_2 formation starting from **RuN** with NH_3 as the ancillary ligand. Relative Gibbs free energies at 298 K in CH_3CN are given in kcal mol^{-1}, bond lengths are given in Å.

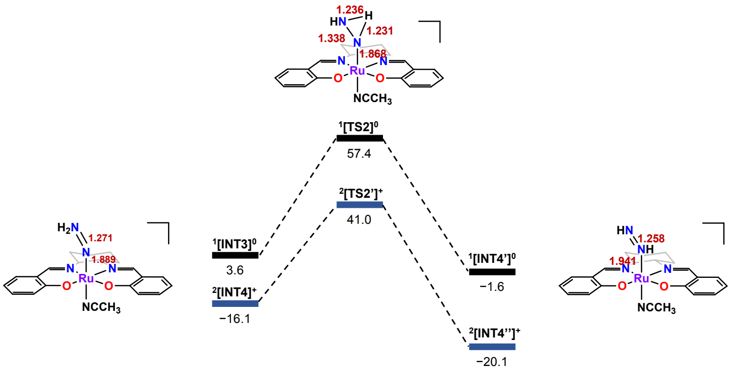

Figure S50 Free energy profile for the formation of a diazene intermediate. Relative Gibbs free energies at 298 K in CH_3CN are given in kcal mol^{-1}, bond lengths are given in Å.

Table S1 Crystal data and structure refinement for **[^{Br}RuNH$_3$]PF$_6$**.

Identification code	**[^{Br}RuNH$_3$]PF$_6$**
Empirical formula	$C_{27}H_{36.5}Br_2F_6N_4O_{3.25}PRu$
Formula weight	874.96
Temperature/K	100.15
Crystal system	triclinic
Space group	P-1
a/Å	11.834(2)
b/Å	13.279(2)
c/Å	13.570(2)
α/°	118.205(4)
β/°	97.287(4)
γ/°	105.076(4)
Volume/Å^3	1734.7(5)
Z	2
ρ_{calc}g/cm^3	1.675
μ/mm^{-1}	2.868
F(000)	871.0
Crystal size/mm^3	0.3 × 0.1 × 0.1
Radiation	MoKα (λ = 0.71076)
2Θ range for data collection/°	4.336 to 50.3
Index ranges	$-14 \leq h \leq 13$, $-15 \leq k \leq 15$, $-15 \leq l \leq 16$
Reflections collected	21255
Independent reflections	6038 [R_{int} = 0.0811, R_{sigma} = 0.0932]
Data/restraints/parameters	6038/0/346
Goodness-of-fit on F^2	1.054
Final R indexes [I>=2σ (I)]	R_1 = 0.0676, wR_2 = 0.1807
Final R indexes [all data]	R_1 = 0.1007, wR_2 = 0.1983
Largest diff. peak/hole / e Å^{-3}	1.27/-0.92

Table S2 Summary of the rate constants from the plot of initial rate vs [^{X}**RuN**] for the reactions with NH_3 (2.0×10^{-3} M) in CH_3CN at 25.0 ºC.

^{X}**RuN**	k_a / $M^{-1}s^{-1}$	k_b / $M^{-1}s^{-1}$
**H**	$(3.53 \pm 0.10) \times 10^{-1}$	9.14 ± 0.32
**F**	0.53 ± 0.12	$(4.87 \pm 0.28) \times 10^{1}$
**Br**	2.83 ± 0.65	$(3.14 \pm 0.16) \times 10^{2}$
**Me**	$(2.23 \pm 0.53) \times 10^{-2}$	2.69 ± 0.14
**Cl**	4.19 ± 0.35	$(1.25 \pm 0.08) \times 10^{2}$

Coordinates of INTs and TSs

Fc

Fe	0.00000001747779	0.00000028349968	0.00000039138471
C	0.29428711758924	2.04294626070043	-0.10289498909474
C	1.06404243314785	1.37909614031596	-1.10884439623301
C	1.95728035195794	0.47324324229618	-0.45556540985248
C	1.73982584340546	0.57763974399168	0.95389956407373
C	0.71188291603083	1.54750374233754	1.17186151950388
H	-0.48865114806042	2.78006758715108	-0.27805052291245
H	0.97026817965803	1.52151354433358	-2.18490727982733
H	2.66249176590220	-0.19646915244514	-0.94660769966204
H	2.25019825467915	0.00126103722089	1.72479224908069
H	0.30271128427977	1.84012071460188	2.13829623539807
C	-0.29428649177240	-2.04294593254291	0.10289483064472
C	-1.06404248490952	-1.37909656628121	1.10884431693261
C	-1.95728052953646	-0.47324376981737	0.45556546527569
C	-1.73982567035873	-0.57763966964236	-0.95389938788379
C	-0.71188230502650	-1.54750314416144	-1.17186157573647
H	0.48865195330217	-2.78006691815571	0.27805027788059
H	-0.97026904184995	-1.52151443565273	2.18490714828024
H	-2.66249311300688	0.19646764442499	0.94660762821527
H	-2.25019874071714	-0.00126107322502	-1.72479188628226
H	-0.30271059219243	-1.84011927895000	-2.13829647918561

Fc^{+}

Fe	-0.00000083692662	0.00000017719627	0.00000216857185
C	0.34461296079521	2.09458812399435	-0.11887934259110
C	1.12605251787969	1.44709614656497	-1.11715816829379
C	1.99777828178246	0.52479626116306	-0.47079698725088
C	1.76019894465341	0.60222325351148	0.93686249806934
C	0.73337522493895	1.57646187164278	1.15457190587542
H	-0.44462889201248	2.82347097062951	-0.30039532273407
H	1.02904338114241	1.58684208561989	-2.19345693864194
H	2.69365021113073	-0.15030285909320	-0.96772246086320
H	2.25919081505708	0.01197075888921	1.70434187073389
H	0.30884367949198	1.85883410852762	2.11713326611178
C	-0.34460392436449	-2.09461575154201	0.11870991268819
C	-1.12596240271928	-1.44724512959165	1.11712535142909
C	-1.99773008869792	-0.52485813994075	0.47094882620735
C	-1.76026213017170	-0.60211010137869	-0.93674159832868
C	-0.73346138768462	-1.57633437995618	-1.15465036995661
H	0.44465421607337	-2.82351771454203	0.30007897745622
H	-1.02885945146809	-1.58711330978148	2.19339998503080
H	-2.69355620106084	0.15018456124020	0.96801488202106

H	-2.25932115946083	-0.01176950806943	-1.70410904125339
H	-0.30901375837842	-1.85860142508392	-2.11727941428135

NH_3

N	0.51545620876867	0.66283680581522	-0.16558526837641
H	1.32915229253097	0.40187255095549	0.39943167461196
H	0.88798720968392	1.33773124384489	-0.84025356839912
H	0.32625428901644	-0.17195060061561	-0.72842283783642

NH_4^+

N	0.56930810303473	0.67284587702335	-0.16536662863155
H	1.35862180770495	0.40585487637029	0.43661057408282
H	0.88070991240552	1.38720638301673	-0.83565230208148
H	0.23468891801671	-0.15402291938123	-0.67608508291399
H	-0.19106874116191	1.05147578297085	0.41343343954419

Coordinates of Ru(NH$_3$)(MeCN)

[1][**INT1**]$^+$

Ru	-0.02983499659757	0.49247360490950	0.69841448784335
N	-0.10622529988390	0.84057718911494	2.24888151616403
C	-3.27729932570059	0.51048444251722	-0.11211772178090
C	-4.67544535861527	0.57387032689838	-0.36617184273831
C	-5.47890961024213	-0.54573433367664	-0.26760873970930
C	-4.88932895396937	-1.78003187657632	0.07615308865473
C	-3.52784116830669	-1.88067658117491	0.32053419458273
C	-2.68483363972407	-0.74558395548794	0.24308718988458
C	-2.52564487448024	1.70902835068969	-0.32972096146916
N	-1.26033354659670	1.89641521516770	-0.10441736802247
O	-1.40148365089490	-0.93040995299359	0.46952472823046
N	1.38022596823647	1.84318535482786	0.15810345716966
C	2.60607682361722	1.55732904586747	-0.14396392852022
C	3.20860428663933	0.25397853302489	-0.13092683782520
O	1.25031543960505	-1.02018346990901	0.58759826151496
C	2.51753342264816	-0.94896935019709	0.22910939626225
C	4.56929334247913	-2.20216687777649	-0.19832154402252
C	3.23477490320778	-2.16760109982524	0.18043116931565
C	-0.54263859912552	3.09643887367117	-0.60737032777883
C	0.79681224460002	3.19669247587117	0.13743282812474
H	-3.08579934459436	2.54346647275551	-0.76847038604685
C	-1.31194797677390	4.41303891259305	-0.50772749147467
H	1.91523972943513	4.06903417487843	-1.49135624193706
H	2.61261695919431	4.37344922056324	0.11759336322909
C	4.57453662073837	0.18073763077945	-0.51496564832145
H	2.69951625186683	-3.08074964465411	0.44812918843284
C	5.25474949113319	-1.02298397596451	-0.55054794670721
H	-5.10402677615669	1.53847051077744	-0.65023174741191
H	5.09057436845968	-3.16288102087723	-0.22586291574735
H	-3.06862286274581	-2.83724830364623	0.57719750011806
H	5.08372916074527	1.10906816107579	-0.78659249529893
H	-6.55076468570285	-0.48016927471684	-0.46314256712462
H	6.30397316496796	-1.06006949666845	-0.84868320304271
H	3.26077293144407	2.38205568315645	-0.44844915384335
H	-5.51119096127805	-2.67669755795374	0.14562104083137
H	-0.31267198964345	2.89034442948802	-1.66779836127610
C	-0.45120965731499	5.55932778619007	-1.05596620527055
H	-2.25148237757901	4.35919039829660	-1.07681326102048
H	-1.57317235252024	4.59460653676787	0.54903188307944
C	0.91152082622403	5.63831026903623	-0.36299017707155
H	-0.30206402013263	5.40735890024939	-2.13944463597184
H	-0.99496359193805	6.50989574451790	-0.94019717622600
C	1.66725045161957	4.30525459831204	-0.44213669381259

H	1.52462463440110	6.43404651832544	-0.81398378417415
H	0.76902939830263	5.90769921278000	0.69821390999066
H	0.57440468672602	3.44006643744130	1.19208344886727
N	0.15493105936051	0.09374372471681	-1.83588999996780
C	0.25877043088854	-0.04703037809071	-2.97852800339967
C	0.39227668640446	-0.21415129428403	-4.41489521058326
H	-0.02553018915105	-1.18593407613793	-4.71818604892297
H	-0.14973988483714	0.59223555400379	-4.93197025722095
H	1.45640241156036	-0.17330176865390	-4.69300176855486

[1][INT3][0]

Ru	0.13728244144275	0.46730404740780	0.21751517005559
N	0.01328953424342	0.77662141904985	2.07679678610840
C	-3.18058608003341	0.58542596904550	0.34243247975936
C	-4.58199738799290	0.72662373817108	0.52607103406812
C	-5.40022883817651	-0.32788616810725	0.89542691035259
C	-4.81656121940926	-1.59846346243481	1.08211868328633
C	-3.45686130097182	-1.78342852315832	0.89950011669277
C	-2.57861409789676	-0.71812006455732	0.53019898512314
C	-2.47863890517658	1.78399418793950	-0.07643904938965
N	-1.19704491707556	1.91050017353690	-0.19272730723155
O	-1.31747326567851	-0.99711443698396	0.37773668864595
N	1.47011836793847	1.95662323751896	0.02143095984289
C	2.75329702935524	1.79705267120780	-0.00911921424662
C	3.46517222438398	0.54446248128120	0.15546838278687
O	1.58461078734657	-0.97534037992322	0.52492603935827
C	2.86001951698228	-0.74413505975786	0.42198001890768
C	5.12564206028897	-1.72144230637864	0.48521687717560
C	3.75075430034275	-1.85126295443598	0.57868438887071
C	-0.54290927012352	3.11779636266899	-0.72831138226891
C	0.81462080244086	3.27512303645348	-0.00850089546853
H	-3.12202214260234	2.63974806053563	-0.31691625629951
C	-1.34931596465955	4.41464335026801	-0.66804081990081
H	1.89032352855735	4.17701239636715	-1.64795656899946
H	2.56403608009923	4.56100615312480	-0.05173001333837
C	4.88065972256834	0.63080852573302	0.06598563158429
H	3.29310892150124	-2.82364823583382	0.77940975943883
C	5.71188708085034	-0.46528831905142	0.22372802968812
H	-5.01785571300434	1.71777712033604	0.36603903174259
H	5.75978678975064	-2.60365661333754	0.61529375577272
H	-3.00173681713578	-2.76727834112474	1.04137154349584
H	5.31723005808810	1.61472223638350	-0.13278995639986
H	-6.47324620437089	-0.17942285476570	1.03499018057476
H	6.79638400155431	-0.35842830401311	0.15018221147114
H	3.39371556509096	2.67579084908297	-0.15624193006762
H	-5.44098161916149	-2.44872159062759	1.37276741840803
H	-0.31557875436010	2.89815459415439	-1.78854855922028
C	-0.54108019365093	5.57720814151545	-1.25679876171420
H	-2.29297296041724	4.30347332226042	-1.22342255199635
H	-1.61181039545051	4.62864563059682	0.38309650458171
C	0.81519014756576	5.72966410095916	-0.56516774672426
H	-0.38161672267431	5.39510285204297	-2.33473251489532
H	-1.12045473404442	6.51103309575864	-1.17560854357928
C	1.62274581721049	4.42678457768768	-0.60587095773177
H	1.39597719995246	6.54106042539383	-1.03296196943902

H	0.65595698796885	6.02108833445091	0.48855719220631
H	0.58486968363366	3.51897460713708	1.04665637342743
N	0.32492934917799	0.10499185979950	-1.86210212651892
C	0.50573204019385	-0.10606276621967	-2.98537997383388
C	0.73826472528220	-0.37325023408926	-4.39430038344552
H	-0.09128546020167	-0.97109612212273	-4.80188008946569
H	0.80795494922426	0.57439592983721	-4.94979562113114
H	1.67839624716059	-0.93330315916615	-4.51445321565157
N	0.05104445929236	-0.23972406780386	2.83857979582098
H	-0.00771569617525	-0.12874402287068	3.85382197067557
H	0.16376824095540	-1.19062550094267	2.46004348903548

^{2}[INT4]$^{+}$

Ru	0.02544597513015	0.42029408812012	0.21922175645750
N	-0.38057993230228	0.82499484458759	2.07004962863610
C	-3.26753989714310	0.60864312356876	0.35766442663066
C	-4.64904613413353	0.76120841829370	0.64850139831585
C	-5.44756991079462	-0.31547343515576	0.98957790764683
C	-4.87855553567120	-1.60687957389893	1.02170239961559
C	-3.54084495196800	-1.80031698950684	0.72171498605201
C	-2.68758384030754	-0.70966972154496	0.39875621964910
C	-2.55470815368634	1.79346443510716	-0.05083578819627
N	-1.27736852836130	1.87311143645058	-0.25278862494263
O	-1.43606285690909	-0.96684379011350	0.12217175659176
N	1.37625044540221	1.91893482567612	0.09365880963136
C	2.64683977453831	1.74318087766520	-0.06143033332686
C	3.33200469394102	0.47063959487268	-0.00197460116070
O	1.46841286521098	-0.85714212739624	0.82716306448736
C	2.72733143499417	-0.75337542240062	0.44483163596990
C	4.88062098688506	-1.87593543319700	0.15168564448979
C	3.54276367396561	-1.90939670345637	0.51875752344357
C	-0.59143827200993	3.08664041076207	-0.73601663890142
C	0.71491562871888	3.23546982336705	0.08364801220623
H	-3.17224103715724	2.68345197350830	-0.21920764946124
C	-1.40663203617728	4.37682856763850	-0.70866645865530
H	1.89990716722816	4.19659914520359	-1.44324985792471
H	2.45019209595188	4.52327368215362	0.21508214660415
C	4.70679687030839	0.47226572537453	-0.34744366536835
H	3.07630635176830	-2.83305409202356	0.86828161848919
C	5.47650664064328	-0.67834795769010	-0.28843293789691
H	-5.07701356499440	1.76636429989920	0.59985848885120
H	5.47629682263936	-2.79108009060834	0.20874756385567
H	-3.09503577369188	-2.79730842580600	0.73943099299642
H	5.15670718443225	1.41551684890893	-0.66964056195249
H	-6.50460077672682	-0.17067899011187	1.22161120131686
H	6.53123243832527	-0.65458236470579	-0.56988326774751
H	3.28307078922024	2.61631469766104	-0.24758705357442
H	-5.49981645186146	-2.46756214834056	1.28439169808915
H	-0.29152282542954	2.87320377721143	-1.77814480622413
C	-0.56007223075768	5.55659984211283	-1.20150298867697
H	-2.30184416482809	4.27074219581142	-1.34015554383173
H	-1.75029762852622	4.56606415359985	0.32331627035687
C	0.73181573260168	5.70006301528719	-0.39404746812562
H	-0.31046687082336	5.40307449419031	-2.26631369637118
H	-1.15316007163401	6.48296291012324	-1.14435684016480
C	1.55549636151434	4.40707862401561	-0.41568734155466
H	1.34040030640706	6.53022960813655	-0.78608863324957

H	0.48378817818540	5.95297291495735	0.65200014969162
H	0.41473872230816	3.43089846266006	1.12941835464120
N	0.51498515758402	0.00759214392917	-1.77708059605258
C	0.91694681441583	-0.19728440103440	-2.84036231783847
C	1.43554780548907	-0.44151110932151	-4.17208165196087
H	1.07048622953462	-1.41214010152658	-4.54073671164260
H	1.10151167441494	0.35753908629893	-4.85136191574639
H	2.53590508513652	-0.45494813529625	-4.13662811441606
N	0.32705181759851	0.35905342324424	2.97883140706325
H	0.11440468603342	0.57258979841580	3.96192680080432
H	1.12750103536785	-0.26716025567767	2.76593420238088

^{1}[INT4]$^{2+}$

Ru	0.01280111643975	0.40807540487577	0.20810594757530
N	-0.41345334869137	0.70132185880234	2.06218477345791
C	-3.26128583715486	0.62219355782111	0.42119033607128
C	-4.63541411563134	0.74895737607878	0.74100090382493
C	-5.41832614987397	-0.36222777186039	0.99415669575858
C	-4.86020021180007	-1.66100702964050	0.88791559137154
C	-3.53674305321687	-1.83419384307463	0.54198420642929
C	-2.68743988737833	-0.70707607480317	0.34195147095182
C	-2.55389911379022	1.80605598664259	0.03516586911562
N	-1.28035944108697	1.85581789879376	-0.23158862373353
O	-1.44055288881508	-0.92852147858167	0.06616692647505
N	1.35747956810405	1.88162908804554	0.09591608846868
C	2.62632988091025	1.70035461163806	-0.12192504579544
C	3.30095979926971	0.43371120727608	-0.07217430264774
O	1.42855814161903	-0.86076453571222	0.75866360472624
C	2.68698104518864	-0.77997364352847	0.41485109980087
C	4.82790609712070	-1.92217708205587	0.20977007412484
C	3.49373902359393	-1.93834249803425	0.57146118394193
C	-0.59923867464429	3.07169850579959	-0.71645702922085
C	0.71474899641698	3.20845215093057	0.09983146587781
H	-3.15441491245761	2.71535942031843	-0.07226814643509
C	-1.40846544564555	4.36376153045192	-0.67803479401410
H	1.89752834917974	4.16216969268810	-1.43321959823670
H	2.45949646561182	4.48113943023863	0.22504734629629
C	4.67789667966798	0.41503019702852	-0.40002404528880
H	3.01619226660643	-2.83945693628105	0.96019030061333
C	5.42970412320167	-0.74180874860381	-0.28276566373019
H	-5.06977760849806	1.75061337434133	0.78135018381958
H	5.42532413050231	-2.83145966708721	0.31141000714356
H	-3.08902056044133	-2.82547269310583	0.45261912057119
H	5.14232515540758	1.34097872933189	-0.74795674017168
H	-6.47118755549111	-0.24272965075751	1.25690645708467
H	6.48716275487753	-0.73946999190231	-0.55399325173204
H	3.25492367363350	2.57182387550283	-0.33306149399101
H	-5.48824806107757	-2.53464951943128	1.07808071364740
H	-0.30722450423052	2.85613060222448	-1.75955082336800
C	-0.55284485504083	5.53624167964907	-1.17424358442597
H	-2.30644283600061	4.26451128637765	-1.30650074439634
H	-1.74285675540812	4.55408600901307	0.35643267818460
C	0.74384672042836	5.66920003242130	-0.37286733541815
H	-0.31115545073642	5.38161479686366	-2.24045806683480
H	-1.13902982987143	6.46615162953011	-1.11180633645517
C	1.56205717042840	4.37239899761785	-0.40294876413171
H	1.35596504794053	6.49526831459477	-0.76694061490342

H	0.50404403495204	5.92078273537546	0.67515345416468
H	0.42323644336060	3.40084830577138	1.14756905213408
N	0.51594756672992	0.01615033865464	-1.78450193992087
C	0.92467866315543	-0.18187592789982	-2.84603731241702
C	1.44404833365872	-0.42203368941968	-4.17558995510423
H	1.36444136781203	-1.49363856212226	-4.41398948005047
H	0.86473889962903	0.16284017990257	-4.90645550271210
H	2.50050083519662	-0.11361041467604	-4.21127562376440
N	0.40058028915988	0.65815000723504	2.98767348037964
H	0.07847657697070	0.78106951390317	3.95932716517579
H	1.41314188020874	0.48307143283756	2.85078862171335

^{2}[INT5]^0

Ru	0.05785789393129	0.46598429268855	0.34356241807083
N	0.02153505136706	0.75877489758699	2.21407963494234
C	-3.28024540895243	0.58800777278496	0.25472963640662
C	-4.69291777682821	0.73223537875974	0.32379326962213
C	-5.53515865714538	-0.31974522470421	0.63595706366077
C	-4.96636951110608	-1.58868497217353	0.88148245856547
C	-3.59752678013008	-1.77569555851159	0.81540075017487
C	-2.69315803403793	-0.71105253698492	0.50833986886221
C	-2.54571449388594	1.77459262200251	-0.12001391601756
N	-1.25760505386142	1.89843630197217	-0.16818284128855
O	-1.42640747916603	-0.98400057204653	0.46667209632570
N	1.41221378234369	1.94541885542438	0.12927054067329
C	2.68420562797571	1.77282912253449	-0.00229956305351
C	3.38408323669916	0.50388326480374	0.06519220455412
O	1.53033185396967	-0.93257588524000	0.75620727084640
C	2.78605908010811	-0.75621870565817	0.43624126440923
C	4.99499988710667	-1.80996319404321	0.16156736950159
C	3.64575989049843	-1.89215038505372	0.46902564303431
C	-0.57480949883806	3.10551819066896	-0.67638715161862
C	0.75418398426862	3.26333728779748	0.09937717352336
H	-3.16675362308950	2.63653658450206	-0.39368807818323
C	-1.38382630160199	4.40157824536781	-0.64808615924270
H	1.89329860349248	4.18839288032744	-1.48474768902001
H	2.49893090639414	4.54859171886682	0.14509407579185
C	4.77039468984883	0.54833083390874	-0.23306736545572
H	3.19268965560112	-2.84620422463551	0.75012238842578
C	5.57663532277439	-0.57819207012246	-0.19749036446967
H	-5.11388030210483	1.72138892886736	0.11903598222589
H	5.61054486681756	-2.71369691237073	0.19815846041592
H	-3.15510105638639	-2.75735990698466	1.00339907996757
H	5.20398791884600	1.51658126135010	-0.50142080214209
H	-6.61616940455594	-0.17359103786921	0.68674696144988
H	6.63972124332240	-0.51116901762188	-0.43833047354276
H	3.32072650802308	2.64629877461125	-0.18984734955920
H	-5.61300867576945	-2.43650501830319	1.12693407160332
H	-0.30218936800846	2.88358168917556	-1.72543403324976
C	-0.55141316851856	5.57056484164301	-1.18795102249895
H	-2.29826387518104	4.29249065665911	-1.25050541121442
H	-1.69802004433362	4.60660645422364	0.39057454350785
C	0.76882304691374	5.72178160616142	-0.42978415365946
H	-0.33891104808472	5.39761266922272	-2.25811284630499
H	-1.13823537876143	6.50110845429963	-1.12747717189211
C	1.58233489277309	4.42264748039055	-0.45131582469183
H	1.36755961995569	6.54069362010904	-0.85986783954426

H	0.55843607054707	5.99871077249069	0.61866472600709
H	0.48405483485071	3.49127807794679	1.14723690014272
N	0.35865124265410	0.01171688813200	-1.67997092053438
C	0.65670063385246	-0.22454898392608	-2.77169384833683
C	1.03881064064475	-0.51745975074402	-4.14083223323949
H	0.51410434875716	-1.41935163585109	-4.49112464259168
H	0.77537393215616	0.33162390532940	-4.78985295634684
H	2.12537885976767	-0.68789714538476	-4.18996761167673
N	0.65968466128762	0.39562180760259	3.17581786136143
H	1.45470215279879	-0.23540339998232	2.88556855530286

^{1}[INT5]$^{+}$

Ru	0.14903294309696	0.42823027956765	0.38383408048103
N	0.01756416281981	0.76533543278617	2.10844944208873
C	-3.17491012574375	0.51186911107912	0.25273610024261
C	-4.59168340143485	0.63147736192660	0.31879804219405
C	-5.40716414067160	-0.44480557535911	0.60867533859905
C	-4.81266380317708	-1.70729275174651	0.82665453746564
C	-3.44056217313173	-1.86891678712609	0.75831190697192
C	-2.56802780615721	-0.77709001730806	0.48059006741194
C	-2.45898872090827	1.70538712861036	-0.10920009839434
N	-1.17244489457818	1.85899649108141	-0.14030825496124
O	-1.28866889515300	-1.02587673569651	0.43238499097471
N	1.49701416964116	1.90924320781097	0.10711857501671
C	2.76784327819164	1.72390683409281	-0.03349433361631
C	3.46502331039986	0.46303330078206	0.03967465919356
O	1.57405504429954	-1.01536277567685	0.51143564014419
C	2.84905426219661	-0.81359605732117	0.29962583314171
C	5.06572969075476	-1.85408240127965	0.12834022795865
C	3.69909578559650	-1.95466997156415	0.32750441389546
C	-0.50133719051394	3.06879011427918	-0.66166896495445
C	0.83719814577375	3.22777036244573	0.08953608213098
H	-3.08483676517868	2.55830945708564	-0.39696317517191
C	-1.31826045551995	4.35847508891265	-0.60763832625967
H	1.94252499479772	4.15001199016459	-1.51787312544822
H	2.58366602283956	4.50905702410868	0.10045131910116
C	4.87076296951438	0.52282920991189	-0.16223186500247
H	3.22768213582109	-2.92135634682198	0.51856667987331
C	5.67056758265938	-0.60445103218432	-0.11961146505261
H	-5.03117190768857	1.61525632745435	0.13175534387075
H	5.67979081406055	-2.75838733995766	0.16404076627785
H	-2.97875504719004	-2.84482751219634	0.92380769417212
H	5.31787089286050	1.50224843145334	-0.35479398028950
H	-6.49100518666630	-0.32451792110478	0.66127533204562
H	6.74837719729052	-0.52888483366519	-0.27565415215199
H	3.40020444703963	2.59899808024312	-0.22397620707943
H	-5.44317939258068	-2.57211485647412	1.05189027847889
H	-0.25289866392598	2.85000730442806	-1.71668031242348
C	-0.49888313111140	5.53144457188471	-1.15973342311825
H	-2.24312770072566	4.24936632887529	-1.19337504218135
H	-1.61034603185030	4.55472213877969	0.43882774739445
C	0.83905105192534	5.68136913852051	-0.43292161094871
H	-0.31367783181722	5.36631433187648	-2.23587963125555
H	-1.08657504603749	6.45935144302415	-1.07712305652779
C	1.65560588224340	4.38409843029777	-0.47778342087136
H	1.42662093884262	6.50104326675093	-0.87573350706029

H	0.65476979817423	5.95502155060767	0.62105483841047
H	0.59021234972798	3.45566845313962	1.14351856557452
N	0.30492415099001	0.05512403052589	-1.67421484535372
C	0.43848500850900	-0.13861615027405	-2.80306539853138
C	0.60578982069621	-0.38290393821444	-4.22015220146882
H	-0.25104386925958	-0.96295291356923	-4.59568808703377
H	0.66263856071220	0.57838224542119	-4.75318540396720
H	1.53436208467229	-0.95099713538569	-4.38421318410966
N	-0.21682696814487	1.02404115751227	3.22955532532327
H	0.53861165301904	1.00781342748532	3.94554924480017

²[INT5]²⁺

Ru	0.11625898502427	0.44810080601994	0.36507660423197
N	-0.09169216418408	0.79635172956139	2.10940991283945
C	-3.18640143101830	0.52519680595950	0.26178208951479
C	-4.59759084346702	0.62524879084005	0.37772022958691
C	-5.38756733779829	-0.47914908396119	0.64138611084619
C	-4.77786794393048	-1.74616067725646	0.77255000547774
C	-3.40804219359738	-1.88999143849211	0.64750243122049
C	-2.56675123692641	-0.76914040574042	0.40744480923081
C	-2.48271152769953	1.73212068383387	-0.08417990459546
N	-1.19636253746009	1.87435431080294	-0.15224005223640
O	-1.28209620768951	-0.99421951995769	0.31530583087486
N	1.47167017071154	1.92561019656050	0.11287293789342
C	2.72296642540656	1.73057767177736	-0.13264508486315
C	3.40610951675921	0.45340858452619	-0.08379272632383
O	1.58252102040293	-0.94267258387839	0.71161746239872
C	2.81225742786023	-0.80020917135088	0.37147050731652
C	4.98183097003093	-1.90968245358857	0.09477903009787
C	3.65433481953657	-1.95958068492470	0.46414849655691
C	-0.52288245173182	3.09248642944240	-0.65051555477648
C	0.81360381189913	3.24463515711411	0.11583755036682
H	-3.11132173802102	2.59463891421229	-0.33220000115495
C	-1.34005180131197	4.37999905840733	-0.58033642163754
H	1.93019819142108	4.20240973231550	-1.46321429711724
H	2.55924268645653	4.51987676012645	0.17103628021794
C	4.76579358927045	0.45806475917747	-0.42853150772045
H	3.18952716208088	-2.88069774846666	0.82025506650277
C	5.54767461443458	-0.69796444121189	-0.35885042134286
H	-5.05670797783261	1.60915996499253	0.25239687421793
H	5.59955979938854	-2.80832904260494	0.15676491243596
H	-2.92745837647860	-2.86555689731731	0.74432221984186
H	5.22120155029678	1.39656500865583	-0.75354113814196
H	-6.46995138151851	-0.37501721475170	0.73679128482810
H	6.60072101960089	-0.65818651356409	-0.64419162123568
H	3.35271800649674	2.59107151624114	-0.38479296190966
H	-5.39389917317937	-2.62665884070991	0.97321932739225
H	-0.26401144846816	2.88627152194158	-1.70499910393029
C	-0.51374029922363	5.56425797793922	-1.09786714538678
H	-2.25603952424343	4.28007215737915	-1.18122315159766
H	-1.64482160484877	4.55459804153120	0.46612955043983
C	0.81415844626184	5.70274655019561	-0.35063740894327
H	-0.31474438177038	5.42185192674150	-2.17457245447988
H	-1.10486573666053	6.48836483180503	-1.00281580043078
C	1.63687043989268	4.41034755885916	-0.41968011647276
H	1.40420188848922	6.53402067515880	-0.76680271476020

H	0.61734061614009	5.94868359856413	0.70763147193197
H	0.55959513741512	3.44281614500710	1.17315904369916
N	0.43122566107206	0.05598547183936	-1.63650104708770
C	0.64010124497461	-0.16564104192657	-2.74891443379720
C	0.90641651427579	-0.44897560472611	-4.14130739144082
H	0.11138711219075	-1.09884793652649	-4.53860983964672
H	0.93120463434752	0.49470441445666	-4.70765447313694
H	1.87829185074769	-0.95936715852010	-4.22699242771599
N	-0.29770526180894	0.98148248208114	3.22941600790227
H	0.31339126798356	1.42125822540960	3.94838315401919

[1][INT6][0]

Ru	0.13440022174727	0.43494541834683	0.25552060428595
N	-0.04696386307649	0.78522166732028	2.12128932841644
C	-3.20372864476761	0.55871271593400	0.26581305766902
C	-4.61117223307390	0.70618195290450	0.39101361718523
C	-5.44673145069899	-0.34544131107657	0.72575775099741
C	-4.87345730581378	-1.61754385214822	0.93679097466185
C	-3.50830885853533	-1.80778788793977	0.81487455282699
C	-2.61097733909808	-0.74476828288751	0.48181892912638
C	-2.48178794121747	1.75396295236049	-0.11863617257957
N	-1.19842554637353	1.88001362041988	-0.19907511343713
O	-1.34815302521006	-1.02646562954031	0.38788408689700
N	1.47739246455625	1.93036717019726	0.07182994209713
C	2.75726187590710	1.77124186505075	0.00130262515600
C	3.47470068451546	0.51816708771707	0.10918140264140
O	1.61313677607879	-0.99340761871919	0.58377246148248
C	2.87708964067115	-0.76901012205129	0.39912609506792
C	5.13924502237912	-1.75251221847611	0.32134431804714
C	3.77234997413387	-1.88075789617654	0.49496829397710
C	-0.52535384376295	3.09096794219536	-0.70642381725025
C	0.81290418073163	3.24646556706847	0.05258511025430
H	-3.11247512470142	2.61699680997529	-0.36566707083083
C	-1.33685642681605	4.38476698485943	-0.65975195762916
H	1.92996762552281	4.17746800733920	-1.54257101489431
H	2.55460299500331	4.53864539967873	0.07942283742074
C	4.88299976403232	0.60437839246148	-0.05696478827146
H	3.32510741519774	-2.85391041933922	0.71377442041073
C	5.71685210014037	-0.49628597471142	0.04013120920703
H	-5.03586765991152	1.69898357635122	0.21291215427088
H	5.77631929567015	-2.63830933976687	0.40477611132860
H	-3.06322506838783	-2.79288593655504	0.97741427341890
H	5.31024214361734	1.58978996977050	-0.26760722623067
H	-6.52425740584153	-0.19511334784521	0.82002662293240
H	6.79582517832846	-0.39420453900237	-0.09396155320508
H	3.39039841794267	2.65546155746962	-0.14316164031657
H	-5.51210022576406	-2.46608871617789	1.20033904869014
H	-0.26594950096219	2.87606806437698	-1.76035931792578
C	-0.51551632213252	5.55790315187122	-1.20732379044504
H	-2.26057963808136	4.27577781015646	-1.24788965310567
H	-1.63529500970329	4.58494155356470	0.38449428922354
C	0.81594350779769	5.70786276551560	-0.46907612333203
H	-0.31977335042413	5.39063771539276	-2.28156028246529
H	-1.10283434200846	6.48714590553643	-1.13287708796453
C	1.63106873271421	4.40997232857154	-0.50519076306396
H	1.40759812122388	6.52848274800556	-0.90571325916773

H	0.62130349552506	5.98189410028460	0.58323041429371
H	0.55520173506781	3.47425228925053	1.10443489900373
N	0.34053596127423	0.04538720326738	-1.71857343180451
C	0.49124210533136	-0.16695455904431	-2.84451683736432
C	0.68578001059008	-0.43111840213310	-4.25831092375411
H	0.36327461933528	-1.45682731221329	-4.49406442880410
H	0.09441777624157	0.28074642634195	-4.85432333791634
H	1.75111184850175	-0.31889171146610	-4.51218912399761
N	-0.16152356341628	1.00095435771404	3.21249928476597

^{2}[INT6]^{1}

Ru	0.09080770703425	0.43033771904014	0.25038273205497
N	-0.35710377258918	0.77195589099759	2.14703630132417
C	-3.21191589500887	0.60299698918776	0.34935708253241
C	-4.59823988163420	0.74584161311974	0.61854447583839
C	-5.38863068398152	-0.33492978749572	0.96660994233541
C	-4.80640945269903	-1.61877770603982	1.02706405505675
C	-3.46253174714330	-1.80312433007157	0.74774272186174
C	-2.62020856595517	-0.70785698999664	0.41942640020245
C	-2.50506753226763	1.78874952671945	-0.06762950927201
N	-1.22518131020722	1.88291715652217	-0.24475267994481
O	-1.36080550468571	-0.95597100542953	0.15626658686037
N	1.41525722812814	1.95127222794446	0.11789855529332
C	2.68508332790157	1.78125319064838	-0.07038309458103
C	3.38271252480602	0.52002975462699	-0.01857216306997
O	1.54579397648009	-0.82424732570731	0.82553038587120
C	2.79389836226113	-0.71065563433571	0.44056089888632
C	4.95954105568525	-1.80800556803209	0.15361950879789
C	3.62684377961187	-1.85695866698166	0.52872947339170
C	-0.54781823949522	3.09389215965867	-0.74199366830908
C	0.74677939044162	3.26464148951981	0.09007304710502
H	-3.12735697464303	2.66949822910363	-0.26314662068187
C	-1.37671331303288	4.37494996641446	-0.74332492872139
H	1.93489907924345	4.20713097414128	-1.44460958978191
H	2.47247270667952	4.56715350505555	0.21150645890253
C	4.75709571919946	0.53472209708130	-0.36984514581288
H	3.17368939822514	-2.78231248016992	0.89052219193050
C	5.53862872058140	-0.60558110076932	-0.30307860504295
H	-5.03662447621172	1.74480682795430	0.54599175277201
H	5.56870846955348	-2.71365754714690	0.21772743734812
H	-3.00648518279979	-2.79466304640855	0.78608930519275
H	5.19450048773113	1.48046394254635	-0.70117978082256
H	-6.45030847069940	-0.19867468963303	1.18145199193702
H	6.59165944798875	-0.57291253335175	-0.58951417456809
H	3.30791121862993	2.66001496526588	-0.27232284606984
H	-5.42213533927991	-2.48194003622780	1.29410197401122
H	-0.23499558919147	2.86617166299703	-1.77728509529336
C	-0.53438153116088	5.55282856811039	-1.24800360514087
H	-2.26350461220602	4.25024769509851	-1.38320099132277
H	-1.73282488978864	4.57757164595618	0.28174340919347
C	0.74872887330794	5.71976126654886	-0.43128070904395
H	-0.27452553834070	5.38332989098980	-2.30789117347729
H	-1.13580380437315	6.47475670785834	-1.21180081698979
C	1.58294153721420	4.43319251844252	-0.42302287001813
H	1.35517087891446	6.54659886907239	-0.83326926113362

H	0.48988379824601	5.99019171787555	0.60763204292882
H	0.43626414369582	3.47526436661016	1.12973872831771
N	0.54145365984002	0.04202662526094	-1.66602352221951
C	0.85140615827313	-0.17650131521870	-2.75551697778586
C	1.25100002706158	-0.44843783335097	-4.12083788793789
H	0.59258362734084	-1.21749974484517	-4.55273996673728
H	1.17803712353817	0.47489284565640	-4.71566459421336
H	2.29126186874463	-0.80900142697002	-4.12959260250120
N	-0.62848198896441	0.95872616215705	3.20367542054710

[1][INT4']^{0}

Ru	0.13323324013501	0.46470050363802	0.18888912585387
N	-0.04962489071535	0.74431388727463	2.10052968420745
C	-3.18374189355121	0.59296388704991	0.33159507146728
C	-4.58344910992592	0.74072686842026	0.52318176249841
C	-5.40907211619422	-0.31593646818525	0.86884707063812
C	-4.83453684473462	-1.59526098248579	1.02209871929717
C	-3.47659246967053	-1.78629484911632	0.83451274648376
C	-2.58915401803531	-0.71867172029684	0.49154073756790
C	-2.47626578960537	1.79480054867105	-0.06691145369822
N	-1.19516776170800	1.91065538221132	-0.20674504787990
O	-1.33028317523289	-1.00139431441521	0.34224541074304
N	1.47057983964570	1.95497818750146	0.01771006142208
C	2.75355006815900	1.79509570590473	-0.01431291854370
C	3.46701083477778	0.54313761219915	0.14337785024039
O	1.59239996521128	-0.96063469563990	0.59226141763825
C	2.86516262831231	-0.73776620236022	0.45047405020707
C	5.13005057215114	-1.71783440361110	0.47841842351859
C	3.75792203143518	-1.84266102166832	0.61173322152890
C	-0.53928348845020	3.12108848300720	-0.73519402236072
C	0.81797796080219	3.27652205082959	-0.01368012500149
H	-3.11358630264871	2.66384964346333	-0.27231225151931
C	-1.34384364638268	4.41852472501458	-0.67369614042103
H	1.89551213885290	4.17538194505236	-1.65293054847887
H	2.57014425454420	4.55827324836490	-0.05648737904484
C	4.87985573036280	0.62587595155938	0.01800445934414
H	3.30421482521870	-2.80932624817956	0.84594489947443
C	5.71234709383483	-0.46940519656552	0.17469423613664
H	-5.01196491832661	1.73861717817299	0.38707993342422
H	5.76571756892765	-2.59875853199159	0.60992929232740
H	-3.02915967367941	-2.77676998201982	0.95269796007529
H	5.31294403711368	1.60528275808248	-0.20854001258652
H	-6.48069327786358	-0.16303815956159	1.01388141526022
H	6.79475539273187	-0.36757648846063	0.07036179102705
H	3.39232656261683	2.67574773212123	-0.15617654531314
H	-5.46514910410722	-2.44787917751224	1.29180180458167
H	-0.30954600552158	2.90183931555897	-1.79494862037383
C	-0.53343342105367	5.58075772370015	-1.26035129083658
H	-2.28703972809580	4.30891435447216	-1.23014382463525
H	-1.60698645849552	4.63137632004276	0.37752450182471
C	0.82328894359277	5.73016076622751	-0.56897587635731
H	-0.37470107309635	5.40041688101866	-2.33861765563735
H	-1.11130984939369	6.51525942739872	-1.17711745835003
C	1.62885016499521	4.42609693300528	-0.61095528789700
H	1.40550981877715	6.54066752273433	-1.03634891183316

H	0.66528222252113	6.02093618654265	0.48509996989745
H	0.58807540176829	3.52685590701084	1.04060255835643
N	0.34616983169646	0.08184910816709	-1.82302763598401
C	0.50948284472739	-0.12099140757065	-2.95072000512566
C	0.71783026787133	-0.37598236052837	-4.36565670265444
H	-0.06585851898902	-1.05118734005954	-4.74228656096322
H	0.67924178997189	0.57042853441640	-4.92647508738862
H	1.70150066689757	-0.84635954672532	-4.51810740403247
N	0.13542191441250	-0.08226779116377	3.03087699472365
H	-0.36595952087971	1.65674856753953	2.46703858329589
H	0.47032444429245	-0.95155695825615	2.57773501385518

^{2}[INT4']$^{+}$

Ru	0.06975386597484	0.43679785426544	0.16720758139252
N	-0.33635464091453	0.78141706228261	2.09631036248487
C	-3.21387029921710	0.63649292738373	0.38380896396653
C	-4.58437270077027	0.79266667373977	0.72071483622673
C	-5.37203134827560	-0.28278869173683	1.08977945774411
C	-4.80425762267510	-1.57461960582802	1.10472681778334
C	-3.47737090317376	-1.77119531409764	0.75989193904824
C	-2.63581476169333	-0.68252119021501	0.40736196360521
C	-2.51076274693865	1.82147068451918	-0.04471385490441
N	-1.23596722875894	1.90041793655013	-0.27155009980778
O	-1.39213998482388	-0.93898634276996	0.08620662002576
N	1.40671362489608	1.95047160043179	0.05329385706678
C	2.68146788643240	1.78213755106475	-0.08633913125801
C	3.37894173597124	0.51977997833022	-0.00095484762967
O	1.51321573084243	-0.81310240626410	0.80083031215111
C	2.77946840199613	-0.70344676357169	0.45578758969324
C	4.95607956274155	-1.79865782714492	0.25656368122517
C	3.60974822061506	-1.84466488077920	0.58659388960975
C	-0.56182269954902	3.11352780560075	-0.77168127625161
C	0.74680421541409	3.26826200845619	0.03925534863571
H	-3.12996723898335	2.71224704830767	-0.20004130407855
C	-1.38117123296470	4.40051165510769	-0.74743082873420
H	1.92458445669847	4.21747247241078	-1.49965397816210
H	2.48066147128577	4.56090483791138	0.15359638434022
C	4.76335454087108	0.53349027982876	-0.30889646683863
H	3.14719222287511	-2.76592301260702	0.94730542725158
C	5.54664152524292	-0.60317876127776	-0.19922203085223
H	-5.01183121302568	1.79848506257122	0.68513476811384
H	5.56431373599384	-2.70189915716044	0.35541253025011
H	-3.03303059295044	-2.76888434034406	0.76357805978134
H	5.20873631415606	1.47582781926917	-0.63985717268410
H	-6.42040842552431	-0.13624255533557	1.35706524431381
H	6.60860296180637	-0.57094493043124	-0.45094394442027
H	3.31219014544531	2.66051916165664	-0.26512154767883
H	-5.41759164173510	-2.43376576036184	1.38974677569795
H	-0.26977401751919	2.89149967730394	-1.81402165195459
C	-0.53911984607707	5.57710542210710	-1.25592762149858
H	-2.28055136741045	4.28754441622063	-1.37176489545111
H	-1.71719501680315	4.59753884623047	0.28556163575864
C	0.75784303546651	5.72870757609356	-0.45809502052875
H	-0.29663129561832	5.41479707490406	-2.32102284254876
H	-1.13293774800626	6.50314387447899	-1.20242626501475
C	1.58407205061636	4.43700916053162	-0.47273397906940
H	1.36295977352609	6.55585295523017	-0.86160329654649

H	0.51630397099644	5.99111743580430	0.58711028653275
H	0.45256257938993	3.47353933621924	1.08532281377158
N	0.54608662248998	0.03536258786475	-1.78764534858985
C	0.87734692151367	-0.17825914710229	-2.87299463981148
C	1.30485075041921	-0.44440249928949	-4.23253390908560
H	0.70662128395525	-1.26570942620374	-4.65558032433113
H	1.17081381799865	0.45973459613822	-4.84584242554306
H	2.36804147416215	-0.73028815363515	-4.23089455689770
N	0.13460667553966	0.22049419349038	3.09414503993553
H	-1.02511119246778	1.51259817176350	2.34581614104165
H	0.81978619054333	-0.48417497791303	2.75133493272355

[1][INT4']$^{2+}$

Ru	0.07143596406574	0.45355014417933	0.17312400439022
N	-0.34214851529127	0.80114913400211	2.13271308177939
C	-3.19377935729358	0.66412059454333	0.46384499244344
C	-4.56056097830311	0.78858644270542	0.81280930802691
C	-5.33343214906766	-0.32473699580159	1.09036804663147
C	-4.77287164721810	-1.62059179642077	0.98036025010418
C	-3.45564957065488	-1.79135010976232	0.60490672820253
C	-2.61947408216511	-0.66286779639516	0.37969777027110
C	-2.49556417951166	1.84972872376567	0.06033119770244
N	-1.22565736514440	1.90594197989243	-0.23448601984717
O	-1.37572144699737	-0.87435177982435	0.06456336586067
N	1.39856421112550	1.94645970084855	0.02540158397753
C	2.66314751293884	1.76643686621375	-0.21514070917855
C	3.35072346552893	0.50921627596016	-0.11353395651285
O	1.49673729740084	-0.74132150289672	0.80482021311039
C	2.75812667780997	-0.67277265258742	0.46677793616791
C	4.91403227204229	-1.79148538980569	0.36812590939289
C	3.58146252944118	-1.79764490242707	0.73487469069034
C	-0.57121933734722	3.12065722227783	-0.75564517376852
C	0.75793396474671	3.27284413261494	0.02820816184934
H	-3.10148066305709	2.75575386478588	-0.04326041452674
C	-1.38769597152792	4.40750967272765	-0.71329466391161
H	1.90323107678454	4.20963921595944	-1.54275414616956
H	2.50070300747697	4.55272404925599	0.09881923628756
C	4.72674911314956	0.48423050269531	-0.44121199620352
H	3.11727181571678	-2.66832326256510	1.20113148006808
C	5.49447248612431	-0.64880664209215	-0.22911892659162
H	-4.99805721455865	1.78868392464588	0.85570754617381
H	5.52734123178875	-2.67762612284859	0.54808137156830
H	-3.00655796631065	-2.78152051608905	0.51104363420219
H	5.17935737009395	1.38660667724805	-0.85927508504600
H	-6.38066711858547	-0.20714411410587	1.37533767998160
H	6.55170891584887	-0.65312736858675	-0.50125365919814
H	3.28254969568067	2.63477000974984	-0.46266307809776
H	-5.39243199215570	-2.49577134601095	1.18996156807312
H	-0.30328039120420	2.89103964047044	-1.80206679585016
C	-0.54865771227793	5.57733809172515	-1.24392796195860
H	-2.29908591431898	4.29589578743124	-1.32012629556903
H	-1.69865226907070	4.60869202650912	0.32634357964408
C	0.76631053529621	5.72572568152202	-0.47570168358397
H	-0.33218433168903	5.41060666771472	-2.31367144558064
H	-1.13735750866488	6.50538718406109	-1.17848759050607
C	1.59037215027448	4.43227919385262	-0.50805940433810
H	1.36508891635575	6.54870206056531	-0.89575493778463

H	0.55113387698486	5.99072871623384	0.57432333613699
H	0.49008856509410	3.47879647567445	1.07998115014271
N	0.55534407339315	0.03986082390638	-1.77174769480204
C	0.90105890115331	-0.17084055435416	-2.85256338558836
C	1.34206925319653	-0.43018160744138	-4.20633289847694
H	0.76786967271252	-1.26959566644160	-4.62725131135734
H	1.18638496022188	0.46975649212607	-4.82087580631254
H	2.41293493641135	-0.68621814467041	-4.19401043917215
N	0.09308809787807	0.21196252142311	3.12272512663063
H	-0.99984956568512	1.55997909480478	2.38605692084299
H	0.75502470136407	-0.52379132096504	2.80174560957976

[1][INT2b]$^{2+}$

Ru	0.01408634239659	-0.30608867429875	0.72085531174269
N	-0.49013228446775	-0.63629723214028	2.19336241986061
C	-3.05442925254293	-0.06268947240338	-0.51504672991988
C	-4.43730244471065	0.02766480794289	-0.83063066956562
C	-5.16335863644769	-1.08356239417995	-1.21586666393900
C	-4.50488852140212	-2.32560660417348	-1.32372429502277
C	-3.15162537096100	-2.44633117663410	-1.04278714039432
C	-2.39239048577025	-1.32979710626436	-0.61978701495782
C	-2.37438855659180	1.15970111795622	-0.20331299452667
N	-1.15799256630415	1.28138289243973	0.23285888404679
O	-1.11078698276816	-1.52408756209660	-0.37756526512168
N	1.34531647144563	1.17510150478034	1.11820782543127
C	2.62971306411471	1.01798690252528	1.14138274866573
C	3.34595764830831	-0.21568618800179	0.99046794306796
O	1.44730598521234	-1.65978014269194	0.46057742411839
C	2.73226739825661	-1.47676926537827	0.69339556424784
C	4.93247303686712	-2.52577895837057	0.84308370774341
C	3.56530497146847	-2.61830752813736	0.62472356960332
C	-0.46462437881954	2.58937880956437	0.32001824920625
C	0.66227987746435	2.46056864232782	1.35314067958840
H	-2.94514668209287	2.07951324054082	-0.37772299882048
C	-1.35103090812074	3.79042425408275	0.64999676329547
H	2.02174068409548	3.82946222757046	0.38200626108541
H	2.33393751892104	3.60858788795925	2.11657354704515
C	4.75215290154448	-0.15469491867210	1.18995324649401
H	3.09304923020868	-3.57653178292633	0.40098241877225
C	5.54205400864646	-1.28747369254902	1.12766830058606
H	-4.92108043176239	1.00520732731200	-0.75986289704252
H	5.54229492243215	-3.43160153276259	0.79161350974509
H	-2.63799857519451	-3.40558063939534	-1.13137104444050
H	5.19711104634961	0.81762199213641	1.41675033619662
H	-6.22769420994140	-0.99988200615418	-1.44296217819187
H	6.61843080772047	-1.22483845044261	1.29641655438813
H	3.24845345620071	1.90558310357799	1.31387164809622
H	-5.06527950631080	-3.21058235248553	-1.63688728924528
H	0.00593525709154	2.74739430481556	-0.66640559866599
C	-0.49951536174850	5.06615184620906	0.71646315337703
H	-2.13433255595033	3.91733853282592	-0.11155323706155
H	-1.85186897866836	3.60871239193394	1.61679078476814
C	0.66920630570363	4.92806967425102	1.69450081289626
H	-0.10722964154314	5.28644838812928	-0.29190389350143
H	-1.14041637896169	5.91538163927637	1.00074815338872
C	1.53782224297799	3.70808731632879	1.36659075403353
H	1.29215566906214	5.83571122340843	1.68333096177732

H	0.28284369127802	4.82186209850892	2.72307719862493
H	0.18684458362684	2.36014425610255	2.34366372767748
N	0.86412647209718	0.27991780446730	-1.59239114838324
C	1.25156445005402	0.53444531063638	-2.65109350907562
C	1.73913238117466	0.85705514784462	-3.98025237740916
H	2.13523547112342	-0.05091090492205	-4.45966536984366
H	0.91513636780614	1.26034333383631	-4.58825554655538
H	2.53954393782640	1.60894349330317	-3.90648476295027
Ru	0.21458469163470	1.31569666732247	5.46801232063902
N	-0.68417989795517	1.24693198732383	4.15690399260201
C	-2.03932256445997	0.00607726843217	7.53508944965779
C	-3.09680781406597	-0.59657791958107	8.26985043289248
C	-4.08530975252731	0.16222123371218	8.86730927981349
C	-4.02481022513875	1.56695593048556	8.76113846912558
C	-2.99810440591260	2.18967621096045	8.06646030300525
C	-1.98648055398634	1.43462511709607	7.42671425181957
C	-1.02941170606602	-0.86849428107145	7.01723383601802
N	-0.04496192808985	-0.54812226430196	6.23404188791509
O	-1.04040063074540	2.10208284312178	6.79714102064285
N	1.91232674785142	0.46328684513579	4.75452462548976
C	2.99457277461217	1.11067564882918	4.46461294147379
C	3.17872680453425	2.53349240133380	4.50235815848760
O	0.98131332582559	3.14758798648894	5.37834080409185
C	2.17839819095037	3.46801684475944	4.92739568395764
C	3.72617917239795	5.28558558584360	4.41444821569277
C	2.49328498716690	4.84616362248247	4.87476051080053
C	1.06493448209939	-1.48502766427339	5.92756223004557
C	1.75565305502518	-0.99749931251646	4.64692404990481
H	-1.09404972872230	-1.91329270136233	7.34347608686256
C	0.66617278536895	-2.95448571945880	5.78695513075696
H	3.73927874513469	-1.67267874599396	5.17730111891192
H	3.48768803390520	-1.44762465150598	3.43129306677416
C	4.43602111785365	3.01865066539953	4.05023855663301
H	1.72862828869858	5.55289490762537	5.20190924481507
C	4.71359767209940	4.37186266183106	3.99513785574402
H	-3.11153315404988	-1.68641819559815	8.35290320296373
H	3.92977502193876	6.35911642918787	4.38010941622150
H	-2.94503684404950	3.27739840156392	7.98937135604089
H	5.18297746245404	2.28868961895395	3.72783084034543
H	-4.89545744243056	-0.31659253420729	9.42025218250790
H	5.68075318004378	4.72669517566141	3.63499940691247
H	3.86438247456515	0.52883520378465	4.13988776513399
H	-4.79529410379829	2.18000569410509	9.23651175822753
H	1.78818978196512	-1.38063944033548	6.75591760737874
C	1.89949235140226	-3.80249962169688	5.44521770232888

H	0.21764879622336	-3.32603930355303	6.71995235552864
H	-0.09512921940575	-3.04176538920177	4.99260716621714
C	2.63440860066401	-3.28265189303689	4.20678229494410
H	2.58892239270626	-3.79366531337827	6.30771989745541
H	1.58918888985035	-4.84860167437722	5.29662140288258
C	3.01417049869990	-1.80461104823856	4.35566842913884
H	3.54121241505651	-3.87802077579507	4.01890989283544
H	1.99093514536515	-3.39393174490435	3.31664467590645
H	1.04686334134608	-1.14973199166190	3.81497724269268
N	1.72973214427407	1.36623997835833	7.50009345133277
C	2.48746089479324	1.43051270933086	8.37046273692004
C	3.44424570225481	1.51213720213975	9.45963366326696
H	4.13182130834312	2.35322676393309	9.28324573567422
H	2.91205567911807	1.66906876506597	10.41007553293386
H	4.02018952478621	0.57590892826746	9.51500092469833

^{1}[TS1b]$^{2+}$

Ru	0.09906310394405	-0.17945170548351	1.30642778924682
N	-0.28765982888707	-0.35493320217707	2.90269250180919
C	-3.13025586871171	-0.04583959236383	0.63132604421215
C	-4.54772998056247	0.02662892373767	0.56484783290262
C	-5.32348397311174	-1.10713686386447	0.41170477057050
C	-4.68707290546978	-2.35977482596046	0.28832067748632
C	-3.30535433623774	-2.46724721252622	0.32793700806859
C	-2.48769290572321	-1.32605170345494	0.51960094982800
C	-2.42022274556266	1.19872779458276	0.69352469186320
N	-1.14170210146841	1.34414739586441	0.86578498454042
O	-1.18709284702802	-1.51509366363736	0.56674262547462
N	1.43763228532967	1.31618647540260	1.51086760640620
C	2.72555421410347	1.15631237286327	1.51180947844728
C	3.43921899581669	-0.08451408936295	1.45854824221438
O	1.53871349206771	-1.59687433586744	1.22078850071744
C	2.82584683246656	-1.37957926040362	1.32624235820416
C	5.05305200948820	-2.38685814822757	1.41612282613394
C	3.67923909118371	-2.51385379758713	1.29872313890467
C	-0.44230418848375	2.64136432029795	0.72196497374553
C	0.77411672402347	2.62623947484968	1.66043525474004
H	-3.02601023278822	2.10152917845065	0.55484559931329
C	-1.29096198768795	3.88859347967736	0.95777039568208
H	2.05347380877883	3.84721205737900	0.42337409883339
H	2.49865495486263	3.85250791491197	2.14341315383634
C	4.85630134609155	0.00466243848865	1.56537727269115
H	3.20941182632678	-3.49333908595630	1.19304187007429
C	5.65853637956713	-1.11880005317802	1.55470311608628
H	-5.01763413288641	1.01091461036884	0.63695860928030
H	5.67509413257036	-3.28577248245751	1.40218032668827
H	-2.80874655279690	-3.43440027905476	0.22896734557883
H	5.30076589593359	0.99757361676429	1.67125362159219
H	-6.41187030008328	-1.03359527763858	0.37401678669327
H	6.74221780847779	-1.02956031582903	1.64866482125520
H	3.34906331925736	2.05386046431335	1.57996969992540
H	-5.28899371302057	-3.26264712785957	0.15530887881053
H	-0.05770276753479	2.65871285120196	-0.31349318384884
C	-0.43102966815405	5.14821134502722	0.78496062792970
H	-2.13325759035145	3.92207793430324	0.25122977650762
H	-1.71103587150723	3.84760775747299	1.97777068490276
C	0.80851139341960	5.12105974664846	1.68109322610511
H	-0.11788405925606	5.22676841465454	-0.27090706969138
H	-1.04215063916235	6.03801030573322	1.00281197842825
C	1.64637615390493	3.85889709814166	1.44931034197901
H	1.43096001230973	6.01247082854311	1.50676741624677

H	0.49931786819912	5.15102433881165	2.73983427427395
H	0.38577470074594	2.64747616934401	2.69410770818542
N	0.76075758200001	0.03746930519055	-0.94421750798085
C	1.25590730100494	0.22904249452148	-1.97014972549859
C	1.88821592811866	0.47579746174810	-3.25146827991136
H	1.15214120816167	0.89769737220907	-3.95240547094782
H	2.71588436527670	1.18896134666239	-3.11587733364189
H	2.28153112073441	-0.46875633965376	-3.65691225873437
Ru	0.26584586568923	1.08202894249929	5.52563639094685
N	-0.52187507746254	0.89406167762827	4.08404494962061
C	-2.22674565153969	-0.27312361186573	7.24956923009797
C	-3.35056760648461	-0.90604440755527	7.84720645509280
C	-4.44516550784398	-0.18081712907026	8.27905603731567
C	-4.43133673153175	1.22353719929174	8.14706865091104
C	-3.34541884273533	1.87783413884781	7.58686493323389
C	-2.22396730172547	1.15625690226645	7.10695843687215
C	-1.10734831200286	-1.10970723665354	6.92936536980462
N	-0.02017485677962	-0.75070686091745	6.31518683585132
O	-1.24097267548661	1.85029124012140	6.58045932806557
N	2.03658309598719	0.28272583812853	4.98813979441183
C	3.10777986898316	0.96723368472744	4.72920484753821
C	3.22559454282561	2.39538075592468	4.68498365384905
O	0.92431194019015	2.96698300305692	5.26174066785331
C	2.15245057331614	3.31258736201498	4.95743303115841
C	3.69796578709814	5.16446501945281	4.56141257733832
C	2.43755795214711	4.70089951198362	4.90039666852007
C	1.17695245156116	-1.62126776692563	6.22548281749456
C	1.98476116106375	-1.19104716090086	4.99165726277826
H	-1.17807808066676	-2.14859770321471	7.27214685167058
C	0.90786833174685	-3.12481621953962	6.19104900698220
H	3.92766343136401	-1.64653123260413	5.81386142109940
H	3.88579367390538	-1.61658060967581	4.03853167085019
C	4.50855771802656	2.91060713208463	4.34724900431669
H	1.62252320281246	5.39152383829139	5.12335481186646
C	4.75000594322762	4.26842357299011	4.27427713498989
H	-3.32972631367967	-1.99265207294523	7.96398184048276
H	3.87671936057826	6.24209992526882	4.51823896619784
H	-3.32658249535704	2.96508125396825	7.49003885776804
H	5.30917042424597	2.19709589860691	4.13662805370635
H	-5.30248836155825	-0.68517424032142	8.72845046974031
H	5.73957674195204	4.64327075791791	4.00707489014122
H	4.02723452542672	0.41160942751933	4.51637106481088
H	-5.28563362569168	1.80943730674372	8.49657882205044
H	1.78653368808540	-1.38404194060746	7.11617727731493
C	2.23373717242772	-3.89228931278635	6.08986299490763

H	0.37302071144620	-3.44274049438452	7.09774780647429
H	0.26238519935828	-3.35126811983705	5.32498278415628
C	3.07657043030546	-3.43150017846409	4.89855304206804
H	2.80596970518652	-3.73968036590837	7.02171292283335
H	2.02422439911926	-4.97097774039115	6.01812128307675
C	3.32202156592107	-1.91893625833603	4.93195847537430
H	4.04291911355787	-3.95931811459830	4.88380154351730
H	2.56065812801278	-3.68821930688187	3.95691970195017
H	1.39483290146408	-1.47010551290223	4.10073983239766
N	1.45938334417972	1.42638119263343	7.54407829563826
C	2.12672701460029	1.61878092487815	8.46693406142061
C	2.96645876193332	1.86418341177670	9.62392771153604
H	3.02801397349797	0.95092529593113	10.23507090439421
H	3.97550768755582	2.14998643894485	9.28959433511068
H	2.53587639405660	2.67937601816533	10.22517316623874

^{1}[INT3b]$^{2+}$

Ru	0.06825332617631	0.22197019148722	1.15777889857261
N	-0.29669750517590	0.34169655060700	3.03105513747245
C	-3.22513955314304	0.37967266272988	0.85968025462276
C	-4.63763727964941	0.48813538895211	0.90378452581121
C	-5.45594196674903	-0.62765523697327	0.94951174186891
C	-4.87275009907634	-1.91425807826175	0.92522482759959
C	-3.50047292524888	-2.06569565086193	0.86335566506892
C	-2.62837443581992	-0.93784523825172	0.84792890287801
C	-2.48590128136824	1.61110666445464	0.74033526825078
N	-1.19466920473709	1.71929347876975	0.75315773279395
O	-1.34796936090344	-1.16617433109165	0.81106790036918
N	1.40495587700308	1.69060498383491	1.39428123514438
C	2.68883353290245	1.52228736955624	1.48311714210162
C	3.37634555652579	0.25952248754617	1.51764799606581
O	1.44816387027152	-1.22438812839165	1.57720708330903
C	2.73104961714872	-1.03517291732790	1.57598478625203
C	4.94559329844308	-2.08301832576068	1.68156827063788
C	3.56939499856954	-2.18799722098556	1.66119183407995
C	-0.47414201957349	2.98680960837599	0.52770186277731
C	0.75189054318382	3.01016098868544	1.47264137055154
H	-3.08920495523732	2.51636865919381	0.60940614229229
C	-1.29486230388265	4.26668816813801	0.66512548726851
H	2.04755399987916	4.13028160981121	0.16181482374363
H	2.48369826575332	4.24688334247806	1.88149597249636
C	4.79446788120434	0.32032698959656	1.55368155409300
H	3.07391398409380	-3.15953187662845	1.71088401921492
C	5.57545518864916	-0.81764675981792	1.63032066223524
H	-5.07869528549648	1.48834512958213	0.89887952016586
H	5.55337337562336	-2.98935360033161	1.74584332329906
H	-3.03794285171983	-3.05459544308553	0.84204584806479
H	5.26682985695017	1.30535019514867	1.52370655814851
H	-6.54100021625556	-0.51487621986023	0.99301830370099
H	6.66414662336925	-0.74143089290702	1.65361144634920
H	3.32714986748374	2.40879883682702	1.55714881408841
H	-5.51224700661061	-2.80038797621903	0.95287759271701
H	-0.07879832086757	2.92535133078409	-0.50274132056384
C	-0.41164502206369	5.49499572090554	0.41103735907106
H	-2.13125255426190	4.26065033247427	-0.05032711118651
H	-1.72672142434147	4.31137410290163	1.68011270092427
C	0.82022754034866	5.50817844663845	1.31831201444042
H	-0.08678019017165	5.49050532740846	-0.64434764605090
H	-1.00682728236664	6.41010006590035	0.55702719271695
C	1.63683694724577	4.21834579099671	1.18262738345040
H	1.45935473139791	6.37437071600565	1.08536494237562

H	0.50314909678003	5.62070888775723	2.37019898452572
H	0.36689364244914	3.09223790456433	2.50654057745715
N	0.67157331397343	0.10585161756797	-0.77158234492579
C	1.07756514705153	0.04540150807271	-1.85018100596824
C	1.58807533041768	-0.02950205208481	-3.20344599027742
H	1.21735855546579	-0.94660542718640	-3.68639758033276
H	1.25034820240460	0.84949118328329	-3.77355729785085
H	2.68861673727873	-0.04851749911797	-3.17593415637884
Ru	0.55381034538912	0.81145223177841	5.87835218125339
N	-0.18336061861314	0.48773609698833	4.14145149844853
C	-2.20992253535775	-0.68372841375324	6.91104670442051
C	-3.42506873030280	-1.36455797445762	7.17230613371675
C	-4.60971071779877	-0.68494707298286	7.40021967330622
C	-4.60804423331329	0.72771543812834	7.40108659833876
C	-3.44140584486581	1.43159600759619	7.17023990595882
C	-2.21247474633581	0.76257872459031	6.89773977521006
C	-1.02880490258324	-1.49784455729997	6.76503589000499
N	0.15054779414538	-1.06348225649357	6.44990640149942
O	-1.16473168942194	1.49848184056781	6.66512067142506
N	2.26657888242618	0.01934684861612	5.22209228517606
C	3.30179879865547	0.70378660156649	4.83917497489380
C	3.37509226482471	2.13506449001553	4.71577598680199
O	1.06571388858718	2.68728568668366	5.24810777622055
C	2.26300923705049	3.04375277922957	4.90321737754226
C	3.73853669256152	4.90523354463294	4.29453440494238
C	2.49718094856448	4.43339273323116	4.67015879643267
C	1.35696561080815	-1.90955307130936	6.41461189315830
C	2.22141304500545	-1.45429299926884	5.21425145191854
H	-1.16052133961720	-2.56782199910981	6.96182930158707
C	1.12728943840676	-3.41794569677603	6.38086334129959
H	4.12990210538429	-1.88125436828439	6.12446484435645
H	4.16446612621507	-1.87061053742029	4.34760531568413
C	4.63194406341867	2.66511873114590	4.32016058836369
H	1.64955911208628	5.10860359239104	4.80293634442085
C	4.82564163361724	4.01805971258405	4.11446923434416
H	-3.41064291715301	-2.45741883401191	7.19326810866404
H	3.87960071773337	5.97650220817507	4.12877012963661
H	-3.42628535861431	2.52346521111627	7.16940433560099
H	5.45978115510196	1.96725788329821	4.17253844761828
H	-5.53391991278114	-1.23483067259397	7.58831087458383
H	5.80374505780591	4.39773610718576	3.81331097211856
H	4.20991194552098	0.15978802154544	4.55949262026515
H	-5.53789437150288	1.27121233138886	7.58820316952221
H	1.92355087445861	-1.65416536562281	7.32894778342739
C	2.47045137392344	-4.15856379015159	6.34797860263007

H	0.55299873779939	-3.73509093259950	7.26448787329607
H	0.53008469889472	-3.67248373699590	5.48798774249235
C	3.35217693064596	-3.68831545581679	5.18918665084269
H	2.99927368665567	-3.98530774688770	7.30186587534201
H	2.28889391910560	-5.24260814839464	6.27808440971625
C	3.56827129030261	-2.17130382094503	5.21937828058758
H	4.32761192902264	-4.19908053684875	5.21665512295629
H	2.87765137181028	-3.96098801454021	4.23016552082769
H	1.67252695597102	-1.73118133516753	4.29430578741716
N	1.51191051729813	1.22534038370332	7.61309919254858
C	2.09135700866252	1.48958498759189	8.57546269662803
C	2.81834485956097	1.82451978976546	9.78246886358543
H	3.86131672437192	1.48542663313536	9.68744087564557
H	2.79770136910401	2.91483528234257	9.93343530389435
H	2.34794094404067	1.32588007284723	10.64397220389280

^{3}[INT2b]$^{2+}$

Ru	0.22513178968256	-0.21275944346247	0.77651628242639
N	-0.32216116771464	-0.51317513704006	2.32948656043470
C	-3.04968365622781	-0.00073225056843	0.17965791055913
C	-4.43994489004339	0.11264762735937	0.12402031502310
C	-5.27060745368676	-1.00262042229593	-0.07377549813413
C	-4.71128894887252	-2.28473812841777	-0.26445460346766
C	-3.34383206528488	-2.44704008910180	-0.24052998404994
C	-2.45794552871079	-1.33357119425674	0.01071998259456
C	-2.30617722807885	1.24403554097973	0.28571149870080
N	-1.03230353641196	1.35569084209965	0.43148819140427
O	-1.21185932703411	-1.58102914997894	0.05220472415411
N	1.53633646456573	1.25106080408682	1.17518446637177
C	2.82428301958448	1.10863420210415	1.19303469704306
C	3.55168560468702	-0.12330806024292	1.05102910440840
O	1.66010038943669	-1.58813317075585	0.59475726148314
C	2.94611505932873	-1.39532764128167	0.77490400733804
C	5.16446233739697	-2.41970398688996	0.86630236738692
C	3.79521592690579	-2.52783521992971	0.68268178112890
C	-0.31343058299686	2.64617883542724	0.39364853980767
C	0.86987562843597	2.55515036752145	1.37737506601610
H	-2.91458743799791	2.15217406752044	0.20457365814286
C	-1.15628021101480	3.88992085365280	0.66018281327141
H	2.21341804454136	3.84385815578160	0.27949057383159
H	2.56782856204940	3.73895428030435	2.01714174048609
C	4.96008721378898	-0.04877958638702	1.22018982225207
H	3.32779613430882	-3.49147845298301	0.47124384481072
C	5.76300576740220	-1.17204036361015	1.13937673212487
H	-4.89190977847162	1.10198266308685	0.22699219342127
H	5.78579046520180	-3.31661140106959	0.79788947513040
H	-2.87398505637701	-3.42207774819229	-0.38182863834753
H	5.40070875138632	0.92873798897654	1.43359332477883
H	-6.35394785552512	-0.86834736752984	-0.09646957221633
H	6.84233047295460	-1.09460824880956	1.28243254830493
H	3.43926340387020	2.00215806545573	1.33959989565079
H	-5.36369773383201	-3.14383091519068	-0.43399420800197
H	0.11203665893745	2.70788914499117	-0.62413860769516
C	-0.27738762622190	5.14575649489074	0.59562429779348
H	-1.96611490517281	3.97106289924233	-0.08015794670311
H	-1.62200474013480	3.80051731039288	1.65711716081220
C	0.91578452161286	5.05115431978733	1.54745663023609
H	0.08716134550185	5.27712357813835	-0.43844209058520
H	-0.88907761290375	6.03048315475276	0.83191607106078
C	1.75384282651731	3.79546738247459	1.28191247816472
H	1.55440749071244	5.94352291747975	1.45679573013827

H	0.55548190692671	5.02397216361025	2.59018920441582
H	0.43737654196251	2.51390246816322	2.39060735801264
N	0.72717635388193	0.16667130824911	-1.50588611970324
C	1.02512566678480	0.42474677106591	-2.59226957840614
C	1.39850947245623	0.75565034103260	-3.95454101820648
H	1.74485158135034	-0.15032169679320	-4.47453169223514
H	0.52739761176230	1.17112552427419	-4.48370922850257
H	2.20762894219671	1.50187234875295	-3.94210035827970
Ru	0.19888831791491	1.26157737668213	5.29202453530083
N	-0.62944353111911	1.22700501674708	3.91343896322085
C	-2.28273729372418	-0.12777760089600	7.00470525774632
C	-3.42654457278562	-0.76570125837265	7.55926289200989
C	-4.49379323729597	-0.04032666569320	8.05331155874246
C	-4.43036773709626	1.36895002908427	8.02870499696192
C	-3.32413022852860	2.02569391456602	7.51358086435669
C	-2.22579688325307	1.30759199789990	6.97564205646877
C	-1.20278987493735	-0.97942603375770	6.59531775916240
N	-0.12700915893331	-0.62323561999395	5.96242959372812
O	-1.21272335544631	2.00397798402954	6.51463372899362
N	1.93936892208282	0.43454324396734	4.65821749171045
C	3.02521360505389	1.09418746636135	4.41475139180704
C	3.19159148955561	2.52099178534910	4.45766217094585
O	0.95812233667949	3.11482803403570	5.24628847256361
C	2.16958743937694	3.44616899192570	4.85471661726264
C	3.73352246049322	5.27742259819266	4.42828697908417
C	2.48754044813909	4.82721262281371	4.83749204025921
C	1.02273916080702	-1.53592690685024	5.75285191779472
C	1.81938597703414	-1.02736252350636	4.54246950131972
H	-1.30287638567711	-2.03559143501133	6.87272842991874
C	0.66817395601072	-3.01121697246652	5.56428206872883
H	3.75996914961126	-1.67047084901988	5.24300567547000
H	3.66211569442721	-1.44171600238207	3.48345774647244
C	4.46271645229064	3.01738576659177	4.05981918686443
H	1.70952365837568	5.52737501806034	5.14737906325829
C	4.73807168432211	4.37191464580634	4.03068545775177
H	-3.44483167675305	-1.85841603207928	7.58651723649775
H	3.93501364074914	6.35192173273408	4.41620988849592
H	-3.26939330665807	3.11617892704647	7.49943201095316
H	5.22488836564794	2.29426072695416	3.75785489123322
H	-5.36797704909850	-0.54780240813407	8.46531421529498
H	5.71653788311524	4.73369529071345	3.70982790655161
H	3.91810488901037	0.52417694206026	4.13594896417453
H	-5.26372186965392	1.95619436388860	8.42414333492054
H	1.66512531530331	-1.42662565876780	6.64514686195622
C	1.94159073783379	-3.83618365666231	5.33250079660014

H	0.13918981204966	-3.39974420443255	6.44661153023778
H	-0.01197143217913	-3.10329829380726	4.69954371741642
C	2.77592204750630	-3.29485146554021	4.16919276625056
H	2.54959529343891	-3.82311554487580	6.25424028030831
H	1.66446244559305	-4.88635820759881	5.14992577485627
C	3.11258626121524	-1.81170743822034	4.35997140275986
H	3.70569639820676	-3.87401511210407	4.05846119071102
H	2.21484487101258	-3.40977933699779	3.22559629019775
H	1.18550091625085	-1.19171405655374	3.65381327027623
N	1.54970842134964	1.31601196371585	7.40390713114191
C	2.32304080685417	1.38718160935380	8.26001150963081
C	3.30160181858713	1.47671799230678	9.32892838699136
H	3.99308291816960	2.30839132501474	9.12524897407996
H	2.79042273335823	1.65330821048749	10.28734225482139
H	3.87013265229538	0.53612496046703	9.38701383114835

^{3}[TS2b]$^{2+}$

Ru	0.28026986118674	-0.37701745909642	1.13278394635206
N	-0.27718429474823	-0.66190436796848	2.68632936975906
C	-2.99612157592511	-0.22374003336453	0.52331703719794
C	-4.38826864733772	-0.13234568467811	0.46597174902101
C	-5.20076503282783	-1.26257011567057	0.28210682508007
C	-4.62062423097541	-2.53773884525419	0.10667850884807
C	-3.25076129076154	-2.67844862426561	0.13277522588561
C	-2.38295830526074	-1.54855206114004	0.37164704792281
C	-2.27120597305530	1.03274145986531	0.60754811831110
N	-0.99851443667307	1.16417463795538	0.74870119607505
O	-1.13260759681413	-1.77473233892936	0.41728443703600
N	1.56358500507745	1.11563637330562	1.51991468454643
C	2.85336113473675	0.99382613579393	1.55462554981451
C	3.60112456132895	-0.22927445562940	1.44729573610954
O	1.73974405372256	-1.73371276127933	0.99399626537480
C	3.01917959052607	-1.51676727405620	1.19121367608902
C	5.25171931928338	-2.50370608248331	1.34018086663068
C	3.88710607271653	-2.63774663244658	1.14013968719808
C	-0.29814434923406	2.46322092202158	0.68511597925372
C	0.87640331067628	2.41526885771261	1.68253688741203
H	-2.89209316845452	1.93084441550461	0.51166530650084
C	-1.16148060969336	3.70189289247407	0.90541066434832
H	2.20927837618150	3.69027917064348	0.55702579289967
H	2.55180008829070	3.64618930500569	2.29911452535084
C	5.00563742462685	-0.12878965457472	1.63472418664300
H	3.43788647926391	-3.61351751599302	0.94537373296855
C	5.82707915255112	-1.24054066306343	1.59109960435898
H	-4.85535027691407	0.85129780789201	0.55542302341556
H	5.88766663279926	-3.39214296235738	1.30196705118093
H	-2.76594421223768	-3.64776202309174	0.00331877365009
H	5.42765763669737	0.86019910473247	1.83254112739144
H	-6.28612813874280	-1.14613108382055	0.25745132459702
H	6.90293636161524	-1.14267991889463	1.74721653644163
H	3.45373566476010	1.89930833661730	1.68849443802729
H	-5.25929609915248	-3.40898949393770	-0.05287126870321
H	0.13861278709853	2.50429656253025	-0.32878020896545
C	-0.29973625456730	4.96737358751821	0.80976385141067
H	-1.96471426163382	3.74737592603934	0.15480375349748
H	-1.63587868385456	3.63771156591543	1.90019122037133
C	0.88399189454215	4.91712376182321	1.77596639498570
H	0.07324170173358	5.07190018567131	-0.22427775514222
H	-0.92516684005705	5.85048242967363	1.01386377469139
C	1.74256849796557	3.66602386282679	1.55700755432605
H	1.51160425956970	5.81519672674308	1.66683349260887

H	0.51162332046637	4.91544078786121	2.81438584295711
H	0.43583093729995	2.39582787905434	2.69285342351125
N	0.82533224802720	-0.01814113529781	-1.14514321279778
C	1.18671136293682	0.25578231544416	-2.20822117073627
C	1.64163597871984	0.60981931573471	-3.53948555003979
H	0.96454757372979	0.17592370366347	-4.29081542286639
H	1.64914817410312	1.70563913960239	-3.64391735770667
H	2.65933554871366	0.22123431273915	-3.69665367405876
Ru	0.11916933200249	1.27783098471869	5.60932856765473
N	-0.70385301864018	1.12516376780637	4.23796508136026
C	-2.26266753768952	-0.08235923564082	7.49143404735939
C	-3.35260266395950	-0.72078403249570	8.14554092249933
C	-4.42761603051302	-0.00249251302201	8.63269603300628
C	-4.42733816002022	1.40192908901038	8.49674099419915
C	-3.37523645897545	2.06022912610176	7.88022784853750
C	-2.27063153608072	1.34694906313668	7.34907915567088
C	-1.16792776961074	-0.92007753612188	7.09571471194080
N	-0.12949125943148	-0.57269546720075	6.39831669774313
O	-1.31021660808968	2.04614344213477	6.79031388982104
N	1.89429320979321	0.48287452492850	5.03221232398448
C	2.96730444788506	1.16267956636340	4.78844725390497
C	3.09586058466799	2.59376241651209	4.79082105900516
O	0.81693572237181	3.14921246621794	5.47128326771386
C	2.03603946743977	3.50157976574850	5.12123438899671
C	3.56803362692258	5.36063216632829	4.70341787613183
C	2.31884712015380	4.88918120592603	5.07821628583620
C	1.04055072287578	-1.46587968786322	6.21004952142061
C	1.81579886626185	-0.98441120028799	4.97522816734727
H	-1.21763307211464	-1.95782251854559	7.44661974751012
C	0.71724933105280	-2.95453346084236	6.07280103139007
H	3.77356492532961	-1.55368339805130	5.69205538043353
H	3.66658676444879	-1.38775252168089	3.92591014105463
C	4.36720653895072	3.11158936040404	4.42308847107517
H	1.51221320060899	5.57665099486990	5.33936909371666
C	4.60895729378400	4.47182031110697	4.36698006567605
H	-3.32152384262120	-1.80786754469515	8.25680088819415
H	3.74321424459726	6.43929337393735	4.67073454795965
H	-3.36896631088848	3.14751821285059	7.78005103339480
H	5.15753872895582	2.40060868066739	4.16825286660594
H	-5.25954396374222	-0.51082895660691	9.12356131693214
H	5.58914106065751	4.85079605272414	4.07228386166529
H	3.88103804593559	0.60710920297673	4.55049790869311
H	-5.26699002841344	1.98440892977493	8.88582597345125
H	1.68749150060544	-1.31277221048691	7.09249763195040
C	2.00813265307628	-3.75823347711104	5.86198534264806

H	0.20077564675433	-3.32563708660193	6.96987883549249
H	0.03530746578303	-3.08961469951051	5.21534885163493
C	2.82700558813363	-3.23769940284342	4.67804037946567
H	2.61912497145834	-3.70009772714863	6.77992066623548
H	1.75410465539224	-4.81986553788219	5.71571898963109
C	3.12831807921747	-1.74097930196637	4.81611314242152
H	3.77041391262688	-3.79776121761052	4.58588399510456
H	2.26838789566839	-3.39952742400416	3.73974665138059
H	1.18355469927483	-1.20038062313301	4.09642280801466
N	1.46789855107666	1.47843741348144	7.72775024141183
C	2.22817166248008	1.55016207534142	8.59545153104761
C	3.19157463892401	1.63708163346387	9.67839429899140
H	3.76261635800705	0.69804175474746	9.73802034607626
H	3.88284691555967	2.47250405059127	9.49006481415824
H	2.66671910002985	1.80507685441022	10.63093848341096

^{3}[INT3b]$^{2+}$

Ru	-0.00297188597193	0.19279820088628	1.14479005460290
N	-0.36211168215924	0.34757102283642	3.05095036548424
C	-3.19555681507849	0.72961630148089	1.51027101602845
C	-4.49366039188846	0.98592288804330	2.02090863104603
C	-5.34957895368413	-0.04185872034654	2.37685673544724
C	-4.93767612573532	-1.37726059335134	2.18829868028372
C	-3.69140386287947	-1.66817909605614	1.65668261300505
C	-2.77719538434580	-0.63597812438210	1.32629293722057
C	-2.39440256181644	1.87398851806229	1.14602942729374
N	-1.13178979217964	1.83714808507980	0.85800226582506
O	-1.60279995880178	-0.97336136276439	0.84170074832810
N	1.47414158431953	1.57632071630204	1.34734656590361
C	2.73762472764488	1.30671671569427	1.41497303319384
C	3.33037147748182	-0.00672120404995	1.44851597281663
O	1.27613535694302	-1.28135759012622	1.63563150657743
C	2.58217897416750	-1.22889470238744	1.55246174778431
C	4.68906444223561	-2.46663077629285	1.62578141472428
C	3.30423238504772	-2.44807620138763	1.63914708273740
C	-0.31380448266715	3.01238960306918	0.52201500602990
C	0.93747849646493	2.94917175703498	1.43068121556834
H	-2.91256197839920	2.83932256618154	1.12201724106676
C	-1.00376202506142	4.36757402240899	0.62934134036697
H	2.32385840297566	3.89929347468865	0.08132482972644
H	2.76886983667946	4.04588978234894	1.79703090627391
C	4.74807396756932	-0.06491619046282	1.46327859428787
H	2.72680207097904	-3.37095124689133	1.72115146457594
C	5.42774472281895	-1.26792898850307	1.54441425441157
H	-4.80457932336499	2.02671668036004	2.14341347108535
H	5.21257944867508	-3.42400674118234	1.69289772322482
H	-3.36696603873504	-2.69948475846875	1.50335984984597
H	5.30181242815103	0.87633772748800	1.41329767038945
H	-6.33437899747271	0.17746706068521	2.79345991915100
H	6.51915008127488	-1.28930553439378	1.55049628622615
H	3.44263880769706	2.14217224992784	1.48321013017819
H	-5.60762936876779	-2.19565640174985	2.46432357286957
H	0.02860243408722	2.86083183488876	-0.51716086976390
C	-0.01530121331256	5.48783872244982	0.28348979513266
H	-1.87234707424670	4.40264357651728	-0.04637492645200
H	-1.37756701571936	4.50630586147993	1.65865258796412
C	1.22741809081866	5.42978866095261	1.17464011654583
H	0.28440588964079	5.39296750867268	-0.77510235414307
H	-0.51604020410848	6.46329262156450	0.38616729357809
C	1.92227864830283	4.06593756840246	1.09597776294432
H	1.94166107117686	6.21973407217996	0.89397593707907

H	0.93746880265193	5.62132289097349	2.22203631694063
H	0.58610563497603	3.08181557668333	2.47032400409048
N	0.53691488180303	0.01763158215349	-0.78171250544526
C	0.90632460118922	-0.10076753138924	-1.86843594468930
C	1.37362115370541	-0.24930432460701	-3.23136041376237
H	0.97780312998132	-1.18457238366273	-3.65602528483068
H	1.02698872407425	0.60385667087963	-3.83436390803458
H	2.47415676734055	-0.28040334135658	-3.23784221269268
Ru	0.44860668267385	0.81541431674600	5.93661439935339
N	-0.26502299623294	0.48620603449839	4.15236194617754
C	-2.22227509311330	-1.01487534649632	6.26433297494028
C	-3.38189854723900	-1.79821055816686	6.03480624747372
C	-4.64212164724619	-1.22894018004577	5.97624646588208
C	-4.78210807661450	0.15703015277913	6.19526613768166
C	-3.67701809282039	0.95014368867721	6.46032512324014
C	-2.36921006380167	0.40243558374799	6.47550380697031
C	-0.96041840095719	-1.71218740315454	6.33865713552217
N	0.20335152618130	-1.14272724968312	6.33483014455745
O	-1.35716039813714	1.20862166008030	6.70831626337824
N	2.25694402004409	0.16918539111081	5.26605677664291
C	3.22756654032283	0.93898898660937	4.89303989181697
C	3.19162195265121	2.37623023852897	4.79486267002449
O	0.83603426099561	2.68536946834858	5.29423474215328
C	2.01155624352453	3.17410601879338	4.99082419172141
C	3.30938623742975	5.16276302838085	4.40461040485642
C	2.11294471549519	4.57516475795040	4.77983299307517
C	1.48763524066978	-1.85707509737910	6.38297271199667
C	2.33687173626148	-1.30576884370355	5.21355413252631
H	-1.01376120430335	-2.80529598167897	6.39919520544350
C	1.41797125494194	-3.37931932886054	6.34463354962733
H	4.27615396852380	-1.53054621529169	6.12510899421130
H	4.30684772005079	-1.54962463081851	4.34645674010593
C	4.39371589086955	3.01360007383037	4.38993785288596
H	1.20980560511186	5.17203265384607	4.92188629276718
C	4.46650430821547	4.38226635461486	4.20052807017596
H	-3.25868042495935	-2.87449378520796	5.88850695853441
H	3.34978070706515	6.24449761683880	4.25219931416910
H	-3.77767386104870	2.02336747816124	6.63430859911786
H	5.27538837910363	2.38950935519346	4.22075502907979
H	-5.51846811655198	-1.84579032175894	5.76938460584426
H	5.40279734039330	4.85042471260670	3.89120361163863
H	4.16905637592818	0.47188829138730	4.58395475436383
H	-5.77393810063164	0.61474582819445	6.15586374084098
H	1.97792493930449	-1.54258919971553	7.32099683765324
C	2.83425408650826	-3.96638154127032	6.37577438487068

H	0.83183311164719	-3.75022191415523	7.19966712108795
H	0.90117373163145	-3.69907324772543	5.42329270201550
C	3.68780852567519	-3.42497629624421	5.22688155799385
H	3.30982805626412	-3.71455279507447	7.34016367995342
H	2.77781994021724	-5.06510043917768	6.32628705001372
C	3.74577759821134	-1.89302340357514	5.22747437678185
H	4.71142914465190	-3.82772048776887	5.28355103948752
H	3.26630374652916	-3.76563315651936	4.26600216602602
H	1.83025099401140	-1.60877178801767	4.27917753304302
N	1.33696279107866	1.21192197577927	7.69369249138627
C	1.89130851333904	1.45612581015613	8.67580216242546
C	2.58976915737946	1.76135306366108	9.90737770322066
H	3.63818274871976	1.43616206101663	9.82300745560933
H	2.55544210453511	2.84640046239440	10.09002661842948
H	2.10788322302213	1.23276544099261	10.74418297316040

^{1}[INT2]$^{+}$

Ru	0.19097992878444	0.81533142966043	0.85450085639271
N	0.10937784912900	1.27534699936845	2.37448578454699
C	-3.09958220727636	0.67872678386305	0.33909373528396
C	-4.51977499887125	0.70717037979332	0.29272810177153
C	-5.27545805016527	-0.39531076300417	0.64612735519002
C	-4.61591596338392	-1.57704908976416	1.04054388971006
C	-3.23048700173517	-1.64634721312145	1.07792715412875
C	-2.43612983406290	-0.52714537850579	0.73516610614214
C	-2.40759140847799	1.85210550302633	-0.10945749216209
N	-1.13308752735627	2.08197255110134	-0.03271355585750
O	-1.12580855336049	-0.67878229174003	0.77189151133092
N	1.51621566138261	2.19629928155720	0.17909407792757
C	2.76364940576659	1.95819804689935	-0.07179108651708
C	3.45047588029638	0.70936226175978	0.10238678315549
O	1.54990992088932	-0.63057946413401	0.85688207890166
C	2.82736901204960	-0.49955125187018	0.55471539393581
C	4.98255154972847	-1.63059963363871	0.35782062984584
C	3.63012613947901	-1.65883130541993	0.66810695447375
C	-0.48389259641396	3.22712503866014	-0.71741569117368
C	0.85349465018733	3.50071044000843	-0.01202986158063
H	-3.03119899830008	2.61787028763274	-0.58584578602510
C	-1.32148939297394	4.50152404364178	-0.81293061236985
H	1.91776859592061	4.18983167546073	-1.75900257722748
H	2.59699112789461	4.76865196534614	-0.21955454799402
C	4.83583786915887	0.69951892146888	-0.21302072383018
H	3.14661189065478	-2.57679815559373	1.00789078332996
C	5.60065324894319	-0.44591011412439	-0.08801910091803
H	-5.00795876192114	1.63038094456533	-0.02999910974658
H	5.57182426084031	-2.54595857475424	0.45916476285000
H	-2.71676950336909	-2.55977362930040	1.38271673890272
H	5.29161407690543	1.63044926863382	-0.56035311994444
H	-6.36574661438757	-0.35445044790388	0.61409985272051
H	6.66439726067485	-0.43333473640230	-0.33212733690977
H	3.37099365789906	2.78435876354944	-0.45901031971849
H	-5.20136221174133	-2.45749474853786	1.31877449021769
H	-0.24842053260349	2.87453283172480	-1.73721935370388
C	-0.52407618717774	5.59677412522170	-1.53362394175783
H	-2.25416259087879	4.30923777701838	-1.36318964554487
H	-1.59494373909311	4.82823713145800	0.20516325763609
C	0.82946050407834	5.85094320876700	-0.86657351801644
H	-0.36374449863895	5.29176601560123	-2.58281918548093
H	-1.11911715347047	6.52302814348369	-1.55932662290073
C	1.65670782928205	4.56365732172087	-0.75376002064491
H	1.40017972303115	6.60395261196767	-1.43211432372009

H	0.66838960890914	6.26438148966225	0.14430661636398
H	0.62501605227834	3.87123561857947	1.00386426883066
N	0.37391462540138	0.21308635508471	-1.62222562017726
C	0.49339431128453	-0.06069670805998	-2.73880519588694
C	0.64830851516492	-0.40453525901858	-4.14134680238911
H	-0.34172168727840	-0.56063018857080	-4.59599282792617
H	1.16494907504228	0.41287400603355	-4.66681966710157
H	1.23963038215676	-1.32826436711216	-4.23235204613138
N	-0.47102538925396	-1.89489354429959	3.52005010867340
H	-0.24072333787705	-2.87674819907201	3.69340798916022
H	-0.56581289294047	-1.81213093168115	2.50395736942848
H	-1.42450555094353	-1.78457058243681	3.87548805046510

^{1}[TS1a]$^{+}$

Ru	0.12804011302711	0.58677518993222	0.61316752930643
N	0.06408885096111	1.00976474928205	2.21965864296627
C	-3.19356192084445	0.58250088610915	0.27426097263940
C	-4.61365073458943	0.65519072201897	0.23699778698540
C	-5.41016031060917	-0.43721936239381	0.52310621329124
C	-4.79186010430139	-1.66507520272415	0.84122588236268
C	-3.41215228196075	-1.77953371882608	0.87212354287068
C	-2.56608768388014	-0.66987764637437	0.60224937408412
C	-2.48417231763466	1.77195276970874	-0.11667570522341
N	-1.20360951105135	1.95970175716653	-0.06675979889779
O	-1.27179467251285	-0.87283981546433	0.64665337995094
N	1.47496386644620	2.03262252518669	0.15534771586003
C	2.73512853933755	1.82652732977157	-0.03835286723445
C	3.42742927992323	0.56854307616372	0.10768519445550
O	1.52896764657072	-0.85351917806949	0.70830934367948
C	2.80847785154162	-0.67924684654990	0.46694964825611
C	5.00525040747728	-1.75573029392165	0.31094898692651
C	3.64228979020933	-1.82571930635641	0.55166859869519
C	-0.53470564106583	3.13645578787599	-0.66912293020227
C	0.81309177235319	3.34562898364661	0.05017571017386
H	-3.10904789119844	2.57934923459154	-0.51684246432410
C	-1.35361705820804	4.42709408181716	-0.68262286831635
H	1.88451792334809	4.15845704984306	-1.63869091394843
H	2.56117823018161	4.62059995879026	-0.06135385533304
C	4.82662258496769	0.59819235133631	-0.13795146766427
H	3.16495947970840	-2.77140912293078	0.81779713287968
C	5.61485965448463	-0.53414565413992	-0.03896572467983
H	-5.07123625056785	1.61195185891124	-0.02934539199387
H	5.61066909565612	-2.66282638865637	0.39183882104274
H	-2.92971009227140	-2.73027271969806	1.10939440519356
H	5.27809476252963	1.55613143271135	-0.41083379490189
H	-6.49841868632423	-0.35497904620472	0.49468937818159
H	6.68863867048102	-0.48289690169120	-0.22927788036254
H	3.36055300673960	2.67417779240028	-0.34247579907469
H	-5.40718865642986	-2.54163898250663	1.06237452098055
H	-0.30165619600147	2.85313155222336	-1.71176746127016
C	-0.54641093904925	5.56347202439161	-1.32259013713794
H	-2.28776240920337	4.28193449836044	-1.24504020673396
H	-1.62803054238686	4.68705462536578	0.35458562332032
C	0.80523431033349	5.75848027064416	-0.63304646334970
H	-0.38177730834121	5.33023947249936	-2.38949240915865
H	-1.13361827766204	6.49466938824643	-1.28751584509852
C	1.62048513976908	4.45956828073403	-0.60976566631773
H	1.38397230126915	6.54729910333119	-1.13907045020964

H	0.64140028773987	6.09932335381772	0.40451919629457
H	0.58400993996800	3.64420442348931	1.09004688715291
N	0.30746759362648	0.13175855433100	-1.72036704876468
C	0.46656598039213	-0.06827739679343	-2.84700978849391
C	0.66930865085807	-0.31428472881241	-4.26282875217150
H	-0.27529898086363	-0.64606914904477	-4.71989400649036
H	1.00585867102733	0.61245770583715	-4.75201060594246
H	1.43335105191250	-1.09509258780525	-4.39654203242554
N	-0.06000241813511	-0.62833955954463	3.30754609889875
H	-0.19235745233516	-0.38540359524673	4.28888500327871
H	0.82157870431975	-1.11991477837259	3.15010364296570
H	-0.83817581973202	-1.17374880840863	2.93243310302902

[1][INT3a]+

Ru	0.11261707749865	0.53402891147495	0.41532009305071
N	0.05390521694793	0.87526842284403	2.18396432598876
C	-3.21547758794651	0.59522078149108	0.28877929289097
C	-4.63383423369980	0.69848349958327	0.32199710942674
C	-5.44268003682298	-0.37735192479912	0.63574635197465
C	-4.84193445817759	-1.62369965437087	0.91413027105187
C	-3.46607154687594	-1.76906481095163	0.87624513180298
C	-2.60661486694419	-0.67701391801388	0.57460042070332
C	-2.50221491244726	1.78434987002804	-0.10686441092811
N	-1.21586267513612	1.93933975897950	-0.12131263775253
O	-1.31413418376154	-0.90905316069063	0.56777278441935
N	1.44925470708127	1.99953275318285	0.10359320818588
C	2.72508595768609	1.81966600457643	-0.00200816898674
C	3.42902939981989	0.56647863318750	0.15483593566667
O	1.53789864322758	-0.89421962490415	0.67443059531061
C	2.82397929352769	-0.69859789225054	0.47849280539893
C	5.04752466736452	-1.73120811582558	0.41120037305679
C	3.67773199428369	-1.82862271842497	0.59223161838348
C	-0.55371745264149	3.13228047073139	-0.69235272643001
C	0.79311842860754	3.31684114902731	0.03505460326158
H	-3.13209354308234	2.61696161370927	-0.44113791736994
C	-1.36897763804348	4.42463389197237	-0.67506515374321
H	1.87926074043814	4.17543448116083	-1.62069063612801
H	2.54110063246125	4.59905006862645	-0.02731598344003
C	4.83754294090983	0.62355095388651	-0.02662004458082
H	3.20806032861430	-2.78520949761919	0.83247612178795
C	5.64427835645412	-0.49332102329464	0.09685253502756
H	-5.08174662737383	1.66865597381803	0.08957223416570
H	5.66811458791086	-2.62597646513026	0.51184945258827
H	-2.99420307264736	-2.73189306247312	1.08519740473023
H	5.28250625640749	1.59264117189206	-0.26962953661077
H	-6.52863526312740	-0.26691467415654	0.66170735393108
H	6.72407890238912	-0.41715035026417	-0.04611065414139
H	3.35964696187318	2.68522188410276	-0.22564598267825
H	-5.46708565796188	-2.48675678768174	1.15932053406151
H	-0.31995232258381	2.87635551426366	-1.74219824152271
C	-0.55715386617717	5.57465746114426	-1.28393851191657
H	-2.30276975521055	4.29571678530164	-1.24222736872287
H	-1.64536684065781	4.66131036617443	0.36730648864449
C	0.79144318274419	5.75004942144980	-0.58319580202088
H	-0.38733662406315	5.36666038558937	-2.35531371205880
H	-1.14209074854319	6.50650114154187	-1.22997447374321
C	1.60520479125033	4.45016294367358	-0.58689064996936
H	1.37369587306904	6.55083729348950	-1.06619518693640

H	0.62235033798022	6.06592541664032	0.46157715747979
H	0.55936286044484	3.58299015522235	1.08303554073829
N	0.30823297930907	0.10724013017888	-1.75000701587409
C	0.46619694366944	-0.12526111896756	-2.87037986324857
C	0.66599399758917	-0.41307388969557	-4.27776508349671
H	-0.28373469352318	-0.74667953175602	-4.72315297998401
H	1.01294681140046	0.49465623885118	-4.79446867434668
H	1.41882467467351	-1.20830515763824	-4.38874777618913
N	0.06175522575430	-0.40081568922232	2.89100537207458
H	0.04201545450453	-0.27706907565287	3.91049966422354
H	0.90574181819495	-0.93833819661388	2.59513370983296
H	-0.74025530583848	-0.98227136583073	2.57250941653241

^{3}[INT2]$^{+}$

Ru	0.10752310863366	0.75898710308443	0.62228105661422
N	-0.25269858575994	1.24165558933790	2.20666254064945
C	-3.17774938734712	0.82082241659579	0.50595514035249
C	-4.57579693021201	0.92561680825156	0.71302061736921
C	-5.33502214967065	-0.16612958971297	1.09953059993801
C	-4.70909355981894	-1.41916656374994	1.25500024254737
C	-3.34908119145829	-1.56773888810733	1.02922578097180
C	-2.54649488976620	-0.45744376283928	0.67332571165693
C	-2.49462280520485	1.98816599019391	0.00025339536112
N	-1.21368432654218	2.11283917753957	-0.12081954543923
O	-1.25922107533016	-0.66779340467627	0.47498819099228
N	1.43536033851230	2.20639523109010	0.12577628944333
C	2.66504367417451	1.96736663065272	-0.20756921802126
C	3.35932853287990	0.71693568999832	-0.04489763361456
O	1.61948215003454	-0.45485170151620	1.16561202004086
C	2.81974065876624	-0.42128660325744	0.66325152896723
C	4.93901989479842	-1.58937111271338	0.34035019408518
C	3.65735184701844	-1.55443804202780	0.85985609719098
C	-0.55180556177073	3.25444425712389	-0.77947263320986
C	0.77424809006195	3.51368532053915	-0.02793075563437
H	-3.13706860418719	2.80900213943413	-0.33912762364336
C	-1.38089207138319	4.53104844178568	-0.89447583845739
H	1.88959025247431	4.27130687586444	-1.71529054934801
H	2.51286070587167	4.79750513527326	-0.13409904025441
C	4.68540541191419	0.65153228672642	-0.53147905103253
H	3.24126134613780	-2.39854889928131	1.41277466315537
C	5.46414077304957	-0.48351064373525	-0.36296451309992
H	-5.04981179740848	1.89839923510445	0.55908105990902
H	5.55449410681186	-2.48090702668784	0.48436019779437
H	-2.85508908213398	-2.53295973119530	1.15070760239846
H	5.09342780115201	1.52422351558632	-1.04763486105324
H	-6.40809958323882	-0.06039358639887	1.26961407598021
H	6.48124972774141	-0.51593813918402	-0.75814182214956
H	3.25306701527227	2.78225965631752	-0.64412333089058
H	-5.30010051304104	-2.28894648305779	1.55334201363686
H	-0.28049105723905	2.90512406443099	-1.79244519551625
C	-0.55642056867781	5.64437098256511	-1.55350718619297
H	-2.28682559685142	4.34126965192444	-1.48958078068052
H	-1.70370799128247	4.84159835342900	0.11430238800692
C	0.75970464162062	5.89077360164483	-0.81297276588554
H	-0.34017938787934	5.36273540483785	-2.59918240915192
H	-1.15549795343595	6.56762374344465	-1.59022945829946
C	1.59177747113662	4.60680210809998	-0.70676464680821
H	1.34914197744518	6.66683368308305	-1.32580294897728

H	0.54625968339408	6.26939226959684	0.20194326574816
H	0.51231972415052	3.83772572094468	0.99530458238775
N	0.61478506710239	0.05105530548134	-1.58828451849799
C	0.95671155506476	-0.26637625096445	-2.64527517317350
C	1.40609601903456	-0.66444313911933	-3.96570681907633
H	0.58450892788108	-1.16041505627318	-4.50433611201499
H	1.72774889171356	0.22515565406046	-4.52841725349093
H	2.25305805272189	-1.36083515888942	-3.86825567187246
N	-0.56240259132603	-2.28727435396027	3.12848172997849
H	-0.40976446943064	-3.29913883735506	3.13841771045822
H	-0.58580015188488	-2.02953657094722	2.13830463702884
H	-1.52700013502834	-2.16695385413935	3.44797903078253

[3][TS1a]+

Ru	-0.00126613495551	0.69138922408943	0.62274294014827
N	-0.28192179612643	1.19251080080610	2.23376304006411
C	-3.28363199151557	0.93002743157740	0.51736019745546
C	-4.67503436386012	1.09494687611028	0.72766915185149
C	-5.48889947368115	0.02657955932902	1.06650903053447
C	-4.92648195321686	-1.26248189370103	1.16056481993129
C	-3.57411227227422	-1.46652724919617	0.93235061383734
C	-2.71428252392113	-0.38125811228589	0.63995338500923
C	-2.54085208050602	2.07914996389826	0.05740757851302
N	-1.25615869704603	2.13992000304184	-0.07265544900324
O	-1.43765459491587	-0.64159904417618	0.44365733909632
N	1.39074229919841	2.11032594097135	0.17435240306748
C	2.61546164343167	1.82918218358856	-0.14025672161975
C	3.25221438764207	0.54420321592277	-0.01368311676939
O	1.45709200874192	-0.57932195024465	1.16667582980771
C	2.66011636664457	-0.58788665530241	0.65281733788733
C	4.72541891080412	-1.83720997909028	0.28171977287127
C	3.44359458831604	-1.76238015972881	0.80115917463082
C	-0.54337659144128	3.26633795607195	-0.70314696063518
C	0.79027349159370	3.44868809362634	0.05586516239543
H	-3.14067192042736	2.94526850106749	-0.24582523695532
C	-1.31530246468403	4.57997728667605	-0.79505362440989
H	1.94609342229603	4.19300499503719	-1.60972416690818
H	2.58253242067776	4.65936293609358	-0.01559192527892
C	4.57711316962718	0.43640598362781	-0.50586053014098
H	2.99174045908034	-2.60892587405615	1.32175188277487
C	5.30513216625798	-0.73450586045736	-0.38039387899296
H	-5.10050885777731	2.09554789114328	0.61455399592643
H	5.29609062575760	-2.76287674509808	0.39360913366329
H	-3.12966905184929	-2.46118417566401	1.00247326266941
H	5.01979920145199	1.30856637060916	-0.99404767705565
H	-6.55595787595919	0.17764331169061	1.24046322268607
H	6.31993544742907	-0.79989770647002	-0.77736956686712
H	3.24829767093277	2.63409911185454	-0.53091855108575
H	-5.56201609128717	-2.11541571752166	1.41272390867461
H	-0.28401918824084	2.92857493427421	-1.72331170932536
C	-0.43993768213176	5.66783200526284	-1.43049518077646
H	-2.22698620379824	4.44114656407563	-1.39544319268612
H	-1.62744428113667	4.88620405217579	0.21842598981100
C	0.88257928302359	5.84164605538720	-0.68052098717039
H	-0.23165017632884	5.39604132978303	-2.48042752317453
H	-0.99688547808835	6.61759476162148	-1.45262559625264
C	1.65719156499823	4.52065911437968	-0.59596347921444
H	1.50753435518008	6.60128396413923	-1.17577996198069

H	0.68089385671215	6.20982856256144	0.34067275803312
H	0.54024514096659	3.76074643926158	1.08594278814050
N	0.49050321447594	0.04164887517798	-1.59761262702759
C	0.82576673837326	-0.24371304812658	-2.66571823124782
C	1.26573074717994	-0.59738933250584	-4.00197036332228
H	0.77445636792576	-1.52894055148043	-4.32121156984992
H	1.00772151837108	0.21085196940189	-4.70299314103631
H	2.35690313049759	-0.74309455648117	-3.99813079784372
N	-1.78256089371563	-0.50819898487828	3.50318955429044
H	-2.77777443428231	-0.71513276561143	3.60719926070388
H	-1.39382175658576	-0.40254189840081	4.44166055425285
H	-1.35254536783456	-1.33962400385820	3.09589767790314

^{3}[INT3a]$^{+}$

Ru	-0.07418782273104	0.38910652012046	0.24192611738706
N	-0.48293405706938	0.73348917837986	2.07962324309929
C	-3.38755030449409	0.59060790615723	0.21500333719146
C	-4.78529084434617	0.74880998769662	0.41188032200260
C	-5.60759725374657	-0.32123314572961	0.71493096819600
C	-5.04327105957311	-1.61218725447281	0.80623756668871
C	-3.68919600808823	-1.81084543686399	0.59921240129627
C	-2.81185727594599	-0.72731933478688	0.31136210143623
C	-2.64583976791004	1.77199180391095	-0.14483498774084
N	-1.35793969637090	1.84984465500105	-0.26463813599580
O	-1.54951675079987	-0.99291140611110	0.11795360250126
N	1.29521076431653	1.86436326852611	0.14020404707793
C	2.56493691453153	1.68034081994771	-0.02713137744266
C	3.23716684182675	0.39993563790426	-0.00092279601964
O	1.36815594662681	-0.93751357705793	0.81235372983686
C	2.62357390941037	-0.83162860158059	0.41553196659043
C	4.76330524615726	-1.96571039966209	0.05898818558905
C	3.42864464474274	-1.99713337455635	0.43802878061009
C	-0.64688142279935	3.07007183836981	-0.69710712634608
C	0.64417434460701	3.18592654762328	0.15234189387735
H	-3.24858576668689	2.66606533209068	-0.34268373699125
C	-1.45187503527401	4.36674247626845	-0.64810350824048
H	1.86274466807813	4.18852826963009	-1.32202990967323
H	2.38709199321642	4.45283912613747	0.35550741989075
C	4.60863901792067	0.39808973502406	-0.36131022328666
H	2.95620131434763	-2.92756192604374	0.76079422395121
C	5.36791019367062	-0.76069848808054	-0.34744307094324
H	-5.20667400934720	1.75397485119242	0.32166380353445
H	5.34924328772922	-2.88874684286116	0.07916647445258
H	-3.24794872725603	-2.80810626447927	0.66326260146599
H	5.06417657962424	1.34647277541962	-0.65985194163920
H	-6.67746519540668	-0.17206161896583	0.87404973020021
H	6.41979567515533	-0.73826844396899	-0.63943327482288
H	3.20821743000885	2.55071505942070	-0.20174185631536
H	-5.68188043900961	-2.46832606466035	1.04126396404276
H	-0.32652506404251	2.88542433733543	-1.73905904436298
C	-0.58749386056814	5.55658089994772	-1.08174675093670
H	-2.33303259005037	4.29037117436909	-1.30309080148703
H	-1.81837171974870	4.52405224030485	0.38149552409520
C	0.68767305647521	5.66358358626178	-0.24287796533079
H	-0.31603314631092	5.43843419281243	-2.14576140519661
H	-1.17623693667875	6.48441862814401	-1.00550715371501
C	1.50214151953788	4.36586934061562	-0.29374047187298
H	1.30917378480432	6.50338096183396	-0.59182930193976

H	0.41897170209130	5.88029249879768	0.80625170973920
H	0.32393848428188	3.35145563069163	1.19700901968037
N	0.45388607209336	-0.04632635263713	-1.79583303752805
C	0.93910363375590	-0.25234264471007	-2.82427326916417
C	1.57080544860705	-0.50963926614739	-4.10402459578315
H	1.24058775782620	-1.48478276278374	-4.49290771076193
H	1.29766710759082	0.28115896687946	-4.81913259455547
H	2.66375693112906	-0.51975525758658	-3.97135483088989
N	0.64792118989126	0.55096520346198	2.95474325692216
H	1.28037041084545	1.36903970133190	2.94764449644977
H	1.21135994272069	-0.26722272655746	2.64068377279558
H	0.34219507143413	0.41021388026226	3.92648533195291

[1][INT1]$^+$+2NH$_3$

Ru	0.21853197875633	0.84543403902908	0.83278529177347
N	0.15035534439353	1.30621560442268	2.35276148665904
C	-3.07700424723627	0.72995813196297	0.36611961830037
C	-4.49732016330028	0.76816857050295	0.34056331350457
C	-5.25655035681980	-0.32265268387652	0.72227977670818
C	-4.60105718507498	-1.50262229411837	1.12807597879401
C	-3.21583046120577	-1.58227732654812	1.14592428375297
C	-2.41817231583946	-0.47656197884073	0.76887620028786
C	-2.38392778683253	1.89683488440418	-0.10167847184658
N	-1.10880396768256	2.12337160594568	-0.03571577274252
O	-1.10881405603795	-0.64124372323968	0.78172265911396
N	1.54756663415175	2.21686268594251	0.14784627277111
C	2.80059053298368	1.97853748395253	-0.07501583399572
C	3.48499912881812	0.73242681973118	0.12661064687999
O	1.55948656251890	-0.61201234944226	0.79574568201750
C	2.84713773083538	-0.47793308748973	0.55302400868003
C	5.01245708647912	-1.60207085152658	0.45378549041112
C	3.64763163972393	-1.63480795501793	0.70372887449265
C	-0.45759075975555	3.25790862415173	-0.73506732861966
C	0.89167441533504	3.52448695220897	-0.04921062102688
H	-3.00940762798241	2.65989078495572	-0.58027442278192
C	-1.28421115228718	4.53948837147784	-0.82820866466168
H	1.94045994271420	4.18581579805965	-1.81562894842334
H	2.64272107322748	4.77666455414760	-0.29144745884205
C	4.88234150553772	0.72624613109749	-0.13018600888904
H	3.15224401641321	-2.55198791080164	1.02788523844101
C	5.64509409652562	-0.41602972656713	0.03206164529887
H	-4.98363855573766	1.69021420205055	0.01177049206344
H	5.60062604104939	-2.51438647511707	0.58474802987578
H	-2.70307944164910	-2.49239854570206	1.46212463915197
H	5.34964098748352	1.65801206341242	-0.45962838551534
H	-6.34682619986715	-0.27320228499148	0.70529218689195
H	6.71860608093225	-0.40002865099106	-0.16442792076167
H	3.41618498706457	2.80267684018959	-0.45364179966465
H	-5.18920000779794	-2.37291228828633	1.43146655638504
H	-0.23899081742766	2.89694426197424	-1.75577587795647
C	-0.48797746266531	5.61999269410919	-1.57190107751206
H	-2.22803004171860	4.35159612085457	-1.36063391376427
H	-1.53698458039946	4.87805585996704	0.19138089027401
C	0.87808334211886	5.86724731596905	-0.92813952408433
H	-0.34707897990744	5.30339942603877	-2.62047343886448
H	-1.07417326646735	6.55189105072523	-1.59735302130316
C	1.69448166106686	4.57330710698994	-0.81172927332635
H	1.44801610093809	6.60771787610602	-1.51081024302347

H	0.73642726401377	6.29414272715522	0.08006468884738
H	0.67989025787390	3.90623402282122	0.96640818853713
N	0.34316445690971	0.24062612066348	-1.65796332740093
C	0.42816332532125	-0.09882786779220	-2.75968390895038
C	0.53868885264991	-0.52575806192977	-4.14371081331591
H	-0.28734729441609	-1.21079941785233	-4.38792420511574
H	0.49183669232389	0.35155132272960	-4.80652125908494
H	1.49764332825457	-1.04463824507213	-4.29390974372811
N	-0.17454776013885	-2.27403143615529	3.25499282646271
H	-0.06711952289993	-3.27949275799544	3.10165359258027
H	-0.38316267770623	-1.87262474386158	2.33592632721066
H	-1.03594876026945	-2.17262332308837	3.79736280967975
N	2.34966128340659	-0.46084404810540	3.86671734971866
H	1.54958575793014	-1.09358136716459	3.70950998281487
H	2.57283584653011	-0.54101770359525	4.86232410310012
H	3.13707749484141	-0.89997294858019	3.38305213372120

[1][TS1]+

Ru	0.12489331470982	0.62866716964885	0.63796972280541
N	0.00584070110200	1.05275124172292	2.19708337661944
C	-3.17621392979972	0.59435489679627	0.31046236665940
C	-4.59531072964291	0.66950940438879	0.35696096849898
C	-5.37139467397553	-0.41617550363753	0.71657218938706
C	-4.73404617275760	-1.63739582497938	1.02073922881103
C	-3.35454272766761	-1.75471139025821	0.96674695997534
C	-2.52955073789816	-0.65178404635114	0.62315753172276
C	-2.48422932817436	1.77245973812360	-0.13833719666876
N	-1.20289858026923	1.96313376249641	-0.12609660243033
O	-1.23377604221628	-0.85262912707111	0.58603334430066
N	1.46416918357827	2.08518656687335	0.15841647676825
C	2.72696828422921	1.88687986541419	-0.02366403291021
C	3.42470362440089	0.63267166448459	0.12471187769207
O	1.51456578738269	-0.80277347221060	0.65086975586572
C	2.80019811593909	-0.62201323029647	0.43812959300312
C	4.99890638589659	-1.69287749376340	0.30825607301202
C	3.62918260106208	-1.77048382734420	0.51126858819403
C	-0.53851634175400	3.13331534827060	-0.74409060917136
C	0.78415460813431	3.38502460855928	0.00883726032488
H	-3.11973139786751	2.57122067776577	-0.53955989582448
C	-1.37883592531365	4.40690929640957	-0.81677571669942
H	1.89298889914819	4.17113276265140	-1.66753389388962
H	2.51265570950163	4.68568613834127	-0.08278635363273
C	4.82876710404090	0.67116319705695	-0.08591698268154
H	3.14630037539392	-2.72330823086032	0.73869757851662
C	5.61587217618601	-0.46301982634411	0.00725099753435
H	-5.06827210538202	1.62135846513458	0.10064090717265
H	5.60256979624080	-2.60172513206708	0.38065018679991
H	-2.85857669393598	-2.70083826049602	1.19395384916798
H	5.28451208333148	1.63546289397949	-0.32693009462837
H	-6.45913231667290	-0.33330215298818	0.75709393158047
H	6.69402105967264	-0.40651045401232	-0.15419220212432
H	3.34893066829986	2.73831582176941	-0.32433778439194
H	-5.33426703049297	-2.50749258431329	1.30093343024684
H	-0.27054914322081	2.82362412991562	-1.77017608395541
C	-0.57060379607408	5.53895125224866	-1.46337515240496
H	-2.29233860573218	4.22931987972699	-1.40337669633440
H	-1.68967497062642	4.69055396870592	0.20384002699771
C	0.75564437213831	5.77744938427659	-0.73864720916193
H	-0.36940755177356	5.27916414419416	-2.51772876894434
H	-1.17460770572530	6.45989857597681	-1.47272646317985
C	1.59241002922920	4.49493561928519	-0.65579412081733
H	1.33600873972263	6.56240435516436	-1.24859136809960

H	0.55443781648258	6.14287938994998	0.28379326930753
H	0.52067896234896	3.70259343770705	1.03459213674714
N	0.37362658798044	0.17778641949260	-1.78532685649808
C	0.60194479972357	-0.05683288996442	-2.89363927299072
C	0.89261408200617	-0.35071973317562	-4.28548526683224
H	-0.04849627014261	-0.45908464823770	-4.84558078622340
H	1.48217263533194	0.47085346278810	-4.72000012812937
H	1.46704407678767	-1.28704614887401	-4.35293729530592
N	-0.36196252152290	-0.76908796439171	3.48505437485150
H	0.63188679237975	-0.97576679734183	3.69114869773452
H	-0.78453237901096	-1.50982473668824	2.92710371209122
H	-0.89763978327576	-0.61692399811800	4.33881204190435
N	2.60659843474745	-1.05546566485306	3.81523698851737
H	3.02394271487833	-1.81005487569005	3.26504295368936
H	3.03813321787025	-1.11869206391684	4.74064331566465
H	2.97287372104705	-0.19449746107670	3.40083312176605

[1][INT3_NH3]⁺

Ru	0.07872262513771	0.45407075579808	0.27382635754742
N	-0.07300091411890	0.80482049758570	2.10261084118127
C	-3.24726857259124	0.55449094828822	0.23345793776868
C	-4.65922554529262	0.68905548024231	0.32438358019086
C	-5.48852834701117	-0.35874817043998	0.68504794957372
C	-4.90719660895808	-1.61454112469194	0.95980403689355
C	-3.53755389556903	-1.79238439220928	0.86926661241347
C	-2.65042326731012	-0.73304155000484	0.51011317510272
C	-2.53036509474592	1.74407305557151	-0.18089979255502
N	-1.24563408286501	1.88369692064463	-0.22478357917255
O	-1.37719733617325	-1.00433679945623	0.44661187511060
N	1.41334388048822	1.93775881217791	0.07736567484667
C	2.69592706852443	1.78017382291326	0.05929028983557
C	3.40434485884044	0.52351708585014	0.22380122730870
O	1.51519254402521	-0.98929048313328	0.57875276467768
C	2.79197901011786	-0.76565669832060	0.45892585914221
C	5.05390759976840	-1.75069532286937	0.56675574037986
C	3.67329391953953	-1.88016106991781	0.61634648459739
C	-0.57422903657155	3.08795597730996	-0.74824117359434
C	0.75426289176165	3.25427811309511	0.02198770560214
H	-3.16490433770499	2.58632857634272	-0.48355829740854
C	-1.38833051915166	4.38110600908668	-0.72351250025506
H	1.88940125762318	4.16269865573661	-1.57389794125353
H	2.49709744803200	4.54648299962748	0.04930227263908
C	4.82011866662623	0.60689067617322	0.17323062223941
H	3.20832658062885	-2.85568818022486	0.78182336076558
C	5.64621209418724	-0.49317434412911	0.34295797732290
H	-5.09218650684798	1.66855008767060	0.09930934411543
H	5.68189288872221	-2.63605264374194	0.70177353331074
H	-3.08314315149948	-2.76434539118564	1.07829459987627
H	5.26426421134051	1.59179059153155	-0.00048414790268
H	-6.56929477365880	-0.21705375352537	0.75248073588638
H	6.73210800191553	-0.38542733159978	0.30443959802288
H	3.33835753699213	2.65812937547414	-0.08031435237965
H	-5.54167731394905	-2.45868589384022	1.24597508409923
H	-0.30772817329664	2.86168583382131	-1.79778642403595
C	-0.56236883447313	5.54738112860738	-1.27900534405716
H	-2.30706553919285	4.26459795474673	-1.31796677064063
H	-1.69542871767564	4.59357945681496	0.31578170611030
C	0.76347960163030	5.70721489398500	-0.53221362417104
H	-0.35781757657585	5.36599906863578	-2.34932373158952
H	-1.14994682408312	6.47771434616281	-1.22172271003316
C	1.57967162853197	4.40914970383087	-0.54283425762928
H	1.35795079974580	6.52251848830480	-0.97498795024926

H	0.55972204307216	5.99549523081620	0.51460245554223
H	0.48483404489025	3.49482302147453	1.06788050806525
N	0.30179513007572	0.04964044988079	-1.81561353995377
C	0.46582410287248	-0.18756840426003	-2.93558523318704
C	0.67310046748441	-0.48718239012967	-4.34150613032235
H	-0.29684957980335	-0.68092528843199	-4.82454241562847
H	1.16040669145081	0.36595877096297	-4.83758438495745
H	1.31045682178291	-1.37904246811797	-4.44141906948927
N	-0.24672626140839	-0.20781739212064	2.88216648591851
H	1.84025326724905	-0.63798960351408	3.29061468867577
H	-0.47918607175295	-1.13217726603851	2.48878836378110
H	-0.45155128565699	-0.04627245507184	3.87150775424730
N	2.84517768994424	-0.64926725823607	3.53498770832783
H	3.34609119941763	-1.26104550454892	2.87193609674250
H	2.96866094341596	-0.99211659112316	4.49463124966446
H	3.21976065210236	0.30364098171696	3.45488111293982

^{1}[TS2]^{0}

Ru	0.18702270911145	0.48885552397924	0.25707574850057
N	0.08054126645237	0.78224185457006	2.09893698087244
C	-3.13889193841680	0.58565606137692	0.33796828981081
C	-4.54583984340268	0.71826524770162	0.48767895151436
C	-5.36091735265719	-0.33638915529529	0.86075289306560
C	-4.76954427445971	-1.59721970795705	1.08727902053997
C	-3.40483463981183	-1.77312190927675	0.93849438451222
C	-2.53117785185758	-0.70659142304515	0.56609716553430
C	-2.43823912292509	1.78149496003121	-0.08553594703197
N	-1.15607049605351	1.91984882598982	-0.18089198437082
O	-1.26185784606731	-0.97576672914195	0.44960950113467
N	1.50989930460219	1.98346320812713	0.03731038585432
C	2.79380328542657	1.83080001699141	-0.00401713383039
C	3.51388750198654	0.58304025783702	0.14879317644646
O	1.64427452769884	-0.94319918571118	0.54164442545204
C	2.91678659040687	-0.70873805659531	0.41486137975792
C	5.18461041677963	-1.67951536311540	0.43460265642537
C	3.81193636116852	-1.81538463536422	0.54679137304283
C	-0.50650667659243	3.12743034526545	-0.72373857989595
C	0.84591087369420	3.29835995858907	0.00167215562078
H	-3.08334871283959	2.62868716260516	-0.34975009318999
C	-1.32326640966230	4.41823205785865	-0.67534979303269
H	1.92273449311168	4.20098787145101	-1.63660016727760
H	2.58583404446319	4.59625106100041	-0.03833931607123
C	4.92780778210888	0.67594305388802	0.04056491360083
H	3.36048201989485	-2.79099101548786	0.74528704484464
C	5.76368243134393	-0.41890876710543	0.17747319025455
H	-4.98670163547247	1.70183248961795	0.29831573072948
H	5.82340301041816	-2.56074088222785	0.54702813409140
H	-2.94279898861581	-2.74848931442080	1.11168870661878
H	5.35798994493502	1.66286901503487	-0.15662842168036
H	-6.43805667668527	-0.19613552483685	0.97403543544917
H	6.84673557241306	-0.30860659460541	0.09004670660432
H	3.42654115890493	2.71383810724949	-0.15703815198195
H	-5.39252726850497	-2.44689341302566	1.38228411899593
H	-0.27352047360417	2.90061518562068	-1.78117377685274
C	-0.52008358795383	5.58410908585641	-1.26435727845991
H	-2.26202084130548	4.29726953391588	-1.23679341764450
H	-1.59427449744993	4.63476448246290	0.37309864042054
C	0.83071453174364	5.74965144605895	-0.56525450364220
H	-0.35294177873150	5.39861380460161	-2.34049299571109
H	-1.10725080415226	6.51357278710588	-1.19035272647507
C	1.64848919219450	4.45289467113144	-0.59682824241426
H	1.40807775356878	6.56346958319129	-1.03289224277750

H	0.66321821689980	6.04371071398617	0.48640550661287
H	0.61067214641192	3.54439233287954	1.05498544520843
N	0.35547527982861	0.09122427652722	-1.82959865818862
C	0.47401135075669	-0.13624491873248	-2.95716756090678
C	0.62292421948154	-0.42271377555998	-4.37316561009597
H	-0.36688325016416	-0.60280324038879	-4.81987325015238
H	1.09770951671663	0.43202947231110	-4.87855795631189
H	1.24896927362721	-1.31798915881104	-4.50780826190296
N	-0.06418367823988	-0.20977338896223	2.98488762383185
H	-0.62693696854094	0.88484207465840	3.10160952411644
H	-0.27288916198349	-1.11710036980529	2.51455686043466

^{2}[TS2']$^{+}$

Ru	0.04906719198845	0.43952811656184	0.17773068622654
N	-0.32993436381249	0.81175660483414	2.04009743784862
C	-3.23492513950989	0.63203901067357	0.38328940320394
C	-4.60577515406394	0.78550552150545	0.71870890083896
C	-5.39425324453315	-0.29225541751835	1.08015021874203
C	-4.82668708895342	-1.58426647321048	1.08711854281201
C	-3.49932807889906	-1.77822479522178	0.74277613899587
C	-2.65657165721419	-0.68703937029707	0.39880243130404
C	-2.53228765972030	1.81766035922830	-0.04229154520674
N	-1.26041937667975	1.89510552446285	-0.27722704392310
O	-1.41283918982177	-0.94274008703791	0.08077844475086
N	1.39199133363695	1.94983076801929	0.05619556520875
C	2.66534014778555	1.78113530721846	-0.08471369251903
C	3.35954499535774	0.51533491854477	-0.00105791080297
O	1.49430735826587	-0.80937836260310	0.82372802966541
C	2.75839824761305	-0.70437876972965	0.46109110422408
C	4.92610551316795	-1.81223278402912	0.23334493807263
C	3.58245411265881	-1.85056137201266	0.57783411398065
C	-0.58273949881548	3.10830809186494	-0.77072245620145
C	0.72614441673275	3.26433028481558	0.04264110093202
H	-3.15253714746621	2.70897276785473	-0.19224658569783
C	-1.40204179395893	4.39564595967966	-0.74595184089665
H	1.90403886251536	4.22033136173838	-1.49228442139312
H	2.45720167918779	4.55918870891712	0.16274116393059
C	4.73997605906948	0.52266617789651	-0.32252340442675
H	3.11848048672530	-2.76983645966547	0.94182420419585
C	5.51850643576332	-0.61923292120816	-0.22406262331217
H	-5.03345635485542	1.79148221594707	0.68894672479510
H	5.52955098421711	-2.71962284664069	0.32296823457657
H	-3.05486479678109	-2.77592200686501	0.74093928917891
H	5.18728726504035	1.46316921792830	-0.65607945278252
H	-6.44294500956624	-0.14728627215222	1.34722496430662
H	6.57799426290995	-0.59182990041166	-0.48655434250298
H	3.29755968716395	2.65755913032555	-0.26891847256862
H	-5.44031209092957	-2.44536673127534	1.36572915280228
H	-0.28746851383544	2.88945506482580	-1.81294402827856
C	-0.56095938859133	5.57460119499842	-1.25002887551264
H	-2.30044324265130	4.28260702494765	-1.37174324266430
H	-1.74037218970095	4.59045393136695	0.28672876234959
C	0.73362587437930	5.72652837748390	-0.44847665327296
H	-0.31525230552644	5.41496229826820	-2.31481422209537
H	-1.15643291504203	6.49959456422723	-1.19618001051401
C	1.56152216269363	4.43612756391573	-0.46520465801672
H	1.33830935885462	6.55600122305235	-0.84788768155420

H	0.48885154760115	5.98518026307895	0.59692878357614
H	0.42908235209215	3.46382802093619	1.08871708177729
N	0.54335212775046	0.02678142443641	-1.80858162783208
C	0.92561134896501	-0.18009566725061	-2.87861891060968
C	1.41564838559299	-0.43849769718751	-4.21844662058484
H	0.61038693857824	-0.87218806746428	-4.83073660153219
H	1.75178599312342	0.50590167236011	-4.67332221047017
H	2.26100704009349	-1.14206526896195	-4.17138263407703
N	0.32443501189560	0.38238418491431	3.05354665127184
H	-0.81283451255967	0.76766515302430	3.20521085367461
H	1.11069353206829	-0.28266073911005	2.85683884600660

^{3}[INT3b]$^{2+}$ + 2NH$_3$

Ru	-0.31602884009319	0.14272264043695	1.35412140764523
N	-0.54229616429252	0.34358222600355	3.27377628972847
C	-3.41523465586011	0.93920834849221	1.90807340915675
C	-4.64317374242466	1.30752900254400	2.51204632002363
C	-5.54504286201719	0.35816436327207	2.96008529368870
C	-5.25524689857557	-1.00822835835419	2.77122840685083
C	-4.08234251200735	-1.40669038891545	2.15004832623436
C	-3.11976809959013	-0.45700664490182	1.72576921820398
C	-2.55733151249634	2.01099564492962	1.46038813125191
N	-1.32313445765803	1.86729487489223	1.09814748389368
O	-2.01478088993886	-0.89577640713877	1.16194837747331
N	1.27243417156582	1.41023260765962	1.45342105341445
C	2.51224531604939	1.04475924256749	1.45228112958284
C	3.00213604437506	-0.31104889214554	1.46240446624777
O	0.87203900888349	-1.43025804268068	1.77237904505827
C	2.17082721552589	-1.47292698642781	1.61370154415274
C	4.18000570312952	-2.86608012573700	1.56008008536573
C	2.80436504897907	-2.74259307482825	1.65645664680023
C	-0.43593635225691	2.96741296926200	0.69132951029327
C	0.84584344472593	2.82110451054915	1.54653966625375
H	-3.00019566385206	3.01165472573319	1.44297507849466
C	-1.01801320366844	4.37281279043297	0.79621779266119
H	2.24588319428222	3.65739069328154	0.13674442717186
H	2.76357891473774	3.78350729679060	1.83410540271429
C	4.40958785148575	-0.47670108583352	1.39319160705133
H	2.16530876912388	-3.61999396398698	1.77180680309146
C	5.00073000827848	-1.72707588480251	1.43320606770118
H	-4.85856807325962	2.37180314036742	2.63632616848950
H	4.63189206516970	-3.86071795735941	1.59358035771071
H	-3.85238851631719	-2.46305962191619	1.99717186174818
H	5.02934226093037	0.41919988203700	1.30402092936518
H	-6.47185765990746	0.66323328926632	3.44916289393932
H	6.08535364463387	-1.83026241669937	1.36949583540601
H	3.28184170304651	1.82390259343103	1.46928568012621
H	-5.96190533071493	-1.76573008009945	3.11959857456450
H	-0.15288923140240	2.76572612488563	-0.35688884951046
C	0.03893674727640	5.40998079091742	0.39940226618871
H	-1.90086967867455	4.45938032623672	0.14362584164469
H	-1.34848705089824	4.54793607118737	1.83331662722920
C	1.30224048102574	5.27070610979899	1.25152882941193
H	0.29699320792261	5.28067040190504	-0.66661983247528
H	-0.38382078798319	6.42168186357157	0.50365290032852
C	1.89523382737809	3.85993357693135	1.16362994259682
H	2.06188695198341	6.00551665920370	0.94162666722092

H	1.05901906028032	5.48992792832664	2.30488514241989
H	0.54935516290110	2.98397545851302	2.59756502048331
N	0.09646624775138	-0.08420718279359	-0.59760344581131
C	0.42180655203475	-0.23982017692391	-1.69461636693609
C	0.89558783372603	-0.47023766224609	-3.03979192006731
H	1.87744612956613	-0.98435342632630	-2.94215072578645
H	0.17960856055103	-1.10306934359426	-3.58581039928496
H	1.01558668296034	0.49107432534557	-3.56227228013333
Ru	0.48047914086420	0.86177284098565	6.08291190142545
N	-0.36895997882863	0.50861479363069	4.36240758699528
C	-2.24774082765476	-0.81164283236399	6.63790978970424
C	-3.45898996404710	-1.53487385133564	6.50106990465874
C	-4.68715637429709	-0.89679130309412	6.49093614191205
C	-4.73774169642530	0.50111550412899	6.66413441739372
C	-3.57780454471758	1.23789020139319	6.84280885975340
C	-2.30559981618245	0.61538005375017	6.81153991150039
C	-1.02181246673864	-1.57444080344512	6.65462992773534
N	0.16700783407322	-1.06984429014786	6.55658020379000
O	-1.23814700884456	1.36750303866059	6.96865858724803
N	2.20827856307495	0.10856302090968	5.31948952305526
C	3.19979292897201	0.82052053457198	4.89305569215431
C	3.23516458257048	2.25767139756811	4.78279682408085
O	0.91625260402199	2.68688488450109	5.35003020618835
C	2.10686414844413	3.11668037481730	5.00760588823398
C	3.48379247111717	5.03160010339584	4.37062430906050
C	2.27073855786234	4.50774348038200	4.78560203859107
C	1.41244235370634	-1.85105060907720	6.52928815473147
C	2.20103421120970	-1.36793234841507	5.28796760925790
H	-1.12742350700542	-2.66078960681302	6.75097141950206
C	1.25822379344417	-3.36736284245466	6.53083438935558
H	4.19132171468407	-1.69679607289376	6.04861390686721
H	4.07768531621321	-1.73804813365933	4.27369939669309
C	4.45447677829601	2.82937301741156	4.33770029629127
H	1.40381989487816	5.15018879971683	4.95162257295685
C	4.59158931153622	4.19171845150646	4.13742359737838
H	-3.40375359248773	-2.62038972764988	6.38577568706222
H	3.57493424079521	6.10904347006259	4.21051961039719
H	-3.60820012360729	2.32034400004549	6.97873181789673
H	5.29757832317098	2.16021322327268	4.14718910880713
H	-5.60648117093795	-1.46914028170482	6.35508127189107
H	5.54085001397277	4.60923786835869	3.79685487102070
H	4.09995023678355	0.30059904996393	4.54794544145245
H	-5.70310109559419	1.01362354963841	6.65833453509327
H	1.98725094609423	-1.54480279989786	7.42109378802839
C	2.63713444574721	-4.03403197683474	6.46019433319645

H	0.72340262713389	-3.68920522329039	7.43782064102065
H	0.65293548277837	-3.67463250648554	5.66051866462807
C	3.42074882554247	-3.56211002422668	5.23376589628995
H	3.20451362067376	-3.79496080055384	7.37690650512102
H	2.51434027717597	-5.12822977802731	6.43844093040690
C	3.56973054843933	-2.03688501476500	5.20228614485327
H	4.41915381753818	-4.02653277405676	5.21033406157456
H	2.89802623283866	-3.88749906264929	4.31855797784844
H	1.60758835777410	-1.65413750013431	4.40083264892707
N	1.50235179640179	1.27724661491589	7.75841164871498
C	2.13046902459623	1.53360283783358	8.69186051351414
C	2.92029284387416	1.85610272508155	9.86233949419882
H	3.83403229067495	1.24232126168253	9.86752395327050
H	3.19397219415160	2.92200910538652	9.83737961990727
H	2.33343586137982	1.64899895881090	10.77023994247977
N	-1.98304249060670	3.52452849817359	4.34764726117073
H	-2.45740687383122	2.61994741480865	4.38382252904631
H	-2.38430337122402	4.06817859133664	5.11510622276537
H	-1.01202070954949	3.34149535587764	4.61792978662769
N	3.59091507375764	-1.82294374153292	-1.91113400352182
H	3.38643608233493	-2.71458367897091	-1.45281173271708
H	3.70278287429692	-1.15589619036509	-1.14359043748257
H	4.52687574329340	-1.93095200279574	-2.30963649908192

2[3][INT7]$^+$ + N_2

Ru	0.67575583683760	0.74052140742986	1.39512835634892
N	0.34329687685210	-2.92711697533927	3.32984367705451
C	-2.58764598171784	0.82816512619318	0.86934242728789
C	-4.00090415680996	0.95671220437500	0.86701111512162
C	-4.83320544917363	-0.11147152028927	1.15022627246249
C	-4.25947163025483	-1.37038346674413	1.42583649187579
C	-2.88545624634933	-1.54140315850387	1.41672448878692
C	-2.00444777358309	-0.45739406350735	1.15258240703956
C	-1.82798976158800	2.00221757632798	0.51286773096269
N	-0.53802898237219	2.11401943205423	0.57147668651172
O	-0.71697531392941	-0.69267149795641	1.15131170140991
N	1.96567754206343	2.28680901861769	1.44622828498826
C	3.24643267147704	2.15720865275378	1.57563135025544
C	3.93758542680098	0.93470011517633	1.90561018010813
O	1.97884260996397	-0.39809743492465	2.42301966587840
C	3.27875651788531	-0.26427771994643	2.34225495075742
C	5.47411812387607	-1.27393563535697	2.73643054378335
C	4.09104274566920	-1.35320509996950	2.75717616457586
C	0.19763085948488	3.31581494977152	0.13755710891270
C	1.30266988919006	3.57526744456112	1.18815472940836
H	-2.41454559656129	2.85424646012934	0.15136830106638
C	-0.63648932499431	4.57046893950046	-0.10520113469062
H	2.75293483063284	4.45417473888050	-0.14852034842614
H	2.95175676738596	4.92919374883005	1.55192324331666
C	5.35378203089452	0.98292153704750	1.90989362696889
H	3.58272743435445	-2.26287385595311	3.08275383763410
C	6.12212831799015	-0.09725782616151	2.30855344965158
H	-4.42840042341091	1.93615668209153	0.63524908654675
H	6.06602074129986	-2.13571545192657	3.05667807326967
H	-2.43523731401111	-2.51392140235654	1.62580349495237
H	5.83778191805654	1.91183826581596	1.59611126261514
H	-5.91756736103947	0.01576066922430	1.15297612961489
H	7.21219919855626	-0.03737669474593	2.29890290630471
H	3.88095255262866	3.04055769530785	1.45469010461794
H	-4.90553664344798	-2.22426725054118	1.64782439196133
H	0.70837987373813	3.03754982718380	-0.80230193982753
C	0.26692833622243	5.74062150673828	-0.51265443759468
H	-1.38202309985287	4.38332228358969	-0.89313083189308
H	-1.18693507790558	4.82402410910188	0.81762655192674
C	1.36732711512903	5.99102093331141	0.52098931944827
H	0.72876335241466	5.51724649280455	-1.49062815339902
H	-0.34484824786166	6.64626559203709	-0.64983344952003
C	2.20499551423390	4.73144222939989	0.76886782276643
H	2.02408616235837	6.81200529979885	0.19265950778090

H	0.90880154344355	6.31073061471658	1.47357372379566
H	0.79767947928488	3.85394304274006	2.13074504037888
N	1.56794448322013	0.21250839942695	-0.34471158761114
C	2.14056811198546	-0.07919560700311	-1.30509881818294
C	2.86847644900287	-0.44164060489089	-2.50644280931144
H	2.65835401146455	-1.49011296532556	-2.76700352464720
H	2.56030161557244	0.21004162133433	-3.33815151164281
H	3.94828156659700	-0.32006504987555	-2.32986928235601
Ru	0.33568124446402	0.73176712530579	7.52797202541545
N	-0.21718530481353	-2.23467402281232	3.97451168164684
C	-1.74601485252118	-0.55416510145787	9.76029159910017
C	-2.72865184645656	-1.20245858159322	10.55416183116888
C	-3.86605088923387	-0.54814199267114	10.99051701840914
C	-4.04320868507901	0.81078311488728	10.65450834379091
C	-3.09848373451632	1.48333641274575	9.89843043694961
C	-1.93073448229936	0.83194510744697	9.41576781904594
C	-0.58099566820087	-1.33115157722179	9.41277030959836
N	0.37116133238866	-0.95434109052084	8.61793150291151
O	-1.07571264002459	1.53423403431490	8.71717277136901
N	1.81358724452267	-0.19253172087636	6.51815679677340
C	2.68677207846947	0.42840515839259	5.79108596612693
C	2.63248452004466	1.82796383036152	5.43784901527637
O	0.36401577344086	2.24567180256655	6.19032845589409
C	1.47307771392752	2.65895915865983	5.62246253254572
C	2.66064807150520	4.48510720584311	4.51017113159986
C	1.51726991824042	3.98158616537012	5.11115332516487
C	1.57751817584321	-1.76157804143009	8.35241732712822
C	1.88972890724402	-1.62545207123311	6.84404974955359
H	-0.51150425602651	-2.31673872357393	9.88623603407029
C	1.51343375706691	-3.22890503713112	8.76801109282854
H	4.03450152476733	-1.83904861593253	6.97265398238933
H	3.35014098536987	-2.25783564870719	5.38753887470902
C	3.77682379484858	2.37423541283013	4.80809124769386
H	0.62250548862749	4.59680195537653	5.22768704984819
C	3.81081967098296	3.68527389331295	4.36585613988934
H	-2.56511380617350	-2.25089478096899	10.81831822745762
H	2.66524690085726	5.51536505658147	4.14477357039022
H	-3.22752527935821	2.53700790367963	9.64108060023774
H	4.64637148301923	1.72820186468092	4.67055037669068
H	-4.61155177499420	-1.07165067914891	11.59236351959886
H	4.70749694731979	4.08500456728156	3.88894847616137
H	3.53752392291193	-0.13115609869682	5.38731180187659
H	-4.93493064873780	1.34326260763140	10.99661995027291
H	2.40302922737931	-1.26889079842400	8.89766469352958
C	2.80986644010213	-3.95245832520688	8.38289279192593

H	1.35237303236762	-3.30816299273262	9.85405803327102
H	0.65225753706347	-3.70879039422234	8.27109827022230
C	3.11766747510951	-3.80889197294255	6.89063372217990
H	3.64596017499179	-3.53208853800436	8.96931721601581
H	2.73146373415388	-5.01580695943038	8.65916992095018
C	3.18568136455997	-2.33625245452611	6.47180594365554
H	4.06903928681204	-4.30636388141217	6.64452255001409
H	2.33144246649806	-4.31468876097766	6.30262455586218
H	1.05856772330232	-2.11167585722559	6.30472055769970
N	1.79822980420231	1.64156036993224	8.58688368474315
C	2.66769579150070	2.17765367953494	9.12698560050864
C	3.76652008343516	2.85231106175822	9.79110440950419
H	4.51020061132425	3.16080847589347	9.04023947815858
H	3.39205920784441	3.74197366554261	10.31984804017973
H	4.23962118499373	2.16971004624195	10.51330097061161
N	-1.23451754461226	-0.12681018330609	6.40830770477621
H	-1.85289035605355	-0.71703798918617	6.97210250711657
H	-1.82264493345504	0.60903602228909	6.00696732604005
N	-0.21560073988242	1.19635413489285	3.23917925901103
H	-1.09808376321035	0.69187092412962	3.36320939766692
H	0.39617645528145	0.89611359158699	4.00020749204239
H	-0.92882036080369	-0.70960504020807	5.62450804587999
H	-0.42444161203597	2.18411024775591	3.40526086580219

^{2}[INT6]$^{+}$ + NH_3

Ru	0.05124629046325	0.25694441338190	0.26071230935805
N	-0.42639536656241	0.52313676895965	2.16082390463368
C	-3.24975927830108	0.53350054569272	0.29889136682044
C	-4.63804807844852	0.70912464938448	0.53753452813714
C	-5.47261715163090	-0.36059704892149	0.80784749444307
C	-4.93361994219139	-1.66450847478008	0.81924274037464
C	-3.58874203395782	-1.87956388599274	0.56790270775102
C	-2.70222260974234	-0.79816080580660	0.31850466964316
C	-2.49387879343797	1.71571678959931	-0.03375524819102
N	-1.20832128197875	1.77712625203100	-0.18017269118712
O	-1.44427151643958	-1.07518750417946	0.07884715720984
N	1.42495239285677	1.73603134743615	0.22249938391769
C	2.69224398284780	1.53905053575988	0.05544630089244
C	3.34949898065888	0.25513381565581	0.05774285138225
O	1.45508352233589	-1.06903589561603	0.80168701503213
C	2.71368255151541	-0.97688478234023	0.44359253508061
C	4.84849128342172	-2.12896574438809	0.14077658906975
C	3.50735843489307	-2.15292044214524	0.48762103885439
C	-0.48352742896531	2.99838942117830	-0.58036410353639
C	0.80163995965183	3.06692064822459	0.27931678482342
H	-3.08190928234391	2.62773903596878	-0.18714123099864
C	-1.27077309207476	4.30304866664179	-0.49926698951927
H	2.05596782225947	4.08544150185279	-1.15492027781713
H	2.55324795677196	4.28945228222512	0.54576910420311
C	4.73079509488808	0.24282917694192	-0.26441592711524
H	3.01743493562828	-3.08035813157521	0.79159556580428
C	5.47485298503978	-0.92429256753965	-0.24063678033491
H	-5.04213970236877	1.72453628918497	0.50511223765351
H	5.42664598885924	-3.05652813994241	0.16829377282483
H	-3.16629974457247	-2.88658579013453	0.56860395080801
H	5.20478134549243	1.18934044101208	-0.53828414358724
H	-6.53523147107714	-0.19957796937326	0.99986027133385
H	6.53433920969729	-0.91125898524033	-0.50389201950142
H	3.34371462359784	2.40839463702404	-0.07977633202495
H	-5.58410020706671	-2.51876555754622	1.02551684484153
H	-0.16259119998850	2.83466344745127	-1.62534145441513
C	-0.38230814161134	5.48720590244164	-0.90007791773548
H	-2.15109068167829	4.25729963238094	-1.15848064278710
H	-1.63602631751115	4.44067230899932	0.53345551490812
C	0.88939633191326	5.55014574330611	-0.05115528723406
H	-0.10771235890480	5.38978492424741	-1.96535362813714
H	-0.95525106625587	6.42300402169202	-0.80424538910615
C	1.68473653808525	4.24158197116704	-0.12714828722130
H	1.52742532605607	6.38750956072442	-0.37516415507440

H	0.61770339743422	5.74602686923225	1.00129822891708
H	0.49331630029334	3.21080025219357	1.33037898796837
N	0.52815323590863	-0.05432037186280	-1.66313856743665
C	0.85723519566474	-0.21498742877048	-2.75712160559331
C	1.28156382463599	-0.41179615878150	-4.12804844393401
H	1.03397891195400	-1.43531417766047	-4.44836652154751
H	0.76791279128384	0.31191785876768	-4.77944218723565
H	2.36958087813038	-0.25813689820953	-4.19614887256912
N	-0.71548330061221	0.66753011519776	3.21959793919762
N	2.86997395700241	2.68784455031567	2.98279864541795
H	2.15290145980932	1.96052209208925	3.03819124037630
H	2.95625999459145	3.04698415623482	3.93721718107905
H	3.74026454407998	2.17575613620943	2.81534984108308

^{2}[INT7]$^+$ + N_2

Ru	0.04045216417451	0.43284322425605	0.13923946381498
N	-3.23911469725459	0.97374601351020	3.59823967486907
C	-3.27055030543990	0.64586885226264	0.25239407551525
C	-4.66210258823316	0.81377375297715	0.48155922332277
C	-5.47267219785172	-0.23666106213675	0.86960945510522
C	-4.90221865992154	-1.51866313001181	1.02198516998186
C	-3.55532124799486	-1.72896451687529	0.78112916143623
C	-2.69159890489870	-0.66608023944003	0.39694761569669
C	-2.54083448244704	1.81709853386548	-0.16508017900821
N	-1.25560954565378	1.89442540881618	-0.32245202680284
O	-1.43472261483705	-0.94536504932935	0.16670243608403
N	1.38170445181903	1.92592639403604	0.02988330019289
C	2.66173690410483	1.75339652212136	-0.07508698728928
C	3.34981434873901	0.48925663276800	0.01598837237078
O	1.46300399766415	-0.86293291134838	0.73703333175807
C	2.73008982594396	-0.74305635369576	0.43273389442473
C	4.91543428373568	-1.84005360054454	0.27243961623160
C	3.56164503560043	-1.88964535070787	0.56037409221543
C	-0.56823402204278	3.10598934138704	-0.81103124508367
C	0.73271622109219	3.24973879177009	0.01262454237172
H	-3.14992100975047	2.70438159687517	-0.37188251712977
C	-1.37908767185631	4.39917603658444	-0.78996383395861
H	1.93118056140871	4.19725694892511	-1.51236602888044
H	2.47529378620859	4.53003601544532	0.14653388426439
C	4.74471490644401	0.50359297316813	-0.24907623963979
H	3.08748018126503	-2.81839975257867	0.88569671969696
C	5.52309547300774	-0.63491345858846	-0.13915869940141
H	-5.08659869294475	1.81332311567398	0.35312601221559
H	5.51888958253341	-2.74689072525827	0.36944590945409
H	-3.11053140629909	-2.72070667230506	0.88964542403550
H	5.20114315224391	1.45127521867557	-0.54891545356750
H	-6.53697489330196	-0.07825557490866	1.05443412979737
H	6.59230238265359	-0.59987218468160	-0.35788262242035
H	3.30088009546354	2.63083771678306	-0.22647141478052
H	-5.52989455343552	-2.35908141516158	1.33091018938995
H	-0.26649963021377	2.88898282758115	-1.85200993483588
C	-0.52495581414507	5.57350008523279	-1.28319488176360
H	-2.27216643870209	4.29740524939809	-1.42503030653131
H	-1.72628824492040	4.59245192265915	0.24016152903730
C	0.76617953345175	5.71220047734099	-0.47371335450770
H	-0.27406212900578	5.41626935528871	-2.34716800605661
H	-1.11315400897861	6.50325738861433	-1.22962521287865
C	1.58303973079030	4.41459651139214	-0.48747478446104
H	1.38007357731148	6.53775110736126	-0.86734530591352

H	0.51711548559357	5.97075279616984	0.57078440890005
H	0.42985320980472	3.45763958468655	1.05547752248227
N	0.48775026932573	0.02100016537564	-1.79175684568497
C	0.82351991039882	-0.17960972199231	-2.87913787533302
C	1.25838148672663	-0.42352421762405	-4.24139976162594
H	1.11556306615349	-1.48492867668115	-4.49502179135343
H	0.67297207873777	0.19990517739565	-4.93437649149653
H	2.32507246589143	-0.16706125977763	-4.33468104326262
N	-3.97517540841283	0.22870739903525	3.93569669260423
N	-0.33555247796684	0.80175529088650	2.17258146339654
H	-0.69680293538019	-0.03869404025501	2.63265181640350
H	-1.00860976299913	1.55120753620213	2.35496156994948
H	0.52290617659995	1.05572394938081	2.67035214664868

^{2}[Ru(salen)(NH_3)(MeCN)]$^+$ (^{2}[I]$^+$)

Ru	-0.06328445192763	0.29887170924875	-0.05650276949056
N	-0.49141816711819	0.47735692231505	1.99517781214627
C	-3.36308364443430	0.59531097564994	0.05855305289043
C	-4.74941864281684	0.77850542478729	0.30751628894215
C	-5.59579981649312	-0.28656873767889	0.55772115690790
C	-5.06915403119959	-1.59580995045931	0.54223112542231
C	-3.72706394042501	-1.81738932602084	0.28403468471955
C	-2.82602186330706	-0.74202346716410	0.04812169804644
C	-2.59809094352097	1.78006483709732	-0.24245899625001
N	-1.31181772779876	1.83068876586042	-0.40043965200278
O	-1.57330768890313	-1.02923243786126	-0.19688535087088
N	1.32180596137443	1.76045699653452	-0.03056641252108
C	2.59424009562528	1.55686485768911	-0.16617266416561
C	3.24185990603389	0.26754680257856	-0.19030986008948
O	1.32057432151721	-1.07111888196853	0.45475830327484
C	2.58676540399489	-0.97276303173387	0.13614975793882
C	4.72533024720361	-2.12815698502252	-0.16612629654242
C	3.37675513537968	-2.15425080720470	0.14966235228402
C	-0.58659139419036	3.05343262460060	-0.79499636833046
C	0.71363600182927	3.10147896334484	0.04233074614782
H	-3.17920978751895	2.70156622833327	-0.36209595830527
C	-1.35961585283752	4.36505618827423	-0.69106072994717
H	1.94590716543735	4.11093931926660	-1.41435531112162
H	2.49292821262853	4.31963027029635	0.26336808416733
C	4.63026037468374	0.25590398485943	-0.48546704249485
H	2.87490021067854	-3.08996219779504	0.40634401582752
C	5.36807382758488	-0.91509116943198	-0.49014481485403
H	-5.14244878391301	1.79899749423384	0.29869061624472
H	5.29617003739665	-3.06090653933010	-0.16132321391778
H	-3.31501710247618	-2.82883541595122	0.26518813892289
H	5.11507331545491	1.20866518496676	-0.71666803438782
H	-6.65635813497827	-0.11839407423789	0.75567459277963
H	6.43268300371037	-0.89950541269032	-0.73221338495245
H	3.25960259835059	2.42299325691891	-0.25780177958115
H	-5.72726342212291	-2.44805068318602	0.73333499764596
H	-0.28310692020852	2.90025303742759	-1.84688727573663
C	-0.46837270891491	5.54225103058487	-1.10530283370144
H	-2.25316304214512	4.33084982559319	-1.33300655140741
H	-1.70507042568411	4.50251068516095	0.34853078108151
C	0.82166082089106	5.58898394572247	-0.28356236060484
H	-0.21694767531537	5.44629508944418	-2.17644645370638
H	-1.02857112373413	6.48410264835764	-0.99414935327840
C	1.59981567053460	4.27101013894525	-0.37830213692046
H	1.46249651589390	6.41934747137397	-0.61992977450645

H	0.57438316239971	5.78646850926293	0.77456896539449
H	0.41227117291730	3.24911341159159	1.09575454042594
N	0.39054811289014	0.04323738088508	-2.01165786471757
C	0.68657559840348	-0.08432924400873	-3.12134351590554
C	1.06542086239354	-0.24143355693243	-4.51300829486485
H	0.77184235213083	-1.24090706359386	-4.86857232934996
H	0.56196878080444	0.52490171999133	-5.12221434040387
H	2.15572751567586	-0.12530280640242	-4.61181125099236
H	-1.15460815071445	-0.24495066273984	2.29147448161368
H	0.35453157248465	0.33602899886088	2.55507441819329
H	-0.88787251360487	1.37654775135598	2.28258236490414

^{1}[Ru(salen)(NH_2)(MeCN)]$^+$ (^{1}[II]$^+$)

Ru	0.01656000964495	0.30586030848788	0.13391824517686
N	-0.15818955882851	0.48913655107128	1.94974813244156
C	-3.28724564251741	0.51258689940403	-0.08711133543673
C	-4.70413774403233	0.68578454622462	-0.09610562339206
C	-5.57020651698821	-0.35794993535968	0.14494624320265
C	-5.03674332195819	-1.64651039155066	0.39524206049425
C	-3.67415959830165	-1.86362032542326	0.40247057854310
C	-2.74188934409702	-0.80552859270932	0.17032672251071
C	-2.51401083083188	1.67663307911816	-0.38479187241047
N	-1.21469329466798	1.78649642625988	-0.36236329387219
O	-1.47966166513151	-1.09434020084846	0.18820668032669
N	1.41146992700926	1.75596474650977	0.05642449296563
C	2.68634882300818	1.55981710751305	0.00533308836047
C	3.34098482657186	0.26979227722671	0.00063006977822
O	1.34886236939240	-1.14270165443845	0.05044297584387
C	2.65762118390955	-0.99171258493301	-0.00480447943187
C	4.82148957838577	-2.12768551228934	-0.10051450671293
C	3.43529041496690	-2.17459708889986	-0.07443204417669
C	-0.49368292450921	3.00744562872225	-0.78380766357769
C	0.79271369120575	3.09675931851185	0.06162724784526
H	-3.09380683279947	2.56621584846039	-0.65578297174511
C	-1.28622791600977	4.31162356001457	-0.71883566244158
H	2.02779183522843	4.06328818490823	-1.42116693155727
H	2.56351260172339	4.33264306083471	0.25197839611797
C	4.75695947608732	0.27877776896059	-0.04125463120868
H	2.90049179978056	-3.12657674636481	-0.09088968990535
C	5.49677044115178	-0.89283517004999	-0.08301532981853
H	-5.09546173766597	1.68617952908333	-0.30075067992301
H	5.39015925064324	-3.06057338622918	-0.13885408509765
H	-3.26059313919254	-2.85645723240401	0.59241867564382
H	5.26591652698224	1.24642068678216	-0.04059189466837
H	-6.65014313497896	-0.19790062864950	0.14070385971201
H	6.58770259130835	-0.85706267216445	-0.10874542121340
H	3.35273103453789	2.42800764110835	-0.04837584149532
H	-5.71446377068445	-2.48353073032424	0.58532482295925
H	-0.18285385613937	2.83651021583668	-1.83069295715100
C	-0.40465700399022	5.48715159013138	-1.15957691174731
H	-2.17173030254257	4.25489215383285	-1.36945931092219
H	-1.64237005862369	4.46821534813240	0.31439256248768
C	0.88518059830961	5.56962846534425	-0.34038129048213
H	-0.15227562544309	5.36674095795329	-2.22796909388959
H	-0.97504095559207	6.42532106966483	-1.07207263940913
C	1.67509380816616	4.25589063955090	-0.39288310887625
H	1.51858776109829	6.39449247112372	-0.70306188721296

H	0.63652276601749	5.79785219954309	0.71115711394213
H	0.48168739418246	3.28303287886369	1.10581495785376
N	0.09031385964508	0.08513656443085	-2.02228841075879
C	0.12167964268308	-0.01509488129242	-3.17164488872762
C	0.16303661342431	-0.14192898734504	-4.61520399624180
H	0.15106644576915	-1.20689460353636	-4.89260209314066
H	-0.71407416077637	0.35978734198013	-5.05184470055169
H	1.08111826009915	0.32780533144605	-4.99979381400954
H	-0.64288936479525	-0.21949208493233	2.50771654041558
H	0.16790477016504	1.28090301270828	2.50906559458416

^{2}[Ru(salen)(NH)(MeCN)]$^+$ (^{2}[III]$^+$)

Ru	-0.02628823784063	0.33688019287917	0.26304000035955
N	-0.19821950141453	0.55118708110486	1.96463266396096
C	-3.32340196217664	0.53294282379207	-0.06921537942379
C	-4.73671546207410	0.69106688358919	-0.07568638670262
C	-5.58953412621572	-0.37219721015258	0.15171711106225
C	-5.03995198727602	-1.65232508064925	0.37800978974963
C	-3.66922736103339	-1.84975540691468	0.37825398836799
C	-2.76490222713368	-0.77460729597393	0.16683792390065
C	-2.55267089321526	1.70639064814766	-0.36619381066189
N	-1.26028769126737	1.81625180314421	-0.33892433988420
O	-1.48505593994279	-1.04713408425340	0.17947596007807
N	1.37899940957743	1.77586943059391	0.06242314169797
C	2.64581575921809	1.55379938944584	-0.07237027699532
C	3.28736207314887	0.26329744155863	-0.07014863513634
O	1.29512384238125	-1.13923459392597	0.15801154440084
C	2.59957182482039	-0.99183986494387	0.04036339387078
C	4.75609940647800	-2.13628333397090	-0.10097527872014
C	3.37384482194821	-2.17743699338845	0.01261461868557
C	-0.52732414178397	3.02930028444823	-0.76671553614610
C	0.76311569127331	3.11541921582032	0.07460004374383
H	-3.13378219065787	2.59120668873127	-0.65063256797895
C	-1.31523891574834	4.33592058775700	-0.70756974171822
H	1.99231534969288	4.07869170252303	-1.41583421434808
H	2.53835033396728	4.34169569994374	0.25608631397088
C	4.70135167047926	0.26523559917792	-0.19670796082747
H	2.84143576922244	-3.12751412783705	0.09108651352940
C	5.43596829369784	-0.90775806848358	-0.20794629437008
H	-5.14230388549110	1.68806408112958	-0.26729702926539
H	5.31954133015017	-3.07320023339515	-0.11060852195068
H	-3.24061170636410	-2.83938333740864	0.54900067900921
H	5.20799514521920	1.23009820006097	-0.28317219111277
H	-6.67137170353124	-0.22628149815079	0.15009477747939
H	6.52330800239965	-0.88103840906469	-0.30025090623850
H	3.31437310369080	2.41325251848821	-0.19910084955666
H	-5.70354327140162	-2.50327665100728	0.55413412226245
H	-0.21867296007258	2.84562282649930	-1.81172708175835
C	-0.42902085997573	5.50743760296152	-1.14951478147329
H	-2.19922481385682	4.27946220516838	-1.36030967170298
H	-1.67256054322888	4.49511448230626	0.32473364843469
C	0.86012186208001	5.58639617045713	-0.32901903523526
H	-0.17644783587545	5.38501621843986	-2.21753284856458
H	-0.99723863106567	6.44687748185002	-1.06312103156387
C	1.64652461965148	4.27084628610357	-0.38516650162002
H	1.49583564657690	6.40981982023358	-0.69054192398274

H	0.61188579500116	5.81321528564850	0.72278425580111
H	0.45914465446657	3.29394048027428	1.12196063826048
N	0.14539548325641	0.10872833895980	-2.02700168038998
C	0.25573107573607	-0.00444378873499	-3.17071547298827
C	0.39540215256118	-0.14495462058588	-4.60706846036828
H	0.01230858139363	-1.12797984698999	-4.92043791455815
H	-0.17623648700125	0.64899834676438	-5.11120765341976
H	1.45800341303005	-0.06169742025838	-4.88166593621720
H	-0.88863177547375	0.00908604808695	2.51740878625429

Coordinates of $Ru(NH_3)_2$

^{1}**[INT1]**$^{+}$

Ru	-0a.03714212602489	0.40596124681438	0.79034679980173
N	-0.10235766830483	0.77502358648081	2.33463469345143
C	-3.31163152575142	0.44564784170122	-0.09071600137051
C	-4.70250759554629	0.52445686679237	-0.37959002253255
C	-5.52855580608960	-0.57789366703094	-0.27391190153432
C	-4.96961651507407	-1.81308635905555	0.11404038194379
C	-3.61644471483274	-1.93101843768240	0.39389339469044
C	-2.74935477307416	-0.81430462852336	0.30822294343965
C	-2.54404179887668	1.63518425452917	-0.31949080390378
N	-1.28136384594109	1.82762954192751	-0.07082325211984
O	-1.47293000158358	-1.02386772094038	0.56793367731454
N	1.38405503268403	1.77512911654159	0.17323833134303
C	2.60743091276174	1.48605178029912	-0.14669528586798
C	3.22732671810976	0.18879065475896	-0.10983851257111
O	1.30210532409267	-1.11448130118169	0.66782076924233
C	2.56423641108787	-1.02136941203031	0.28510859609919
C	4.63075417964510	-2.24587324566500	-0.15786164094332
C	3.30310536755398	-2.22767854581072	0.24482217062834
C	-0.55680275905300	3.01729017064098	-0.61305763398690
C	0.79696571372859	3.13576513066513	0.10881235702464
H	-3.08916795463720	2.45915771960083	-0.79546630148146
C	-1.31904089400379	4.33944867189326	-0.52827244158158
H	1.88846091524498	3.95526086946370	-1.56563918552517
H	2.61696904331554	4.30242866916430	0.02111958290589
C	4.58727492305140	0.13010080420702	-0.51822772111204
H	2.78860840631829	-3.14484872703517	0.53811578601807
C	5.28855326028756	-1.06159569993107	-0.54403344614868
H	-5.10833737633987	1.48861973823610	-0.69675665243728
H	5.16821905368879	-3.19781479324252	-0.17783297654628
H	-3.18082674630185	-2.88888427350286	0.68467737037534
H	5.07548694840129	1.06112137223039	-0.81806259734712
H	-6.59409056134388	-0.49783301766778	-0.49685344394495
H	6.33267273888978	-1.08523438821140	-0.86108278494928
H	3.24675540797597	2.30633536582630	-0.49268253857197
H	-5.60869453109850	-2.69703076121835	0.19038971041495
H	-0.34716709998394	2.78483175167802	-1.67210264785971
C	-0.46603800662833	5.46778875154285	-1.12421806109398
H	-2.27177097659199	4.27634704917555	-1.07378290045969
H	-1.55461347647024	4.54709083637730	0.52962108929173
C	0.91133255057411	5.55983318091968	-0.46250749045576
H	-0.34069204845379	5.28708539258541	-2.20629513673269
H	-1.00402106525795	6.42317192273126	-1.02161101913225
C	1.66128899042193	4.22175007018031	-0.51899660858158

H	1.51790576355460	6.33983954717842	-0.94841114598490
H	0.79227396578156	5.85920997574096	0.59348251740790
H	0.58966717223164	3.41389947749026	1.15793511109843
N	0.09567404107277	-0.13625363567118	-1.91881133342375
H	0.32683461899760	-1.12699110274946	-2.01310421220358
H	0.78264840173240	0.38000006852245	-2.47160827201624
H	-0.80787599374025	-0.00415170904550	-2.37790130987118

[1][INT3][0]

Ru	0.16300770588662	0.43822323793973	0.04695778376452
N	0.06652180122125	0.71751238569541	1.91000114909826
C	-3.16782184107540	0.52202832062515	0.05417815149634
C	-4.57851935272177	0.64677683153529	0.16827259505642
C	-5.40035737349105	-0.41514057185969	0.50640677266317
C	-4.81085091675367	-1.67686256827813	0.73270993747680
C	-3.44179888867361	-1.84590230045140	0.61633461631441
C	-2.56075627293743	-0.77271765917175	0.27873801914349
C	-2.45956128758353	1.72706184878009	-0.32988457816982
N	-1.17372337601413	1.87317195905985	-0.37630333086064
O	-1.28979355580655	-1.03499999410796	0.18502644770212
N	1.48482348463056	1.92195303515403	-0.15111310057261
C	2.76988684993700	1.77009252472001	-0.21485045579775
C	3.48807076046979	0.51589675505647	-0.09681195905594
O	1.61230972874755	-1.01894173714579	0.23209069011663
C	2.88757042372666	-0.78343393552942	0.12256291016880
C	5.15469293060701	-1.75974925583214	0.12024623281256
C	3.78036553664654	-1.89518431863352	0.22080548414384
C	-0.52313585650207	3.09861439262472	-0.87909562699897
C	0.82917765751178	3.24149558846538	-0.14960213160876
H	-3.10033596070947	2.57391551072082	-0.60570410214358
C	-1.33623513123154	4.38976766073742	-0.78444852764538
H	1.90890861230117	4.19632091518130	-1.75817064564946
H	2.57420445729312	4.53309799644643	-0.14788261861099
C	4.90211666044236	0.60783720758290	-0.19728173889848
H	3.32596664353042	-2.87613240660059	0.38415943388838
C	5.73651088952872	-0.49271213883750	-0.09201235725099
H	-5.01802678834137	1.63162027633250	-0.01886280232904
H	5.79091937576780	-2.64575737002451	0.20724458805000
H	-2.98155757446000	-2.82255517169013	0.78844640012747
H	5.33551180934498	1.59963574796178	-0.36098609013463
H	-6.48058854915985	-0.27904013437246	0.59338711462791
H	6.82018743836344	-0.38068476591729	-0.17047547872176
H	3.40315188309484	2.65419202892110	-0.35832221815629
H	-5.43812198433383	-2.53220924343221	1.00143907233419
H	-0.29305717282965	2.91464271129106	-1.94598107068538
C	-0.53050038487493	5.57305599779929	-1.33419979093497
H	-2.27651067098781	4.29300247476588	-1.34796312820319
H	-1.60480496267275	4.57016383653620	0.27146891255727
C	0.82273353141035	5.71053009501781	-0.63349498220583
H	-0.36658810699608	5.42482388487953	-2.41672463190884
H	-1.11439651007105	6.50150389915242	-1.22718162531400
C	1.63579169766423	4.41259725524858	-0.71007564735400
H	1.40137159156879	6.53812969373592	-1.07483375617569

H	0.65836610547122	5.96950755441576	0.42785063624866
H	0.59391975052923	3.45677746299064	0.91026412122935
N	0.31145313121198	0.04090508152665	-2.13193776652995
H	-0.60649543354653	-0.05337833928873	-2.57262315491555
H	0.82218681684371	0.74514803504340	-2.67016035651783
H	0.79746929081900	-0.84676985154116	-2.27734772018701
N	-0.09097433990816	-0.30804130990681	2.65479758890792
H	-0.24163718019969	-1.24285612644778	2.25050666818434
H	-0.16061709268819	-0.20443300687469	3.66941606742453

^{2}[INT4]$^{+}$

Ru	0.23832570157992	0.36316863688744	0.08917749203275
N	0.13138624569084	0.64148515008459	1.96497356609929
C	-3.09734300199052	0.42204388351516	0.09005351271526
C	-4.51165026997172	0.51877387870239	0.21602621049320
C	-5.30020447471110	-0.58267195309850	0.48521215674285
C	-4.68381828016017	-1.84622191018808	0.62414661633641
C	-3.31365290252528	-1.98526434161749	0.49828150821968
C	-2.46869607677128	-0.86903627537941	0.23593650158959
C	-2.39991558962221	1.64090204077047	-0.23280911998311
N	-1.11235706016073	1.79076612216288	-0.30629153073994
O	-1.18550482054439	-1.08502693449445	0.13632396379351
N	1.55501275170791	1.85966182336007	-0.12400887496162
C	2.83484632210760	1.70874929085022	-0.26732116681486
C	3.55375714623882	0.46181650843829	-0.23287590412375
O	1.69810714023912	-1.03325973815045	0.28979915441919
C	2.96316976162012	-0.82525571857598	0.04801823571763
C	5.19279318051565	-1.83177852138224	-0.15700381252342
C	3.83453288657530	-1.95264105052595	0.07557579205184
C	-0.47080498865709	3.03678369802933	-0.78369647956123
C	0.88687208304961	3.17373256968852	-0.06273749194092
H	-3.03984762924365	2.50554549642325	-0.44297768362556
C	-1.29744269513435	4.31404158195536	-0.65001072516126
H	1.94197833841292	4.18221169200211	-1.65338352944775
H	2.61951657727184	4.47583614335081	-0.03727263473333
C	4.95509896201220	0.54245286974458	-0.46098814129198
H	3.38361168883780	-2.92431285884073	0.28949979107177
C	5.77078967918104	-0.57269723716580	-0.42996126968677
H	-4.97086441244826	1.50349880452714	0.09292348237385
H	5.82436501232726	-2.72409096810156	-0.12677917205256
H	-2.83221351594989	-2.95990214773525	0.60441496377623
H	5.38478487123290	1.52750826154161	-0.66377652381669
H	-6.38267104535895	-0.48006747986810	0.58355125089697
H	6.84394278521345	-0.48175334799979	-0.60934635628561
H	3.45192534778441	2.60247530664918	-0.41650226013347
H	-5.29538413814810	-2.72811737160156	0.83411324976626
H	-0.25089020872097	2.87129121229316	-1.85490921018492
C	-0.50385189643117	5.51865728950890	-1.17105793469655
H	-2.23710527279888	4.22289061672928	-1.21505500438141
H	-1.56271345995640	4.46144778433873	0.41158799339352
C	0.85126252331706	5.65034803386342	-0.47335642490807
H	-0.34546681652603	5.40228131764609	-2.25793399715308
H	-1.09746991630506	6.43630949767702	-1.03377991104254
C	1.67843801150023	4.36511549659902	-0.59693775252916
H	1.41887767049798	6.49554019721201	-0.89387326137945

H	0.69188501133259	5.87434090024124	0.59628849715058
H	0.66517893252359	3.34833069391162	1.00670106265959
N	0.38052324086294	-0.04380474732292	-2.04815301470091
H	-0.51730498791043	0.06800075729324	-2.52669571232666
H	1.05349652448829	0.54496087485262	-2.54609609194827
H	0.67231375833215	-1.01385855339033	-2.19294059897532
N	-0.89678545186180	0.74725939940240	2.66046388493229
H	-1.85559767434199	0.71267481299582	2.27636545041441
H	-0.81680556820313	0.87135851219050	3.67807725446352

^{1}[INT4]$^{2+}$

Ru	0.16300770588662	0.43822323793973	0.04695778376452
N	0.06652180122125	0.71751238569541	1.91000114909826
C	-3.16782184107540	0.52202832062515	0.05417815149634
C	-4.57851935272177	0.64677683153529	0.16827259505642
C	-5.40035737349105	-0.41514057185969	0.50640677266317
C	-4.81085091675367	-1.67686256827813	0.73270993747680
C	-3.44179888867361	-1.84590230045140	0.61633461631441
C	-2.56075627293743	-0.77271765917175	0.27873801914349
C	-2.45956128758353	1.72706184878009	-0.32988457816982
N	-1.17372337601413	1.87317195905985	-0.37630333086064
O	-1.28979355580655	-1.03499999410796	0.18502644770212
N	1.48482348463056	1.92195303515403	-0.15111310057261
C	2.76988684993700	1.77009252472001	-0.21485045579775
C	3.48807076046979	0.51589675505647	-0.09681195905594
O	1.61230972874755	-1.01894173714579	0.23209069011663
C	2.88757042372666	-0.78343393552942	0.12256291016880
C	5.15469293060701	-1.75974925583214	0.12024623281256
C	3.78036553664654	-1.89518431863352	0.22080548414384
C	-0.52313585650207	3.09861439262472	-0.87909562699897
C	0.82917765751178	3.24149558846538	-0.14960213160876
H	-3.10033596070947	2.57391551072082	-0.60570410214358
C	-1.33623513123154	4.38976766073742	-0.78444852764538
H	1.90890861230117	4.19632091518130	-1.75817064564946
H	2.57420445729312	4.53309799644643	-0.14788261861099
C	4.90211666044236	0.60783720758290	-0.19728173889848
H	3.32596664353042	-2.87613240660059	0.38415943388838
C	5.73651088952872	-0.49271213883750	-0.09201235725099
H	-5.01802678834137	1.63162027633250	-0.01886280232904
H	5.79091937576780	-2.64575737002451	0.20724458805000
H	-2.98155757446000	-2.82255517169013	0.78844640012747
H	5.33551180934498	1.59963574796178	-0.36098609013463
H	-6.48058854915985	-0.27904013437246	0.59338711462791
H	6.82018743836344	-0.38068476591729	-0.17047547872176
H	3.40315188309484	2.65419202892110	-0.35832221815629
H	-5.43812198433383	-2.53220924343221	1.00143907233419
H	-0.29305717282965	2.91464271129106	-1.94598107068538
C	-0.53050038487493	5.57305599779929	-1.33419979093497
H	-2.27651067098781	4.29300247476588	-1.34796312820319
H	-1.60480496267275	4.57016383653620	0.27146891255727
C	0.82273353141035	5.71053009501781	-0.63349498220583
H	-0.36658810699608	5.42482388487953	-2.41672463190884
H	-1.11439651007105	6.50150389915242	-1.22718162531400
C	1.63579169766423	4.41259725524858	-0.71007564735400
H	1.40137159156879	6.53812969373592	-1.07483375617569

H	0.65836610547122	5.96950755441576	0.42785063624866
H	0.59391975052923	3.45677746299064	0.91026412122935
N	0.31145313121198	0.04090508152665	-2.13193776652995
H	-0.60649543354653	-0.05337833928873	-2.57262315491555
H	0.82218681684371	0.74514803504340	-2.67016035651783
H	0.79746929081900	-0.84676985154116	-2.27734772018701
N	-0.09097433990816	-0.30804130990681	2.65479758890792
H	-0.24163718019969	-1.24285612644778	2.25050666818434
H	-0.16061709268819	-0.20443300687469	3.66941606742453

^{2}[INT5]^{0}

Ru	0.19400409967267	0.35873608327426	0.02540882079736
N	-0.08916919790997	0.67266241803186	1.85890354238903
C	-3.14019872202667	0.43876517989235	0.06569154394947
C	-4.54360347615893	0.57134166038712	0.24443234547686
C	-5.35527984567297	-0.49625558194600	0.58546882741300
C	-4.76351711706986	-1.76752978747418	0.74715242143412
C	-3.40239011560415	-1.94292803793040	0.57096152341557
C	-2.52933402637184	-0.86371431441501	0.22912136232624
C	-2.43448235192329	1.64885873790580	-0.29931195700834
N	-1.15040735199977	1.78766464306377	-0.39950491145541
O	-1.26717649942553	-1.12303868715791	0.07738763336556
N	1.51536327171287	1.86678053821610	-0.14134109527224
C	2.79968645456668	1.73071716012468	-0.20695633706230
C	3.53558953114064	0.48768134312449	-0.11503004994691
O	1.68263329483893	-1.06655786730983	0.24160201045350
C	2.95227277606189	-0.81729460261898	0.11122923025268
C	5.23024818137798	-1.76264797608150	0.07997382310877
C	3.85997807153909	-1.91783096142525	0.19897995256929
C	-0.50414698088376	3.03368781006208	-0.86053551811940
C	0.83673064882772	3.17529704102043	-0.10671415968411
H	-3.07557101609954	2.51425906898839	-0.50652251941384
C	-1.33550211960476	4.31043969117367	-0.74423453634164
H	1.92834269925115	4.19765912968367	-1.66478087398771
H	2.56077790593669	4.48817157968024	-0.03146805271533
C	4.94701018727640	0.60146466948666	-0.23191804362516
H	3.42148646678307	-2.90437973120397	0.37008136936011
C	5.79498172857505	-0.48863954330428	-0.13936776080778
H	-4.98293529018468	1.56413070705868	0.10722654017179
H	5.87925598294514	-2.63999763325040	0.15922868149312
H	-2.94258762186080	-2.92648696101081	0.69719236705760
H	5.36466579040318	1.59956850910788	-0.39655415000908
H	-6.42969405508952	-0.35871911068880	0.72402648259154
H	6.87615139391551	-0.36446286136368	-0.23053021176057
H	3.41977126651452	2.62809938191780	-0.32378926772468
H	-5.38497824374810	-2.62666536516770	1.01707557111915
H	-0.25643524230414	2.87503578342480	-1.92716161882566
C	-0.53613387861647	5.52114007065363	-1.24090790657180
H	-2.26286785285549	4.21717963562122	-1.32930382679472
H	-1.62662510229218	4.45516027335431	0.31114267383711
C	0.79998825494151	5.65614686218474	-0.50801191195634
H	-0.34823162450472	5.40975408694916	-2.32378165086895
H	-1.13685501718644	6.43634145537382	-1.11627936581126
C	1.63466410624128	4.37461251439879	-0.61506517541241
H	1.37392528454245	6.50735814103896	-0.90808781120368

H	0.61060747354267	5.87346788843184	0.55835541009987
H	0.58122078173883	3.35165941930925	0.95527301830046
N	0.36878463674670	-0.06274245907309	-2.11306968604439
H	-0.54275064735384	-0.22548706511422	-2.54692492012898
H	0.83368935949778	0.66582565751216	-2.66025810741392
H	0.91359696961505	-0.91932799895865	-2.23970367111413
N	-0.92343638938632	0.99265129319935	2.68302559523806
H	-1.86220683207181	1.19953811184252	2.25570435086050

^{1}[INT5]$^{+}$

Ru	0.15235799191280	0.39730373196734	0.14979753525347
N	0.07238364749174	0.67551510061162	1.89670628739592
C	-3.17267767862115	0.42604786348761	0.00550382335924
C	-4.59047666161439	0.51765384923522	0.08128422713395
C	-5.38529434539786	-0.58304297335730	0.33707128951399
C	-4.76760910976487	-1.84129037271995	0.50703030514295
C	-3.39287562007332	-1.97562297848472	0.42581578429962
C	-2.54169568408439	-0.85898415923893	0.18431438078164
C	-2.47754570636188	1.64201751908009	-0.32636643135969
N	-1.19363211817979	1.81519490500710	-0.35170708952718
O	-1.25762154251834	-1.08098440262528	0.12099732051769
N	1.47016908483298	1.90868791050470	-0.08394031508654
C	2.75174712163612	1.75739238392410	-0.16666421475551
C	3.47642079890577	0.51257785545916	-0.08749432462317
O	1.60281644992078	-1.02665398284921	0.24272626412653
C	2.88263666437972	-0.78658084543695	0.10541373970238
C	5.13273973502116	-1.76429710188976	0.01480294937057
C	3.76128477863835	-1.90528662851135	0.14389776547352
C	-0.54343558255021	3.04889517917316	-0.84471620319145
C	0.78717102713797	3.21740777796087	-0.08365768236600
H	-3.11804390057052	2.49007859418367	-0.59596496758147
C	-1.38442885227150	4.32200145858148	-0.77230878578546
H	1.88609077384920	4.18571039950853	-1.66877259756389
H	2.50997861624050	4.53203932659777	-0.04126404531297
C	4.88854988965034	0.61381782701113	-0.21839966698618
H	3.30764063472776	-2.88896398993221	0.28455164549634
C	5.71543350704772	-0.49299982591864	-0.16761809070754
H	-5.04872299617966	1.49909160074894	-0.06990598001954
H	5.76847176316360	-2.65329030812998	0.05548365071667
H	-2.91296182703229	-2.94854819686387	0.55334036855378
H	5.31769374473742	1.60947366368621	-0.36231935834276
H	-6.47080631295594	-0.48401865882205	0.39873687353269
H	6.79717183570151	-0.38529614132347	-0.26825876471803
H	3.37100715152293	2.65102753019491	-0.30794672883947
H	-5.38127233505201	-2.72486932001798	0.70428532765668
H	-0.28709575797458	2.85937021681183	-1.90383119624253
C	-0.58381377554415	5.51881595056976	-1.30045964146068
H	-2.30406307416713	4.20584505675269	-1.36489801527148
H	-1.68517325835996	4.49528701207526	0.27578461186047
C	0.74822896307451	5.67947390452872	-0.56532040407374
H	-0.39144028543448	5.37570900860310	-2.37852585224118
H	-1.18782442195483	6.43489696318976	-1.20464517756093
C	1.58817884830152	4.39776322813157	-0.62703067454175
H	1.32304401861743	6.51693576454385	-0.99131316577365

H	0.55431599487197	5.93174924065009	0.49222982606228
H	0.53050374982495	3.42366840493030	0.97210310094012
N	0.30023702169786	0.01303076872132	-1.95672364047400
H	-0.59166105156452	0.14585978886883	-2.44172066548678
H	0.99352466098919	0.58670666751206	-2.44508404015271
H	0.57239265625833	-0.96464307787177	-2.09154089870200
N	0.17161405512368	0.85042049656998	3.05568686901140
H	-0.67472328705014	1.04505601461057	3.62836467284640

^{2}[INT5]$^{2+}$

Ru	0.13876310440101	0.42017070195631	0.10846372000818
N	-0.19980003859930	0.73534467690725	1.85236477200843
C	-3.15605043264707	0.47989177045310	0.01191288142777
C	-4.56060537068195	0.56957765978151	0.17944579238123
C	-5.33576161658414	-0.54697098447617	0.44314195677785
C	-4.71983924978697	-1.81566107345686	0.51570009017708
C	-3.35571768015186	-1.95098094092698	0.33358298344270
C	-2.52861855827150	-0.81825254281608	0.09956788587970
C	-2.46602801484282	1.69863230344172	-0.33010133474846
N	-1.18140031694640	1.84120644460993	-0.42209474697624
O	-1.24747244607675	-1.03023983076119	-0.04344395352993
N	1.47464448222628	1.91274202141938	-0.07647277269044
C	2.74616387677375	1.74384564188951	-0.25140608559460
C	3.44823612918841	0.48463045708277	-0.18283301675574
O	1.59663876495712	-0.93064457261110	0.50822502358969
C	2.85077950968078	-0.77461929798598	0.22416300059337
C	5.04573090200772	-1.84240415431867	0.04811714227496
C	3.69882489473357	-1.91726483262518	0.34533400853472
C	-0.51126765552591	3.07729457913163	-0.87758643691290
C	0.80135325419597	3.22604831633060	-0.06940220988204
H	-3.10394476018807	2.56297063743215	-0.54635389547337
C	-1.34463994602328	4.35347121565482	-0.80479680085303
H	1.95428379498143	4.22376604339550	-1.59743372575805
H	2.52963538433333	4.52003859153801	0.06041768664442
C	4.83191583763660	0.51833535888307	-0.45585110113131
H	3.23413412555409	-2.85173452622420	0.66506881335133
C	5.62395926585296	-0.61990112468719	-0.35828917826537
H	-5.02936445862107	1.55334467385991	0.09724376368566
H	5.66940579183298	-2.73549906330145	0.13328373802426
H	-2.86766486313387	-2.92620042683791	0.38464383971959
H	5.28241202039771	1.47085071615058	-0.74541382860445
H	-6.41394567241416	-0.44943636632326	0.58274751111414
H	6.69068555804720	-0.56507839552127	-0.58364158638786
H	3.36860957582126	2.62257255472037	-0.45374250212900
H	-5.32618961020651	-2.70282602942845	0.71565940639090
H	-0.22211282390324	2.89703327794550	-1.92916748038733
C	-0.51701078744495	5.55535792292472	-1.27764468762023
H	-2.24375499393495	4.25421065279942	-1.43100375385859
H	-1.67781987396267	4.50582469299867	0.23653311217601
C	0.78851494123559	5.69241478784446	-0.49136860802266
H	-0.28733404308317	5.43537186086232	-2.35094062232205
H	-1.11976223425021	6.47177589678361	-1.18151417800147
C	1.62708308366477	4.41004182597610	-0.55989346200112
H	1.38117765143932	6.53728292272242	-0.87512416994700

H	0.55958253415188	5.91630297447942	0.56532267594307
H	0.51418874371610	3.40536345376366	0.98258213225950
N	0.47357904695796	-0.00484625971224	-1.94165968653969
H	-0.36986705070372	0.17267290254762	-2.49605902247605
H	1.22781593057114	0.53006035239487	-2.38273987689698
H	0.69947565026838	-0.99683919065746	-2.06606262041081
N	-0.37298299872521	1.00499609964059	2.95656148739569
H	-1.00972835791758	0.61310562434993	3.67948792037656

[1][INT6][0]

Ru	0.14325753571762	0.41543247688870	0.07237353619998
N	-0.02016001679129	0.73752750171582	1.93381064077739
C	-3.19713274648023	0.51525599549744	0.03945920725729
C	-4.60565365136488	0.65324833541063	0.15971192993484
C	-5.43650099598890	-0.40761321418562	0.47848705098550
C	-4.85691497229777	-1.67889496320646	0.67542315389459
C	-3.48980029835135	-1.86000021098390	0.55518964745368
C	-2.59779189699606	-0.78770684851375	0.23932340860031
C	-2.47755271567824	1.71787445047881	-0.32663969810966
N	-1.19264317677338	1.85327136044821	-0.38170551774285
O	-1.33205892359792	-1.05724764003631	0.14316174256571
N	1.47820815982296	1.90915687400659	-0.11719689878506
C	2.76059115400303	1.75888045917527	-0.18473155041969
C	3.48521791118351	0.50938513526340	-0.07630794906323
O	1.62362570726235	-1.02811865055396	0.31233236254529
C	2.89226207875544	-0.78851032721130	0.17079051261373
C	5.16462816334492	-1.75142642998972	0.13718591725048
C	3.79417897863369	-1.89421434642095	0.26778995127579
C	-0.52845021731610	3.07795759143657	-0.87174134389286
C	0.81252360347966	3.22579279680905	-0.11836591695640
H	-3.11003178093883	2.57758702066516	-0.58087814253955
C	-1.34467421201975	4.36799153539367	-0.79758097664707
H	1.91752752257631	4.18631415802258	-1.70554859441136
H	2.55153384265266	4.52189087973603	-0.08175956677190
C	4.89676465034394	0.60998327440548	-0.20317849403691
H	3.34920011776888	-2.87518669873842	0.45421503608195
C	5.73803926160635	-0.48476583304495	-0.10192345362802
H	-5.03564198390661	1.64588778221903	-0.00727729477930
H	5.80704206110009	-2.63311023467427	0.22282148845276
H	-3.03982392891744	-2.84473804110085	0.70678146328879
H	5.32088088797882	1.60271542122216	-0.38395275317242
H	-6.51533564674716	-0.26424232716946	0.57114793414251
H	6.81946698339789	-0.36974567895406	-0.20202973273167
H	3.38940700293699	2.64746899433841	-0.32109145100787
H	-5.49179161935506	-2.53400396219209	0.92671426283766
H	-0.27629030858007	2.88761939220752	-1.93244893138430
C	-0.53060084496435	5.55322667015634	-1.32997991403682
H	-2.27154966891798	4.26707220047232	-1.38205185353192
H	-1.63727492934419	4.54864429935399	0.25188300045605
C	0.80572747101724	5.69484744573357	-0.59883647506106
H	-0.34201710352659	5.40578246790399	-2.40846531755463
H	-1.12031083887584	6.47918585617697	-1.23539365500971
C	1.62461934301267	4.40025382001955	-0.66245997902596
H	1.39132068824317	6.52521973017544	-1.02515290675899

H	0.61723816999109	5.94976926873423	0.45939244529408
H	0.55921816802707	3.43846965824996	0.93764164354200
N	0.32849122407754	-0.01696661883436	-2.00119733608884
H	-0.57755978559466	-0.06133260901938	-2.47474463390695
H	0.90566293250330	0.64622395280410	-2.52536692368069
H	0.76643574826675	-0.93574008559943	-2.10965809878430
N	-0.12391710437929	0.93688791530814	3.03318902406965

^{2}[INT6]^{1}

Ru	0.13947172243551	0.40592216360800	0.06759274916433
N	-0.34661750158359	0.75593507357750	1.96254995907155
C	-3.16901100401227	0.53913795702499	0.05873890747901
C	-4.56549941114187	0.66383489217114	0.28011652531643
C	-5.35345662235126	-0.42665017926400	0.60330421770540
C	-4.75677929183260	-1.70218956782707	0.68774988229642
C	-3.40157365898062	-1.86872766272246	0.45459149864777
C	-2.56384445034655	-0.76345132675868	0.15069089992702
C	-2.46192391189408	1.73542366523219	-0.32899661279418
N	-1.17735253879830	1.84681598269613	-0.45866402053608
O	-1.29262420565273	-0.99428658206833	-0.07090752689666
N	1.45415805817762	1.92977680418808	-0.05069924112724
C	2.73340511661807	1.77770455737651	-0.19396325433767
C	3.44480984008562	0.52552019734691	-0.12478149295237
O	1.59409168734589	-0.85519641091879	0.63092894313041
C	2.85488842206747	-0.71878918111436	0.29566520625914
C	5.04641088386673	-1.78308012721772	0.07611864163821
C	3.70048332868319	-1.85546726335377	0.39562716969807
C	-0.50320956404150	3.07575546459102	-0.92100727140267
C	0.77774832929315	3.24013737005030	-0.06893043695711
H	-3.08866901239308	2.60888782703990	-0.54196087346668
C	-1.34253775204182	4.35034251103210	-0.90422810325448
H	1.98079203182654	4.22558701261969	-1.56646983280422
H	2.49189584449904	4.55135427764059	0.10521776130943
C	4.83256997962102	0.56320752976283	-0.42204878368804
H	3.24672502385415	-2.79292303447896	0.72407772915060
C	5.62726270108326	-0.56603771642579	-0.33848089882474
H	-5.01459373918645	1.65679855695129	0.19195767333939
H	5.66499269310644	-2.68156190616559	0.15105507158609
H	-2.93364996047249	-2.85390935396717	0.51143651555484
H	5.26887808024943	1.51922896761953	-0.72418425858126
H	-6.42349886647758	-0.30368436696543	0.78146175736715
H	6.69068832703742	-0.51483991932186	-0.58056207865306
H	3.35163159690452	2.66576568993949	-0.36716208089385
H	-5.36917893524994	-2.57298171857105	0.93710518049793
H	-0.17669878875997	2.87708184966815	-1.95841963411165
C	-0.50392011204269	5.54745497486992	-1.36858340601585
H	-2.21941933274627	4.23467145565202	-1.55900820197836
H	-1.71390462302802	4.52495314146331	0.12060528858071
C	0.76820545949240	5.70494962316443	-0.53284063807038
H	-0.22989270379603	5.40755132648897	-2.42916530374407
H	-1.11344923533182	6.46324758536444	-1.31607136402383
C	1.61281482319668	4.42516929959439	-0.54506538045587
H	1.37205707205730	6.54674633360658	-0.90660722550117

H	0.49451971681104	5.94677809212954	0.50930982398713
H	0.45162787106242	3.43089273516519	0.96969955276796
N	0.58564768629046	-0.03859992462569	-1.92395393461208
H	-0.26666360055339	-0.09501528368352	-2.48959064618138
H	1.20063833072707	0.63844413363421	-2.38505559388369
H	1.04373407143661	-0.95236862488894	-1.99743678042772
N	-0.65058987511416	0.94794309906976	3.01040392170141

^{1}[INT4']^{0}

Ru	0.14139557672484	0.43889741409456	0.02744249385852
N	0.00151099865541	0.65731504497270	1.94013342233234
C	-3.18497431828828	0.54573107013301	0.08290934881689
C	-4.59153253079036	0.68243356013871	0.22695239246645
C	-5.41766522903621	-0.37680739784767	0.56383721592013
C	-4.83629639134333	-1.64753732793597	0.75757086462995
C	-3.47147318466281	-1.82825051336496	0.61306582689210
C	-2.58490643612772	-0.75830262909835	0.27696256172623
C	-2.47306085780038	1.75019839991851	-0.29675165231592
N	-1.18669857556357	1.88133469704635	-0.37357637218255
O	-1.31928244797418	-1.03037146925212	0.16017430455551
N	1.47026153975087	1.92395815631624	-0.13449059525539
C	2.75647242133451	1.77048455492895	-0.16022824402276
C	3.47119237777761	0.51669471881362	-0.02534632888707
O	1.59057076828164	-1.01745365649403	0.27566800989289
C	2.86646953110582	-0.78211310960673	0.18997778620522
C	5.13508752455461	-1.75413127472077	0.24589650545877
C	3.75954521508098	-1.89157241136950	0.31738764448942
C	-0.53240144220941	3.10503214842949	-0.87744272656446
C	0.81979551650397	3.24868895066647	-0.14755389092658
H	-3.10962456080164	2.60998938127960	-0.53964869260390
C	-1.33976536338299	4.39996943403454	-0.79264670642956
H	1.90466082327462	4.17746805862986	-1.76593224851764
H	2.57184121242243	4.53173629794223	-0.16002024272127
C	4.88732535717294	0.61049829791689	-0.09431422589088
H	3.30335315655658	-2.87183598668890	0.47950524093947
C	5.72040985831585	-0.48781711528573	0.03685538911922
H	-5.02462650936555	1.67436318935712	0.06384640753777
H	5.77070463242553	-2.63826973279482	0.35402430254090
H	-3.01826472371114	-2.81194394416099	0.76222335019666
H	5.32289873808891	1.60196299530283	-0.25401304269780
H	-6.49459631325768	-0.23182316485358	0.67390589206867
H	6.80542076428272	-0.37511125537751	-0.01819172042079
H	3.39367744464078	2.65582622170275	-0.27591550353248
H	-5.46686839184427	-2.50116760066593	1.02416715386141
H	-0.30043081390713	2.91390692559720	-1.94244464149357
C	-0.52885727890803	5.57404156868493	-1.35487043557607
H	-2.28106573128992	4.30181942723559	-1.35415952298268
H	-1.60625703165374	4.59139951737770	0.26186518546209
C	0.82663983246595	5.71184595510831	-0.65858897690142
H	-0.36812125712979	5.41464835438259	-2.43621029398758
H	-1.10775924228897	6.50636110437636	-1.25528872822408
C	1.63306858294808	4.40893980342293	-0.72077786604864
H	1.40925103050169	6.53050650825053	-1.11112436314806

H	0.66645108125562	5.98477871873945	0.39990549061921
H	0.58457853502245	3.48289800485850	0.90911034895996
N	0.31911871887577	0.03072930972693	-2.10226438076625
H	-0.58889647884557	-0.12876864585485	-2.54535541452414
H	0.78700386520277	0.75873502146003	-2.64805265615212
H	0.86235409182653	-0.82600893841938	-2.23364737016926
N	-0.08310662440250	-0.23856992441039	2.82971278491990
H	-0.07698074701013	-1.14098666166915	2.32427917974391
H	-0.00829671345420	1.59737994902574	2.36701773972934

^{2}[INT4']$^{+}$

Ru	0.10955061376734	0.40490930445138	-0.00126235966190
N	-0.33499240934630	0.73123491243085	1.93178631549556
C	-3.18604838292004	0.56606170873803	0.08119400231830
C	-4.57371989418052	0.70262131172213	0.35077175189306
C	-5.36728062485046	-0.38541315817639	0.66647503752842
C	-4.78632635746964	-1.67120981106466	0.69436615226434
C	-3.44152273927041	-1.84849039363629	0.41488263592736
C	-2.59490716627039	-0.74678532124026	0.11773639186954
C	-2.47632986357471	1.76439866283685	-0.29641364085615
N	-1.19264167242938	1.86070632959698	-0.46241804132064
O	-1.33366316994054	-0.98624792342145	-0.14094138900699
N	1.44185574748384	1.91504821642249	-0.08958688365043
C	2.72353242005220	1.75743904204498	-0.18592289154131
C	3.42676195635832	0.49950193087759	-0.08726327333771
O	1.54306359415919	-0.86246483178283	0.62267409794094
C	2.81974468156866	-0.73522907389929	0.32864511490661
C	5.01253672207581	-1.81139705113141	0.19399388922308
C	3.65525142851455	-1.87254823347278	0.47043892009318
C	-0.51730142989044	3.08963763102925	-0.92445583125131
C	0.77935423744494	3.23287604096725	-0.09459880919743
H	-3.09840769022468	2.65003369338281	-0.46901732669976
C	-1.34255490187905	4.37264366758660	-0.88157504334093
H	1.97211678286168	4.21543309838477	-1.60184328063591
H	2.50897842400387	4.52739592232421	0.06378071572997
C	4.82236559889612	0.52752504662215	-0.34256393804747
H	3.18689680197945	-2.80341874711642	0.79765088376567
C	5.61063912426409	-0.60377944142707	-0.21934329495149
H	-5.01065090229521	1.70405567077141	0.30616383008165
H	5.62376264110006	-2.71161589187207	0.30188379634469
H	-2.98667340184613	-2.84142407849630	0.42949536859576
H	5.27210526593143	1.47802364123718	-0.64297432823819
H	-6.42943465970721	-0.25326229699554	0.88198899972947
H	6.68129201862957	-0.55927147745635	-0.42890068989689
H	3.35396743792952	2.64162315206271	-0.33431504900704
H	-5.40330484161241	-2.54063779879576	0.93749680443692
H	-0.21261585752328	2.89675014520820	-1.96936808182845
C	-0.49984459414324	5.56368333529070	-1.35429357208900
H	-2.23398664136644	4.27144485397251	-1.51903706561529
H	-1.69176540057167	4.54453817040373	0.15158938413323
C	0.78930512753275	5.70195336000018	-0.54144893624603
H	-0.24707925751025	5.42730894900214	-2.42067997343522
H	-1.09754041000401	6.48622885754601	-1.28507801944044
C	1.61966564886813	4.41308575520692	-0.57449763689931
H	1.39545831550181	6.53947980084533	-0.92133145141316

H	0.53692068121978	5.94116698952151	0.50676605119535
H	0.47089488148825	3.42075563985999	0.95065463562272
N	0.58612478059019	-0.04083210458124	-2.03438826164651
H	-0.25873988315239	-0.16919376110268	-2.59829438365075
H	1.14479138942187	0.67715785109922	-2.50396461002930
H	1.11493576678714	-0.91499662232314	-2.10531559106001
N	0.11707147973695	0.16478899440064	2.93721258304440
H	0.81299874066174	-0.53292507004656	2.60391412103449
H	-1.03536015685051	1.45336140219157	2.17507217082035

^{1}[INT4']$^{2+}$

Ru	0.11534044310936	0.41167600469108	-0.02540416638288
N	-0.35501663997168	0.73071870871009	1.94688458400502
C	-3.15961928794458	0.59656292448650	0.16954460488097
C	-4.53917987945356	0.70467348824638	0.47391183891575
C	-5.31344470676552	-0.41921524324603	0.69851411549350
C	-4.73865878384361	-1.70726123274401	0.57905547941137
C	-3.40671701960426	-1.85998357444346	0.24686773835147
C	-2.57376550713271	-0.72191269713810	0.07533634377047
C	-2.45625868523949	1.79452237569405	-0.18489505685370
N	-1.17818334727193	1.86482065293338	-0.44162573959242
O	-1.31544362728105	-0.91489202430244	-0.20584487679070
N	1.43721804670508	1.90921198703184	-0.13449213131180
C	2.71496073130944	1.74610725624094	-0.31675903932278
C	3.41276822983077	0.49688028834652	-0.19010883445552
O	1.52836734967947	-0.77335618610217	0.62941225081729
C	2.80625036566548	-0.69354685137729	0.35159265921778
C	4.97424452832687	-1.79128240687260	0.34631356958213
C	3.62467152786093	-1.81347391495003	0.64696796405766
C	-0.52414059114406	3.09873524062638	-0.91923622809815
C	0.79275547222319	3.23520959676122	-0.11682774111778
H	-3.06466608433363	2.69896225177651	-0.28778688245283
C	-1.34686335732859	4.38062183677938	-0.84962070538259
H	1.95503637459200	4.21221074613923	-1.65084843654265
H	2.53159594334622	4.51801176851477	0.00540312251668
C	4.80384177592246	0.48861703244735	-0.45137997673721
H	3.14697225591132	-2.69287818720457	1.08231334405613
C	5.57206849523396	-0.63810227568214	-0.21101443710936
H	-4.98456304181244	1.70098574227165	0.52429025500807
H	5.58625241588484	-2.67354645664290	0.54854897588418
H	-2.94789102183977	-2.84508191417846	0.14654546462184
H	5.26587431424825	1.39909166016121	-0.84068531507180
H	-6.37104860494133	-0.31546660001180	0.94841243921022
H	6.64135778896691	-0.62977606871642	-0.43084296743688
H	3.33483580456145	2.62395828074796	-0.52659760669935
H	-5.35771292525750	-2.59191814492057	0.74641545200707
H	-0.24604856692337	2.90555332987609	-1.97045576973814
C	-0.50734398506866	5.56714775420242	-1.34117704607581
H	-2.25215282809321	4.28335780619296	-1.46763465910436
H	-1.66826870181958	4.55249289546490	0.19219524739745
C	0.80095695867007	5.69909595176673	-0.55865053535787
H	-0.28193120141798	5.43064965032859	-2.41339184519245
H	-1.09949414751940	6.49137986325758	-1.25544840679258
C	1.62907228328329	4.40885615933280	-0.61491444350734
H	1.40097690868626	6.53410463363695	-0.95235675253485

H	0.57581816324619	5.93670833102057	0.49582966934478
H	0.51115069800857	3.41793681149508	0.93580077294089
N	0.60214249584284	-0.03452776614868	-2.04471451327561
H	-0.24307027436986	-0.25266498114847	-2.58185000194924
H	1.08059092908090	0.71646876413342	-2.55148607341408
H	1.20318178454432	-0.86222619112068	-2.11367571428208
N	0.00438193187875	0.08538015929123	2.93303035783284
H	0.63865710220769	-0.67371983307924	2.61110415495019
H	-0.98060830244912	1.51585259742410	2.20097549830910

[1][INT2b]$^{2+}$

Ru	0.11067510211500	-0.26855362630358	0.62188407948043
N	-0.37166277165350	-0.61493253029928	2.11023771080823
C	-3.03427568673661	-0.03235301783354	-0.36774683846302
C	-4.44019365670301	0.05252441473665	-0.55710856904856
C	-5.19344802836998	-1.05584537188659	-0.89622679887495
C	-4.54082725591400	-2.29097328907001	-1.08500692661365
C	-3.16763644176721	-2.40726909287210	-0.92655742627352
C	-2.37595927986172	-1.29468363209657	-0.55201443679596
C	-2.34200507005964	1.19645622029221	-0.09820000808651
N	-1.09564608993319	1.32138635407883	0.23502710501803
O	-1.07859975132807	-1.48971220395280	-0.42448202678522
N	1.44188768551732	1.20691193806015	1.05022269993442
C	2.72314648535116	1.03647083209936	1.11060521621276
C	3.43350476977980	-0.20295093859494	0.96664668894691
O	1.54578288698153	-1.64097801272499	0.37731893961761
C	2.82306052647569	-1.46116319218476	0.64345960525282
C	5.01904925085799	-2.51681251028274	0.83787540512662
C	3.65763933789796	-2.60376690373263	0.58714560146351
C	-0.39524881384172	2.62465289484367	0.30391946972038
C	0.76248950322848	2.49120883969694	1.30453069994868
H	-2.93522455721646	2.11170277123115	-0.21096456444738
C	-1.26798613991640	3.82892173406503	0.65597759827934
H	2.10162541894876	3.86225834883821	0.30807976451693
H	2.44818415180270	3.64040364576819	2.03621993720522
C	4.83558264695079	-0.14855736478940	1.19846834819743
H	3.18880634448354	-3.55920791417124	0.34466323766770
C	5.62482605266521	-1.28194260017955	1.14587466276253
H	-4.92058121301120	1.02556883240563	-0.42555176857873
H	5.62774063697065	-3.42382934854592	0.79364391632142
H	-2.66009737516441	-3.36216230805608	-1.07678405518060
H	5.27774216995508	0.82102985863310	1.44218379104546
H	-6.27420612744483	-0.97447037230103	-1.02571723313284
H	6.69725957928573	-1.22267380780450	1.33916625599868
H	3.34598982556112	1.91508589547730	1.31142007739047
H	-5.12209247657820	-3.17424189205666	-1.36311418513411
H	0.04753611722152	2.78469729829313	-0.69563189363928
C	-0.41248595617102	5.10249572736889	0.69919728944108
H	-2.06983991893136	3.95531301599983	-0.08608954291138
H	-1.74426777697830	3.64968008309344	1.63561165643078
C	0.77798775345073	4.96135468704839	1.64970036857916
H	-0.04378156297042	5.32013701142806	-0.31861252709029
H	-1.04388125643731	5.95407694024115	0.99735236067698
C	1.63727157560494	3.74017657800694	1.30197347748398
H	1.40234404738014	5.86778574648012	1.62558482309156

H	0.41394225079464	4.85574538252557	2.68625618083488
H	0.31326435271100	2.38727626718910	2.30672759059495
H	1.26647359708853	0.90133504331748	8.43132368837381
H	2.66321548015677	1.02721208852808	7.58056664718478
N	0.82280872872978	0.21802583530302	-1.62801887014231
H	1.09800107923469	-0.67458735298760	-2.04417648048390
H	0.07043419254903	0.58890182813965	-2.21306045774372
H	1.61871100046187	0.84952378285913	-1.74316448907069
Ru	0.16023006972728	1.36156806954198	5.38314173831376
N	-0.71687846209349	1.30625278487195	4.05941703835161
C	-2.05502127567063	-0.02546416752505	7.52583790220816
C	-3.06975905621774	-0.65997043471947	8.29395558597516
C	-4.06908946250339	0.06468263355729	8.91527261216600
C	-4.06532098462993	1.46966146991794	8.79825229185953
C	-3.08328215716617	2.12524187493378	8.07043834850009
C	-2.05838958900505	1.40616971610222	7.40987965567970
C	-1.03798433046979	-0.87869763227940	6.98021816860522
N	-0.08415670493017	-0.54966164794709	6.15879108151397
O	-1.15231418777568	2.11276307875087	6.75828169502344
N	1.91655139625441	0.48956002357204	4.71007182676776
C	3.00944374369278	1.13411392179519	4.44279889417613
C	3.20564591630119	2.55780633867162	4.47446470873705
O	1.00248103559676	3.21862275843923	5.32220313637062
C	2.21478005284626	3.51250128321006	4.88331813452636
C	3.80164732606178	5.30138865909991	4.38506213629333
C	2.55548084613989	4.88477843445214	4.82996858851662
C	1.04182637957893	-1.48480704112465	5.85465348266745
C	1.76944009775970	-0.98111906872562	4.59752832316607
H	-1.06468141734081	-1.92037756270968	7.32166476530744
C	0.63876312942812	-2.94822667092932	5.66718049402805
H	3.73766624014886	-1.66383321958993	5.17723583375486
H	3.53648124745121	-1.41192558060281	3.42755564165332
C	4.47708887766495	3.02164691688886	4.03874832648967
H	1.79902936171485	5.60585045023004	5.14450205390738
C	4.77859057566980	4.36958868331717	3.98179898652566
H	-3.04296155989960	-1.74914754841694	8.38260948828430
H	4.02364003491614	6.37125837605625	4.34979169110890
H	-3.07434610923035	3.21357774787107	7.98472326598399
H	5.21662598861915	2.27838148342795	3.72977574580341
H	-4.84447401245809	-0.44090580816585	9.49360937765115
H	5.75646058173779	4.70614058142424	3.63320376477709
H	3.88327425398170	0.54435356744201	4.14467148674264
H	-4.84484387222580	2.05705808159778	9.29100391321871
H	1.74127864820586	-1.40089556726476	6.70503232003481
C	1.87955306314642	-3.79578158109077	5.35179657793909

H	0.15214448599910	-3.33831172173355	6.57288575423809
H	-0.09155281038328	-3.01076482787579	4.84219747276868
C	2.65857353895529	-3.25722116052671	4.14880979656142
H	2.53858527696566	-3.80789953445103	6.23772858135990
H	1.56959916039272	-4.83715042558246	5.17217375125498
C	3.03673594339723	-1.78221713595525	4.33295325585131
H	3.56985578529456	-3.85201242937207	3.98213521043754
H	2.04560862120810	-3.35189708805369	3.23563695056593
H	1.08324858490733	-1.12921727913393	3.74640630640730
N	1.71554742990646	1.40047281166660	7.66094430376741
H	1.80623295510581	2.37472277361543	7.95513373904645

^{1}[TS1b]$^{2+}$

Ru	0.01468520732041	-0.01963187582113	1.00647596981263
N	-0.40189833561588	-0.14182146770564	2.61469366210608
C	-3.18042660286776	0.21176552146219	0.15419912131932
C	-4.58157684776590	0.34221155232889	-0.04634156637290
C	-5.39137013554987	-0.76012911438364	-0.24511388511395
C	-4.80513616968328	-2.04281205187995	-0.27419751475192
C	-3.44036502133366	-2.20890359508427	-0.09846926009674
C	-2.59030340776374	-1.09888618232404	0.13734808335050
C	-2.42801295714210	1.42980376974948	0.24705977343877
N	-1.15949498857848	1.53770886653322	0.50108930374719
O	-1.31120734671577	-1.34468408657539	0.30455650065651
N	1.40603581443411	1.42749998934813	1.19539841034071
C	2.68835692441812	1.22883894849052	1.19069138033023
C	3.36363440545950	-0.03363643892728	1.11909120052213
O	1.40869184339140	-1.48578071375620	0.96993921742874
C	2.70652069678862	-1.30806969230764	1.00997719586965
C	4.90261582313797	-2.38345072578314	0.98769391467362
C	3.52155358163167	-2.46735676885068	0.93271329359859
C	-0.41297688554401	2.81155697403657	0.37228732707599
C	0.78432789619904	2.75863892477884	1.33413053968536
H	-2.98977378268444	2.35019983188950	0.05008619040023
C	-1.22433042520181	4.08677059001473	0.59074836345466
H	2.12625828483523	3.93374570377017	0.11799454433109
H	2.53885368880783	3.92423815209085	1.84693764998373
C	4.78570931935947	0.01189002080152	1.16178333423617
H	3.01728607902038	-3.43063037837625	0.83802952684059
C	5.55187863183325	-1.13564600321754	1.10799365261219
H	-5.01085022027543	1.34744401394706	-0.04542993890656
H	5.49547128459414	-3.30051413168215	0.93661741213351
H	-2.98193331207785	-3.19934466826739	-0.12609395948925
H	5.26560077745295	0.99011358007284	1.24743274419329
H	-6.46642596726437	-0.63974456581731	-0.39050326325582
H	6.64099104791911	-1.07963222188806	1.15095765434462
H	3.33898121278134	2.10717422327619	1.25245228742851
H	-5.43260303430940	-2.92196007643039	-0.44342281209672
H	-0.00641382514170	2.81937300723036	-0.65550663528848
C	-0.31936175539964	5.31664582465958	0.43312330874776
H	-2.05032265245832	4.14818600665609	-0.13277059743948
H	-1.66669770866214	4.06052844170747	1.60161580381451
C	0.90335010986631	5.25114861516741	1.35077528371542
H	0.01568965120820	5.38161528603005	-0.61700430622055
H	-0.90455183607021	6.22688929982108	0.63715168357093
C	1.70140434772118	3.96054666663657	1.13647406033618
H	1.55829755232670	6.12003686951104	1.18190270689223

H	0.57930158392754	5.29655186501417	2.40487932911772
H	0.38153795858166	2.79303805710093	2.36201426924518
H	1.33402483777173	0.76105147829179	8.28885011771729
H	2.66381182343794	1.31198444105093	7.48888723653219
N	0.60591750709119	0.00714344899895	-1.23020940328711
H	0.73544377419929	-0.96326435233125	-1.52604039433963
H	-0.12110097512077	0.40306671396052	-1.83137807178161
H	1.47220111592885	0.50274070278915	-1.45402799257972
Ru	0.30395609456070	1.15279684972662	5.29252294125624
N	-0.47855670220904	1.09106260800089	3.84318913041999
C	-2.23333301444041	-0.35451671888385	6.89726158523546
C	-3.38338218237674	-1.03736385850511	7.37874748051587
C	-4.49045751899656	-0.35271957591772	7.84365221448602
C	-4.46217037731600	1.05713552827016	7.86884020749467
C	-3.34982499479645	1.75864921164461	7.42962680825903
C	-2.21562670014839	1.08354900867852	6.91596526316207
C	-1.11037679895756	-1.16644933044871	6.52019983995682
N	-0.01010158212169	-0.76290495134804	5.95033482832666
O	-1.20409809822389	1.82425990170804	6.50559965673600
N	2.09022425148255	0.33798641938233	4.69791113695250
C	3.16642610379260	1.01253586676835	4.40618863313141
C	3.33577243643987	2.43519776849820	4.35616296014989
O	1.10931974926368	3.15701767604984	5.07184320996015
C	2.32607412497822	3.41714314345069	4.67826551289710
C	3.94468373900805	5.17777508149351	4.13938487370759
C	2.68703321685576	4.78993735504774	4.56224054141937
C	1.18925153191390	-1.64288518097969	5.81598326477480
C	2.02409191086956	-1.14677300142278	4.62217774975672
H	-1.18929037518359	-2.23052109877426	6.76990490718637
C	0.90630900781219	-3.13808425410547	5.68823657856410
H	3.94911560681337	-1.65898830764362	5.45258134719847
H	3.93820136532692	-1.54478006126685	3.67997177494382
C	4.62337527805280	2.88130516861477	3.93493131642622
H	1.92328951860938	5.52862723034009	4.81211388637658
C	4.93195964075560	4.21989547613666	3.81444784232415
H	-3.37486304433559	-2.13042486986974	7.37318950270007
H	4.17475191710337	6.24334092619004	4.05507171852580
H	-3.32253822378145	2.84978984919379	7.45243551027856
H	5.37254523458646	2.12303820125176	3.69294257539250
H	-5.36961919859194	-0.89430307760329	8.19731904752377
H	5.92166983648748	4.53510655160880	3.47938673907104
H	4.05991344013850	0.43451577938369	4.14882755016651
H	-5.32765666546050	1.61011223411318	8.24364457280310
H	1.78511690032923	-1.46489722492011	6.72904282239243
C	2.22946524634884	-3.90850079450271	5.57896143962352

H	0.34708828844684	-3.50287973427507	6.56179909406964
H	0.28189913780807	-3.30950437756451	4.79448570155488
C	3.10099392775211	-3.39044620153377	4.43374743114965
H	2.77880436520872	-3.80900758470882	6.53140803550799
H	2.01571830736030	-4.98029331237292	5.44430692671734
C	3.35660474358138	-1.88307013851374	4.54869749809456
H	4.06453936030151	-3.92311676905488	4.41049220637583
H	2.60361799400237	-3.59437659026408	3.47016214820996
H	1.44989850631662	-1.36786984750210	3.70464074511216
N	1.65265804400260	1.42358684335706	7.57945992747658
H	1.48031806241128	2.36337390726456	7.94025086305008

[1][INT3b]$^{2+}$

Ru	0.05680471013359	0.23701060924405	0.75518197461936
N	-0.39824847555315	0.36930514348322	2.66674895047552
C	-3.20842344827225	0.57612004084577	0.69580648559607
C	-4.59465595581879	0.74693751317602	0.94628566473893
C	-5.44823397050294	-0.33329119522531	1.08670464466718
C	-4.93211279266282	-1.63820383289468	0.94536385357272
C	-3.58898435426693	-1.84534474729043	0.67425920477902
C	-2.68473490475028	-0.75806885058298	0.56101669439267
C	-2.42726920585731	1.77451854579916	0.50559031825382
N	-1.13662789154001	1.82072576921299	0.40040048372361
O	-1.42452113905171	-1.02301845763499	0.30134455335203
N	1.45467165525795	1.67804408489799	1.01042048714128
C	2.73073636630924	1.46820453836957	1.08546133526784
C	3.38041803826210	0.18138817885738	1.09167004067591
O	1.38496580333451	-1.18866406261561	1.25444760639797
C	2.68758862331094	-1.07573808080285	1.17143190680840
C	4.84765886588457	-2.22132638689402	1.18969634630923
C	3.46381308447200	-2.26365172541061	1.21776199558514
C	-0.36928778347227	3.05691831520294	0.16591977315738
C	0.85248823951425	3.02238226317777	1.11283124747600
H	-2.99494214806777	2.70931992349528	0.43570152888459
C	-1.13734448068968	4.36718309139419	0.31298258037300
H	2.20092183712018	4.09491806547950	-0.18332738591925
H	2.63058522913448	4.18639743766587	1.53944520055524
C	4.79985746474696	0.18445634321177	1.09003905935661
H	2.92783910327738	-3.21264824644147	1.28074787615267
C	5.53199059681097	-0.98889247328927	1.13255376681676
H	-4.98182971421533	1.76581215599850	1.03224005792315
H	5.41327892582124	-3.15623930015499	1.22361198374627
H	-3.18432404986343	-2.85294079383097	0.55766170535814
H	5.31162487754776	1.14973255797213	1.05659280121962
H	-6.50879245346083	-0.17765988086863	1.29399915937244
H	6.62328066012408	-0.96196110229007	1.12467905384666
H	3.39633404009657	2.33311116244459	1.17498037560750
H	-5.59797433458272	-2.49936359030055	1.04752773712765
H	0.01845682431908	2.98602959964374	-0.86669123030664
C	-0.20472613847825	5.55844093020310	0.05990520931110
H	-1.97961577039642	4.39522456731572	-0.39523200449081
H	-1.55892374702318	4.42720168130641	1.33154665378928
C	1.02472320286213	5.51825479963710	0.97008812479829
H	0.12057701925454	5.54266320892223	-0.99521704674573
H	-0.76157492981654	6.49722777605854	0.20690665463630
C	1.78753883449347	4.19583010034723	0.83533263158884
H	1.70081128091747	6.35677965105884	0.74013722970575

H	0.71029559174894	5.64332105632426	2.02102126224955
H	0.46905876732822	3.10825813299913	2.14594078072768
H	0.93389312727975	0.97688505798392	8.13886075552298
H	2.40374354674550	0.91946300801537	7.40306201109908
N	0.66839882218104	0.07460337216957	-1.23653266427914
H	1.10073783473666	-0.83827716684421	-1.40922058083377
H	-0.12973188772444	0.14464079229768	-1.87523667094480
H	1.34528216464475	0.78429527352033	-1.53174456511953
Ru	0.47846449805879	0.87084867518570	5.54184566574442
N	-0.32051613672835	0.51526802883064	3.77094822677242
C	-2.27474026043189	-0.71226671822786	6.40425069867875
C	-3.50106706953152	-1.41665476033573	6.52173853523752
C	-4.70537183166050	-0.75857051805400	6.69936766713689
C	-4.71105342825866	0.64855998843597	6.79723860577302
C	-3.53163046251729	1.37046996950515	6.71271957354734
C	-2.28711202587306	0.72407273898649	6.49643222949081
C	-1.07361096162550	-1.50399410893619	6.29341831354256
N	0.11425498896881	-1.04048663106475	6.06175812852726
O	-1.20710072933234	1.46715079685149	6.43217268902764
N	2.20656325786350	0.08123074341883	4.85076338770929
C	3.23548005816023	0.77050549962829	4.46928449659146
C	3.30917539939055	2.20669231991882	4.37256600049229
O	0.97709893039868	2.69254337439043	4.84216995352400
C	2.19077033344107	3.09253508238889	4.55729971421729
C	3.64972094194938	4.98106856513536	4.01979276540669
C	2.40514585222771	4.48394159908038	4.36702498850226
C	1.33064514521612	-1.87167135867811	6.01809442326794
C	2.16846625480413	-1.39476666537282	4.81011748367408
H	-1.19815605205402	-2.58316372437063	6.43822057740022
C	1.11635569692553	-3.38173878805073	5.97465007642865
H	4.10144360378235	-1.81249695777499	5.67262233173583
H	4.09544078606957	-1.78728201169368	3.89523416743338
C	4.56431210370210	2.75378917209729	3.99627051371338
H	1.54877606816993	5.14747619890116	4.50272054277634
C	4.74617788444522	4.11438694583134	3.82521249626870
H	-3.47676710709471	-2.50834951193650	6.46653798804414
H	3.77790468072755	6.05830116437866	3.88445757107786
H	-3.52710204626953	2.45953359265317	6.79393817530640
H	5.39807377543981	2.06521431803954	3.83621296988188
H	-5.63798061608308	-1.32123035536112	6.77409744169759
H	5.72190345328246	4.51174015158200	3.53941989060882
H	4.13813309625673	0.23154676954231	4.16266249641514
H	-5.65562564551078	1.17885996053181	6.94546217319431
H	1.90395711721586	-1.62017388716027	6.92926600814984
C	2.46696284060192	-4.10649438578977	5.91480308186951

H	0.55603380884375	-3.71115767646682	6.86303425948027
H	0.51126231934250	-3.63627292089404	5.08710560764529
C	3.31943469302830	-3.61883387312051	4.74137401172889
H	3.01139925735050	-3.93246587603029	6.85966811262733
H	2.29685704727522	-5.19220995867554	5.84183618249367
C	3.52149419680237	-2.09990024105808	4.77822643800520
H	4.30040980567306	-4.11971470019071	4.74369516187502
H	2.82742124296401	-3.89020719131597	3.79107471080507
H	1.60832668302389	-1.66041015976058	3.89486491869516
N	1.46233218900028	1.31590771812342	7.32915631236759
H	1.55623080042838	2.32986475106841	7.44456661703003

^{3}[INT2b]$^{2+}$

Ru	0.28428309191173	-0.23001393232046	0.61701227393000
N	-0.39757566381148	-0.51660728482940	2.15783945230171
C	-2.95430639453759	-0.04769219530016	0.23452361724040
C	-4.36467351394285	-0.02059887111194	0.36850577846184
C	-5.13189982535610	-1.15536027892435	0.16618465784257
C	-4.50180120187291	-2.35498328070384	-0.22477022958695
C	-3.12805014240727	-2.41274805026969	-0.40043071749023
C	-2.31739099017138	-1.27960955405691	-0.14838580804434
C	-2.25415075836456	1.21014631614027	0.33780549656397
N	-0.96832757388403	1.34743509285897	0.37485136389717
O	-1.01749074834164	-1.39445084076084	-0.32333484423192
N	1.60261267712459	1.24845274254522	1.05575773267409
C	2.89177365034781	1.12906588733180	0.96207386006857
C	3.62435829308391	-0.09100136970241	0.76580713319851
O	1.73002507204923	-1.59256355417727	0.83124253400157
C	3.01772585272358	-1.39512077698143	0.72653020067600
C	5.23615017935055	-2.38979072562995	0.54511939717829
C	3.86127482652971	-2.53149161625380	0.62413255881630
C	-0.26970388520548	2.64333568001112	0.35919774246922
C	0.92555248845860	2.52947260638864	1.32835306612889
H	-2.88090647563099	2.10905402868868	0.34706549366803
C	-1.12553618113533	3.86168164333075	0.69548336139606
H	2.23082475290829	3.89352950621202	0.27908537593506
H	2.61921535399187	3.69791007718953	2.00194440083136
C	5.03760249870372	0.00971065520410	0.70387004983262
H	3.38791263647959	-3.51456369274058	0.60610288313122
C	5.83777170454112	-1.11332528077637	0.58700055911534
H	-4.84128906505385	0.92589553253127	0.63624206046215
H	5.86244443129961	-3.28118549257942	0.45714552951294
H	-2.63223485569226	-3.33476639455508	-0.70977919237420
H	5.48878216412804	1.00362946386693	0.75714225108706
H	-6.21576054671032	-1.11842238128027	0.29106449827552
H	6.92362445030686	-1.01452568548233	0.53637410118905
H	3.50108615804883	2.03402332557373	1.06069680536302
H	-5.10247802512629	-3.25169191225558	-0.39758881098092
H	0.14321414688568	2.75693455884447	-0.65946640676666
C	-0.26695412047292	5.13265179138597	0.68885853174145
H	-1.94488383403247	3.96321094652116	-0.03203869305875
H	-1.57886111657698	3.71482732358481	1.69127583416185
C	0.93498145539763	5.00857820162060	1.62698889918404
H	0.08841688223368	5.32256147856013	-0.33923584003581
H	-0.89082881153797	5.99419872870813	0.97379323038839
C	1.79099030569263	3.78337760831754	1.28547361104123
H	1.55850568508374	5.91464779734251	1.57951707342238

H	0.58442692966481	4.91962975821033	2.66978000594317
H	0.50054445402547	2.42876160216856	2.33980716815480
H	1.11805696155651	0.67376164533837	8.35576254906586
H	2.52671145757642	0.90662291800633	7.54643589750915
N	1.03689523271618	0.02926733012635	-1.57327878487040
H	1.28956666614165	-0.87803837083979	-1.97254074866829
H	0.28181561013964	0.39694228979794	-2.15754489095176
H	1.83887200034267	0.64596852456318	-1.72331966478229
Ru	0.08446542947082	1.39628279907359	5.33212589738509
N	-0.77863114688040	1.49185452152692	3.99876327660594
C	-2.30072764401598	-0.05011541070543	7.23214770066361
C	-3.39139157760957	-0.69868593393542	7.87423673372102
C	-4.39913151666587	0.01664210865472	8.49231071821459
C	-4.32744160875090	1.42495597514225	8.50222591507687
C	-3.27075485481740	2.09257171401487	7.90165659242297
C	-2.23360083666870	1.38536170017777	7.24555693010889
C	-1.27948286199300	-0.90092918700230	6.69034254101308
N	-0.25876863011497	-0.54848945984700	5.96437615848934
O	-1.25530537386703	2.09859960201629	6.72098119729356
N	1.81701310524934	0.50274251645035	4.62905704322620
C	2.92108181955615	1.13056569862257	4.36502657558340
C	3.15824056148396	2.54468268876419	4.44942854902663
O	1.00621402200486	3.22081635829510	5.40372346468077
C	2.20856935582339	3.50503051836123	4.93674712662784
C	3.83133993143272	5.27084671719653	4.46920400508400
C	2.58796020641219	4.86898397424229	4.93414059729732
C	0.86426536522463	-1.49040023406538	5.67788344674104
C	1.64907004352755	-0.96086588862775	4.46794025130595
H	-1.36776330093293	-1.96315970843892	6.94694810814147
C	0.45521219266769	-2.94383524696683	5.43666755251076
H	3.58149955263293	-1.68126552626004	5.11322033106167
H	3.46337023652867	-1.38522039525258	3.36586770147883
C	4.43065558832602	2.99222198318499	3.99853693427520
H	1.86217958478413	5.59564278748996	5.30357372258698
C	4.76961330951966	4.33207251735655	3.99466634079860
H	-3.41587054679590	-1.79153599698958	7.86830113180472
H	4.08242273323491	6.33486446940013	4.47356685821296
H	-3.20997937243362	3.18258015237324	7.91497758607784
H	5.13810781801518	2.24391712571586	3.63166434099091
H	-5.23287316308834	-0.49889713533367	8.97239975641148
H	5.74569862536119	4.65786833659359	3.63117927087986
H	3.77227310320713	0.53194664606335	4.02201392322232
H	-5.11375549359412	2.00443744238840	8.99362587814417
H	1.52866622002051	-1.43822374921150	6.55860755114241
C	1.69949649834340	-3.79735696309718	5.15326007295531

H	-0.07092192929264	-3.35163711871722	6.31185847072345
H	-0.24065180976390	-2.97783904396452	4.58071773872949
C	2.53462967677388	-3.23818457258108	3.99890665578912
H	2.31919415468439	-3.83912590266058	6.06626211997995
H	1.38691049266351	-4.83051263314085	4.93468341518161
C	2.91645991726977	-1.77229682032663	4.23671838314242
H	3.44718040304804	-3.83786524703262	3.85829230566363
H	1.96048283768005	-3.30400070854926	3.05918104332826
H	0.99222695231055	-1.07757777204915	3.58712109580037
N	1.56809789409289	1.24743677800738	7.63985021892653
H	1.62762969635532	2.19567200420566	8.01542436745241

^{3}[TS2b]$^{2+}$

Ru	0.00169715018213	-0.23631273160469	0.93971945797649
N	-0.37440242312035	-0.58016868213372	2.47426175122967
C	-3.23433266247879	-0.03105436562816	0.28328249813516
C	-4.65237390778160	0.06252274986755	0.23297036319787
C	-5.44665900232792	-1.04635777299819	0.01219960329651
C	-4.82712145099805	-2.29699622310050	-0.19325359193660
C	-3.44737511858368	-2.42448814430562	-0.16821078664694
C	-2.60617932040228	-1.30928585011294	0.08136828495377
C	-2.51554327741718	1.20282316345379	0.43715269492862
N	-1.24152747428960	1.33549369286710	0.63645336402366
O	-1.30988403481094	-1.51518011834081	0.08180625722177
N	1.35536478386684	1.24665078174093	1.23746509782638
C	2.63925020753065	1.08361377344213	1.22187414920007
C	3.34880805389983	-0.15469792524293	1.06147342889077
O	1.43865534380813	-1.61900188464855	0.64742334257170
C	2.72801379556819	-1.42420150587113	0.80373377335475
C	4.94778381186018	-2.45385643771510	0.84472355669452
C	3.57377457023772	-2.55806238641870	0.69559172497453
C	-0.54146301229205	2.63995172129346	0.60105147384002
C	0.69851806880299	2.53896497836362	1.50179590712088
H	-3.11870396793677	2.11491870897503	0.35713397287211
C	-1.38179597006653	3.85626452732022	0.98868894924992
H	1.95034368842549	3.87697217708565	0.35775079528467
H	2.43677743286559	3.70859360644476	2.05833617681619
C	4.76350690401176	-0.08324435629125	1.18912838241314
H	3.09964605785281	-3.52191803080429	0.50102648627657
C	5.56039440130138	-1.20825009917003	1.09459691373685
H	-5.10796507380338	1.04608269446379	0.37580128782398
H	5.56229570561697	-3.35480015034306	0.76771204653672
H	-2.96644608617334	-3.39124343016680	-0.33079372078849
H	5.21147252579200	0.89429785417857	1.38649623181123
H	-6.53418427387937	-0.95559494335487	-0.01236910868625
H	6.64317944579742	-1.13493129858272	1.21011921160606
H	3.26868788030633	1.96927106279050	1.36115260048759
H	-5.44166774749839	-3.18246849325452	-0.37745362839205
H	-0.18790058596025	2.76705969847676	-0.43848185924742
C	-0.52831855453395	5.13019291232213	0.91962305521835
H	-2.24510835169653	3.95980861334872	0.31528484045470
H	-1.77062242719522	3.70776781609507	2.01106997103311
C	0.74076661295422	5.01979025141132	1.76800978621992
H	-0.24844929672147	5.31638198673178	-0.13218568630382
H	-1.13334495037115	5.99138674138617	1.24408599471157
C	1.56815164495072	3.78613709134520	1.38946957299198
H	1.35781353563814	5.92499464609685	1.65738744541817

H	0.46779658843168	4.95006639753774	2.83512555771736
H	0.32889290161142	2.46692298126693	2.54034964580302
H	1.23457338363906	0.84864380612615	8.18665232351180
H	2.64520298922742	1.25022525738276	7.45739871604334
N	0.50344527132922	0.13004214162969	-1.36580831625445
H	0.70289196325857	-0.78385094812202	-1.77816454685860
H	-0.27998047299832	0.52262997479709	-1.89283379244662
H	1.31394519681414	0.72164063725932	-1.56243825480021
Ru	0.57110946020273	1.34358927366311	5.41887104871134
N	-0.43679087783857	1.24029348207992	4.05838066473407
C	-2.19197452612927	-0.06573436007687	6.51102888495940
C	-3.43406554818473	-0.68993116568279	6.73809206560441
C	-4.57071246095389	0.03920664395108	7.07493226665645
C	-4.48489742664370	1.43920725610100	7.24553990089250
C	-3.28231447303148	2.09151890047508	7.06832820451783
C	-2.10775222171848	1.38124223173747	6.66250097319909
C	-1.06084450431938	-0.93870273043251	6.28371611650457
N	0.13221704172471	-0.55858081870009	5.96334701592611
O	-1.02606979591082	2.06177813215786	6.49225447056610
N	2.22674586171018	0.47452455287739	4.67106813517447
C	3.33937348678451	1.08600485305912	4.40499952027743
C	3.56453935781839	2.50589487603732	4.44122634244313
O	1.34487465537049	3.16409149554975	5.16913921708869
C	2.56669515911640	3.47119452133161	4.81071148340582
C	4.17581950586534	5.25331644872680	4.36986100575330
C	2.91289380793362	4.84647175584763	4.76614331931380
C	1.28485360333052	-1.46493558033776	5.81397773651633
C	2.07795206679963	-0.99163739421248	4.57456582320360
H	-1.25511544018552	-2.00816245506456	6.42254582091391
C	0.95443630645999	-2.95192066851765	5.72409514949733
H	4.02792275190769	-1.60841978779295	5.26601339084798
H	3.89376447692342	-1.47930166566305	3.49911857752947
C	4.84665935105077	2.96207564470395	4.03888967779983
H	2.14657711408190	5.57014332138699	5.04982229205688
C	5.15735121333535	4.30995988465331	3.99795150016866
H	-3.49484926811720	-1.77743308839865	6.65222720306316
H	4.41124418662240	6.32047637840346	4.34374044276102
H	-3.19043271866721	3.17157563146895	7.19695942307845
H	5.59194799520197	2.21680958192024	3.74902668020906
H	-5.52224067214018	-0.47378067786909	7.22716418524796
H	6.14881514123937	4.64003460350614	3.68255700794289
H	4.20110501433740	0.48154208817765	4.10552624361740
H	-5.37488857923658	2.00743579940005	7.52594800391500
H	1.92574324416344	-1.28977735644827	6.69679199159486
C	2.23750923329188	-3.76872525371475	5.52743861298966

H	0.43941800587309	-3.28124357358133	6.63944691672132
H	0.26766510875023	-3.11702066688961	4.87582073244006
C	3.02834177863154	-3.28935245238471	4.30903359383703
H	2.86559204912164	-3.67769543563504	6.43099864845063
H	1.97810775677688	-4.83395152241911	5.42495383357372
C	3.36166618178594	-1.79630219670053	4.40619400319365
H	3.96011594320089	-3.86574409189889	4.20071478713117
H	2.43813464473775	-3.46281995768003	3.39261529030901
H	1.43042887566057	-1.16731490477312	3.70001115979073
N	1.64519249515845	1.46556278697668	7.48140686933174
H	1.56192516586363	2.41770099339068	7.84565036542258

^{3}[INT3b]$^{2+}$

Ru	0.05463752304968	0.23536256006092	0.75600240604474
N	-0.39596674327171	0.36920748632024	2.66803275170200
C	-3.20664179744722	0.58934233649660	0.73677000287040
C	-4.58695538918161	0.76575988117944	1.01453499970963
C	-5.44096163214768	-0.31130628240642	1.17552014086037
C	-4.93229710513633	-1.61826261064798	1.02690295391352
C	-3.59573631311103	-1.83082097611839	0.72826872179855
C	-2.69062421342762	-0.74694529095570	0.59416034280331
C	-2.42372584122725	1.78428212137669	0.53141071109453
N	-1.13434603520844	1.82497692696960	0.40948205897753
O	-1.43639629082794	-1.01563256812069	0.30913786432995
N	1.45891594685650	1.67338517074597	1.00811254695143
C	2.73406380216439	1.45953945015760	1.08290119258988
C	3.38020223505593	0.17064347530582	1.08916506745180
O	1.38010793974259	-1.19166375417085	1.25369554434070
C	2.68343177738314	-1.08387713298530	1.16889202313287
C	4.83929221894870	-2.23685697918690	1.18671008909933
C	3.45514392763329	-2.27446666316648	1.21474504529818
C	-0.36352554764136	3.05734850707508	0.16806028699353
C	0.86139459835523	3.01994314867907	1.11082214230278
H	-2.98836307588937	2.72134041921050	0.46698178823344
C	-1.12589689612523	4.37082872714418	0.31479898889887
H	2.21119101265823	4.08435908662103	-0.19036030902249
H	2.64423910370933	4.17907313624599	1.53135937798606
C	4.79953427817086	0.16907703210175	1.08765434451245
H	2.91592749601933	-3.22163967939317	1.27751937831198
C	5.52775120036228	-1.00686695640895	1.12997637938957
H	-4.96881309452144	1.78616250816908	1.10607151244865
H	5.40168949104213	-3.17372871300506	1.22042722438034
H	-3.19703706758407	-2.84009699145855	0.60577596151784
H	5.31454356016077	1.13264785966061	1.05448770692745
H	-6.49644241040082	-0.15160724435480	1.40452259951539
H	6.61913380787465	-0.98360933564313	1.12225190913244
H	3.40230625495312	2.32242259463694	1.17241328946211
H	-5.59876474593199	-2.47687025942192	1.14536936597778
H	0.01973708248478	2.98219402169987	-0.86587838216381
C	-0.18947097462447	5.55745571048703	0.05431392917249
H	-1.97138361812047	4.39971520436389	-0.38954183554488
H	-1.54240438648171	4.43578002873326	1.33513982636626
C	1.04236756952385	5.51529457150416	0.96121632786535
H	0.13252078267974	5.53673150505716	-1.00174363067988
H	-0.74205524720350	6.49899890615948	0.19976308203738
C	1.79990199539091	4.18958068508905	0.82872020574871
H	1.72117380335052	6.35041134887004	0.72689743476032

H	0.73123517116907	5.64491841217473	2.01255065779598
H	0.48133650656058	3.10862865105767	2.14492035963303
H	0.92240231007692	0.97228479919852	8.14403855875594
H	2.39400190692045	0.91689859458130	7.41136089009678
N	0.65822009604233	0.07241233892111	-1.23788896203199
H	1.08797027392641	-0.84136010312360	-1.41243311792225
H	-0.14158813875843	0.14487801804255	-1.87419678556399
H	1.33615512621462	0.78052739909726	-1.53451202557245
Ru	0.47464708979022	0.87159463524915	5.54540433665569
N	-0.32009246687944	0.51668056366715	3.77189923083860
C	-2.27956784486667	-0.72627205538080	6.36460025607770
C	-3.50579276501474	-1.43521156768452	6.45196147791345
C	-4.71534913328748	-0.78187251288434	6.61050704327500
C	-4.72693172172674	0.62428997915779	6.72033863277583
C	-3.54805760206529	1.35027097867095	6.66591420878067
C	-2.29787834094725	0.70915506552695	6.46861359681650
C	-1.07391426526737	-1.51308343843549	6.26806982297384
N	0.11514472389883	-1.04374017226331	6.05492741688449
O	-1.21801977837722	1.45484393816666	6.43164810678208
N	2.20739405761499	0.08763650627211	4.85416751852170
C	3.23346312192784	0.78010394895089	4.47170681200773
C	3.30302942827186	2.21668181918510	4.37524023810585
O	0.97019529679104	2.69414193718399	4.84838313039096
C	2.18241910893047	3.09875022791503	4.56232656451336
C	3.63404788416977	4.99188403106177	4.02174260655050
C	2.39171332749808	4.49069871025153	4.37176657804897
C	1.33480223162481	-1.87001247126598	6.01621009757184
C	2.17403344010974	-1.38862211554807	4.81102076845970
H	-1.19623006788897	-2.59353318092785	6.40492032257179
C	1.12668763448373	-3.38081538208584	5.97127774642914
H	4.10752304608211	-1.79945088026115	5.67552510153977
H	4.10338945370659	-1.77428982481440	3.89811000619677
C	4.55557096910086	2.76760432806241	3.99624312538775
H	1.53353706429195	5.15152401861761	4.50927327171790
C	4.73278563287365	4.12886324585461	3.82475209012657
H	-3.47711070551997	-2.52631917437316	6.38770577406403
H	3.75831588243148	6.06951881467178	3.88594147726053
H	-3.54846310253173	2.43860881978691	6.75643700305523
H	5.39104920584573	2.08157174182078	3.83410385958069
H	-5.64771836344474	-1.34759204855123	6.66131517443196
H	5.70656743963558	4.52928041433544	3.53660191321806
H	4.13717867345678	0.24395648993672	4.16315858110110
H	-5.67583662519490	1.15076234290862	6.85384490741498
H	1.90379023007904	-1.61666568418240	6.92952510337274
C	2.48046845262740	-4.09985410569489	5.91511901237317

H	0.56457819678910	-3.71273162890932	6.85763819224001
H	0.52579590593860	-3.63764224287641	5.08153474934524
C	3.33333525314403	-3.60875509324158	4.74338820445116
H	3.02202145947667	-3.92348704565405	6.86121698637319
H	2.31515183811574	-5.18629012722482	5.84182017605976
C	3.52958136017108	-2.08904123165973	4.78052506672759
H	4.31624721199134	-4.10582983688383	4.74750707960882
H	2.84412568349575	-3.88205001558933	3.79223946250404
H	1.61653839301140	-1.65481145445917	3.89432564522831
N	1.45259583853595	1.31316089378924	7.33627266024126
H	1.54619601235202	2.32689875572836	7.45402592048312

^{1}[INT2]$^{+}$

Ru	0.10412208730667	0.70198033247888	0.84788618426965
N	0.03633921077776	1.18081937585936	2.37444000606464
C	-3.20106822729680	0.66919481354011	0.45952438769772
C	-4.62088550774626	0.73884591573465	0.45440166059498
C	-5.40187554793620	-0.35077828202566	0.79152607867221
C	-4.76861120853148	-1.56527224387582	1.12547641669276
C	-3.38602016994113	-1.67439126259893	1.12581359555832
C	-2.56174428676893	-0.56778363983809	0.80592819550124
C	-2.49570037266693	1.83696888869780	0.01013587317297
N	-1.21375651787353	2.02399325733409	0.03168680620646
O	-1.25967897607131	-0.76319246559407	0.82161867093880
N	1.45372629029053	2.09242430474959	0.22632125209713
C	2.71483644357444	1.86392477084005	0.05379518081631
C	3.39921034841852	0.61650461802925	0.26657707159977
O	1.47468189761751	-0.75282506866098	0.90589390398633
C	2.76492292861393	-0.60286902790814	0.67702654772743
C	4.94502884251690	-1.70536301621614	0.61018234535013
C	3.57676050162626	-1.75145711127801	0.83604184641621
C	-0.54586936141475	3.15760960167711	-0.65116780608307
C	0.80355112674497	3.40497547432330	0.04470596297043
H	-3.11802900129266	2.63106448175248	-0.41919538920774
C	-1.36059716356557	4.44701822990198	-0.74094360447092
H	1.87078331152753	4.08585247759018	-1.70305781884198
H	2.56184126903032	4.65316109508292	-0.16532994432840
C	4.80161955230662	0.62394632228556	0.03745555526866
H	3.08554071907039	-2.67689183175109	1.14327687428338
C	5.57353151798873	-0.51104874143506	0.20679977094789
H	-5.08922765438687	1.68550936100973	0.17253103723494
H	5.53901668638021	-2.61312110748638	0.74662320670494
H	-2.89284124826823	-2.61595838661823	1.37577251519424
H	5.26589624433754	1.56223765801362	-0.27755138814584
H	-6.49084297478915	-0.27590188933623	0.78925179208487
H	6.65018435267909	-0.48260732739964	0.02973156194045
H	3.34284437616413	2.69535565074415	-0.28666552447450
H	-5.37289495606005	-2.43879143652927	1.38481479484803
H	-0.32363225501106	2.80713137885182	-1.67534844173643
C	-0.55013541784017	5.53070924951422	-1.46399765852592
H	-2.29966452382766	4.27022469411624	-1.28551187560545
H	-1.62275757043016	4.77707580680122	0.27909229488463
C	0.80990152837887	5.76142802695308	-0.80237287722302
H	-0.39958463588318	5.22551075946727	-2.51458747680347
H	-1.13050686615803	6.46635598148696	-1.48492088045533
C	1.61729575286797	4.46122891802831	-0.69637631797710
H	1.39017812491370	6.50719643249907	-1.36787403646241

H	0.65958068536724	6.17433988771159	0.21044211975057
H	0.58685146161053	3.77311716110249	1.06443669215074
N	0.21447577874361	0.04952718544552	-1.47898255614404
H	-0.67133449213833	0.17140312316922	-1.97522513664376
H	0.93419205617863	0.49363724008390	-2.05360703516544
H	0.41361044925120	-0.95329872069072	-1.48710865345277
N	0.07649013507380	-1.80394632149263	3.49696875763458
H	-0.76178998521688	-1.93997942942615	2.92595713385010
H	0.83365401736363	-1.83341269503798	2.80814395438136
H	0.17175122439412	-2.66084246967686	4.04775437425472

^{1}[TS1a]$^{+}$

Ru	0.12404251780614	0.52350785560894	0.66552650079244
N	0.06932255414811	0.96232206319906	2.27212147337472
C	-3.20007796332781	0.54348634472800	0.40609930671537
C	-4.61956479236544	0.62949424718580	0.41241900128655
C	-5.41844014979265	-0.45584470061815	0.71943739753000
C	-4.80269999216793	-1.69012982848203	1.01524822321652
C	-3.42361187177699	-1.81749272442082	1.00428844931558
C	-2.57422776192951	-0.71605216994855	0.71149191654298
C	-2.49361724474966	1.73067372434252	-0.00197257198195
N	-1.21145255081778	1.90642544661471	0.01022240289296
O	-1.28136126820156	-0.93305019196241	0.71756238440329
N	1.47025344056378	1.97210805005507	0.20815747870441
C	2.73525742586060	1.77236395049359	0.04177941034399
C	3.43017703745680	0.51640205046454	0.19803899193994
O	1.52999802240250	-0.91803979677957	0.76692019279896
C	2.81151149923484	-0.73576306835095	0.54475497154052
C	5.01719020924680	-1.79999378107278	0.41936462624711
C	3.65122861977114	-1.87754392054003	0.63946214152833
C	-0.54559951794921	3.08187023556497	-0.59620235272520
C	0.80928634894171	3.28652493660899	0.11105234744342
H	-3.12416414793868	2.54479075475293	-0.37900396764093
C	-1.36162635993580	4.37434117401497	-0.60105036776429
H	1.86880371699957	4.10115733951970	-1.58453358563082
H	2.55781229579440	4.56075306056907	-0.01205582016026
C	4.83302396107505	0.55379670149385	-0.02553108334815
H	3.17515630135508	-2.82654712831587	0.89597439680044
C	5.62598529461298	-0.57433212425925	0.08248841872768
H	-5.07573654591831	1.59146103552053	0.16281925372489
H	5.62584960653068	-2.70431229744370	0.50694112360332
H	-2.94383284085730	-2.77352405527390	1.22526617737362
H	5.28343060362233	1.51492758417363	-0.28889152540083
H	-6.50616440159698	-0.36249496103406	0.72428932094510
H	6.70224654940229	-0.51679625086399	-0.09120131019891
H	3.36386164877683	2.62399368192865	-0.24428877486744
H	-5.41920372783193	-2.56155290180323	1.25288345268819
H	-0.32311955983899	2.80191882992336	-1.64250985172366
C	-0.55812450490270	5.51039373130278	-1.24603343034189
H	-2.30116965490328	4.23141367362871	-1.15498326145145
H	-1.62619229505933	4.63320753133918	0.43899452474042
C	0.79965816535181	5.70143457254168	-0.56765724728901
H	-0.40327987460919	5.27916404102758	-2.31482350347499
H	-1.14329016483262	6.44258672000132	-1.20390293834478
C	1.61283241352523	4.40103215510006	-0.55321156620958
H	1.37571351515659	6.49003597621671	-1.07708072101780

H	0.64522710173287	6.04081730709399	0.47184301567783
H	0.58942115481441	3.58341756091947	1.15349120347293
N	0.23613610502134	-0.00980103710102	-1.61633632502541
H	-0.63920039570460	0.16075542872835	-2.11703427758004
H	0.97053173770719	0.46359899422080	-2.14749961055715
H	0.42255691591295	-1.01229047703030	-1.69128375114568
N	-0.04578254261033	-0.70840368530120	3.39749062943132
H	-0.82453296712863	-1.25412086104500	3.02578109381925
H	0.83374206358070	-1.19894317233413	3.22950005628008
H	-0.17081372965757	-0.48260762490314	4.38358795997802

^{1}[INT3a]$^{+}$

Ru	0.14429910499306	0.49480016047871	0.20723329971810
N	0.09300982876741	0.80884486408752	1.99302928774246
C	-3.18226258389734	0.54732558387976	0.07841153457162
C	-4.60079796588124	0.64742309664613	0.11252390561639
C	-5.40814388772666	-0.43369789483082	0.41249076962614
C	-4.80509030088515	-1.68197600905420	0.67572192475006
C	-3.42878218836788	-1.82424536751711	0.63629239483262
C	-2.57072309819962	-0.72751418451670	0.34785192329472
C	-2.47189274855137	1.74515731113348	-0.29741923719637
N	-1.18557184438590	1.90429504454725	-0.30651929898880
O	-1.27762078421089	-0.95638382433685	0.33724766253233
N	1.47440108023677	1.96594522339468	-0.08342463077917
C	2.75297574070878	1.79556043257141	-0.18062792584902
C	3.46179727067320	0.54293616763539	-0.03947759773023
O	1.57198214577936	-0.93694362132893	0.42880141858323
C	2.85955447098784	-0.73138186625453	0.25217902598628
C	5.08956305275710	-1.75114793538153	0.18848716110781
C	3.71839662862139	-1.85887356285645	0.35278714946675
C	-0.52987317263944	3.11365184934722	-0.85091763850066
C	0.81679391976326	3.28424253778537	-0.12167718538454
H	-3.10423266565369	2.58103454799559	-0.61859646580420
C	-1.34895220761251	4.40283082829629	-0.79966910302883
H	1.89996213963615	4.18677096581202	-1.75653020850084
H	2.56172531720764	4.57187394922487	-0.15363805193466
C	4.87190553820398	0.61050514515120	-0.20487736916991
H	3.25120802779276	-2.82241309302115	0.56902672336730
C	5.68333158776171	-0.50440835669719	-0.09466695405858
H	-5.05037522322405	1.61991147318078	-0.10684017065585
H	5.71376774382397	-2.64456185624883	0.27836951069621
H	-2.95548104986713	-2.78876942905740	0.83405918159719
H	5.31419951492150	1.58649913762689	-0.42408359714152
H	-6.49428512902861	-0.32525542373381	0.43984595128925
H	6.76418881427568	-0.41985692365301	-0.22435539227655
H	3.38492108738461	2.66829970910487	-0.38249832503852
H	-5.42880368513626	-2.54894341296237	0.91067641202710
H	-0.29873093687575	2.88771497910587	-1.90857450181641
C	-0.54144031517665	5.57021166935343	-1.38061182340708
H	-2.28363717548421	4.28609672068198	-1.36802081962042
H	-1.62399003979616	4.61211315699401	0.24892160896910
C	0.80841579405097	5.73183832155183	-0.67895566078461
H	-0.37386567295503	5.39055029225722	-2.45747615038253
H	-1.12871047867050	6.49882181583910	-1.30144348498167
C	1.62576808352951	4.43463875421720	-0.71599854984910
H	1.38716169482478	6.54593004832567	-1.14359166966860

H	0.64101163479275	6.02158293567598	0.37364441498964
H	0.58264964350650	3.52493608984039	0.93234050125238
N	0.30971622488251	0.03367285631545	-1.98979966605118
H	-0.56320523072057	0.20588743110194	-2.49526254079406
H	1.03406546125964	0.56484760616618	-2.47937494121156
H	0.53214028201038	-0.95573378723039	-2.12044031997292
N	0.09912124717889	-0.48138935554959	2.67659642999126
H	0.07972134521772	-0.38164044406688	3.69863037605506
H	0.94152000153762	-1.01464708096349	2.37144723287106
H	-0.70267983472159	-1.05611044630207	2.34636907248959

^{3}[INT2]$^+$

Ru	-0.00394504298447	0.73595825940486	0.73384466168644
N	-0.46962955654646	1.29674809650757	2.27959523540585
C	-3.26978471712428	0.88015747878269	0.57747841639585
C	-4.65952494269857	1.03469661456634	0.80265890763232
C	-5.43421690524556	-0.00991945299251	1.28093222892217
C	-4.83515828974103	-1.26387926100786	1.51600784194817
C	-3.48445202253574	-1.46447140243168	1.27138337556178
C	-2.67002517663583	-0.40207295269277	0.81632904379336
C	-2.55621247513150	1.99451765677440	-0.00381855461165
N	-1.27153657743978	2.07424439689510	-0.12573315941619
O	-1.39531558207529	-0.65268942385403	0.58732215416742
N	1.35995312097126	2.16959320011905	0.27955562050018
C	2.62406908064091	1.93765665823194	0.10463725751648
C	3.30877254341906	0.70212403042600	0.38108092787732
O	1.45988326512173	-0.42389800009347	1.45781438139849
C	2.70549265911101	-0.41658625867122	1.05532530524584
C	4.83868414181999	-1.59097914940805	0.96246718671739
C	3.50784308638305	-1.55039488345659	1.34309452587524
C	-0.56233119397958	3.17148569576511	-0.80665168080007
C	0.71096256132390	3.46748239016357	0.01935942216453
H	-3.17484063914076	2.81265954896316	-0.39066258658249
C	-1.37600722057124	4.43930224060414	-1.05088697253820
H	1.95240814646300	4.13911515419312	-1.61448991713307
H	2.44948619896553	4.75437302838290	-0.02113847352019
C	4.68164965483488	0.63152066925620	0.03467182739560
H	3.02788329173542	-2.38791748241964	1.85242845662984
C	5.43886606368335	-0.49553224857124	0.30538775012528
H	-5.11446743897500	2.00616786535831	0.59276297943015
H	5.43175386612415	-2.48271315993830	1.18139075405331
H	-3.00383448115764	-2.42774066813541	1.45304307601666
H	5.13723105829139	1.49509673987336	-0.45666873539198
H	-6.50049122607966	0.13414368507618	1.46540542545413
H	6.49220245752878	-0.53361854398980	0.02140645522563
H	3.24976152764521	2.74950579685169	-0.28268171271541
H	-5.44022476814680	-2.09253850988550	1.89292832360837
H	-0.22730662659874	2.76368755772390	-1.77764239729215
C	-0.50243096140701	5.51308132865466	-1.71149186264113
H	-2.24001007491738	4.21413031751738	-1.69451305205333
H	-1.76641099553531	4.80890695147458	-0.08675616197321
C	0.75591794432744	5.80171077627241	-0.89045135681576
H	-0.21130398273042	5.17271649489001	-2.72086353277035
H	-1.09393763842954	6.43247528792230	-1.84394073415456
C	1.57630145774793	4.52797575947263	-0.65240979408919
H	1.38319027739655	6.55037414908115	-1.39906946962837

H	0.46810163966411	6.23453351917978	0.08370592819273
H	0.37945136209503	3.84095027677024	1.00530610505385
N	0.53177729922167	-0.13463586473443	-1.36698617799038
H	-0.27427028937776	-0.03830506502335	-1.98934160894272
H	1.32496028988495	0.28072867790257	-1.86064997905395
H	0.72328997035378	-1.13729909798585	-1.30147620341762
N	0.23006101432554	-3.17212898818764	2.35908182149613
H	0.60660479487768	-3.75305013399968	1.60537744440398
H	0.60893799889447	-2.23750992581265	2.18076974202391
H	0.70554205235367	-3.49939982976633	3.20398154161353

^{3}[TS1a]$^+$

Ru	-0.11929044111546	0.27871071841518	0.49750471325914
N	-0.66293717209677	0.57755798210225	2.10359117699039
C	-3.39432306210050	0.53508999106852	0.10190156000752
C	-4.80188372765715	0.69241272893829	0.17234206570057
C	-5.64595966333668	-0.39307400535407	0.33411274200813
C	-5.09458704662821	-1.68892970755440	0.39583412888951
C	-3.72502095483920	-1.88519284953185	0.30203807332737
C	-2.83870411068098	-0.78760727073828	0.17438753072793
C	-2.61222728455067	1.71635720798923	-0.17621247673796
N	-1.32125989209705	1.78808947947039	-0.16935290765442
O	-1.55082405010674	-1.03457301209013	0.07008325983631
N	1.27771565009732	1.73359530676203	0.38412398245710
C	2.54258417921907	1.51039285284657	0.21185763011586
C	3.19018616534619	0.22474440115367	0.24613810704249
O	1.27949355740417	-1.04131276504164	1.01963921290501
C	2.53721321286617	-0.98967188291260	0.65294223796922
C	4.64299765571843	-2.18456016317951	0.35899997160307
C	3.30251454027627	-2.18180893151296	0.70876454828491
C	-0.56239680109663	2.99042434434691	-0.55837567046163
C	0.66551575652544	3.07145862113372	0.37606541469955
H	-3.18524218010672	2.61066093154604	-0.44671432526738
C	-1.34797430038339	4.29924704987433	-0.55966918386552
H	1.99375059206839	4.05163106521609	-1.01602692537087
H	2.42451863391453	4.29275123191914	0.69295977399839
C	4.57026398892583	0.18556040658898	-0.07835558107248
H	2.79561332204911	-3.09516661764453	1.02632951089430
C	5.29066078690859	-0.99559948008026	-0.03773336524256
H	-5.21485047022521	1.70142733149067	0.09246204890166
H	5.20484846038794	-3.12150299639103	0.39753526825818
H	-3.29189758084380	-2.88677034292299	0.33791302010722
H	5.06045273934613	1.11875915398455	-0.36801728578272
H	-6.72627875430618	-0.24905257243871	0.39794466382799
H	6.34988179204109	-1.00671180759198	-0.30145359983054
H	3.19911254308517	2.36707986429706	0.02388085523682
H	-5.75292141512311	-2.55388902485711	0.51281647180670
H	-0.17829884991697	2.79811468390082	-1.57706169801795
C	-0.42989714335560	5.47247068249144	-0.92294020453440
H	-2.17869901454980	4.24177322204216	-1.27926435793147
H	-1.78505044267346	4.45590503030530	0.44181089966551
C	0.78775535729596	5.54409437683199	0.00095171192482
H	-0.09155395391192	5.35708536888480	-1.96779750588669
H	-1.00321037856535	6.41160568028452	-0.87673768582780
C	1.57858632687099	4.22996850761340	-0.00892422429708
H	1.44917165514620	6.37222834101916	-0.29798754184980

H	0.45436095772568	5.75740812923961	1.03182790959091
H	0.28846280746761	3.23206567692407	1.40178379957948
N	0.44572929228913	-0.20318879095674	-1.71654803152243
H	-0.35645837449694	-0.01018518968489	-2.32109749191300
H	1.23432230305118	0.31294788995703	-2.11334713186255
H	0.65817087385515	-1.19673856759132	-1.83408631284389
N	1.47428392268154	1.11732937624099	3.53218715684738
H	1.22298310497684	1.16710271506996	4.52098387805442
H	1.79852885616384	0.16434878274710	3.36163841362493
H	2.28543803106043	1.72567684537855	3.40256176963022

^{3}[INT3a]$^+$

Ru	0.03205260468649	0.37066567250713	0.09836370811322
N	-0.34908314797019	0.70940466420271	1.94415050014801
C	-3.28658714515740	0.49898409539709	-0.10924046780805
C	-4.69961935523688	0.62975143661832	-0.02518815804159
C	-5.52332411734937	-0.45282994208449	0.22104628499850
C	-4.94280349550317	-1.73158507313215	0.37161635208803
C	-3.57320238180981	-1.90393677895515	0.27912821988447
C	-2.69382431897144	-0.80661790952995	0.04874996439925
C	-2.54686884499529	1.69546813497828	-0.41542504439019
N	-1.25458353085712	1.80699672766737	-0.44872690337082
O	-1.41680015361051	-1.05113403258340	-0.03018151005766
N	1.39095340799807	1.84458957417199	-0.00848664388590
C	2.66828069729579	1.67474308782094	-0.13332097125102
C	3.35726489615641	0.40433730969787	-0.07053678043081
O	1.47262754895519	-0.97600245851930	0.63955084041703
C	2.74412480824348	-0.83945570166621	0.31339677857247
C	4.92390402676542	-1.93267890456713	0.07259615600171
C	3.57126019782716	-1.99001387164866	0.37486445179177
C	-0.55593963645513	3.04902555374731	-0.84573288194336
C	0.73032316092713	3.16216143225269	0.00729882569835
H	-3.15595853617057	2.57612595383878	-0.65039281672540
C	-1.37601270780978	4.33541230279735	-0.76525256914015
H	1.93956393708357	4.19869238600555	-1.45229674995620
H	2.46518904396422	4.43826644558207	0.22847681558920
C	4.74672948536498	0.42703340743125	-0.35750361408004
H	3.09965386094835	-2.93011776407563	0.66982886265432
C	5.52717930231220	-0.71539285870164	-0.29921185527332
H	-5.13140111555442	1.62572045663974	-0.15870187726934
H	5.52541543069957	-2.84435183172986	0.12610831990289
H	-3.11937205694160	-2.89135578331379	0.39137925134687
H	5.19923726677902	1.38401846212884	-0.63244019093963
H	-6.60522356527875	-0.32370385124580	0.29182951030074
H	6.59320364101089	-0.67192956721755	-0.53149575055439
H	3.30616813909702	2.55194828576196	-0.29314116192071
H	-5.58146188938637	-2.59859646092617	0.56298097573843
H	-0.23278880147768	2.90237511867325	-1.89329557055727
C	-0.52532573605776	5.54420366712625	-1.17370880019372
H	-2.25671121010201	4.26730542292541	-1.42142663624242
H	-1.74323150892773	4.46220391247050	0.26835942877925
C	0.75463119821653	5.64500150210166	-0.34133015933774
H	-0.25973076092477	5.45434394361267	-2.24199862416294
H	-1.12271119559396	6.46406037822770	-1.07178533094012
C	1.57963979572148	4.35510531662196	-0.42031227465096
H	1.36677743965679	6.49672423443686	-0.67776205317048

H	0.49096588245721	5.83931378901879	0.71348036928622
H	0.40634364407259	3.31440479035222	1.05284619210179
N	0.49132029529045	-0.13593108189246	-2.00887551008977
H	-0.22876981764864	-0.76429814928169	-2.37409125509990
H	0.53829052196604	0.66272276258783	-2.64640419933419
H	1.38175711878921	-0.63121183032960	-2.10354256574063
N	0.77366725596572	0.52309701414751	2.82077347007095
H	1.42045672917068	1.33026128457128	2.80713138965094
H	1.32451404075505	-0.30760840116963	2.52018645777324
H	0.46996585903289	0.39514055621128	3.79528139409638

^{1}[INT1]$^{+}$+2NH$_3$

Ru	0.24762417608447	0.78471785794991	0.68851560574322
N	0.17547903029516	1.24763023609487	2.21968848600867
C	-3.06189328497080	0.72242829342915	0.37225036383509
C	-4.48088051064691	0.78341156950895	0.41201262287050
C	-5.24409224472948	-0.30405958551622	0.79633284435565
C	-4.59320879344383	-1.50579933896685	1.13912807151475
C	-3.21025068830324	-1.60779642905357	1.09401242935903
C	-2.40619521371457	-0.50639855233959	0.71414337798116
C	-2.37791675389891	1.89675801574858	-0.09995009132979
N	-1.09945540195576	2.10081007912992	-0.09636977440320
O	-1.10147664532832	-0.69751950697847	0.67356020294088
N	1.57268133417864	2.19329757301768	0.06409420786632
C	2.83965398425927	1.98436725898369	-0.09526447672022
C	3.53955233401995	0.74789304530537	0.12553648460244
O	1.62274576180686	-0.65759760734437	0.69786682093875
C	2.91390272595226	-0.48469318537028	0.51058044775605
C	5.11404112071018	-1.55001670955789	0.49999766767368
C	3.74084373032903	-1.62086745826340	0.68550145953500
C	-0.45265507994789	3.24300732356025	-0.78346566262209
C	0.90590772939660	3.49959139461315	-0.10807822537133
H	-3.01809764549581	2.68013281556606	-0.52319874358864
C	-1.28019933091096	4.52536300524801	-0.85352823421368
H	1.93742924837217	4.19157942332198	-1.87252014189696
H	2.64887112182193	4.76549525341869	-0.34676881061879
C	4.94737803091326	0.77965892871208	-0.06546894110020
H	3.25635569044418	-2.55455241173270	0.97771928733860
C	5.73347967071452	-0.34286873195535	0.12052424966580
H	-4.96450695137560	1.72214950653730	0.12959544616229
H	5.71966386473008	-2.44795640681662	0.64972512807116
H	-2.70164305727326	-2.53570622196940	1.36128268983393
H	5.40422749609974	1.72736072404105	-0.36279589397860
H	-6.33297539181885	-0.23477610162178	0.82812982771084
H	6.81413690468258	-0.29530150885149	-0.02519537792820
H	3.45888317780722	2.82624509146951	-0.42614461548833
H	-5.18260437255746	-2.37523200316837	1.44268932839966
H	-0.24450652243699	2.90052422198913	-1.81329381303473
C	-0.49307995307918	5.61756328774937	-1.58911380441536
H	-2.22824399569012	4.34169290731593	-1.37996470800975
H	-1.52516587048381	4.85015321420460	0.17245792989527
C	0.87727833350690	5.85790747130063	-0.95257303469163
H	-0.36003763483643	5.31583040838646	-2.64307723318176
H	-1.08131062879324	6.54850232304830	-1.59676230786686
C	1.69740254763411	4.56433994229650	-0.86153963163616
H	1.44125861127479	6.60846713388511	-1.52815703261045

H	0.74241520985222	6.26926926840300	0.06305634282846
H	0.70233577859610	3.86458814793906	0.91566389791748
N	0.31295517741810	0.15608506568877	-1.65006093348492
H	2.50812362487582	-0.54354965272103	4.80661609210995
H	3.09574608756805	-0.91923349181197	3.34024039924548
H	-0.57790813811646	0.27675199847373	-2.13734279731281
H	1.02519553900777	0.60729394716555	-2.22851556691389
H	0.51776056436129	-0.84545805844987	-1.66771995855028
N	-0.20519569094765	-2.29577500401132	3.17427864861872
H	-0.09069192625638	-3.30083634172129	3.02363012529369
H	-0.40862015591055	-1.89666843298164	2.25277643275534
H	-1.07109365652620	-2.19797991004557	3.70994773696323
N	2.29948610492375	-0.47752406372849	3.80685974622933
H	1.50369421901162	-1.11456854222489	3.64499754054817

^{1}[TS1]$^{+}$

Ru	0.15632592642575	0.60994199963976	0.58693005484953
N	0.06899986116289	1.06534025297515	2.14467184798571
C	-3.15503650128413	0.58310027305338	0.29180875386141
C	-4.57488567107320	0.65553212957513	0.32055803952947
C	-5.35535555394631	-0.43543510373833	0.65371715355071
C	-4.72022740252860	-1.65903198855345	0.95204171923500
C	-3.33973627522465	-1.77261188364723	0.91915203405102
C	-2.50997003253819	-0.66561583923505	0.59906666231815
C	-2.46412461585773	1.76875726381316	-0.14082407899061
N	-1.18365419843027	1.95927960005306	-0.13833094121842
O	-1.21469130290425	-0.86845412564378	0.57687205369028
N	1.49272663152074	2.06990110329899	0.09731761365916
C	2.75990526411416	1.88078530184213	-0.05736095594438
C	3.46295311169343	0.63050049330106	0.10372296412033
O	1.55767811207152	-0.82156648105740	0.61106376585266
C	2.84372993139847	-0.62892244925122	0.41064285709929
C	5.05396783711966	-1.68106520470563	0.31297306745992
C	3.68208367073726	-1.76988491917236	0.49560758576142
C	-0.52566041691028	3.13180171300043	-0.75864536738649
C	0.81275208538989	3.37252317482961	-0.03155329297654
H	-3.10269701929286	2.57224502416750	-0.52708188774709
C	-1.36313229366055	4.40914541568891	-0.80484417867527
H	1.88897856523512	4.16790427268698	-1.72503541069453
H	2.54240468874297	4.67185873343424	-0.15047934416165
C	4.86992123538657	0.68070213689107	-0.08430461940710
H	3.20417703011302	-2.72668303243134	0.71670546065909
C	5.66557362872926	-0.44618936267516	0.02252456180989
H	-5.04552480044593	1.60963080104813	0.06815755177819
H	5.66363919775934	-2.58511432935212	0.39428207799964
H	-2.84616501541786	-2.72114332754322	1.14136028675239
H	5.32091634693358	1.64891264664683	-0.31843052106422
H	-6.44378114716561	-0.35477021263761	0.67670697450525
H	6.74571120633520	-0.38013205918011	-0.12064227755074
H	3.38262159772296	2.73784871251729	-0.33999835113170
H	-5.32280033454535	-2.53440329606527	1.20988546801554
H	-0.28015737094939	2.83354278643189	-1.79427244596214
C	-0.56569121549481	5.54402752143938	-1.45956595188266
H	-2.28910179276786	4.23801683364147	-1.37350260385561
H	-1.65149972820600	4.68524747211785	0.22431162079113
C	0.77611103803316	5.77145901353440	-0.76094439854146
H	-0.38785550770508	5.29278176113639	-2.52017791681684
H	-1.16662124695837	6.46693124863350	-1.44774194625879
C	1.61021541662614	4.48541390781099	-0.70502770011236
H	1.34879930024458	6.55827746337199	-1.27669195344941

H	0.59735253512464	6.12968500595909	0.26816596515627
H	0.57124887081274	3.68457957516535	1.00139930431767
N	0.28673740447843	0.08338714921585	-1.74284855309244
H	3.04205120709015	-1.04343678241894	4.70864721040432
H	2.90675157150358	-0.15495535004148	3.35099631755859
H	-0.58532893152215	0.24378761503387	-2.25213348402272
H	1.02394972265224	0.55499043120004	-2.27153068236042
H	0.47972084706342	-0.91860739061607	-1.80619924965875
N	-0.40767162342302	-0.79788923219028	3.49369143076481
H	0.58688685473471	-0.99703985466856	3.69281539158213
H	-0.79668436631206	-1.52233898379132	2.89128792818820
H	-0.94547523667249	-0.74608270142562	4.35880332295388
N	2.59584164038372	-1.02634407012957	3.78821090884726
H	3.05079634549766	-1.76711320988380	3.24901480055397

[1][INT3_NH4]+

Ru	0.08316904167373	0.46604681099397	0.28036086910303
N	-0.04951773301788	0.80236658871813	2.11065486036980
C	-3.24241405147306	0.55301610006605	0.21813998450526
C	-4.65535135099815	0.68225254977375	0.30289998950058
C	-5.48223416251303	-0.36934636484649	0.65787833308438
C	-4.89774959631798	-1.62407566035346	0.93155455891754
C	-3.52714474578133	-1.79703673404965	0.84515644015417
C	-2.64232492043915	-0.73344417657538	0.49312862748869
C	-2.52669779703855	1.74406880766071	-0.19175410002255
N	-1.24068670185140	1.89006605638752	-0.21963477549584
O	-1.36748939912645	-0.99820806695355	0.43176816303901
N	1.41300441929482	1.94038646532592	0.06020682418978
C	2.69723270860630	1.78293849397149	0.02572578727382
C	3.40679309190683	0.52795904799804	0.19414812716686
O	1.51534970237620	-0.98738571943333	0.52157025785539
C	2.79447983948646	-0.76272309210673	0.41987740849992
C	5.05586671230752	-1.74537945501727	0.54896891423648
C	3.67489382511233	-1.87627682912122	0.58504974244449
C	-0.57597974914393	3.09818949926364	-0.74519993257096
C	0.75913957828242	3.26032359387015	0.01155055050082
H	-3.16133091031823	2.58198548001527	-0.50585798804135
C	-1.38974324063784	4.39141816415100	-0.70529066213562
H	1.87805814937536	4.17182886885625	-1.59493050981868
H	2.50518554156532	4.54939182760805	0.02221337732646
C	4.82297958814489	0.61247458973528	0.15403121362479
H	3.20951502434166	-2.85238529589704	0.74621352466255
C	5.64842091555524	-0.48689674350961	0.33088567737544
H	-5.09087206505200	1.66083229912348	0.07856936320480
H	5.68367391815494	-2.62980525112371	0.69053968645601
H	-3.07050008869670	-2.76822452702553	1.05310257351157
H	5.26769853037007	1.59790456306949	-0.01520355946448
H	-6.56373184786727	-0.23143017970957	0.72193524877511
H	6.73452979997756	-0.37779523445640	0.30250465127833
H	3.33763593834841	2.65995660332655	-0.12712561888210
H	-5.53056005907896	-2.47092712268624	1.21337632569748
H	-0.32140823706295	2.88062162623476	-1.79994950419954
C	-0.56918811419555	5.55959803842226	-1.26505045269618
H	-2.31522221891233	4.27892511242906	-1.28985487158529
H	-1.68471361515771	4.59864169700918	0.33854854405234
C	0.76642020955621	5.71431773912633	-0.53467892907986
H	-0.37875448703547	5.38348033512670	-2.33888172506326
H	-1.15472122812045	6.49041254408862	-1.19560374259589
C	1.58066649460998	4.41496000186407	-0.55946313597021
H	1.35646321343154	6.53032187274850	-0.98210843584234

H	0.57642653880482	5.99945483886555	0.51558413303684
H	0.50088758208027	3.50030218729919	1.06046619256603
N	0.27368420928448	0.01525705297723	-1.89222479035351
H	2.93959778264230	-0.97148042187027	4.46239120851787
H	3.17109562985764	0.32679164009139	3.42313018245867
H	-0.60891357539981	0.12375398735934	-2.39773191444583
H	0.95918221079251	0.59659713406422	-2.38091514447090
H	0.56801811046453	-0.95487752049142	-2.02340088217164
N	-0.21212896721993	-0.22603072606934	2.88927002430294
H	1.79815025003208	-0.62900677729245	3.26333781239230
H	-0.48768791633615	-1.13314350864634	2.48388780458453
H	-0.44367403831955	-0.06387744476145	3.87232549213199
N	2.80768511744057	-0.63029776746429	3.50347307132876
H	3.31448300003482	-1.23487911066043	2.83873971079117

[1][TS2][0]

Ru	0.19762243162184	0.50704858748561	0.28931786472759
N	0.09930426112141	0.81276636125682	2.12992594722808
C	-3.13184336409512	0.57981637810684	0.29844310792454
C	-4.54319823292823	0.70188078557136	0.41192183379733
C	-5.35878866973306	-0.35671586109966	0.77268520280477
C	-4.76334993557540	-1.61130801080100	1.02289399715466
C	-3.39397471156412	-1.77713246105574	0.90874868828595
C	-2.51983276353616	-0.70599742072598	0.55053488398536
C	-2.42888245490004	1.77655718501896	-0.11633571706098
N	-1.14401154705779	1.92543947501589	-0.17550228525830
O	-1.24582422036198	-0.96318607360638	0.46569862749110
N	1.51616520469712	1.98925282238577	0.03316462227880
C	2.80041238040043	1.83606271519961	-0.03681954843582
C	3.52199840467926	0.58899828870856	0.11395371994869
O	1.65289962702425	-0.93271153124591	0.52434216037901
C	2.92621508736114	-0.70164146462991	0.38649821808217
C	5.19222439946676	-1.67626841729887	0.38590927564460
C	3.82010208470561	-1.80968487485663	0.51003913372662
C	-0.49950855945022	3.13787629456664	-0.71693984377845
C	0.85631093972133	3.30662507983481	0.00003122873817
H	-3.07283703460241	2.61526884697354	-0.40854958910178
C	-1.31700537810261	4.42811421477207	-0.65450731362751
H	1.92056487233650	4.21496916293108	-1.64451932455997
H	2.59788229001123	4.60223552994921	-0.05025494710108
C	4.93485823884278	0.67937504922928	-0.00859533528532
H	3.36876221901844	-2.78447547157957	0.71302944189811
C	5.77042231251183	-0.41659641476597	0.12290593509386
H	-4.98711598533331	1.68058610907094	0.20493180093701
H	5.83072290589130	-2.55823268071499	0.49394873731320
H	-2.92886464141703	-2.74768280533328	1.10019828940424
H	5.36473203722066	1.66534703046942	-0.21129149658979
H	-6.43948640630662	-0.22429334325470	0.85863905626673
H	6.85285738175698	-0.30765840904975	0.02625828694557
H	3.42986655687352	2.71686643424983	-0.21316141249869
H	-5.38682109758523	-2.46372339336275	1.30889043854969
H	-0.27484753912579	2.92186173051514	-1.77878696724300
C	-0.51895977949351	5.59777866916466	-1.24318919220491
H	-2.26008733008731	4.31129537372663	-1.20932505829835
H	-1.57966482989292	4.63733295603975	0.39753456537040
C	0.83873448517914	5.75916314812155	-0.55645001263607
H	-0.36241306525940	5.41935315749784	-2.32215013697135
H	-1.10525585438756	6.52684887789188	-1.15772781336034
C	1.65556363243734	4.46198520366120	-0.60119881651199
H	1.41185752810377	6.57491630459269	-1.02595882161218

H	0.68151730968749	6.04860623632900	0.49807359773040
H	0.62780817784826	3.55245603153158	1.05481415744505
N	0.34246853749593	0.02621033941314	-1.86929716457559
H	-0.59885888713782	0.94239329966489	3.13464113515031
H	-0.31489754410754	-1.07200443968295	2.56920079745727
H	-0.57818195435999	-0.05432036690740	-2.30715041661684
H	0.87419768827435	0.69766960448863	-2.42857652392808
H	0.80137055438326	-0.88025394706297	-1.98426626638051
N	-0.07078935397073	-0.17032499400047	3.03322405007762

^{2}[TS2']$^+$

Ru	0.06636433492368	0.44882082870016	0.17932004879063
N	-0.26685948034026	0.82857949872350	2.05105067319220
C	-3.22741401237405	0.61537308537971	0.34093702077112
C	-4.60700075233204	0.76099163601197	0.64515575442147
C	-5.39561936013466	-0.31873786530978	0.99920609798644
C	-4.81822974289792	-1.60635382989882	1.03144621661503
C	-3.48231852544192	-1.79296755199090	0.71874729778578
C	-2.63887357696316	-0.69927277251588	0.38114782615239
C	-2.52492398635571	1.80386431574597	-0.07421967238971
N	-1.24769790048580	1.89226703873335	-0.28087447241493
O	-1.38760682018403	-0.94956492484917	0.09542372215735
N	1.40798171073789	1.94726888722973	0.02786461938476
C	2.68495599964736	1.78371629090875	-0.09606416026244
C	3.38287285731287	0.52058874088068	-0.00442639606629
O	1.51083424053695	-0.82454900825409	0.77764283474259
C	2.77927042737191	-0.70668654610060	0.43632682450945
C	4.95770459861453	-1.80185494702123	0.23207170995613
C	3.60842523000148	-1.85041156445462	0.55123183470083
C	-0.57597475559093	3.11121203295385	-0.77306461652783
C	0.74249798430016	3.26355731175146	0.02356979131289
H	-3.14924184293718	2.68947265923403	-0.24056308045714
C	-1.39581413370131	4.39804149547043	-0.72870185105118
H	1.90061594450413	4.22825695513905	-1.52184448389584
H	2.47557543251420	4.55787820125231	0.12737590444884
C	4.76910224812148	0.53732394336751	-0.30249917132327
H	3.14306486267918	-2.77621399135406	0.89658144001421
C	5.55234860825566	-0.60066078255029	-0.20105103961517
H	-5.04109384721697	1.76359191447379	0.59764468557601
H	5.56408306525900	-2.70721283785575	0.32272714902386
H	-3.03038549039148	-2.78721551157830	0.73780306202260
H	5.21687205254873	1.48329616016267	-0.61969835290579
H	-6.45090847362393	-0.17919603189167	1.24214582923780
H	6.61631762943347	-0.56469638128983	-0.44370224476926
H	3.31609039912264	2.66247093157496	-0.27268085175188
H	-5.43114428621977	-2.46956326798926	1.30530972382383
H	-0.29525807452164	2.90358449461650	-1.82197867812879
C	-0.56248275200235	5.58162707857108	-1.23493388871358
H	-2.30182277357126	4.29027102593562	-1.34416850975801
H	-1.72175447336347	4.58375076717813	0.30971502900035
C	0.74401054246695	5.72810309111740	-0.45204638897526
H	-0.33275448508064	5.43111562775290	-2.30463696254918
H	-1.15746752628911	6.50587290004094	-1.16463747568480
C	1.57160605760344	4.43781446919107	-0.48904424567752
H	1.34245908468513	6.56050956442538	-0.85478637751301

H	0.51486773493876	5.97960972554995	0.59865546398763
H	0.45873377140442	3.46009599656684	1.07382580995849
N	0.47258261225323	-0.03156169449668	-1.89204585171156
H	-0.72334068644772	0.76545891925741	3.22590312605663
H	1.22924731564209	-0.20175633480005	2.85438515415280
H	-0.37698045538727	-0.00608310149462	-2.46238691009833
H	1.14281947766559	0.59852721443263	-2.34061946414334
H	0.85398335976296	-0.97719183590589	-1.98280634658675
N	0.42923534844667	0.44280710097165	3.05951812728871

^{3}[INT3b]$^{2+}$ + 2NH$_3$

Ru	-0.19557270798540	0.16421584308992	1.29802007445270
N	-0.45014379415859	0.34425081294598	3.23469173930595
C	-3.32590961356568	0.88777536993857	1.79216655343373
C	-4.57312190045971	1.22329192795810	2.37578841080632
C	-5.46469860255190	0.25047486679736	2.79407558713795
C	-5.14089034161453	-1.10639953924792	2.59583856559554
C	-3.94572122142421	-1.47219561490153	1.99563592152828
C	-2.99592699046578	-0.49855880260640	1.59919353910556
C	-2.48014008985590	1.98310399329169	1.37878614169424
N	-1.23449730071296	1.87164523185532	1.04473679903282
O	-1.86956441405601	-0.90473141551747	1.05196661132391
N	1.36548435070387	1.45803774420634	1.40771031154078
C	2.61355660277110	1.11959241125149	1.45514316619824
C	3.13369221579663	-0.22276605445253	1.51139337099423
O	1.01788828648279	-1.38782839651700	1.72362207182986
C	2.32309670442573	-1.40023260777856	1.64887971053024
C	4.36433602512648	-2.74613655820119	1.71992453363105
C	2.98392985731577	-2.65250301594806	1.75675728581695
C	-0.37105375028199	3.00269516049131	0.67091616347784
C	0.91606943702265	2.86222834501620	1.51526869443600
H	-2.94470356900293	2.97417496352990	1.36499179575495
C	-0.98090665492153	4.39364066545723	0.81134780801683
H	2.28434917954736	3.74607146517772	0.10270767362257
H	2.82270806937384	3.85285951813165	1.79424447967182
C	4.54595111101929	-0.35706009028829	1.49934094212626
H	2.35995191552355	-3.54179844258859	1.86452318753490
C	5.16399687911364	-1.59142737697700	1.59379535327935
H	-4.81254401338060	2.28159180271318	2.50820653578257
H	4.83696130290891	-3.72847905611449	1.80118431683573
H	-3.69055423185074	-2.52202152938537	1.83669934764368
H	5.14810329242075	0.55153206213149	1.41535647668829
H	-6.40788701867619	0.53002200689456	3.26700378977179
H	6.25255570240994	-1.67047310437959	1.57760057546541
H	3.36491819780621	1.91559735105486	1.49069628911251
H	-5.83793966910226	-1.88349322625506	2.92011496063271
H	-0.08684179528298	2.84594938839416	-0.38497749667742
C	0.05207878483061	5.46013019103990	0.42895292833180
H	-1.86890390187362	4.47797432659257	0.16550739526729
H	-1.30927750662777	4.53749295143045	1.85387454371582
C	1.32918291259677	5.32363121328731	1.26075170303561
H	0.29857470916241	5.36221708314889	-0.64323743223369
H	-0.38738797969664	6.46117074977386	0.56340677363660
C	1.94395152592498	3.92482947998364	1.13752885885430
H	2.07224031953100	6.07748581724311	0.95610632556617

H	1.09684972146580	5.51725717511692	2.32161658654353
H	0.62449123542748	3.00933136341981	2.57031418798346
N	0.18032503360948	-0.06788724818915	-0.73573670462566
H	3.21503836001448	-1.56266445055063	-1.13587524876279
H	2.39629592185596	-1.95744057306387	-2.49419548325892
H	0.95350656957154	-0.75444084591229	-0.95317810391517
H	-0.66544840418561	-0.41813892256231	-1.19568473027900
H	0.42789031395830	0.80139605125400	-1.21602511267754
Ru	0.50393359257061	0.83904557370594	6.08736011580690
N	-0.30153259304821	0.49404225096058	4.33033862000925
C	-2.23424757635598	-0.83330348457476	6.60531147377503
C	-3.44435097088298	-1.55450194540691	6.44896460093211
C	-4.67166117206642	-0.91471800782026	6.41996675097996
C	-4.72178269657438	0.48291523933528	6.59204435203711
C	-3.56248239043187	1.21741101388775	6.78810886718923
C	-2.29140846992081	0.59284895828547	6.77863710769339
C	-1.00883133328825	-1.59720916296133	6.63811018820481
N	0.18075036812457	-1.09220986341363	6.55149762949365
O	-1.22290867945347	1.34133578834556	6.95414971498315
N	2.25233298109125	0.09248033589870	5.37425272635299
C	3.23832339857778	0.80135049665388	4.92612635970413
C	3.27007853030465	2.23637248036317	4.79234892404205
O	0.96461749394112	2.67869179590579	5.40558889077513
C	2.14746191106055	3.09988953799866	5.02947983638328
C	3.51771979632500	5.00508454824157	4.34521429154685
C	2.31225845483703	4.48856144090391	4.78954781291073
C	1.42502640955919	-1.87639677407070	6.55169658031819
C	2.25182282145769	-1.38569654062335	5.34050716180929
H	-1.11651102278151	-2.68317692600732	6.73698738198138
C	1.27017522561479	-3.39288535332452	6.53876246616936
H	4.21118278561303	-1.71260379654767	6.17718119416413
H	4.17036274881099	-1.74663499827288	4.39959492141144
C	4.48315500819491	2.80132254032075	4.31999444937344
H	1.44994493929460	5.13495090838593	4.96430419740264
C	4.61988028085789	4.16045119619612	4.10089345914979
H	-3.38882369624037	-2.64011812687951	6.33380581068765
H	3.60727929075279	6.08037587084668	4.16992552118981
H	-3.59346407500572	2.30007606861388	6.92286024842645
H	5.32122025208181	2.12780687529685	4.12234782181606
H	-5.58977468298361	-1.48557044475872	6.26986065256972
H	5.56347748475269	4.57189293599841	3.73787782909750
H	4.13627187569736	0.27873722666507	4.57967421962267
H	-5.68583348003436	0.99771953885309	6.57131048894728
H	1.97523653555614	-1.58312478553389	7.46368305825314
C	2.65067196696633	-4.05960307860052	6.51208679338265

H	0.70586010766096	-3.72096471886786	7.42535936573894
H	0.69367670700040	-3.69415318281522	5.64701356964665
C	3.47971466760188	-3.57629501471357	5.32056823050027
H	3.18373233084107	-3.82932403252171	7.45144575759217
H	2.52915349307660	-5.15360558961040	6.47590416801072
C	3.62528081487169	-2.05036992970880	5.30471828901227
H	4.47943685796327	-4.03848005276691	5.33211871131208
H	2.99321627561490	-3.89595239377612	4.38367931588004
H	1.69168658687729	-1.67141127079615	4.43137483685008
N	1.46880124228927	1.28626305950908	7.88599112367786
N	2.30285981945155	-1.87266242253730	-1.47905281645792
H	2.18922863161217	-2.82550384668269	-1.12528515945729
H	2.44639517630356	0.98275645367601	7.91879484910280
H	1.46767378965848	2.29867743392386	8.04471574105897
H	0.99893225166045	0.86150389258125	8.69105812125243
N	-1.91131120724686	3.49296792822862	4.32478089421420
H	-2.38389661325322	2.58671421672541	4.32262011119966
H	-2.34247834282429	4.01878077042918	5.08834097846427
H	-0.94982463371798	3.30699865707579	4.62596665349214

2^3[INT7]$^+$ + N_2

Ru	0.69963551076675	0.75448834828223	1.37535859387530
N	0.49171662798762	-2.98167341144211	3.43662768953118
C	-2.57003429501411	0.84306965748827	0.88069393110617
C	-3.98379288767390	0.97162634249771	0.87664628124597
C	-4.81821825053128	-0.09552105359649	1.15684348891907
C	-4.24609089206892	-1.35530952997654	1.43279913338761
C	-2.87217247610344	-1.52607738998901	1.42680035044190
C	-1.98764547379136	-0.44353786140028	1.16435831615782
C	-1.81215866937024	2.01917171858857	0.52761811838043
N	-0.52100435232644	2.13185427797169	0.58322687417886
O	-0.70136607665349	-0.68115408232260	1.16987107124138
N	2.00773838729216	2.27152977752229	1.38026135919528
C	3.29019230421806	2.13698324057485	1.51213224424912
C	3.97586298024495	0.91836872753067	1.86358908861959
O	2.01599805230828	-0.43837531725250	2.32713605691407
C	3.31385140475668	-0.28430429507515	2.29044976204829
C	5.50751056583132	-1.27278405303409	2.75655641449646
C	4.12603082858324	-1.36261886474669	2.73824156780650
C	0.20700902473443	3.33780954645002	0.14240291151804
C	1.35354440369657	3.57162152974085	1.15158291732670
H	-2.40110138725215	2.87204002911804	0.17196007897640
C	-0.62385054439234	4.60421616818468	-0.04585843777030
H	2.76227959385513	4.44868620416747	-0.23058978732692
H	3.03276462993025	4.90234322084990	1.46592916027932
C	5.39267339083133	0.97440089725191	1.90320432483352
H	3.61422541617687	-2.27180955361474	3.06006477728473
C	6.15820832098635	-0.09467321643121	2.33358738757863
H	-4.40995345075786	1.95194545065979	0.64556631570136
H	6.09712439891103	-2.12589191661492	3.10334839227575
H	-2.42318203895091	-2.49913606648655	1.63732429426375
H	5.87863136151460	1.90388211215232	1.59389693417588
H	-5.90251347981874	0.03288580231516	1.15752293719343
H	7.24765044272179	-0.02574302176420	2.35309562403286
H	3.92831938560706	3.01680967044574	1.38440782125279
H	-4.89278884077200	-2.20921118821170	1.65312295379354
H	0.67717973550767	3.07633125951963	-0.82351149301854
C	0.27456810526731	5.77092478509751	-0.47467143507635
H	-1.40411989456264	4.43879335934109	-0.80432542943597
H	-1.13209973909385	4.84824694033854	0.90340163979799
C	1.42240479895856	5.99438612855667	0.51253646081893
H	0.69038254229532	5.55667424363121	-1.47512727847426
H	-0.33187732162689	6.68528589729371	-0.57245992861669
C	2.25381509001541	4.72164943910852	0.71058005815400
H	2.07467988168553	6.81135753734928	0.16560240502891

H	1.01007698025764	6.30798270210155	1.48802771629304
H	0.88694744393762	3.84825813169955	2.11448249694138
N	1.51042607913960	0.14987752110682	-0.48822220243400
H	-0.92394149856410	-0.70897733497634	5.59788277287864
H	-0.27142927628133	2.21392932226083	3.45938632493014
H	2.10348863562290	-0.67706061633442	-0.37340512963206
H	0.77524209550065	-0.11479486711950	-1.15002558484303
H	2.08316664835494	0.85859677910913	-0.95479236792588
Ru	0.33010236711963	0.71869511772289	7.54508935196048
N	-0.14265871022123	-2.24705915707864	3.95373142856019
C	-1.73326298815172	-0.56062082988093	9.79999314900522
C	-2.70035430412780	-1.20405896074209	10.61726585832727
C	-3.82794683629972	-0.54773684506394	11.07526925178793
C	-4.01157846417534	0.80990549908646	10.73690020639255
C	-3.08281777509888	1.47741677429885	9.95725879304655
C	-1.92446703804037	0.82433940451426	9.45227835621564
C	-0.57720793385809	-1.34174656682243	9.43342500251684
N	0.36330479435626	-0.96918633634120	8.62094465584900
O	-1.08808537508228	1.52308057320824	8.72913244316677
N	1.82300169032813	-0.17294518530433	6.55063933913889
C	2.68346342373425	0.45213426292911	5.80778145320022
C	2.61354750568804	1.84791596770689	5.44458845482299
O	0.37402199522142	2.29289939651680	6.27606676076802
C	1.46222319727652	2.68607225507359	5.66108289515297
C	2.61487586840500	4.49314203895786	4.47604480396656
C	1.49535460935534	4.00294587869548	5.12868128976546
C	1.56741073391512	-1.78090030512725	8.35157324011788
C	1.90338518633375	-1.61259785965128	6.85293083304075
H	-0.50319058459584	-2.32627311289334	9.90844217213238
C	1.48840886330558	-3.25761333319651	8.73175012656811
H	4.04520298934140	-1.82967313895568	7.01971762037884
H	3.39930427214576	-2.21705025620764	5.41123579301317
C	3.73673270619335	2.38491595558450	4.76714822302765
H	0.60781128180471	4.62355186618741	5.27014412970040
C	3.75775416115499	3.68736877806713	4.30222073802326
H	-2.53225917733900	-2.25165964478817	10.88231207963471
H	2.60805873770422	5.51719388851026	4.09332971444310
H	-3.21739583498112	2.52979631044589	9.69691332520456
H	4.59989348739543	1.73508465571984	4.60901781321471
H	-4.56060408254662	-1.06850811117631	11.69508844014100
H	4.63767266202013	4.07695119303906	3.78730888206197
H	3.53234718365912	-0.10354242527211	5.39444730641516
H	-4.89559217479978	1.34451791037947	11.09551448894827
H	2.39006199432853	-1.31139294511716	8.92168241484482
C	2.78853839209918	-3.97821305030408	8.35335261618520

H	1.30881695982506	-3.36235909699981	9.81254898621864
H	0.63309224055549	-3.72038064043802	8.20903407843742
C	3.12842267700896	-3.79705144025476	6.87226278820037
H	3.61421419366070	-3.57761136371083	8.96772957877124
H	2.69982902756617	-5.04815814384369	8.59958893614816
C	3.20697980925552	-2.31459271262864	6.48960463303739
H	4.08383430123753	-4.29068773941945	6.63394272294401
H	2.35438552138632	-4.28636509616893	6.25474077194124
H	1.08265333529954	-2.09063002934986	6.29134500321930
N	1.79076385376246	1.67201938641801	8.74815127522667
H	-1.03503875900431	0.76545142172582	3.39465877823985
H	0.48451612075079	0.86042501956683	3.99819913420279
H	2.73945779261389	1.30152456013888	8.64399955617693
H	1.84193083710426	2.67371966945937	8.54138184738834
H	1.55731486062732	1.59626168329246	9.74235646025982
N	-1.23575078594370	-0.14011502580859	6.38914452046825
H	-1.84243440627312	-0.74671049798765	6.94806094700350
H	-1.83630713828072	0.59191151646036	6.00020213910885
N	-0.12740952461213	1.22031848101026	3.26380414029584

^{2}[INT6]$^{+}$ + NH_3

Ru	0.05990065627147	0.26962532213463	0.26466324186309
N	-0.40715241382880	0.55590743828988	2.17363373111180
C	-3.24446403608304	0.53899677111824	0.25894462246220
C	-4.63704017438509	0.71174632846503	0.47360492231114
C	-5.47350397617584	-0.35887925017522	0.73544333080267
C	-4.93086615471761	-1.66069194413421	0.76306086066663
C	-3.58081973965482	-1.87252286282935	0.53571454479841
C	-2.69391054075152	-0.79029239967622	0.29388575174510
C	-2.48552767535280	1.72261162790341	-0.06411574249783
N	-1.19737345617961	1.78794577887469	-0.18680470479676
O	-1.43050422839795	-1.06311793067326	0.07417256567208
N	1.43619680403339	1.73828387825729	0.19514454603172
C	2.70676136722700	1.54329673036713	0.04053264646007
C	3.36611560835200	0.26075784009444	0.05290290964305
O	1.46475677821929	-1.06741317476016	0.77409280538469
C	2.72720704969097	-0.97272785451801	0.42883221883537
C	4.86725510362606	-2.12149693930847	0.14216214103609
C	3.52218892672323	-2.14839474945504	0.47269470811067
C	-0.47470775489713	3.01413402031813	-0.58097291547394
C	0.81836204288242	3.07213163360724	0.26504010151388
H	-3.07449847120408	2.63166004865970	-0.23107018192351
C	-1.25897147910689	4.31921980638337	-0.47672040591734
H	2.06121957559432	4.10300665174691	-1.17163710313609
H	2.57659425225680	4.28760093736546	0.52547479547202
C	4.75157808102946	0.25106553918491	-0.25480198418618
H	3.03004645661343	-3.07817834597756	0.76595500590669
C	5.49726833074994	-0.91429634871850	-0.22590258741623
H	-5.04318159386248	1.72597382777302	0.42973117543458
H	5.44591299674049	-3.04872671020552	0.17195086201291
H	-3.15585773820214	-2.87849961499276	0.54911470619721
H	5.22655432248753	1.19927307283954	-0.52117334108686
H	-6.53964054116018	-0.19988455609754	0.90881452096346
H	6.55985514257275	-0.89892199388118	-0.47631472169539
H	3.35805336667423	2.41302729164106	-0.09171289078097
H	-5.58180068683663	-2.51617428861265	0.96305561567330
H	-0.16621792063621	2.86527296661509	-1.63221127845094
C	-0.37278414830013	5.50688879305020	-0.87242122308413
H	-2.14588295853445	4.28439224503829	-1.12751205718997
H	-1.61411036441195	4.44368353254780	0.56122175978455
C	0.90922699487403	5.55763169277536	-0.03838874098392
H	-0.11084128941167	5.42243478393549	-1.94199323012312
H	-0.94301546587480	6.44232139711836	-0.75839179003771
C	1.70077963792839	4.24834103867852	-0.13840718651014
H	1.54480026605755	6.39761323864527	-0.36046759171889

H	0.65073866021983	5.74198675679189	1.01946516688160
H	0.52064703139309	3.20954413906286	1.32005171186973
N	0.47061772951324	-0.11242731248970	-1.74727729924366
H	3.00951214631888	3.00951818320189	3.91032958133744
H	3.76782370054406	2.12028329053653	2.78497432552706
H	0.89655903826034	-1.03726264300937	-1.86246894562238
H	-0.39126843132734	-0.11867482652489	-2.30110582701145
H	1.10079390550639	0.56148352601051	-2.19215965357054
N	-0.70536291025512	0.71093724419545	3.22940983262071
N	2.91052918199621	2.65245747796050	2.95635929352645
H	2.17702150219148	1.94204680895145	3.01551563660151

^{2}[INT7]$^{+}$ + N_2

Ru	0.05112940373981	0.46279078833708	0.17885636249114
N	-3.25864011835358	0.90300918900138	3.51586401700482
C	-3.26707566352631	0.65203646522559	0.23612140874207
C	-4.66798723943101	0.80962762445902	0.41211539211513
C	-5.48550336009310	-0.24370367642856	0.77669491624101
C	-4.91164307371984	-1.52050895135849	0.95933306611160
C	-3.55504591914018	-1.72076914021822	0.77229734711162
C	-2.68349133861668	-0.65424860299035	0.41387833754711
C	-2.53381703275125	1.82740906300920	-0.16258020867373
N	-1.24431458140498	1.91451863476251	-0.28643595673732
O	-1.41654518872397	-0.92579143822103	0.24320543723674
N	1.39779336696505	1.92930122287535	0.01072295536309
C	2.68018822606826	1.75203841472532	-0.08593726338246
C	3.36511155045275	0.48842125616596	0.02284624260422
O	1.46914800548456	-0.87188313016508	0.71348114983683
C	2.73973253983471	-0.74567509522880	0.43003210044495
C	4.92986544048963	-1.84180789418539	0.29486229471997
C	3.57263571088658	-1.89255736720100	0.56254828631956
C	-0.56251872348055	3.12898587150032	-0.78140290660459
C	0.76061416851228	3.26052362285605	0.00478265714727
H	-3.14450864659775	2.70899037613007	-0.38826258375017
C	-1.36433004393683	4.42744537220372	-0.72708593823521
H	1.92205260955240	4.20365113950723	-1.55257423166549
H	2.51813339872241	4.52578552817813	0.08999846509258
C	4.76436150054470	0.50221691288594	-0.22495487631323
H	3.09434230599085	-2.82298240838101	0.87725316051832
C	5.54261786582224	-0.63458899682952	-0.10512269594249
H	-5.09485352588159	1.80505258035848	0.26053292417617
H	5.53252535408837	-2.74856277391118	0.39803183646414
H	-3.10691167807259	-2.70801122313920	0.90640661901871
H	5.22381837160571	1.45030558471876	-0.51914059568872
H	-6.55724480983303	-0.09196353253559	0.91971251011817
H	6.61477197696390	-0.59800757540953	-0.30889795832346
H	3.32227689502786	2.62622320047942	-0.24350250798894
H	-5.54419820792635	-2.36398781515530	1.24968651651041
H	-0.29290896246753	2.92451271566433	-1.83407455711338
C	-0.51766804835700	5.59980320579127	-1.23814475690341
H	-2.27652316473184	4.33831726537081	-1.33609667016571
H	-1.68017223406791	4.61385309425007	0.31442758252791
C	0.80087323519694	5.72378448084115	-0.47166687937414
H	-0.30338278243667	5.44939692739703	-2.31114026020350
H	-1.09678130659765	6.53345217847082	-1.15829942934798
C	1.60618238651563	4.41953049207505	-0.51688695531082
H	1.40780567137434	6.54654559787435	-0.88174686834341

H	0.58873608259548	5.97949296074755	0.58169852800191
H	0.48815700503406	3.47257106901278	1.05521412748690
N	0.38611784380320	-0.05006842790342	-1.84844140771275
H	-0.95218592487583	1.59746543076031	2.42267721766183
H	0.60057882398370	1.14909797043623	2.70332752920764
H	0.92460557503149	-0.91868834316546	-1.91460678080452
H	-0.49410580655600	-0.21840854976382	-2.34405295891598
H	0.89538705127739	0.65466747057887	-2.38909819585279
N	-4.00143788484469	0.17013299935171	3.86510629616692
N	-0.26401775999399	0.86367402352861	2.23434680767796
H	-0.59002266854558	0.02316723316039	2.71934086198749

^{2}[Ru(salen)(NH$_3$)(NH$_3$)]$^+$ (^{2}[I]$^+$)

Ru	-0.03135567451913	0.24380381348838	-0.02811000571606
N	-0.44320948109031	0.51278601698165	2.03253424837023
C	-3.33943297193684	0.51342124789668	-0.02500694775861
C	-4.73762590972889	0.69432743967305	0.15186211106127
C	-5.58841618155951	-0.36523080227219	0.40930581086918
C	-5.05243819923345	-1.66935407673277	0.47731684529684
C	-3.69823259537494	-1.88933318997206	0.29239220556651
C	-2.79266719704129	-0.81850498288110	0.04811250588431
C	-2.57140218522399	1.69420620240156	-0.33328681329708
N	-1.27877501431214	1.75279917650449	-0.43774325301199
O	-1.52894407466681	-1.10768330369175	-0.12425250638184
N	1.35215914607149	1.68994225472744	-0.06020042968391
C	2.63125445032756	1.48676038254672	-0.14786824410298
C	3.28320019084492	0.20094046045658	-0.11081679795013
O	1.34383326163453	-1.14392788274729	0.47533818726766
C	2.62064491633417	-1.03809557491081	0.21165517730496
C	4.77920124544677	-2.18557313305804	0.02071777613164
C	3.41890157390449	-2.21441619043995	0.27690007017773
C	-0.55525731581902	2.97493713992671	-0.84511937254679
C	0.74568715787445	3.03412790284832	-0.01266967081348
H	-3.15569422216434	2.60512123909328	-0.50662236279908
C	-1.32906001809174	4.28758505784302	-0.74985233737825
H	1.97473610923848	4.02565195853839	-1.48522731122549
H	2.52736503184302	4.25308374669989	0.18787374509290
C	4.68412397714260	0.19112762598687	-0.34717929833043
H	2.91178844864383	-3.14915075277664	0.52719796740843
C	5.42889317311600	-0.97342344315157	-0.29656959394306
H	-5.13647708842091	1.71025862740496	0.08059910331308
H	5.35480910222321	-3.11430358165104	0.06751352737795
H	-3.27929663789321	-2.89719033735329	0.33934173083199
H	5.17267735208817	1.14246576887207	-0.57696218483472
H	-6.65809246986211	-0.19718281557714	0.55032381222616
H	6.50307252709471	-0.95498045794039	-0.49185458815570
H	3.29698636423054	2.35221824004446	-0.24256540501830
H	-5.71249603136621	-2.51832480365443	0.67628877099957
H	-0.25564527637345	2.81755045282173	-1.89791779157305
C	-0.44059003820171	5.46260062697954	-1.17676742533408
H	-2.22453312860224	4.24993337572908	-1.38857701724377
H	-1.67169505616205	4.43173301189055	0.28987138573346
C	0.85490253730939	5.51673086806764	-0.36411257785277
H	-0.19576646232088	5.35963663764997	-2.24883876192553
H	-1.00053262639859	6.40506887428966	-1.06873061441092
C	1.63195617730307	4.19744029033568	-0.44986960255982
H	1.49351574718086	6.34403570123076	-0.71220330814298

H	0.61427157637891	5.72430118170330	0.69367985035862
H	0.44599970450526	3.19796784571639	1.03892015014288
N	0.37341724227404	-0.13789853616219	-2.07165841517219
H	0.77411913105045	-1.07235006129572	-2.19289558049977
H	1.02827789217720	0.52207868932166	-2.50064918865983
H	-0.47869231392431	-0.11256962976886	-2.63912454259752
H	-0.95971632395023	-0.28861169754955	2.40598684837216
H	0.41663521577817	0.58473146251353	2.58410388914714
H	-0.99736685857797	1.34658860030260	2.24703738848547

[1][Ru(salen)(NH_2)(NH_3)]$^+$ ([1][II]$^+$)

Ru	-0.00647365763163	0.23682342556395	0.10239304598803
N	-0.19691644166738	0.45501433939115	1.92633671042648
C	-3.31054241398125	0.45583837512928	-0.04503731692200
C	-4.72511683047066	0.63650930617617	-0.00233477783520
C	-5.58917514160955	-0.40303049823094	0.26656716563339
C	-5.05461578497686	-1.69493161824740	0.49112193798930
C	-3.69315918374465	-1.91888063732604	0.44707036484049
C	-2.76367123371458	-0.86558390353888	0.18675876339511
C	-2.54186731613885	1.61891362617877	-0.37011323037466
N	-1.24309791217052	1.71928838552024	-0.38695419427271
O	-1.50170111682012	-1.16260256896878	0.15890679531642
N	1.38678205003926	1.68131592282199	-0.00568680382106
C	2.66334051109453	1.48708470698107	-0.05340409382538
C	3.32058824144216	0.19897196274360	-0.03616810050591
O	1.32784639422159	-1.21021943579831	0.01620127969218
C	2.63798719095171	-1.06283370230236	-0.02647790848098
C	4.80306021573642	-2.19817604105213	-0.08541342950452
C	3.41678357611431	-2.24603637210744	-0.06906323856959
C	-0.52626288338440	2.93767990150350	-0.82269509116111
C	0.77253176188592	3.02474559536744	0.00234927849757
H	-3.12586698681201	2.51007199604242	-0.62659583901646
C	-1.31374333143582	4.24434736101880	-0.74833619944744
H	1.99000101047718	3.98626877555876	-1.49847242790978
H	2.54893840125053	4.25661856269915	0.16647790299752
C	4.73703304559461	0.20886678091547	-0.06797586712840
H	2.88288002516809	-3.19867265804658	-0.07274047576108
C	5.47768044923327	-0.96267176626301	-0.08414801437508
H	-5.11787395050172	1.63993346253369	-0.18883949538541
H	5.37267040228238	-3.13116842036076	-0.10221118674417
H	-3.27924364083936	-2.91503245439950	0.61822720407455
H	5.24517355124937	1.17698820098931	-0.07888792102411
H	-6.66752128120096	-0.23612278623389	0.30273855235274
H	6.56878183508835	-0.92675176851689	-0.10142135135685
H	3.32765526338748	2.35598811661937	-0.11595310601742
H	-5.72914591790813	-2.52928594333428	0.70318009436451
H	-0.23240358653579	2.76653455045523	-1.87485972698939
C	-0.43601581822911	5.41711859875778	-1.20342642478463
H	-2.20940537018255	4.18845727014546	-1.38492710174272
H	-1.65408955615211	4.40312098901920	0.28987945466101
C	0.86614987236591	5.49713240767780	-0.40380071021919
H	-0.19997414872983	5.29552190795991	-2.27540811798772
H	-1.00293130600324	6.35668925930425	-1.10837289159148
C	1.65148964339758	4.18102245570552	-0.46580803439708
H	1.49653782298456	6.31937726188797	-0.77763019204528

H	0.63394484745668	5.72815098782630	0.65090643612882
H	0.47708960747567	3.21333503401354	1.05071581970516
N	0.01478917093984	-0.07794470790134	-2.08461865978685
H	0.08991935902581	-1.08122610825883	-2.26848633576240
H	0.80275274372096	0.37206921330121	-2.55811643818760
H	-0.83395324900349	0.24574573600938	-2.55501050530767
H	-0.66321093154858	-0.25508504765553	2.49832942461334
H	0.06504563150898	1.27832666812507	2.47461924146473

^{2}[Ru(salen)(NH)(NH$_3$)]$^+$ (^{2}[III]$^+$)

Ru	-0.04856072056440	0.30831465811483	0.24354325525292
N	-0.08732466344155	0.52998140513191	1.96686155685373
C	-3.35096921736738	0.50007053660941	-0.02386981935959
C	-4.76381167374556	0.63352653279749	0.03317218851147
C	-5.59184719931423	-0.46318801822241	0.18922259703580
C	-5.01535112697816	-1.74745425178813	0.26560543979136
C	-3.64111014176346	-1.91808672071158	0.19303357530210
C	-2.76703008196615	-0.80924724268148	0.06415955028944
C	-2.59735253470243	1.69753843771260	-0.28253952260925
N	-1.30588292870389	1.80133029685471	-0.27931171159597
O	-1.47617558007458	-1.06002585123157	0.01253113285343
N	1.35487510256364	1.72299486691945	-0.06708903962614
C	2.62238243148135	1.47621917889075	-0.17910505485746
C	3.26092357669284	0.19113693189063	-0.09474965629474
O	1.30256580052955	-1.19449662436236	0.37381183691419
C	2.58747216684691	-1.05497898049464	0.17943951228452
C	4.75366666772020	-2.19351434205060	0.03507408085904
C	3.38421553024057	-2.23174894053491	0.23239740019054
C	-0.58942118836177	3.00359297834814	-0.76452693622737
C	0.76478476793277	3.07315026163681	-0.03585321972344
H	-3.18928785579672	2.58561309953695	-0.53341841824692
C	-1.34473925282316	4.32620256863256	-0.64800441495197
H	1.88448938571397	3.98512650988114	-1.64028793520394
H	2.57631719566138	4.26153311379933	-0.02593648890448
C	4.67080394121971	0.18621859774140	-0.29091369710951
H	2.86927745120104	-3.17309900410747	0.43585953443787
C	5.41553870934756	-0.97547446741544	-0.23042718907541
H	-5.19050730987654	1.63646011472980	-0.05225843559036
H	5.32726204944654	-3.12314659990992	0.08590137795571
H	-3.19137965863393	-2.91167809508094	0.24506323124422
H	5.16255380895132	1.14146287951659	-0.49389436726993
H	-6.67511468764028	-0.33792529238809	0.24069620338489
H	6.49598505086124	-0.95276739189796	-0.38406421756766
H	3.29476832054490	2.32510437675464	-0.35002098637913
H	-5.65785476220556	-2.62478134803016	0.37939135438539
H	-0.37264578188539	2.81586164913385	-1.83250044220169
C	-0.47872332076702	5.47571728204669	-1.17961245465407
H	-2.28447686816140	4.28486347207242	-1.21852531171521
H	-1.60597870750677	4.49864445017740	0.41057420965616
C	0.88242539413357	5.53534336382028	-0.48328900581526
H	-0.32870427884182	5.33934026891108	-2.26519082710328
H	-1.01737326738751	6.42779917201843	-1.05127009211196
C	1.63190907696072	4.20131307068947	-0.58756408285889
H	1.49979848051026	6.33799356422498	-0.91643998536140

H	0.73841677193629	5.78446899994901	0.58284448841457
H	0.55093713090743	3.27778833103345	1.02972660084353
N	0.21266321450929	-0.09052202271014	-1.96472371878508
H	0.19894143855686	-1.10416726650110	-2.10008932951049
H	1.08788555917190	0.25174102608957	-2.36830777333104
H	-0.54462914373006	0.29414617198927	-2.53576124592873
H	0.33227343289788	-0.09274750693624	2.67893541020948

References

[1] Man, W. L.; Tang, T. M.; Wong, T. W.; Lau, T. C.; Peng, S. M.; Wong, W. T. Highly Electrophilic (Salen)ruthenium(VI) Nitrido Complexes. *J. Am. Chem. Soc.* **2004**, *126*, 478–479.

[2] CrysAlis, Oxford Diffraction Ltd., version 1.171.41.123a, **2022**.

[3] Sheldrick, G. M. SHELXT-Integrated Space-Group and Crystal-Structure Determination. *Acta Cryst*. **2015**, *A71*, 3-8.

[4] Sheldrick, G. M. Crystal Structure Refinement with SHELXL. *Acta Cryst*. **2015**, *C71*, 3–8.

[5] Dolomanov, O. V.; Bourhis, L. J.; Gildea, R. J.; Howard, J. A. K.; Puschmann, H. OLEX2: A Complete Structure Solution, Refinement and Analysis Program. *J. Appl. Cryst*. **2009**, *42*, 339–341.

[6] Olex2 1.5 - compiled 2022.04.07 svn. rca3783a0 for OlexSys, GUI svn.r6498.

[7] Kratzert, D., Krossing, I. Recent improvements in DSR. *J. Appl. Cryst*. **2018**, *51*, 928–934.

[8] (a) Li, Y.; Chen, J. Y.; Miao, Q.; Yu, X.; Feng, L.; Liao, R. Z.; Ye, S.; Tung, C. H.; Wang, W. A Parent Iron Amido Complex in Catalysis of Ammonia Oxidation. *J. Am. Chem. Soc*. **2022**, *144*, 4365–4375. (b) Zott, M. D.; Peters, J. C. Enhanced Ammonia Oxidation Catalysis by a Low Spin Iron Complex Featuring *Cis* Coordination Sites. *J. Am. Chem. Soc*. **2021**, *143*, 7612–7616.

[9] (a) Lee, D. G.; Lee, E. J.; Chandler, W. D. Oxidation of Hydrocarbons. 16. Mechanism of the Reaction between Alkynes and Permanganate Ion. *J. Org. Chem*. **1985**, *50*, 4306–4309. (b) Chandler, W. D.; Lee, E. J.; Lee, D. G. Computer-Assisted Analysis of Reaction Rate Data. *J. Chem. Ed*. **1987**, *64*, 878–881. (c) Yiu, S.-M.; Lam, W. W. Y.; Ho, C.-M.; Lau, T.-C. Facile N…N Coupling of Manganese(V) Imido Species. *J. Am. Chem. Soc*. **2007**, *129*, 803–809.

[10] (a) Neese, F. Software Update: The ORCA Program System, Version 4.0. WIREs Comput. Mol. S128 Sci. **2018**, *8*, 4–9. (b) Neese, F. Software Update: The ORCA Program System—Version 5.0. WIREs Comput. Mol. Sci. **2022**, *12*, 1–15.

[11] Neese, F.; Wennmohs, F.; Hansen, A.; Becker, U. Efficient, Approximate and Parallel Hartree– Fock and Hybrid DFT Calculations. A 'Chain-of-Spheres' Algorithm for the Hartree–Fock Exchange. *Chem. Phys*. **2009**, *356*, 98–109.

[12] (a) Barone, V.; Cossi, M. Quantum Calculation of Molecular Energies and Energy Gradients in Solution by a Conductor Solvent Model. *J. Phys. Chem. A* **1998**, *102*, 1995–2001. (b) Andreussi, O.; Dabo, I.; Marzari, N. Revised Self-Consistent Continuum Solvation in Electronic-Structure Calculations. *J. Chem. Phys*. **2012**, *136*, 064102.

[13] Kruse, H.; Grimme, S. A Geometrical Correction for the Inter- and Intra-Molecular Basis Set Superposition Error in Hartree-Fock and Density Functional Theory Calculations for Large Systems. *J. Chem. Phys*. **2012**, *136*, 154101.

[14] (a) Nakajima, K.; Toda, H.; Sakata, K.; Nishibayashi, Y. Ruthenium-Catalysed Oxidative Conversion of Ammonia into Dinitrogen. *Nat. Chem*. **2019**, *11*, 702–709. (b) Nyhlén, J.; Duan, L.; Åkermark, B.; Sun, L.; Privalov, T. Evolution of O2 in a Seven-Coordinate Ru^{IV} Dimer Complex with a [HOHOH]− Bridge: A Computational Study. *Angew. Chem. Int. Ed*. **2010**, *49*, 1773–1777. (c) Tong, L.; Duan, L.; Xu, Y.; Privalov, T.; Sun, L. Structural Modifications of Mononuclear Ruthenium Complexes: A Combined Experimental and Theoretical Study on the Kinetics of Ruthenium-Catalyzed Water Oxidation. *Angew. Chem. Int. Ed*. **2011**, *50*, 445–449. (d) Song, D.; Chen, J.; Wang, R.; Guo, Z.; Xie, J.; Liu, Y. Catalytic Water Oxidation by

Ruthenium Complexes with Pyridine Alkoxide Ligands. *Eur. J. Inorg. Chem*. **2024**, *27*, e202300536. e) Feng, S.; Chen, J.; Wang, R.; Li, H.; Xie, J.; Guo, Z.; Lau, T.-C.; Liu, Y. Dual Pathways in Catalytic Ammonia Oxidation by a Ruthenium Complex Bearing a Tetradentate Bipyridine–Bipyrazole Ligand: Isolation of a Diruthenium Intermediate with a μ-Hexazene Derivative. *J. Am. Chem. Soc*. **2024**, *146*, 21490−21495.

[15] Man, W. L.; Lam, W. W. Y.; Kwong, H. K.; Peng, S. M.; Wong, W. T.; Lau, T. C. Reaction of a (Salen)ruthenium(VI) Nitrido Complex with Thiols. C−H Bond Activation by (Salen)ruthenium(IV) Sulfilamido Species. *Inorg. Chem*. **2010**, *49*, 73–81.
